# A Neutral PC_NHC_P Co(I)–Me Pincer Complex as a Catalyst for *N*-Allylic Isomerization with a Broad Substrate Scope

**DOI:** 10.1021/acs.joc.3c02349

**Published:** 2024-03-23

**Authors:** Sakthi Raje, Tofayel Sheikh Mohammad, Graham de Ruiter

**Affiliations:** Schulich Faculty of Chemistry, Technion—Israel Institute of Technology, Technion City, 3200008 Haifa, Israel

## Abstract

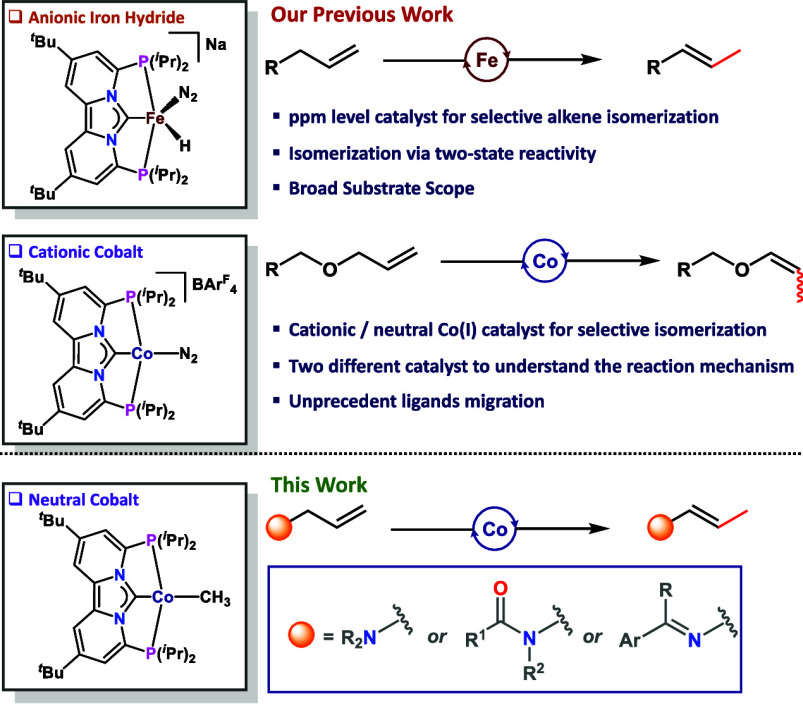

Earth-abundant-metal catalyzed double bond transposition offers a sustainable and atom-economical route toward the synthesis of internal alkenes. With an emphasis specifically on internal olefins and ethers, the isomerization of allylic amines has been particularly under represented in the literature. Herein, we report an efficient methodology for the selective isomerization of *N*-allylic organic compounds, including amines, amides, and imines. The reaction is catalyzed by a neutral PC_NHC_P cobalt(I) pincer complex and proceeds via a π-allyl mechanism. The isomerization occurs readily at 80–90 °C, and it is compatible with a wide variety of functional groups. The in situ formed enamines could additionally be used for a one-pot inverse-electron-demand Diels–Alder reaction to furnish a series of diversely substituted heterobiaryls, which is further discussed in this report.

## Introduction

**Figure 1:**
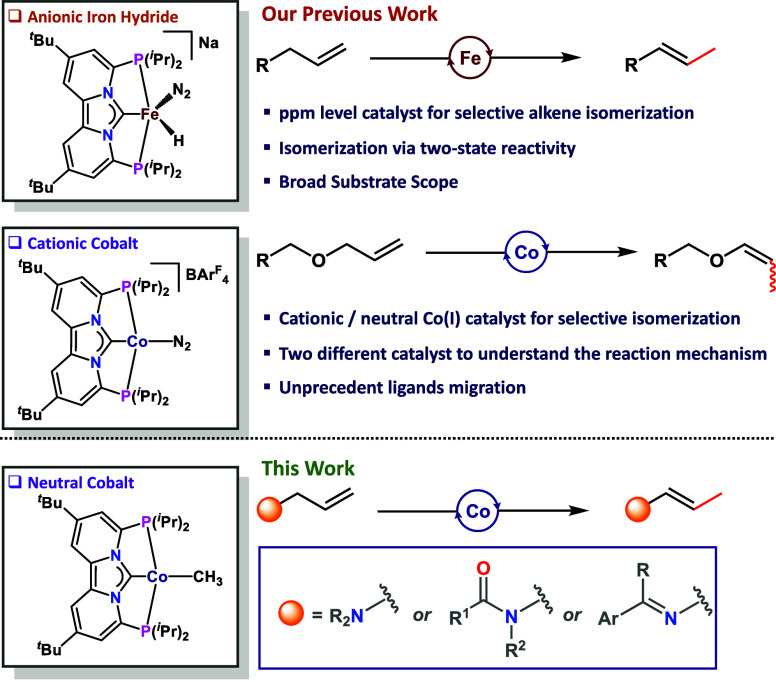
Reactivity comparison between iron(0) and cobalt(I) PC_NHC_P pincer complexes active for olefin isomerization.

Alkenes are ubiquitous in a wide variety of natural and industrial products. The selective transposition of terminal carbon–carbon bonds to internal ones has been investigated for decades, mainly with precious metal catalysts (e.g., Pd, Ru, and Ir).^1^ Recently, significant efforts have been made to replace these precious metals with their earth-abundant congeners, such as iron, cobalt, and nickel.^2^ Using these metals has resulted in hallmark examples of earth-abundant-metal-catalyzed double bond migration (Figure 1), where the emphasis has mainly been on olefins and allyl ethers.^1a,1d,2a,3^ By contrast, double bond migration from an *N*-allyl motif has been underrepresented in the literature despite its presence in a variety of natural products, agrochemicals, and industrially relevant compounds.^3a,4^

The isomerization of *N*-allylic framework enables a selective and atom-economical pathway to highly polarized *N*-(1-propenyl) or generally *N*-vinyl intermediates,^3a^ whose enamines, enamides, and aza-dienes are commonly used in cycloadditions,^5^ cyclopropanations,^6^ heterocycle synthesis,^7^ halofunctionalizations,^8^ and transition-metal-catalyzed C–C bond-forming reactions.^9^ In addition, the transition-metal catalyzed tandem isomerization of *N*-allylic double bonds followed by functionalization of the in situ formed *N*-vinyl intermediate offers access to functionalized molecules that would be difficult to synthesize otherwise.^10^ Furthermore, the added benefit of *N*-allyl isomerization is that in these reactions the regio- and stereoselectivity are often well-defined.^3a,11^

Because of their synthetic utility, Otsuka and co-workers reported in the 1980s the first Co(I)-hydride catalyzed isomerization of two allylamines to their corresponding *trans*-enamines.^12^ Stille, on the other hand, demonstrated the ruthenium-, rhodium-, and iron catalyzed isomerization of allylamides to enamides, although different reaction conditions were necessary for each metal.^13^ Later, the scope and stereoselectivity were greatly improved by Krompiec and co-workers who used noble-metal containing catalysts.^3a,14^ Following these early examples, several recent studies have reported the stereoselective isomerization of allyl amines and allyl amides.^1a,4a,4b,15^ Most notably, Trost and co-workers reported the isomerization of highly substituted *N*-allylamides to *Z*-enamides by utilizing a cationic ruthenium catalyst,^16^ while Schoenebeck and co-workers used an air-stable Pd(I) dimer for the *E*-selective synthesis of enamides.^17^ Recently, a new strategy by Matsunaga and co-workers was reported, who elegantly demonstrated that poly-substituted enamides could be synthesized via Co-catalyzed hydrogen atom transfer-mediated alkene isomerization.^18^ Besides these hallmark examples, there are only a few studies that report the transition-metal-catalyzed isomerization of allyl imines to azadienes,^19^ which is an interesting building block for the cycloaddition reactions. Overall, most of these reactions are catalyzed by precious metals, leaving ample opportunity to develop earth-abundant alternatives. Furthermore, no universal strategy has been developed that allows the isomerization of general *N*-allylic substrates such as allylamines, allylamides, and allylimines with a single catalyst, again leaving ample chemical space for such protocols to be developed.

Recently, our group reported efficient alkene isomerization catalyzed by well-defined iron(0) and cobalt(I) PC_NHC_P pincer complexes that proceeded either by an alkyl- (Fe) or allyl-type (Co) mechanism (Figure 1).^20^ Building upon the success of these isomerization catalysts, herein we report that the cobalt PC_NHC_P pincer complex [(PC_NHC_P)CoCH_3_] (**Co–Me**) is an excellent catalyst for the selective isomerization of allylamines, allylamides, allyl-aldimines, and allyl-ketimines (Figure 1).^21^ In addition, the resulting enamines were used in a one-pot sequential procedure for the inverse-electron-demand Diels–Alder reaction that enables facile synthesis of diversely substituted heterobiaryls, which is further discussed in this report.

## Results and Discussion

**Scheme 1:**
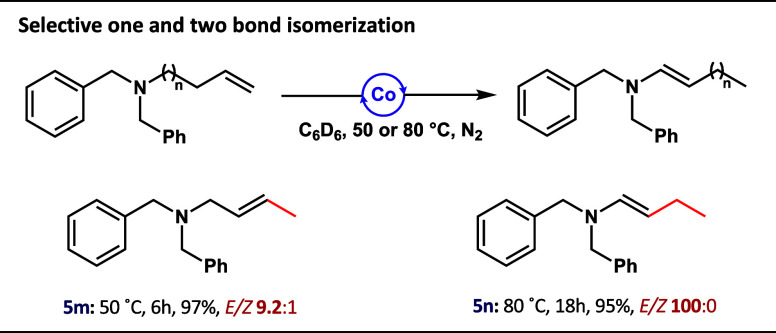
Selective One and Two Bond Isomerization of Terminal Alkene Catalyzed by Co(I)–Me Complex

Given our previous experiences in alkene isomerization and the availability of well-defined cobalt(I) PC_NHC_P pincer complexes, we sought to establish if [(PC_NHC_P)CoMe] (**Co–Me**) could efficiently isomerize *N*-allylic substrates. To the best of our knowledge, there has been only one report on cobalt-catalyzed isomerization of allylamines,^12^ while no universal protocol is available to isomerize all three sets of *N*-allylic substates. We started our investigation into *N*-allylic isomerization with **Co–Me** as the catalyst (5 mol %), *N*,*N*-dibenzylallylamine as a model substrate, and toluene-*d*_8_ as the solvent at 80 °C. Gratifyingly, the allylamine completely isomerized to the corresponding enamine with exceptional stereoselectivity (*E*/*Z*: 37:1). A short optimization protocol revealed that the resulting enamine could also be obtained in excellent yields with 2 mol % of catalyst (Table S1). Using the optimized conditions, we explored a diverse set of electronically or sterically differentiated allylamines (Table 1). As evident from Table 1, allylamines substituted with alkyl, aryl, cycloalkyl, heterocycles, diallyl, and triallyl substituents are all well tolerated, and their isomerization proceeded smoothly with excellent stereoselectivity. Sterically encumbered substrates such as *N*,*N*-dicyclohexyl or *N*,*N*-diphenyl allylamines, or a combination thereof, all provided the corresponding enamines (**5f**–**5h**) in excellent yield, although slightly higher temperatures were required for isomerization of *N*,*N*-diphenyl allylamine. Interestingly, heteroatom-substituted allylamines were also well tolerated (**5j**–**5l**), and the isomerization proceeded with complete conversion, although the isolation of the resulting enamine resulted in somewhat moderate yields. Interestingly, the methodology reported herein is not limited to single-bond isomerization. The neutral Co–Me complex is also an excellent catalyst for multiple bond isomerization. While at 50 °C single-bond isomerization was observed, at 80 °C selective two-bond isomerization products were obtained (**5m** and **5n**, Scheme 1).

**Table 1:**
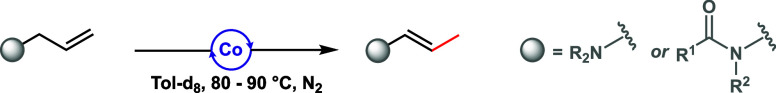

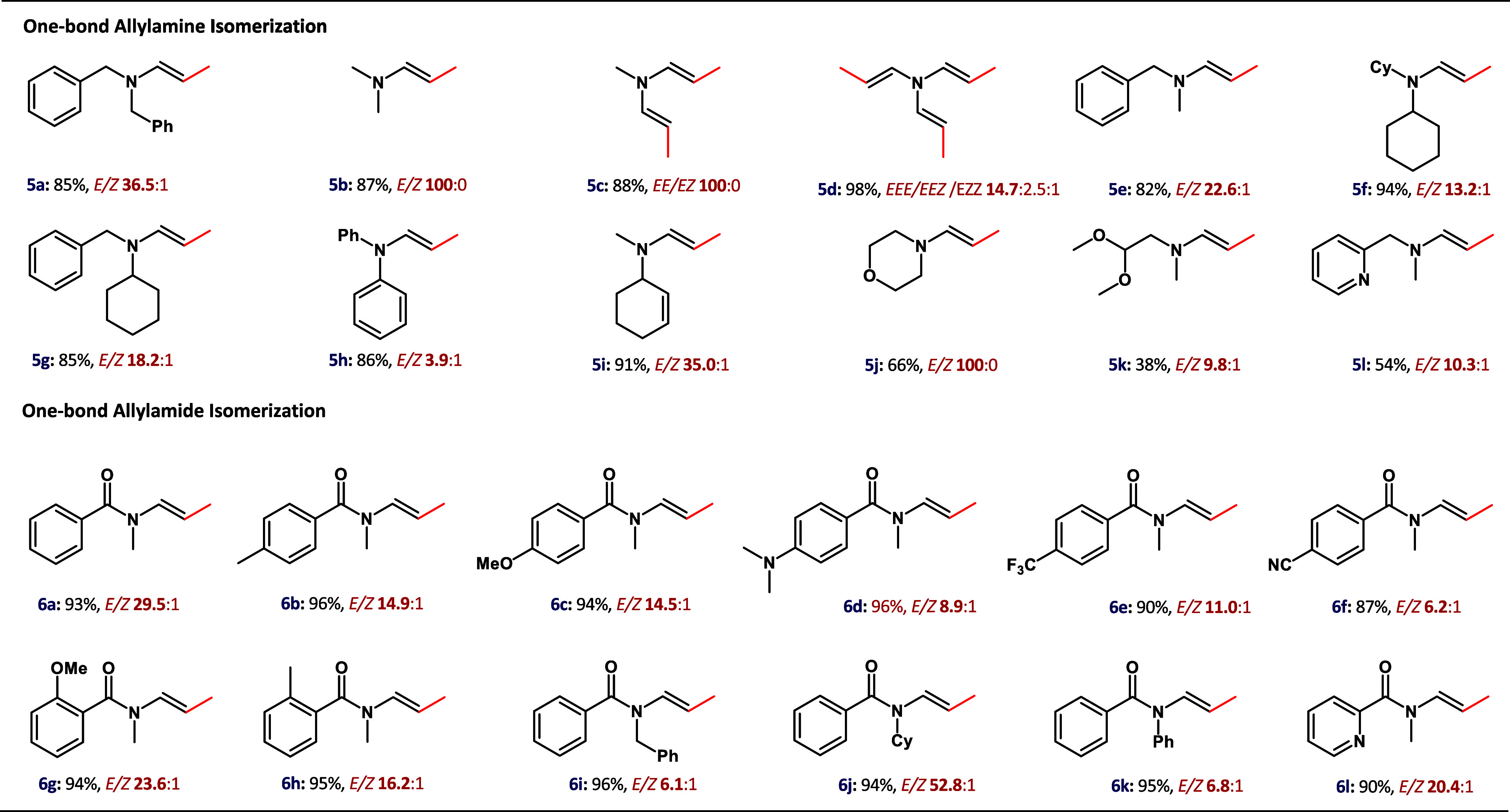
Isomerization of *N*-Allylamines and *N*-Allylamides Catalyzed a Neutral Co(I)–Me Catalyst

a Reactions were performed with 2–5 mol % catalyst, 0.15 mmol substrate, in 400 μL of toluene-*d*_8_ for 6–24 h at 80–90 °C. Yields and stereoselectivity (*E* vs *Z*) were determined by ^1^H and ^13^C NMR spectroscopy.

Besides enamines, we were also interested if **Co–Me** could be used to isomerize *N*-allylamides, since the resulting enamides are extensively utilized in various organic transformations.^5c,5d,22^ Although several methods are available for their synthesis,^23^ transition-metal-catalyzed isomerization is one of the most convenient and atom-economical routes.^4a,4b,16−18^ Consequently, we set out to test the isomerization of *N*-allylamides with our previous established reaction protocol (Table 1). Gratifyingly, the isomerization of *N*-allyl-*N*-methylbenzamide proceeded readily at 80 °C and produced the corresponding enamide with excellent stereoselectivity (Table 1; **6a**). Changing the nature of the benzamide to include electron-donating (e.g., −Me, −OMe, or −NMe_2_) or electron-withdrawing substituents (e.g., −CN or −CF_3_) did not affect the yield nor stereoselectivity of the reaction (Table 1; **6b**–**6f**). Likewise, changing the substituent pattern at the arene-ring did not affect yield or stereoselectivity (Table 1; **6g** and **6h**). To investigate how steric parameters influence the isomerization reaction, we modified the *N*-methyl substituent to either benzyl, phenyl, or cyclohexyl. In all cases, the corresponding enamides (**6i**–**6k**) were obtained in good yields (>94%) with moderate to excellent *E*-stereoselectivity (*E*/*Z* ≥ 6:1). Even *N*-allyl-*N*-methyl-picolinamide could be isomerized with excellent *E*-selectivity (Table 1; **6l***E*/*Z*: 20.4:1). These results demonstrate that our recently reported **Co–Me** complex is an excellent catalyst for the stereoselective isomerization of *N*-allylamines and *N*-allylamides.

Driven by the successful isomerization of these substrates, we sought to provide easy access to 1,3-azadienes via the isomerization of *N*-allylimines. While useful substrates in organic syntheses, accessing the 1,3-azadiene motif is difficult and frequently relies on base-mediated isomerization of allylimines that proceeds with poor yields and selectivity.^24^ Recently, a different route was reported by Trost and co-workers who accessed the azadiene via a palladium-catalyzed oxidative allylic alkylation.^25^ To the best of our knowledge, there has been no report on first-row transition-metal-catalyzed one-bond isomerization of *N*-allylimines.

**Scheme 2:**
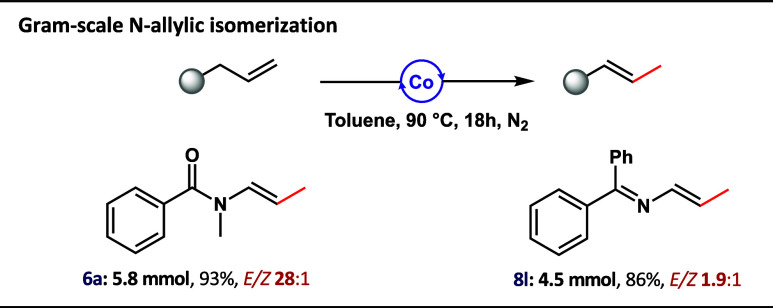
Gram-Scale *N*-Allylic Isomerization Catalyzed by Co(I)–Me Complex

To test the isomerization of *N*-allylimines, we selected phenyl aldimine as a benchmark substrate with **Co–Me** as a catalyst. Using the optimized reaction conditions (vide supra), the corresponding 2-aza-1,3-diene (**7a**) was obtained in a 94% yield. Compared to the isomerization of *N*-allylamines and amides, *E*-stereoselectivity is only moderate (*E*/*Z* = 2.2:1). Further exploring the substrate scope revealed that electronically differentiated phenyl aldimines are isomerized efficiently, where both electron-donating (e.g., −Me, −OMe, and −NMe_2_) or electron-withdrawing (e.g., −CN or −CF_3_) substituents are well tolerated (Table 2; **7b**–**7f**). Furthermore, *ortho* substitution on the phenyl ring (**7g**) did not impede the transformation. Similarly, the trisubstituted aryl (**7j**) and 1-naphthyl (**7k**) allylimines were also tolerated, albeit longer reaction times were necessary to obtain complete conversion of the substrate. To our delight, nonaromatic (**7l**) and heteroaromatic (**7h**, **7i**) allylimines were efficiently isomerized to the corresponding 2-aza-1,3-dienes in good to moderate yields. Finally, we were also able to extend this methodology to include *N*-allylketimines. Akin to their imine congeners, similar yields and stereoselectivities were obtained (Table 2; **8a**–**8l**), although slightly higher temperatures (90 °C) were required to complete the reaction. Finally, to demonstrate the applicability and scalability of the herein reported *N*-allylic isomerization protocol, the gram-scale synthesis of **6a** and **8l** was demonstrated (Scheme 2).

**Table 2:**
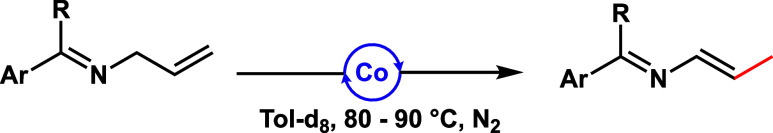

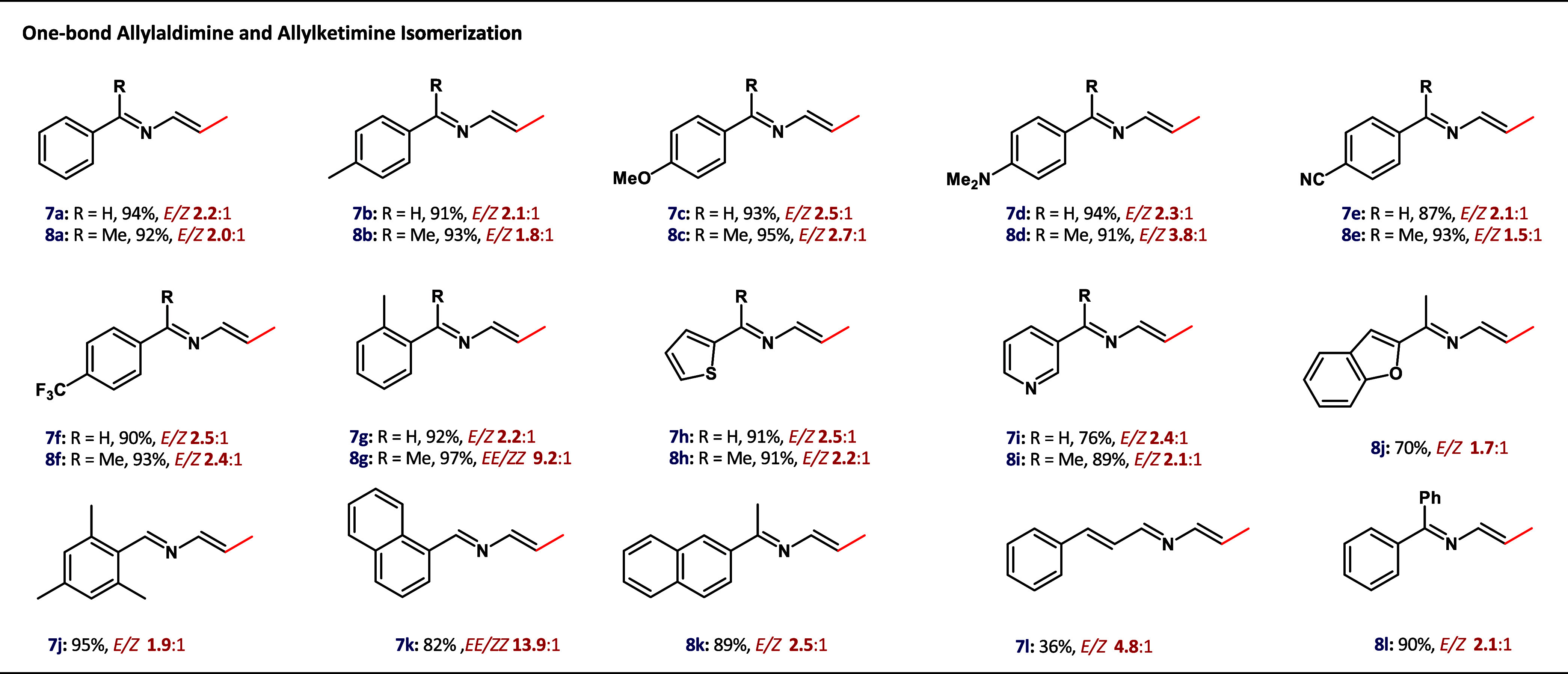
Isomerization of *N*-Allylaldimines and *N*-Allylketimines Catalyzed by a Neutral Co(I)–Me Catalyst^a^

a Reactions were performed with 5 mol % catalyst, 0.15 mmol substrate, in 400 μL of toluene-*d*_8_ for 6–24 h at 80–90 °C. Yields and stereoselectivity (*E* vs *Z*) were determined by ^1^H and ^13^C NMR spectroscopy.

Overall, the methodology reported herein is applicable for the isomerization of allyl (i) amines, (ii) amides, and (iii) imines, which can also be extended to one- and multiple-bond isomerization strategies. Although a wide scope of substrates are tolerated (vide supra), any substitution on the allyl-fragment results in a complete loss of catalytic activity, most likely due to steric crowding around the metal center.^20a^ Current research is centered around enabling the isomerization of *N*-allylic di-, tri-, and tetra-substituted alkenes that bear great synthetic relevance.

**Scheme 3:**
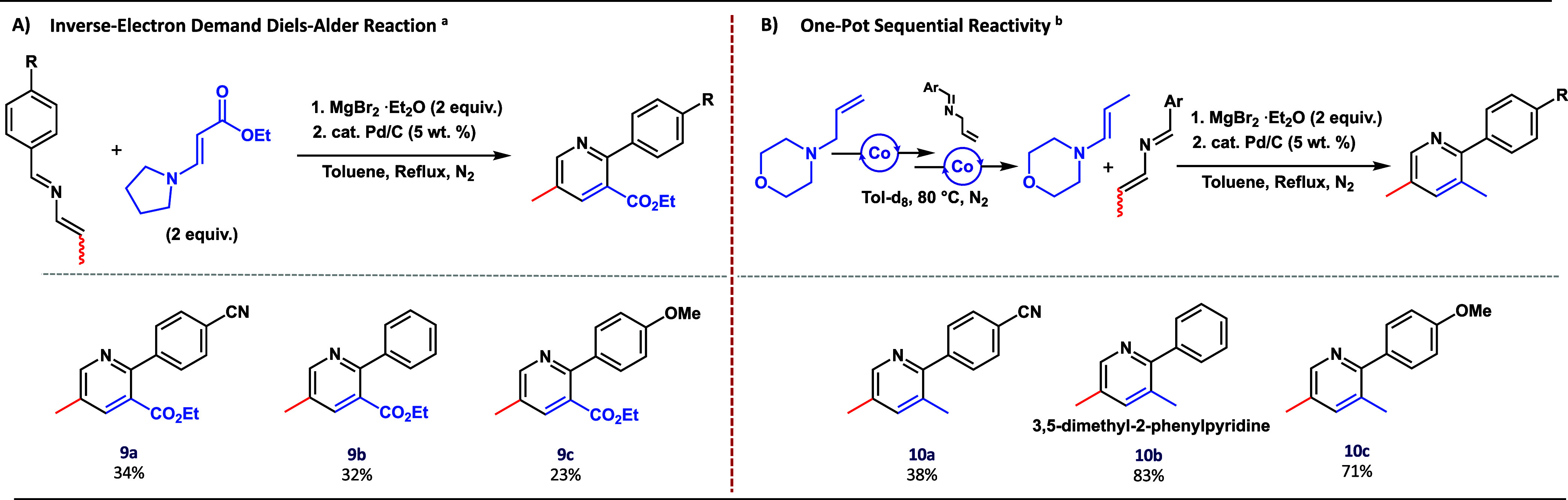
(A) Inverse Electron-Demand [4 + 2] Diels–Alder Cycloaddition for Hetero-Aryl Synthesis (B) One-Pot Sequential Reactivity for Hetero-Aryl Synthesis Reactions were performed with 0.25 mmol azadiene, 0.5 mmol enamine ester, and 23 mol % Pd/C (5 wt %, based on metal). Reactions were performed with 2.5 mol % catalyst, 0.25 mmol amine, 0.3 mmol imine, and 23 mol % Pd/C (5 wt %, based on metal). More synthetic details can be found in Supporting Information.

Considering the importance of 2-aza-1,3-dienes as substrates in organic chemistry, the isomerized products can be readily converted into other six-membered heterocycles^25^ via an inverse-electron-demand Diels–Alder cycloaddition (Scheme 3A). The one-step formation of pyridine-containing motifs would be a valuable asset in the synthesis of natural products and pharmaceuticals. We performed this cycloaddition with electron-deficient 2-aza-1,3-diene **7a** and ethyl 3-(pyrrolidin-1-yl)acrylate (**11**) in the presence of MgBr_2_·Et_2_O as a promotor. Subsequent oxidation with catalytic amounts of Pd/C (23 mol %; 5 wt %, based on metal) resulted in the formation of various heterobiaryls as single regioisomers in low to moderate yield (**9a**–**9c**). Note that in the study by Trost and co-workers, similar yields were obtained for a multistep synthesis. Realizing that enamine coupling partners could also be accessed via our isomerization protocol, we envisioned developing a one-pot procedure where both the 2-aza-1,3-diene and the enamine starting materials are obtained via our cobalt-catalyzed isomerization protocol. To test the one-pot cycloaddition, *N*-allyl morpholine and phenyl aldimine were mixed in a J-Young tube, and the reaction was heated at 80 °C with 5 mol % **Co–Me** catalyst. Unfortunately, only the phenyl aldimine was completely converted to the 2-aza-1,3-diene, with less than 5% conversion of the *N*-allylamine. Even increasing the reaction time and catalyst loading did not improve the conversion of *N*-allylamine to the corresponding enamine. Most likely, strong coordination of 2-aza-1,3-diene to the cobalt metal centers prevents further isomerization of the *N*-allylamine. Indeed, when first *N*-allyl morpholine was added to a mixture of **Co–Me** in toluene-*d*_8_, complete isomerization was observed, as reported in Table 1. Subsequent addition of the *N*-allylaldimine resulted in quantitative formation of 2-aza-1,3-diene, as judged by ^1^H NMR spectroscopy. With both substrates now available through cobalt-catalyzed isomerization, a sequential one-pot procedure was developed for the synthesis of diversely substituted 2-phenylpyridines (Scheme 3B).

To illustrate, in a one-pot procedure, *N*-allyl morpholine was isomerized with a 5 mol % Co–Me catalyst at 80 °C. Subsequent addition of the aryl aldimine to the same reaction mixture resulted in the formation of the 2-aza-1,3-diene product. To facilitate the Diels–Alder reaction, MgBr_2_·Et_2_O was added, followed by catalytic amounts of Pd/C (23 mol %; 5 wt %, based on metal), to furnish the desired pyridine-biaryl as a single regio-isomer as the product (Figures S150–151). This methodology is widely applicable and can be used to access both electron-rich and electron-poor 2-phenylpyridines (**10a**–**10c**) in moderate to excellent yields (Scheme 3B).

Mechanistically, our previous studies have shown that the isomerization reaction occurs via a π-allyl mechanism.^20b^ We envisioned that such a mechanism is also operable for the isomerization of *N*-allyllic substrates to generate the respective *N*-vinyl products. However, in the case of *N*-allylimines, two intermediates are possible during the isomerization process: (i) an all-carbon-π-allyl Co(III) complex and (ii) a 2-aza-π-allyl Co(III) complex that are most likely in equilibrium. Our experiments indicate that for the *N*-allylimines, 2-aza-1,3-dienes are the sole product of the reactions with no trace of the 1-azadienes, which suggests that the reaction follows through the all-carbon-π-allyl Co(III) intermediate.

## Conclusions

In conclusion, we have established the versatility of the neutral **Co(I)–Me** complex as an efficient catalyst for the isomerization of *N*-allyl substrates. The isomerization of *N*-allylamines, *N*-allylamides, and *N*-allylimines exhibits excellent *E*-stereoselectivity, occurs under moderate conditions, and is compatible with a wide variety of functional groups that include electron-donating, electron-withdrawing, and heteroaromatics substituents. Furthermore, the **Co(I)–Me**-catalyzed isomerization protocol could be extended to a sequential one-pot inverse-electron-demand Diels–Alder reaction to give access to diversely substituted 2-phenylpyridines. To the best of our knowledge, the methodology reported herein represents the first example of a single catalyst that is able to tackle the isomerization of any kind of *N*-allyllic substrate under mild reaction conditions. Current efforts are directed to develop Z-selective protocols and to enable the isomerization of di- and trisubstituted alkenes, which is currently problematic.

## Data Availability

The data underlying this study are available in the published article and its Supporting Information.

## Supporting Information Available

Synthetic procedures, characterization data, and catalysis (PDF)
