# Supplementary material for: A Neutral PCNHCP Co(I)–Me Pincer Complex as a Catalyst for N-Allylic Isomerization with a Broad Substrate Scope
Source: J Org Chem. 2024 Mar 23;89(7):4319–25. doi: 10.1021/acs.joc.3c02349 (PMC11002938; doi:10.1021/acs.joc.3c02349)
Supplement: Supplementary file 1 — jo3c02349_si_001.pdf [file jo3c02349_si_001.pdf]

Supporting Information

A Neutral $PC_{NHC}P$ Co(I)-Me Pincer Complex as a Catalyst for N-Allylic Isomerization with a Broad Substrate Scope.

Sakthi Raje., Tofayel Sheikh Mohammad., and Graham de Ruiter*

[†]*Schulich Faculty of Chemistry, Technion – Israel Institute of Technology; Technion City, Haifa 3200008, Israel.*

E-mail: graham@technion.ac.il

Table of Contents.

General Information **6**
Synthesis of N-substituted allylamines 6
Synthesis of N-substituted allylamides 7
Synthesis of N-substituted allylaldimines 8
Synthesis of N-substituted allylketimines 9
Optimization for N-allylic isomerization **11**
Substrate scope for selective isomerization of allyl amines, amides and imines. **12**
General procedure for selective isomerization of N-allylamines (A). 12
Procedure for multiple bond isomerization of N-allylamine 15
General procedure for selective isomerization of N-allylamides (B) 16
General procedure for selective isomerization of N-allylimines (C) 20
General procedure for selective isomerization of N-allyl substrate at gram scale (D). 28
General method for the synthesis of 2-phenylpyridine derivatives (E) 28
General method for the synthesis of 2-phenylpyridine derivatives (F) 29
NMR and HRMS of synthesized starting materials. **31**
Figure S1. ^{1}H NMR spectrum (400 MHz) of 1g in $CDCl_3$. 31
Figure S2.^{13}C {^{1}H} NMR spectrum (101 MHz) of 1g in $CDCl_3$. 31
Figure S3. High resolution mass spectrum of 1g. 32
Figure S4. ^{1}H NMR spectrum (400 MHz) of 1i in $CDCl_3$. 33
Figure S5. ^{13}C {^{1}H} NMR spectrum (101 MHz) of 1i in $CDCl_3$. 33
Figure S6. High resolution mass spectrum of 1i. 34
Figure S7. ^{1}H NMR spectrum (400 MHz) of 1k in $CDCl_3$. 35
Figure S8. ^{13}C {^{1}H} NMR spectrum (101 MHz) of 1k in $CDCl_3$. 35
Figure S9. High resolution mass spectrum of 1k. 36
Figure S10. ^{1}H NMR spectrum (400 MHz) of 2j in $CDCl_3$. 37
Figure S11. ^{13}C {^{1}H} NMR spectrum (101 MHz) of 2j in $CDCl_3$. 37
Figure S12. High resolution mass spectrum of 2j. 38
Figure S13. ^{1}H NMR spectrum (400 MHz) of 3g in $CDCl_3$. 39
Figure S14. ^{13}C {^{1}H} NMR spectrum (101 MHz) of 3g in $CDCl_3$. 39
Figure S15. High resolution mass spectrum of 3g. 40
Figure S16. ^{1}H NMR spectrum (400 MHz) of 3h in $CDCl_3$. 41
Figure S17. ^{13}C {^{1}H} NMR spectrum (101 MHz) of 3h in $CDCl_3$. 41
Figure S18. High resolution mass spectrum of 3h. 42
Figure S19. ^{1}H NMR spectrum (400 MHz) of 3i in $CDCl_3$. 43
Figure S20. ^{13}C {^{1}H} NMR spectrum (101 MHz) of 3i in $CDCl_3$. 43
Figure S21. High resolution mass spectrum of 3i. 44
Figure S22. ^{1}H NMR spectrum (400 MHz) of 4h in $CDCl_3$. 45
Figure S23. ^{13}C {^{1}H} NMR spectrum (101 MHz) of 4h in $CDCl_3$. 45
Figure S24. High resolution mass spectrum of 4h. 46
Figure S25. ^{1}H NMR spectrum (400 MHz) of 4j in $CDCl_3$. 47
Figure S26. ^{13}C {^{1}H} NMR spectrum (101 MHz) of 4j in $CDCl_3$. 47

Figure S27. High resolution mass spectrum of 4j. 48
NMR of Isomerized N-substituted allylamines 49
Figure S28. ^{1}H NMR spectrum (400 MHz) of 5a in toluene-d_8. 49
Figure S29. ^{13}C {^{1}H} NMR spectrum (101 MHz) of 5a in toluene-d_8. 49
Figure S30. ^{1}H NMR spectrum (400 MHz) of 5b in toluene-d_8. 50
Figure S31. ^{13}C {^{1}H} NMR spectrum (101 MHz) of 5b in toluene-d_8. 50
Figure S32. ^{1}H NMR spectrum (400 MHz) of 5c in toluene-d_8. 51
Figure S33. ^{13}C {^{1}H} NMR spectrum (101 MHz) of 5c in toluene-d_8. 51
Figure S34. ^{1}H NMR spectrum (400 MHz) of 5d in toluene-d_8. 52
Figure S35. ^{13}C {^{1}H} NMR spectrum (101 MHz) of 5d in toluene-d_8. 52
Figure S36. ^{1}H NMR spectrum (400 MHz) of 5e in toluene-d_8. 53
Figure S37. ^{13}C {^{1}H} NMR spectrum (101 MHz) of 5e in toluene-d_8. 53
Figure S38. ^{1}H NMR spectrum (400 MHz) of 5f in toluene-d_8. 54
Figure S39. ^{13}C {^{1}H} NMR spectrum (101 MHz) of 5f in toluene-d_8. 54
Figure S40. ^{1}H NMR spectrum (400 MHz) of 5g in toluene-d_8. 55
Figure S41. ^{13}C {^{1}H} NMR spectrum (101 MHz) of 5g in toluene-d_8. 55
Figure S42. ^{1}H NMR spectrum (400 MHz) of 5h in toluene-d_8. 56
Figure S43. ^{13}C {^{1}H} NMR spectrum (101 MHz) of 5h in toluene-d_8. 56
Figure S44. ^{1}H NMR spectrum (400 MHz) of 5i in toluene-d_8. 57
Figure S45. ^{13}C {^{1}H} NMR spectrum (101 MHz) of 5i in toluene-d_8. 57
Figure S46. ^{1}H NMR spectrum (400 MHz) of 5j in toluene-d_8. 58
Figure S47. ^{13}C {^{1}H} NMR spectrum (101 MHz) of 5j in toluene-d_8. 58
Figure S48. ^{1}H NMR spectrum (400 MHz) of 5k in toluene-d_8. 59
Figure S49. ^{13}C {^{1}H} NMR spectrum (101 MHz) of 5k in toluene-d_8. 59
Figure S50. ^{1}H NMR spectrum (400 MHz) of 5l in toluene-d_8. 60
Figure S51. ^{13}C {^{1}H} NMR spectrum (101 MHz) of 5l in toluene-d_8. 60
Figure S52. ^{1}H NMR spectrum (400 MHz) of 5m in benzene-d_6. 61
Figure S53. ^{13}C {^{1}H} NMR spectrum (101 MHz) of 5m in benzene-d_6. 61
Figure S54. ^{1}H NMR spectrum (400 MHz) of 5n in benzene-d_6. 62
Figure S55. ^{13}C {^{1}H} NMR spectrum (101 MHz) of 5n in benzene-d_6. 62
NMR and HRMS of Isomerized N-substituted allylamides 63
Figure S56. ^{1}H NMR spectrum (400 MHz) of 6a in $CDCl_3$. 63
Figure S57. ^{13}C {^{1}H} NMR spectrum (101 MHz) of 6a in $CDCl_3$. 63
Figure S58. ^{1}H NMR spectrum (400 MHz) of 6b in $CDCl_3$. 64
Figure S59. ^{13}C {^{1}H} NMR spectrum (101 MHz) of 6b in $CDCl_3$. 64
Figure S60. ^{1}H NMR spectrum (400 MHz) of 6c in $CDCl_3$. 65
Figure S61. ^{13}C {^{1}H} NMR spectrum (101 MHz) of 6c in $CDCl_3$. 65
Figure S62. ^{1}H NMR spectrum (400 MHz) of 6d in $CDCl_3$. 66
Figure S63. ^{13}C {^{1}H} NMR spectrum (101 MHz) of 6d in $CDCl_3$. 66
Figure S64. ^{1}H NMR spectrum (400 MHz) of 6e in $CDCl_3$. 67
Figure S65. ^{13}C {^{1}H} NMR spectrum (101 MHz) of 6e in $CDCl_3$. 67
Figure S66. ^{1}H NMR spectrum (400 MHz) of 6f in $CDCl_3$. 68

Figure S67. $^{13}C\{^1H\}$ NMR spectrum (101 MHz) of 6f in $CDCl_3$. 68
Figure S68. ^1H NMR spectrum (400 MHz) of 6g in $CDCl_3$. 69
Figure S69. $^{13}C\{^1H\}$ NMR spectrum (101 MHz) of 6g in $CDCl_3$. 69
Figure S70. ^1H NMR spectrum (400 MHz) of 6h in $CDCl_3$. 70
Figure S71. $^{13}C\{^1H\}$ NMR spectrum (101 MHz) of 6h in $CDCl_3$. 70
Figure S72. ^1H NMR spectrum (400 MHz) of 6i in $CDCl_3$. 71
Figure S73. $^{13}C\{^1H\}$ NMR spectrum (101 MHz) of 6i in $CDCl_3$. 71
Figure S74. ^1H NMR spectrum (400 MHz) of 6j in $CDCl_3$. 72
Figure S75. $^{13}C\{^1H\}$ NMR spectrum (101 MHz) of 6j in $CDCl_3$. 72
Figure S76. High resolution mass spectrum of 6j. 73
Figure S77. ^1H NMR spectrum (400 MHz) of 6k in $CDCl_3$. 74
Figure S78. $^{13}C\{^1H\}$ NMR spectrum (101 MHz) of 6k in $CDCl_3$. 74
Figure S79. High resolution mass spectrum of 6k. 75
Figure S80. ^1H NMR spectrum (400 MHz) of 6l in $CDCl_3$. 76
Figure S81. $^{13}C\{^1H\}$ NMR spectrum (101 MHz) of 6l in $CDCl_3$. 76
NMR and HRMS of Isomerized N-substituted allylimines....77
Figure S82. ^1H NMR spectrum (400 MHz) of 7a in $CDCl_3$. 77
Figure S83. $^{13}C\{^1H\}$ NMR spectrum (101 MHz) of 7a in $CDCl_3$. 77
Figure S84. ^1H NMR spectrum (400 MHz) of 7b in $CDCl_3$. 78
Figure S85. $^{13}C\{^1H\}$ NMR spectrum (101 MHz) of 7b in $CDCl_3$. 78
Figure S86. High resolution mass spectrum of 7b. 79
Figure S87. ^1H NMR spectrum (400 MHz) of 7c in $CDCl_3$. 80
Figure S88. $^{13}C\{^1H\}$ NMR spectrum (101 MHz) of 7c in $CDCl_3$. 80
Figure S89. ^1H NMR spectrum (400 MHz) of 7d in $CDCl_3$. 81
Figure S90. $^{13}C\{^1H\}$ NMR spectrum (101 MHz) of 7d in $CDCl_3$. 81
Figure S91. ^1H NMR spectrum (400 MHz) of 7e in $CDCl_3$. 82
Figure S92. $^{13}C\{^1H\}$ NMR spectrum (101 MHz) of 7e in $CDCl_3$. 82
Figure S93. ^1H NMR spectrum (400 MHz) of 7f in $CDCl_3$. 83
Figure S94. $^{13}C\{^1H\}$ NMR spectrum (101 MHz) of 7f in $CDCl_3$. 83
Figure S95. High resolution mass spectrum of 7f. 84
Figure S96. ^1H NMR spectrum (400 MHz) of 7g in $CDCl_3$. 85
Figure S97. $^{13}C\{^1H\}$ NMR spectrum (101 MHz) of 7g in $CDCl_3$. 85
Figure S98. High resolution mass spectrum of 7g. 86
Figure S99. ^1H NMR spectrum (400 MHz) of 7h in $CDCl_3$. 87
Figure S100. $^{13}C\{^1H\}$ NMR spectrum (101 MHz) of 7h in $CDCl_3$. 87
Figure S101. ^1H NMR spectrum (400 MHz) of 7i in $CDCl_3$. 88
Figure S102. $^{13}C\{^1H\}$ NMR spectrum (101 MHz) of 7i in $CDCl_3$. 88
Figure S103. ^1H NMR spectrum (400 MHz) of 7j in $CDCl_3$. 89
Figure S104. $^{13}C\{^1H\}$ NMR spectrum (101 MHz) of 7j in $CDCl_3$. 89
Figure S105. High resolution mass spectrum of 7j. 90
Figure S106. ^1H NMR spectrum (400 MHz) of 7k in $CDCl_3$. 91
Figure S107. $^{13}C\{^1H\}$ NMR spectrum (101 MHz) of 7k in $CDCl_3$. 91

Figure S108. ^{1}H NMR spectrum (400 MHz) of 7l in $CDCl_3$. 92
Figure S109. ^{13}C {^{1}H} NMR spectrum (101 MHz) of 7l in $CDCl_3$. 92
Figure S110. ^{1}H NMR spectrum (400 MHz) of 8a in $CDCl_3$....................93
Figure S111. ^{13}C {^{1}H} NMR spectrum (101 MHz) of 8a in $CDCl_3$....................93
Figure S112. ^{1}H NMR spectrum (400 MHz) of 8b in $CDCl_3$. 94
Figure S113. ^{13}C {^{1}H} NMR spectrum (101 MHz) of 8b in $CDCl_3$. 94
Figure S114. ^{1}H NMR spectrum (400 MHz) of 8c in $CDCl_3$. 95
Figure S115. ^{13}C {^{1}H} NMR spectrum (101 MHz) of 8c in $CDCl_3$....................95
Figure S116. ^{1}H NMR spectrum (400 MHz) of 8d in $CDCl_3$. 96
Figure S117. ^{13}C {^{1}H} NMR spectrum (101 MHz) of 8d in $CDCl_3$. 96
Figure S118. High resolution mass spectrum of 8d. 97
Figure S119. ^{1}H NMR spectrum (400 MHz) of 8e in $CDCl_3$. 98
Figure S120. ^{13}C {^{1}H} NMR spectrum (101 MHz) of 8e in $CDCl_3$....................98
Figure S121. ^{1}H NMR spectrum (400 MHz) of 8f in $CDCl_3$. 99
Figure S122. ^{13}C {^{1}H} NMR spectrum (101 MHz) of 8f in $CDCl_3$. 99
Figure S123. High resolution mass spectrum of 8f. 100
Figure S124. ^{1}H NMR spectrum (400 MHz) of 8g in $CDCl_3$. 101
Figure S125. ^{13}C {^{1}H} NMR spectrum (101 MHz) of 8g in $CDCl_3$....................101
Figure S126. ^{1}H NMR spectrum (400 MHz) of 8h in $CDCl_3$. 102
Figure S127. ^{13}C {^{1}H} NMR spectrum (101 MHz) of 8h in $CDCl_3$. 102
Figure S128. ^{1}H NMR spectrum (400 MHz) of 8i in $CDCl_3$....................103
Figure S129. ^{13}C {^{1}H} NMR spectrum (101 MHz) of 8i in $CDCl_3$....................103
Figure S130. ^{1}H NMR spectrum (400 MHz) of 8j in $CDCl_3$. 104
Figure S131. ^{13}C {^{1}H} NMR spectrum (101 MHz) of 8j in $CDCl_3$....................104
Figure S132. ^{1}H NMR spectrum (400 MHz) of 8k in $CDCl_3$. 105
Figure S133. ^{13}C {^{1}H} NMR spectrum (101 MHz) of 8k in $CDCl_3$....................105
Figure S134. High resolution mass spectrum of 8k. 106
Figure S135. ^{1}H NMR spectrum (400 MHz) of 8l in $CDCl_3$. 107
Figure S136. ^{13}C {^{1}H} NMR spectrum (101 MHz) of 8l in $CDCl_3$. 107
Figure S137. ^{1}H NMR spectrum (400 MHz) after gram scale isomerization (4a) in $CDCl_3$. 108
Figure S138. ^{1}H NMR spectrum (400 MHz) after gram scale isomerization (8l) in $CDCl_3$....................108
NMR of 2-phenylpyridine derivatives 109
Figure S139. ^{1}H NMR spectrum (400 MHz) of 9a in $CDCl_3$....................109
Figure S140. ^{13}C {^{1}H} NMR spectrum (101 MHz) of 9a in $CDCl_3$....................109
Figure S141. High resolution mass spectrum of 9a....................110
Figure S142. ^{1}H NMR spectrum (400 MHz) of 9b in $CDCl_3$. 111
Figure S143. ^{13}C {^{1}H} NMR spectrum (101 MHz) of 9b in $CDCl_3$. 111
Figure S144. ^{1}H NMR spectrum (400 MHz) of 9c in $CDCl_3$....................112
Figure S145. ^{13}C {^{1}H} NMR spectrum (101 MHz) of 9c in $CDCl_3$....................112
Figure S146. High resolution mass spectrum of 9c....................113
Figure S147. ^{1}H NMR spectrum (400 MHz) of 10a in $CDCl_3$....................114
Figure S148. ^{13}C {^{1}H} NMR spectrum (101 MHz) of 10a in $CDCl_3$....................114

Figure S149. High resolution mass spectrum of 10a......115
Figure S150. ^{1}H NMR spectrum (400 MHz) of 10b in $CDCl_3$. 116
Figure S151. ^{13}C {^{1}H} NMR spectrum (101 MHz) of 10b in $CDCl_3$. 116
Figure S152. ^{1}H NMR spectrum (400 MHz) of 10c in $CDCl_3$......117
Figure S153. ^{13}C {^{1}H} NMR spectrum (101 MHz) of 10c in $CDCl_3$......117
One-pot isomerization. 118
One-pot isomerization of N-allylamine and N-allylaldimine with Co-Me as catalyst......118
One-pot sequential isomerization of N-allylamine and N-allylaldimine with Co-Me as catalyst. .119
References......120

General Information

All reactions were performed at room temperature either by using standard Schlenk techniques or by using an N_2-filled MBraun or Vigor Glovebox unless otherwise specified. Glassware was oven dried at 140 °C for at least 2h prior to use, and allowed to cool under vacuum. All reagents were used as received unless mentioned otherwise. [$(PC_{NHC}P)Co(CH_3)$] (**Co-Me**)[1] and ethyl 3-(pyrrolidin-1-yl)acrylate (**11**)[2] was synthesized by following reported procedure. The ^{1}H and ^{13}C {^{1}H} spectra were recorded on Bruker AVANCE III 400 NMR spectrometer at room temperature. All chemical shifts (δ) are reported in ppm, and coupling constants (*J*) are in Hz. The ^{1}H and ^{13}C {^{1}H} NMR spectra were referenced using residual solvent peaks in the deuterated solvent. Deuterated solvents ($CDCl_3$, benzene-d_6 and toluene-d_8) were purchased from Cambridge Isotope Laboratories, dried over calcium hydride, degassed by three freeze-pump-thaw cycles and vacuum-transferred prior to use. High-resolution mass spectrometry (HRMS) were measured on Bruker Maxis impact system using an atmospheric-pressure chemical ionization (APCI, positive ion) solid probe.

Synthesis of N-substituted allylamines

Compounds **1b**, **1c** and **1d** were purchased from Sigma Aldrich. Compounds **1a**, **1e, 1f, 1h, 1j** and **1l**, **1m**, **1o** were synthesized according to known literature procedures,[3-8] while compounds **1g**, **1i** and **1k** were synthesized according to standard procedure outlined below.

Standard procedure for N-allylamine synthesis. A round bottom flask was charged with the relevant secondary amine (5 mmol, 1 equiv.), K_2CO_3 (15 mmol, 3 equiv.) and allyl bromide (7.5 mmol, 1.5 equiv.) in MeCN (15 mL). The reaction mixture was stirred at room temperature (compounds **1g** and **1i**) or 70 ˚C (compounds **1k**) under an inert atmosphere. The reaction was monitored by TLC and after completion of the reaction, it was filtered through a pad of ®Celite, which was washed with additional Et_2O. The filtrate was concentrated and purified by flash column chromatography (neutral alumina; hexane/diethyl ether).

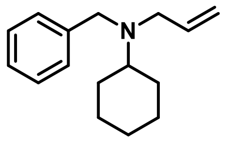

Compound 1g. Prepared according to standard procedure. The title compound was purified by flash column chromatography (neutral alumina; hexane/diethyl ether 60:40) as colorless oil. Yield: 0.98 g (86%). ^{1}H NMR (400 MHz, $CDCl_3$) δ (ppm) 7.36 (d, *J* = 7.5 Hz, 2H), 7.30 (t, *J* = 7.4 Hz, 2H), 7.22 (t, *J* = 7.2 Hz, 1H), 5.83 (dq, *J* = 10.2, 6.2 Hz, 1H), 5.16 (d, *J* = 17.2 Hz, 1H), 5.05 (d, *J* = 10.1 Hz, 1H), 3.63 (s, 2H), 3.13 (d, *J* = 6.2 Hz, 2H), 2.54 (tt, *J* = 11.3, 3.2 Hz, 1H), 1.81 (dd, *J* = 25.9, 12.2 Hz, 4H), 1.62 (d, *J* = 12.4 Hz, 1H), 1.33 – 1.02 (m, 5H). ^{13}C {^{1}H} NMR (101 MHz, $CDCl_3$) δ (ppm) 141.6, 138.0, 128.5, 128.2, 126.5, 116.0, 58.8, 53.7, 53.2, 29.1, 26.6, 26.2. HRMS (APCI, positive ion, m/z): calcd. for ($[C_{16}H_{23}N+H]^+$): 230.1909; found 230.1953.

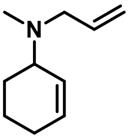

Compound 1i. Prepared according to standard procedure. The title compound was purified by flash column chromatography (neutral alumina; hexane/diethyl ether 50:50) as colorless oil. Yield: 0.61 g (81%). ^{1}H NMR (400 MHz, $CDCl_3$) δ (ppm) 5.91 – 5.76 (m, 2H), 5.62 (d, *J* = 10.1 Hz, 1H), 5.16 (d, *J* = 15.4 Hz, 1H), 5.08 (d, *J* = 10.1 Hz, 1H), 3.34 – 3.27 (m, 1H), 3.15 – 2.98 (m, 2H), 2.21 (s, 3H), 1.99 – 1.92 (m, 2H), 1.82 – 1.71 (m, 2H), 1.58 – 1.39 (m, 2H). ^{13}C {^{1}H} NMR (101 MHz, $CDCl_3$) δ (ppm) 137.2, 130.1, 130.0, 116.8, 59.1, 56.8, 37.5, 25.4, 22.7, 21.8. HRMS (APCI, positive ion, m/z): calcd. for ($[C_{10}H_{17}N+H]^+$): 152.1439; found 152.1452.

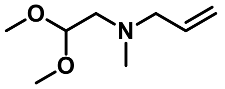

Compound 1k. Prepared according to standard procedure. The title compound was purified by flash column chromatography (neutral alumina; hexane/diethyl ether 20:80) as colorless oil. Yield: 0.56 g (76%). ^{1}H NMR (400 MHz, $CDCl_3$) δ (ppm) 5.86 (ddt, *J* = 16.8, 10.2, 6.6 Hz, 1H), 5.18 – 5.10 (m, 2H), 4.49 (t, *J* = 5.4 Hz, 1H), 3.34 (s, 6H), 3.04 (d, *J* = 6.6 Hz, 2H), 2.50 (d, *J* = 5.4 Hz, 2H), 2.28 (s, 3H). ^{13}C {^{1}H} NMR (101 MHz, $CDCl_3$) δ (ppm) 135.6, 117.9, 102.8, 61.8, 58.1, 53.3, 43.2. HRMS (APCI, positive ion, m/z): calcd. for ($[C_8H_{17}NO_2+H]^+$): 160.1338; found 160.1336.

Synthesis of N-substituted allylamides

The substrates **2a**–**2i**, **2k** and **2l** were synthesized based on the reported literature,[9-11] while compound **2j** was synthesized according to standard procedure outlined below.

Standard procedure for N-allylamide synthesis. A round bottom flask was charged with the relevant secondary amine (5 mmol, 1 equiv.), triethylamine (7.5 mmol, 1.5 equiv.) in dichloromethane (10 mL) under N_2 atmosphere. It was cooled to 0 ˚C then acyl chloride (5 mmol, 1 equiv.) dissolved in

dichloromethane (10 mL) was added drop-wise. The reaction mixture was stirred at room temperature for 14h under an inert atmosphere. After completion of the reaction, it was quenched with water (20 mL) and the aqueous phase was extracted with Et_2O (3 x 25 mL). The combined organic phases were dried over Na_2SO_4 and the solvent was removed under reduced pressure. The crude compound was purified by column chromatography (SiO_2; hexane/ethyl acetate).

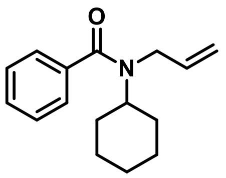

Compound 2j. Prepared according to standard procedure. The title compound was purified by column chromatography (SiO_2; hexane/ethyl acetate 70:30) as colorless oil. Yield: 1.12 g (92%). ^{1}H NMR (400 MHz, $CDCl_3$): (mixture of rotamers) δ (ppm) 7.41 – 7.31 (m, 5H), 5.95 (bs, 0.67H), 5.72 (bs, 0.33H), 5.31 – 4.9 (m, 2H), 4.34 (bs, 0.33H), 4.04 (bs, 1.33H), 3.80 (bs, 0.67H), 3.51 (bs, 0.67H), 1.92 – 1.38 (m, 8H), 1.16 – 9.93 (m, 2H). ^{13}C {^{1}H} NMR (101 MHz, $CDCl_3$): (mixture of rotamers) δ (ppm) 171.5, 137.4, 136.1, 135.3, 129.1, 128.4, 126.1, 116.5, 115.8, 58.9, 54.8, 48.2, 43.9, 31.9, 30.8, 25.7, 25.2. HRMS (APCI, positive ion, m/z): calcd. for ($[C_{16}H_{21}NO+H]^+$): 244.1701; found 244.1700.

Synthesis of N-substituted allylaldimines

The substrates **3a–3f, 3j, 3k** and **3l** were synthesized based on reported literature,[12-16] while compounds **3g, 3h** and **3i** were synthesized according to standard procedure outlined below.

Standard procedure for N-allylaldimine synthesis. A round bottom flask was charged with the relevant aldehyde (5 mmol, 1 equiv.), $MgSO_4$ (1 g) and allylamine (6 mmol, 1.2 equiv.) in diethyl ether (10 mL) under N_2 atmosphere. The reaction mixture was stirred at room temperature for 12h then filtered through a sintered glass funnel. Solvent was removed under reduced pressure and the crude product was transferred to glovebox and filtered through neutral alumina.

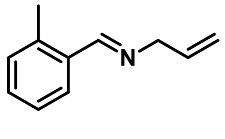

Compound 3g. Prepared according to standard procedure. Obtained as colorless oil. Yield: 0.76 g (95%). ^{1}H NMR (400 MHz, $CDCl_3$) δ (ppm) 8.65 (s, 1H), 7.95 (d, J = 7.7 Hz, 1H), 7.34 (t, J = 7.4 Hz, 1H), 7.28 (t, J = 7.2 Hz, 1H), 7.22 (d, J = 7.4 Hz, 1H), 6.13 (ddd, J = 22.7, 10.8, 5.6 Hz, 1H), 5.28 (d, J = 17.2 Hz, 1H), 5.20 (d, J = 10.3 Hz, 1H), 4.32 (d, J = 6.6 Hz, 2H), 2.55 (s, 3H). ^{13}C {^{1}H} NMR (101 MHz, $CDCl_3$) δ (ppm) 160.6, 137.6, 136.2, 134.2, 130.9, 130.4, 127.5, 126.3, 116.0, 64.1, 19.4. HRMS (APCI, positive ion, m/z): calcd. for ($[C_{11}H_{13}N+H]^+$): 160.1126; found 160.1139.

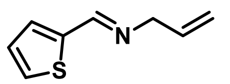

Compound 3h. Prepared according to standard procedure. Obtained as colorless oil. Yield: 0.69 g (91 %). ^{1}H NMR (400 MHz, $CDCl_3$) δ (ppm) 8.37 (s, 1H), 7.39 (d, *J* = 5.0 Hz, 1H), 7.30 (d, *J* = 3.5 Hz, 1H), 7.10 – 7.02 (m, 1H), 6.04 (ddd, *J* = 16.0, 10.8, 5.7 Hz, 1H), 5.18 (dd, *J* = 25.6, 14.4 Hz, 2H), 4.22 (d, *J* = 4.6 Hz, 2H. ^{13}C {^{1}H} NMR (101 MHz, $CDCl_3$) δ (ppm) 155.2, 142.5, 135.8, 130.6, 129.0, 127.4, 116.4, 63.1. HRMS (APCI, positive ion, m/z): calcd. for ($[C_8H_9NS+H]^+$): 152.0534; found 152.0506.

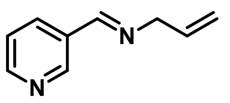

Compound 3i. Prepared according to standard procedure. Obtained as colorless oil. Yield: 0.66 g (90%). ^{1}H NMR (400 MHz, $CDCl_3$) δ (ppm) 8.84 (s, 1H), 8.62 (d, *J* = 4.7 Hz, 1H), 8.30 (s, 1H), 8.10 (d, *J* = 7.9 Hz, 1H), 7.31 (dd, *J* = 7.8, 4.9 Hz, 1H), 6.03 (ddd, *J* = 16.1, 11.2, 5.7 Hz, 1H), 5.18 (dd, *J* = 24.3, 13.1 Hz, 2H), 4.25 (d, *J* = 5.7 Hz, 2H). ^{13}C {^{1}H} NMR (101 MHz, $CDCl_3$) δ (ppm) 159.1, 151.6, 150.3, 135.4, 134.5, 131.7, 123.7, 116.5, 63.7. HRMS (APCI, positive ion, m/z): calcd. for ($[C_9H_{10}N_2+H]^+$): 147.0922; found 147.0883.

Synthesis of N-substituted allylketimines

The substrates **4a–4g, 4i, 4k** and **4l** were synthesized based on reported literature,[13, 17] while compounds **4h** and **4j** were synthesized according to standard procedure outlined below.

Standard procedure for N-allylketimine synthesis. Under inert atmosphere, ketone (3 mmol, 1 equiv.) and allylamine (12 mmol, 4 equiv.) in diethyl ether (20 mL) was added a 1M solution of $TiCl_4$ in toluene (1.5 mmol, 0.5 equiv.) at 0 ˚C in drop-wise manner over a period of 30 minutes. The reaction mixture was warmed to room temperature and stirred for additional 2h. The precipitate was filtered through a pad of ®Celite and washed with ethyl acetate (30 mL). The filtrate was washed with brine (20 mL), dried over Na_2SO_4 and the solvent was removed under reduced pressure. The obtained crude product was transferred to glovebox and filtered through neutral alumina.

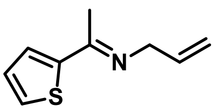

Compound 4h. Prepared according to standard procedure. Obtained as yellow oil. Yield: 0.45 g (92%). ^{1}H NMR (400 MHz, $CDCl_3$) δ (ppm) 7.34 – 7.31 (m, 2H), 7.05 – 7.01 (m, 1H), 6.06 (ddt, *J* = 22.4, 10.4, 5.3 Hz, 1H), 5.23 (d, *J* = 17.2 Hz, 1H), 5.13 (d, *J* = 10.3 Hz, 1H), 4.16 (d, *J* = 5.2 Hz, 2H), 2.24 (s, 3H). ^{13}C {^{1}H} NMR (101 MHz, $CDCl_3$) δ (ppm) 161.1, 147.7, 135.7, 128.7, 127.2, 126.8, 115.3, 54.0, 15.5. HRMS (APCI, positive ion, m/z): calcd. for ($[C_9H_{11}NS+H]^+$): 166.0690; found 166.0722.

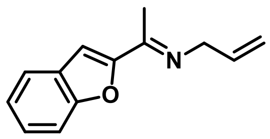

Compound 4j. Prepared according to standard procedure. Obtained as pale yellow solid. Yield: 0.53 g (88%). ^{1}H NMR (400 MHz, $CDCl_3$) δ (ppm) 7.60 (d, *J* = 7.8 Hz, 1H), 7.56 (d, *J* = 8.3 Hz, 1H), 7.33 (t, *J* = 7.8 Hz, 1H), 7.23 (t, *J* = 7.5 Hz, 1H), 7.12 (s, 1H), 6.13 (ddt, *J* = 16.0, 10.8, 5.7 Hz, 1H), 5.23 (d, *J* = 15.7 Hz, 1H), 5.16 (d, *J* = 10.2 Hz, 1H), 4.26 (d, *J* = 5.6 Hz, 2H), 2.26 (s, 3H). ^{13}C {^{1}H} NMR (101 MHz, $CDCl_3$) δ (ppm) 157.9, 155.3, 155.1, 135.2, 128.0, 126.0, 123.1, 121.8, 115.8, 112.1, 107.8, 54.8, 14.9. HRMS (APCI, positive ion, m/z): calcd. for ($[C_{13}H_{13}NO+H]^+$): 200.1075; found 200.1035.

Optimization for N-allylic isomerization

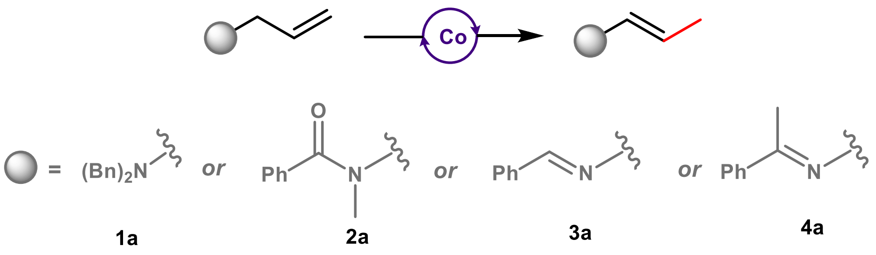

Inside the glovebox, an oven-dried J-Young tube was charged with N-allylic substrate (0.15 mmol) and **Co-Me** (5 mol%), whereafter 400 μL of toluene-d_8 was added. The reaction mixture was stirred at 80 °C for 18h. The progress of the reaction was monitored by ^{1}H NMR spectroscopy. The reaction was optimized by changing to reaction conditions according to **Table S1**.

Table S1. Reaction optimization for N-allylic isomerization.[a]

Entry	Cat. (mol%) / Substrate	Temperature (°C)	Time (h)	Product Conversion[b] (E:Z)
**1**	5 / **1a**	60	16h	99% (31: 1)
**2**	2 / **1a**	60	16h	16% (38:1)
**3**	1 / **1a**	60	24h	8% (100:0)
**4**	2 / **1a**	80	18h	99% (36:1)
**5**	5 / **2a**	60	18h	74% (29:1)
**6**	5 / **2a**	80	12h	98% (28:1)
**7**	3 / **2a**	90	18h	99% (30:1)
**8**	2 / **3a**	60	16h	18% (2.2:1)
**9**	2 / **3a**	80	18h	54% (2.1:1)
**10**	2 / **3a**	90	24h	94% (2.1:1)
**11**	5 / **3a**	80	16h	99% (2.4:1)
**12**	5 / **3a**	85	14h	98% (2.1:1)
**13**	2 / **4a**	80	24h	37% (2.1:1)
**14**	2 / **4a**	90	24h	58% (2.0:1)
**15**	5 / **4a**	80	24h	75% (2.1:1)
**16**	5 / **4a**	85	24h	91% (2.0:1)
**17**	5 / **4a**	90	18h	99% (2.0:1)

[a]Unless otherwise noted, the reaction was performed with N-allylic substrate (0.15 mmol), catalyst (mol%) in 400 μL toluene-d_8 at specified temperature and time. [b] The product conversion and *E*/*Z* ratio were determined by ^{1}H NMR analysis.

Substrate scope for selective isomerization of allyl amines, amides and imines.

General procedure for selective isomerization of N-allylamines (A). Inside the glovebox, an oven-dried J-Young tube was charged with substrate (0.15 mmol) and catalyst **Co-Me** in toluene-d_8 (50 µL to 125 µL, 0.003 mmol (2 mol%) to 0.0075 mmol (5 mol%), from a 0.06M stock solution prepared in toluene-d_8) was added. To the reaction mixture was added an additional amount of toluene-d_8 to make a total volume 400 µL. The J-Young tube was sealed and immersed in a pre-heated oil bath at 80, 90 or 100 ˚C and the progress of the reaction was monitored by ^{1}H NMR spectroscopy. After completion of the reaction, the J-Young tube was transferred to the glovebox and the crude reaction mixture was filtered through short alumina (basic) plug to remove the cobalt catalyst. The alumina was washed with an additional toluene-d_8 (400 µL) to collect all the organic products. For heteroatom containing products, CD_3CN (300 µL) was used. The ^{1}H NMR spectrum was taken after the addition of 1,3,5-trimethoxybenzene (2.9 mg, 0.017 mmol, 0.34M stock solution in toluene-d_8) as an internal standard. The yield and *E*/*Z* ratio of the products were determined by combination of ^{1}H and ^{13}C NMR spectroscopy. *Isolation of the resulting enamines by column chromatography leads to decomposition of the product.*

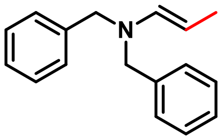

Compound 5a. **Prepared according to general procedure** (**A**). (18h, 85% yield, *E/Z* 36.5:1 (**2 mol%, Co-Me**)) ***E*-5a**: ^{1}H NMR (400 MHz, toluene-d_8): δ (ppm) 7.16 – 7.01 (m, 10H), 6.04 (d, *J* = 13.7 Hz, 1H), 4.24 (dq, *J* = 13.0, 6.4 Hz, 1H), 3.86 (s, 4H), 1.64 (d, *J* = 6.4 Hz, 3H). ^{13}C {^{1}H} NMR (101 MHz, toluene-d_8): 139.3, 139.2, 128.5, 127.9, 127.9, 127.0, 55.0, 15.7. NMR spectra are consistent with previous reported data.[18]

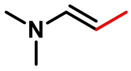

Compound 5b. **Prepared according to general procedure** (**A**). (14h, 87% yield, *E/Z* 100:0 (**2 mol%, Co-Me**)) ***E*-5b** (Internal standard 3.3 mg)**:** ^{1}H NMR (400 MHz, toluene-d_8): δ (ppm) 5.77 (dd, *J* = 13.6, 1.4 Hz, 1H), 4.14 (dq, *J* = 13.0, 6.4 Hz, 1H), 2.29 (s, 6H), 1.68 (d, *J* = 6.4 Hz, 3H). ^{13}C {^{1}H} NMR (101 MHz, toluene-d_8): 141.2, 93.6, 40.8, 15.6. NMR spectra are consistent with previous reported data.[19]

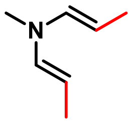

Compound 5c. **Prepared according to general procedure** (**A**). (18h, 90 ˚C, 88% yield, *EE/EZ* 100:0 (**2 mol%, Co-Me**)) ***EE*-5c** (Internal standard 4.4 mg): ^{1}H NMR (400 MHz, toluene-d_8): δ (ppm) 5.89 (d, *J* = 13.6 Hz, 2H), 4.26 (dq, *J* = 13.1, 6.6 Hz, 2H), 2.45 (s, 3H), 1.66 (s, 3H), 1.64 (s, 3H). ^{13}C {^{1}H} NMR (101 MHz, toluene-d_8): 135.8, 94.0, 32.7, 15.4.

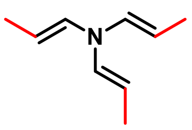

Compound 5d. **Prepared according to general procedure** (**A**). (24h, 100 $^{\circ}$C, 98% yield, *EEE/EEZ/EZZ* 14.7:2.5:1 (**2 mol%, Co-Me**)) ***EEE*-5d**: ^{1}H NMR (400 MHz, toluene-d_8): δ (ppm) 6.03 (d, *J* = 13.6 Hz, 3H), 4.78 (dq, *J* = 13.3, 6.6 Hz, 3H), 1.60 (d, *J* = 6.6 Hz, 9H). ^{13}C {^{1}H} NMR (101 MHz, toluene-d_8): 131.8, 104.3, 15.2. NMR spectra are consistent with previous reported data.[20]

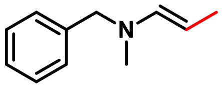

Compound 5e. **Prepared according to general procedure (A)**. (14h, 82% yield, *E/Z* 22.6:1 (**2 mol%, Co-Me**)) ***E*-5e**: ^{1}H NMR (400 MHz, toluene-d_8): δ (ppm) 7.20 – 7.00 (m, 5H), 5.96 (dd, *J* = 13.6, 1.3 Hz, 1H), 4.20 (dq, *J* = 13.0, 6.4 Hz, 1H), 3.72 (s, 2H), 2.29 (s, 3H), 1.71 (dd, *J* = 6.4, 1.2 Hz, 3H). ^{13}C {^{1}H} NMR (101 MHz, toluene-d_8): 140.4, 139.3, 128.5, 128.0, 127.1, 93.0, 59.3, 36.5, 15.6.

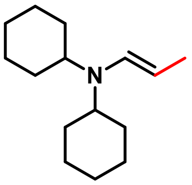

Compound 5f. **Prepared according to general procedure (A)**. (14h, 94% yield, *E/Z* 13.2:1 (**2 mol%, Co-Me**)) ***E*-5f**: ^{1}H NMR (400 MHz, toluene-d_8): δ (ppm) 6.08 (d, *J* = 13.7 Hz, 1H), 4.23 (dq, *J* = 12.7, 6.3 Hz, 1H), 2.98 – 2.89 (m, 2H), 1.84 (d, *J* = 6.3 Hz, 3H), 1.69 – 1.64 (m, 8H), 1.55 – 1.50 (m, 2H), 1.35 – 1.12 (m, 8H), 1.05 – 0.90 (m, 2H). ^{13}C {^{1}H} NMR (101 MHz, toluene-d_8): 133.3, 88.2, 55.4, 32.9, 26.8, 26.2, 16.5.

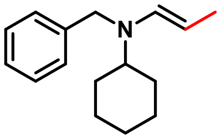

Compound 5g. **Prepared according to general procedure (A)**. (18h, 85% yield, *E/Z* 18.2:1 (**2 mol%, Co-Me**)) ***E*-5g**: ^{1}H NMR (400 MHz, toluene-d_8): δ (ppm) 7.25 (d, *J* = 7.5 Hz, 2H), 7.17 (t, *J* = 7.6 Hz, 2H), 7.06 (t, *J* = 7.3 Hz, 1H), 6.02 (d, *J* = 13.6 Hz, 1H), 4.12 (dt, *J* = 12.9, 6.4 Hz, 1H), 3.90 (s, 2H), 2.71 – 2.64 (m, 1H), 1.72 – 1.66 (m, 5H), 1.62 – 1.58 (m, 2H), 1.48 – 1.44 (m, 1H), 1.19 – 0.99 (m, 4H), 0.94 – 0.81 (m, 1H). ^{13}C {^{1}H} NMR (101 MHz, toluene-d_8): 141.0, 137.0, 128.4, 127.1, 126.6, 92.7, 62.4, 51.1, 31.5, 26.4, 26.1, 16.1.

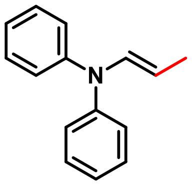

Compound 5h. **Prepared according to general procedure (A)**. (24h, 110 $^{\circ}$C, 86% yield, *E/Z* 3.9:1 (**5 mol%, Co-Me**)) ***E*-5h**: ^{1}H NMR (400 MHz, toluene-d_8): δ (ppm) 7.09 – 7.02 (m, 4H), 6.97 – 6.94 (m, 4H), 6.87 – 6.82 (m, 2H), 6.45 (dd, *J* = 13.7, 1.4 Hz, 1H), 4.71 (dq, *J* = 13.4, 6.7 Hz, 1H), 1.58 (dd, *J* = 6.7, 1.3 Hz, 3H). ^{13}C {^{1}H} NMR (101 MHz, toluene-d_8): 146.5, 134.2, 129.5, 123.5, 122.2, 105.3, 15.2.

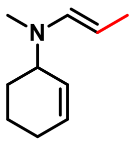

Compound 5i. **Prepared according to general procedure (A)**. (18h, 91% yield, *E/Z* 35.0:1 (**2 mol%, Co-Me**)) ***E*-5i**: ^{1}H NMR (400 MHz, toluene-d_8): δ (ppm) 6.03 (d, *J* = 13.5 Hz, 1H), 5.71 – 5.66 (m, 1H), 5.60 – 5.56 (m, 1H), 4.20 (dq, *J* = 12.9, 6.4 Hz, 1H), 3.45 – 3.35 (m, 1H), 2.37 (s, 3H), 1.80 – 1.73 (m, 5H), 1.59 – 1.52 (m, 2H), 1.43 – 1.31 (m, 2H). ^{13}C {^{1}H} NMR (101 MHz, toluene-d_8): 138.7, 130.5, 130.3, 92.7, 59.9, 33.2, 26.4, 25.1, 21.6, 15.9.

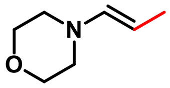

Compound 5j. **Prepared according to general procedure (A)**. (18h, 66% yield, *E/Z* 100:0 (**2 mol%, Co-Me**)) ***E*-5j** (Internal standard 5.8 mg): ^{1}H NMR (400 MHz, toluene-d_8): δ (ppm) 5.63 (dd, *J* = 13.8, 1.4 Hz, 1H), 4.27 (dq, *J* = 13.0, 6.4 Hz, 1H), 3.45 – 3.42 (m, 4H), 2.43 – 2.41 (m, 4H), 1.65 (dd, *J* = 6.5, 1.4 Hz, 3H). ^{13}C {^{1}H} NMR (101 MHz, toluene-d_8): 141.0, 95.8, 66.5, 49.7, 15.5.

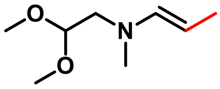

Compound 5k. **Prepared according to general procedure (A)**. (20h, 38% yield, *E/Z* 9.8:1 (**2 mol%, Co-Me**)) ***E*-5k**: ^{1}H NMR (400 MHz, toluene-d_8): δ (ppm) 5.88 (d, *J* = 13.5 Hz, 1H), 4.25 (t, *J* = 5.2 Hz, 1H), 4.12 (dq, *J* = 12.9, 6.3 Hz, 1H), 3.10 (s, 6H), 2.92 (d, *J* = 5.1 Hz, 2H), 2.50 (s, 3H), 1.72 (d, *J* = 6.3 Hz, 3H). ^{13}C {^{1}H} NMR (101 MHz, toluene-d_8): 139.8, 103.5, 91.4, 57.2, 53.2, 37.7, 15.7.

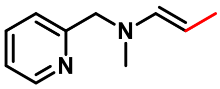

Compound 5l. **Prepared according to general procedure (A)**. (20h, 54% yield, *E/Z* 10.3:1 (**2 mol%, Co-Me**)) ***E*-5l**: ^{1}H NMR (400 MHz, toluene-d_8): δ (ppm) 8.13 (d, *J* = 4.5 Hz, 1H), 7.20 (t, *J* = 8.5 Hz, 1H), 6.83 (d, *J* = 7.8 Hz, 1H), 6.74 – 6.70 (m, 1H), 5.72 (d, *J* = 12.4 Hz, 1H), 3.83 – 3.73 (m, 3H), 2.17 (s, 3H), 1.28 (d, *J* = 6.4 Hz, 3H). ^{13}C {^{1}H} NMR (101 MHz, toluene-d_8): 159.9, 149.7, 140.4, 136.8, 122.5, 122.2, 92.9, 61.1, 37.4, 15.4.

Procedure for multiple bond isomerization of N-allylamine. Inside the glovebox, an oven-dried J-Young tube was charged with substrate (0.15 mmol) and catalyst **Co-Me** in benzene-d_6 (0.0075 mmol (5 mol%), from a 0.06M stock solution prepared in benzene-d_6) was added. To the reaction mixture was added an additional amount of benzene-d_6 to make a total volume 400 μL. The J-Young tube was sealed and immersed in a pre-heated oil bath at 50 or 80˚C and the progress of the reaction was monitored by ^{1}H NMR spectroscopy. After completion of the reaction, the J-Young tube was transferred to the glovebox and the crude reaction mixture was filtered through short alumina (basic) plug to remove the cobalt catalyst. The alumina was washed with an additional benzene-d_6 (400 μL) to collect all the organic products. The ^{1}H NMR spectrum was taken after the addition of 1,3,5-trimethylbenzene (0.34M stock solution in benzene-d_6) as an internal standard. The yield and *E*/*Z* ratio of the products were determined by combination of ^{1}H and ^{13}C NMR spectroscopy. *Isolation of the resulting enamines by column chromatography leads to decomposition of the product.*

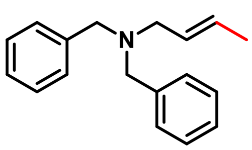

Compound 5m. **One bond isomerization**. (6h, 50˚C, 97% yield, *E/Z* 9.2:1 (**5 mol%, Co-Me**)) ***E*-5m** (Internal standard 3.4 mg): ^{1}H NMR (400 MHz, benzene-d_6): δ (ppm) 7.40 (d, *J* = 7.4 Hz, 4H), 7.20 (t, *J* = 7.5 Hz, 4H), 7.11 (t, *J* = 7.3 Hz 2H), 5.57 – 5.41 (m, 2H), 3.50 (s, 4H), 2.99 (d, *J* = 5.5 Hz, 2H), 1.53 (d, *J* = 5.4 Hz, 3H). ^{13}C {^{1}H} NMR (101 MHz, benzene-d_6): 140.3, 129.0, 128.5, 128.3, 127.8, 127.1, 58.1, 55.8, 17.9. NMR spectra are consistent with previous reported data.[21]

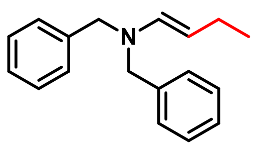

Compound 5n. Two bond isomerization. (18h, 80˚C, 95% yield, *E/Z* 100:0 (**5 mol%, Co-Me**)) ***E*-5n** (Internal standard 2 mg): ^{1}H NMR (400 MHz, benzene-d_6): δ (ppm) 7.16 – 7.13 (m, *J* = 6.0 Hz, 8H), 7.1– 7.0 (m, 2H), 6.11 (d, *J* = 13.7 Hz, 1H), 4.37 (dt, *J* = 13.6, 6.8 Hz, 1H), 2.03 (p, *J* = 7.1 Hz, 2H), 1.00 (t, *J* = 7.4 Hz, 3H). ^{13}C {^{1}H} NMR (101 MHz, benzene-d_6): 139.3, 138.0, 128.6, 128.0, 127.1, 101.4, 55.0, 24.3, 16.5.

General procedure for selective isomerization of N-allylamides (B). Inside the glovebox, an oven-dried J-Young tube was charged with substrate (0.15 mmol) and catalyst **Co-Me** in toluene-d_8 (75 µl to 125 µL, 0.0045 mmol (3 mol%) to 0.0075 mmol (5 mol%), from a 0.06M stock solution prepared in toluene-d_8) was added. To the reaction mixture was added an additional amount of toluene-d_8 to make a total volume of 400 µL. The J-Young tube was sealed and immersed in a pre-heated oil bath at 90 ˚C or 100 ˚C, and the progress of the reaction was monitored by ^{1}H NMR spectroscopy. After completion of the reaction, the mixture was exposed to air and subsequently filtered through a short alumina (neutral) plug to remove the cobalt catalyst. The alumina was washed with an ethyl acetate/hexane (2 mL 50:50) mixture to collect all the organic products. The resulted mixture was evaporated to dryness then the resulted product was re-dissolved in 400 μL of $CDCl_3$ solvent. The yield and *E*/*Z* ratio of the products were determined by combination of ^{1}H and ^{13}C NMR spectroscopy. The enamides were isolated by column chromatography using SiO_2.

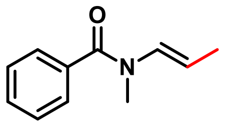

Compound 6a. **Prepared according to general procedure (B)**. Purified by column chromatography (SiO_2; 0% to 20% in EtOAc in hexane, Colorless oil, 16h, 24 mg, 93% yield, *E/Z* 29.6:1 (**3 mol%, Co-Me**)) ***E*-6a**: ^{1}H NMR (400 MHz, $CDCl_3$): (mixture of rotamers) δ (ppm) 7.62 – 7.33 (m, 5.25H), 6.65 – 6.39 (m, 0.75H, major rotamer), 5.17 – 4.94 (m, 1H), 3.24 (s, 2.25H, major rotamer), 3.05 (s, 0.75H, minor rotamer), 1.79 (br, s, 0.75H, minor rotamer), 1.59 (br, s, 2.25H, major rotamer). ^{13}C {^{1}H} NMR (101 MHz, $CDCl_3$): δ (ppm) 170.2, 135.8, 130.9, 130.1, 128.4, 128.0, 105.8, 30.4, 15.4. NMR spectra are consistent with previous reported data.[9]

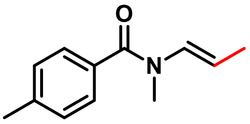

Compound 6b. **Prepared according to general procedure (B)**. Purified by column chromatography (SiO_2; 0% to 30% in EtOAc in hexane, Colorless oil, 16h, 27 mg, 96% yield, *E/Z* 14.9:1 (**3 mol%, Co-Me**)) ***E*-6b** ^{1}H NMR (400 MHz, $CDCl_3$): δ (ppm) 7.34 (d, *J* = 8.0 Hz, 2H), 7.22 – 7.20 (m, 2H), 6.62 – 6.46 (m, 1H), 5.03 – 5.01 (m, 1H), 3.21 (s, 3H), 2.39 (s, 3H), 1.62 (s, 3H). ^{13}C {^{1}H} NMR (101 MHz, $CDCl_3$): δ (ppm) 170.3, 140.3, 132.8, 131.2, 129.0, 128.1, 105.4, 30.5, 21.5, 15.4. NMR spectra are consistent with previous reported data.[9]

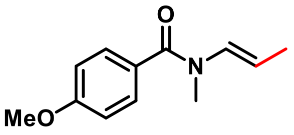

Compound 6c. **Prepared according to general procedure (B)**. Purified by column chromatography (SiO_2; 0% to 30% in EtOAc in hexane, Colorless oil, 16h, 29 mg, 94% yield, *E/Z* 14.5:1 (**3 mol%, Co-Me**)) ***E*-6c**. ^{1}H NMR (400 MHz, $CDCl_3$): δ (ppm) 7.44 – 7.40 (m, 2H), 6.92 – 6.88 (m, 2H), 6.74 – 6.27 (m, 1H), 5.08 – 4.97 (m, 1H), 3.82 (s, 3H), 3.17 (s, 3H), 1.64 (s, 3H). ^{13}C {^{1}H} NMR (101 MHz, $CDCl_3$): δ (ppm) 169.9, 161.1, 130.1 (130.14), 130.1 (130.12), 127.8, 113.6,

105.5, 55.4 (55.41), 55.4 (55.39), 15.4. NMR spectra are consistent with previous reported data.[9]

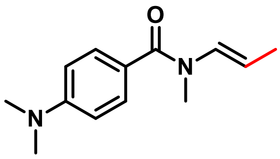

Compound 6d. **Prepared according to general procedure (B)**. Purified by column chromatography (SiO_2; 0% to 40% in EtOAc in hexane, Colorless oil, 16h, 31 mg, 96% yield, *E/Z* 8.9:1 (**3 mol%, Co-Me**)) ***E*-6d**. ^{1}H NMR (400 MHz, $CDCl_3$): δ (ppm) 7.40 (d, *J* = 8.9 Hz, 2H), 6.96 – 6.74 (m, 1H), 6.65 (d, *J* = 8.6 Hz, 2H), 4.97 (dq, *J* = 13.3, 6.6 Hz, 1H), 3.17 (s, 3H), 2.99 (s, 6H), 1.66 (d, *J* = 6.3 Hz, 1H). ^{13}C {^{1}H} NMR (101 MHz, $CDCl_3$): δ (ppm) 170.5, 151.6, 131.3, 130.3, 122.2, 110.9, 104.6, 40.2, 15.4. NMR spectra are consistent with previous reported data.[9]

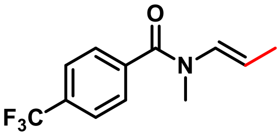

Compound 6e. **Prepared according to general procedure (B).** Purified by column chromatography (SiO_2; 0% to 30% in EtOAc in hexane, Colorless oil, 16h, 33 mg, 90% yield, *E/Z* 11.0:1 (**3 mol%, Co-Me**)) ***E*-6e**. ^{1}H NMR (400 MHz, $CDCl_3$): (mixture of rotamers) δ (ppm) 7.68 (d, *J* = 8.1 Hz, 2H), 7.55 (d, *J* = 8.0 Hz, 2H), 7.47 – 7.37 (minor rotamer, m, 0.3H), 6.38 (major rotamer, d, *J* = 13.7 Hz, 0.7H), 5.19 – 5.07 (m, 1H), 3.26- 3.02 (m, 3H), 1.80 – 1.60(m, 3H). ^{13}C {^{1}H} NMR (101 MHz, $CDCl_3$): δ (ppm) 168.7, 139.3, 131.9 (q, *J* = 33.0 Hz), 130.2, 128.3 – 127.6 (m), 125.6 (d, *J* = 3.2 Hz), 123.8 (d, *J* = 272.4 Hz), 107.3, 30.5, 15.3. NMR spectra are consistent with previous reported data.[9]

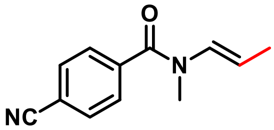

Compound 6f. **Prepared according to general procedure (B)**. Purified by column chromatography (SiO_2; 0% to 40% in EtOAc in hexane, Colorless oil, 18h, 26 mg, 87% yield, *E/Z* 6.2:1 (**3 mol%, Co-Me**)) ***E*-6f**. ^{1}H NMR (400 MHz, $CDCl_3$): (mixture of rotamers) δ (ppm) 7.74 – 7.71 (m, 2H), 7.54 (d, J = 8.2 Hz, 2H), 7.45 – 7.38 (minor rotamer, m, 0.2H), 6.31 (major rotamer, d, *J* = 13.9 Hz, 0.8H), 5.24 – 5.07(m, 1H), 3.25 (major rotamer, s, 2.2H), 3.02 (minor rotamer, s, 0.8H), 1.81 – 1.80 (major rotamer, m, 2.2H), 1.62 – 1.60 (minor rotamer, m, 0.8H). ^{13}C {^{1}H} NMR (101 MHz, $CDCl_3$): δ (ppm) 168.1, 140.1, 132.4, 129.9, 128.6, 118.2, 113.9, 108.0, 30.7, 15.4. NMR spectra are consistent with previous reported data.[9]

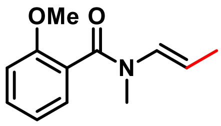

Compound 6g. **Prepared according to general procedure (B)**. Purified by column chromatography (SiO_2; 0% to 40% in EtOAc in hexane, Colorless oil, 18h, 29 mg, 94%, yield, *E/Z* 23.6:1 (**3 mol%, Co-Me**)) ***E*-6g**. ^{1}H NMR (400 MHz, $CDCl_3$): (mixture of rotamers) δ (ppm) 7.49 (minor rotamer, d, *J* = 14.5 Hz, 0.3H), 7.40 – 7.33 (m, 1H), 7.24 (minor rotamer, d, *J* = 7.5 Hz, 0.3H), 7.19 (major

rotamer, d, *J* = 7.4 Hz, 0.7H), 6.99 (t, *J* = 7.4 Hz, 1H), 6.92 (t, *J* = 8.4 Hz, 1H), 6.26 (major rotamer, d, *J* = 15.3 Hz, 0.7H), 5.11 (minor rotamer, dq, *J* = 13.4, 6.6 Hz, 0.3H), 4.98 (major rotamer, dq, *J* = 13.4, 6.6 Hz, 0.7H), 3.81-3.80 (m, 3H), 3.23 (s, 2.1H), 2.91 (s, 0.9H), 1.78 (d, *J* = 6.6 Hz, 0.9H), 1.56 (d, *J* = 6.6 Hz, 2.1H). ^{13}C {^{1}H} NMR (101 MHz, $CDCl_3$): δ (ppm) 168.2, 167.6, 155.6, 155.5, 130.8, 130.3, 128.3, 128.2, 127.6, 126.9, 126.0, 125.5, 121.0, 120.9, 111.3, 111.0, 107.1, 105.3, 55.7, 55.6, 33.7, 29.5, 15.5, 15.4. NMR spectra are consistent with previous reported data.[9]

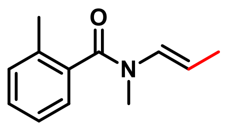

Compound 6h. **Prepared according to general procedure (B).** Purified by column chromatography (SiO_2; 0% to 30% in EtOAc in hexane, Colorless oil, 18h, 27 mg, 95% yield, *E/Z* 16.2:1 (**3 mol%, Co-Me**)) ***E*-6h**. ^{1}H NMR (400 MHz, $CDCl_3$): (mixture of rotamers) δ (ppm) (mixture of rotamers) δ (ppm) 7.51 (minor rotamer, d, *J* = 14.5 Hz, 0.3H), 7.32 – 7.27 (m, 1H), 7.24– 7.16 (m, 3H), 6.22 (major rotamer, dd, *J* = 13.9, 1.5 Hz, 0.7H), 5.14 (minor rotamer, dq, *J* = 13.3, 6.7 Hz, 0.3H), 5.01 (major rotamer, dq, *J* = 13.4, 6.7 Hz, 0.7H), 3.25 (major rotamer, s, 2.1H), 2.90 (minor rotamer, s, 0.9H), 2.27 – 2.24 (m, 3H), 1.80 (minor rotamer, dd, *J* = 6.6, 1.6 Hz, 0.9H), 1.55 (major rotamer, dd, *J* = 6.7, 1.6 Hz, 2.1H). ^{13}C {^{1}H} NMR (101 MHz, $CDCl_3$): δ (ppm) 170.2, 169.6, 136.4, 136.0, 134.5, 134.2, 130.4, 129.8, 129.2, 129.1, 127.3, 126.5, 126.0, 107.5, 105.9, 33.8, 29.3, 19.1, 19.0, 15.5, 15.3. NMR spectra are consistent with previous reported data.[9]

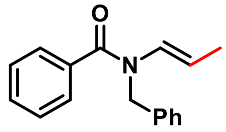

Compound 6i. **Prepared according to general procedure (B).** Purified by column chromatography (SiO_2; 0% to 40% in EtOAc in hexane, White hydroscopic solid, 18h, 36 mg, 96% yield, *E/Z* 6.1:1 (**3 mol%, Co-Me**)) ***E*-6i**. ^{1}H NMR (400 MHz, $CDCl_3$): δ (ppm) 7.49 – 7.22 (m, 10H), 6.71 – 6.32 (m, 1H), 5.04 – 4.96 (m, 3H), 1.54 (s, 3H). ^{13}C {^{1}H} NMR (101 MHz, $CDCl_3$): δ (ppm) 170.3, 137.3, 135.7, 130.3, 129.9, 128.7, 128.5, 128.1, 127.1, 126.8, 107.5, 47.3, 15.5. NMR spectra are consistent with previous reported data.[9]

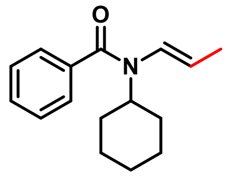

Compound 6j. **Prepared according to general procedure (B).** Purified by column chromatography (SiO_2; 0% to 40% in EtOAc in hexane, White Solid, 18h, 34 mg, 94% yield, *E/Z* 52.8:1 (**3 mol%, Co-Me**)) ***E*-6j**. ^{1}H NMR (400 MHz, $CDCl_3$): (mixture of rotamers) δ (ppm) 7.48 – 7.44 (m, 1H), 7.42 – 7.37 (m, 1H), 7.35 – 7.25 (m, 3H), 5.99 (dd, *J* = 20.7, 11.3 Hz, 1H), 5.35 – 5.09 (m, 1H), 4.55 – 4.25 (m, 1H), 1.90 – 1.76 (m, 4H), 1.70 – 1.05 (m, 6H). ^{13}C {^{1}H} NMR (101 MHz, $CDCl_3$) δ (ppm) 170.6, 170.3, 137.6, 137.5, 129.5, 129.2, 128.2, 128.1, 127.9,

127.5, 127.4 – 127.3 (m), 126.9, 125.6, 55.1, 54.9, 30.5, 30.2, 25.9, 25.7, 25.6, 15.3, 12.7. HRMS (APCI, positive ion, m/z): calcd. for ($[C_{16}H_{21}NO+H]^+$): 244.1701; found 244.1723.

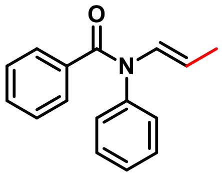

Compound 6k. **Prepared according to general procedure (B).** Purified by column chromatography (SiO_2; 0% to 40% in EtOAc in hexane, Colorless oil, 100 ˚C, 34 mg, 24h, 95% yield, E/Z 6.8:1 (**3 mol%, Co-Me**)) ***E*-6k** ^{1}H NMR (400 MHz, $CDCl_3$): δ (ppm) 7.45 – 7.26 (m, 1H), 7.25 – 7.07 (m, 6H), 7.11 – 7.07 (m, 2H), 7.02 (d, *J* = 7.3 Hz, 2H), 4.66 (dq, *J* = 13.6, 6.8 Hz, 1H), 1.59 (d, *J* = 6.8 Hz, 3H). ^{13}C {^{1}H} NMR (101 MHz, $CDCl_3$): δ (ppm) 168.7, 140.4, 135.8, 130.3, 129.8, 129.3, 129.2, 128.6, 127.8, 127.6, 111.3, 15.3. HRMS (APCI, positive ion, m/z): calcd. for ($[C_{16}H_{15}NO+H]^+$): 238.1232; found 238.1267.

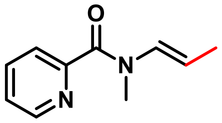

Compound 6l. **Prepared according to general procedure (B).** Purified by column chromatography (SiO_2; 0% to 45% in EtOAc in hexane, Colorless oil, 24h, 24 mg, 90% yield, *E/Z* 20.4:1 (**3 mol%, Co-Me**)) ***E*-6l**. ^{1}H NMR (400 MHz, $CDCl_3$): (mixture of rotamers) δ (ppm) 8.62 – 8.58 (m, 1H), 7.82 – 7.76 (m, 1H), 7.63 – 7.60 (m, 1H), 7.43 (minor rotamer, d, *J* = 14.6 Hz, 0.4H), 7.39 – 7.32 (m, 1H), 6.64 (major rotamer, d, *J* = 13.9 Hz, 0.6H), 5.19 (minor rotamer, dq, *J* = 13.6, 6.9 Hz, 0.4H), 5.07 (major rotamer, dq, *J* = 13.4, 6.6 Hz, 0.6H), 3.26 (major rotamer, s, 2H), 3.11 (minor rotamer, s, 1H), 1.78 (minor rotamer, d, *J* = 6.5 Hz, 1H), 1.59 (major rotamer, d, *J* = 6.6 Hz, 2H). ^{13}C {^{1}H} NMR (101 MHz, $CDCl_3$): δ (ppm) (mixter of rotamer) 167.6, 154.3, 154.0, 148.6, 148.4, 137.2, 137.2, 130.3, 128.1, 124.7, 124.3, 124.1, 108.2, 106.6, 34.6, 30.4, 15.6, 15.4. NMR spectra are consistent with previous reported data.[9]

General procedure for selective isomerization of N-allylimines (C). Inside the glovebox, an oven-dried J-Young tube was charged with substrate (0.15 mmol) and catalyst **Co-Me** in toluene-d_8 (125 μL, 0.0075 mmol (5 mol%) from a 0.06M stock solution prepared in toluene-d_8) was added. To the reaction mixture was added an additional amount of toluene-d_8 to make a total volume of 400 μL. The J-Young tube was sealed and immersed in a pre-heated oil bath at 80 ˚C or 90 ˚C and the progress of the reaction was monitored by ^{1}H NMR spectroscopy. After completion of the reaction, the J-Young tube was transferred to the glovebox and filtered through short alumina (neutral) plug to remove the cobalt catalyst. The alumina was washed with an additional DCM (2 mL) to collect all the organic products. The resulted mixture was evaporated to dryness where after the ^{1}H NMR spectrum was recorded in $CDCl_3$, after the addition of 1,3,5-trimethylbenzene (2 mg, 0.017 mmol, 0.17M stock solution in $CDCl_3$) as an internal standard. The yield and *E*/*Z* ratio of the products were determined by combination of ^{1}H and ^{13}C NMR spectroscopy. The attempts for the isolation of the resulted isomerized products leads to decomposition for majority of the compounds due to its moderate stability.

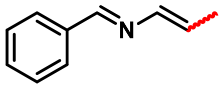

Compound 7a. **Prepared according to general procedure (C)**. (80 ˚C, 16h, 94% yield, *E/Z* 2.2:1.0 (**5 mol%, Co-Me**)) ***E*-7a**: ^{1}H NMR (400 MHz, $CDCl_3$): δ (ppm) 8.17 (s, 1H), 7.79 (m, 2H), 7.43 (m, 3H), 6.87 (d, *J* = 13.0 Hz, 1H), 6.19 (td, *J* = 13.7, 6.9 Hz, 1H), 1.87 (d, *J* = 6.9 Hz, 3H). ^{13}C {^{1}H} NMR (101 MHz, $CDCl_3$): δ (ppm), 159.03, 143.93, 136.40, 130.80, 128.74, 128.45, 127.81, 15.75. ***Z*-7a**: ^{1}H NMR (400 MHz, $CDCl_3$): 8.21 (s, 1H), 7.85 (m, 2H), 7.43 (m, 3H), 6.81 (d, *J* = 9.4 Hz, 1H), 5.58 – 5.48 (m, 1H), 2.09 (d, *J* = 6.9 Hz, 3H). ^{13}C {^{1}H} NMR (101 MHz, $CDCl_3$): δ (ppm) 159.4, 141.6, 136.7, 130.8, 128.7, 128.5, 127.0, 12.7. NMR spectra are consistent with previous reported data.[22 23]

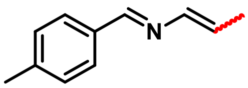

Compound 7b. **Prepared according to general procedure (C)**. (80 ˚C, 16h, 91% yield, *E/Z* 2.1:1.0 (**5 mol%, Co-Me**)) ***E*-7b**: ^{1}H NMR (400 MHz, $CDCl_3$): δ (ppm) 8.14 (s, 1H), 7.68 (d, *J* = 7.7 Hz, 2H), 7.25 (d, *J* = 15.0 Hz, 2H), 6.85 (d, *J* = 13.5 Hz, 1H), 6.15 (td, *J* = 13.7, 6.9 Hz, 1H), 2.40 (s, 3H), 1.86 (d, *J* = 6.9 Hz, 3H).^{13}C {^{1}H} NMR (101 MHz, $CDCl_3$): δ (ppm), 159.08, 144.04, 141.18, 133.78, 129.50, 128.45, 127.07, 21.66, 15.72. ***Z*-7b**: ^{1}H NMR (400 MHz, $CDCl_3$): 8.18 (s, 1H), 7.74 (d, *J* = 7.7 Hz, 2H), 7.25 (d, *J* = 15.0 Hz, 2H), 6.79 (d, *J* = 7.4 Hz, 1H), 5.55 – 5.46 (m, 1H), 2.40 (s, 3H), 2.08 (d, *J* = 6.8 Hz, 3H) ^{13}C {^{1}H} NMR (101 MHz, $CDCl_3$): δ (ppm) 159.5, 141.7, 141.2, 134.1, 129.4, 128.5, 127.0, 21.7, 12.7. HRMS (APCI, positive ion, m/z): calcd. for ($[C_{11}H_{13}N+H]^+$): 160.1126; found 160.1168.

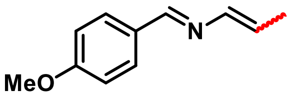

Compound 7c. **Prepared according to general procedure (C)**. (80 ˚C, 16h, 93% yield, *E/Z* 2.5:1.0 (**5 mol%, Co-Me**)) ***E*-7c**: ^{1}H NMR (400 MHz, $CDCl_3$): δ (ppm) 8.10 (s, 1H), 7.73 (d, *J* = 8.5 Hz, 2H), 6.94 (d, *J* = 8.5 Hz, 2H), 6.86 – 6.80 (m, 1H), 6.11 (m, 1H), 3.84 (s, 3H), 1.85 (d, *J* = 6.8 Hz, 3H). ^{13}C {^{1}H} NMR (101 MHz, $CDCl_3$): δ (ppm), 161.85, 158.53, 144.06, 130.06, 129.33, 126.27, 114.19, 55.44, 15.69. ***Z*-7c**: ^{1}H NMR (400 MHz, $CDCl_3$): 8.14 (s, 1H), 7.78 (d, *J* = 8.5 Hz, 2H), 6.94 (d, *J* = 8.5 Hz, 2H), 6.76 (d, *J* = 7.5 Hz, 1H), 5.49 – 5.42 (m, 1H), 3.84 (s, 3H), 2.06 (d, *J* = 6.8 Hz, 3H). ^{13}C {^{1}H} NMR (101 MHz, $CDCl_3$): δ (ppm) 161.9, 158.9, 141.7, 130.1, 129.7, 127.0, 114.1, 55.4, 12.6. NMR spectra are consistent with previous reported data.[23]

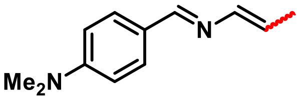

Compound 7d. **Prepared according to general procedure (C)**. (80 ˚C, 14h, 94% yield, *E/Z* 2.3:1.0 (**5 mol%, Co-Me**)) ***E*-7d**: ^{1}H NMR (400 MHz, $CDCl_3$): δ (ppm) 8.05 (s, 1H), 7.66 (d, *J* = 8.9 Hz, 2H), 6.82 – 6.78 (m, 1H), 6.70 (d, *J* = 9.0, 2H), 6.03 (dq, *J* = 13.8, 7.0 Hz, 1H), 3.02 (s, 6H), 1.83 (dd, *J* = 7.0, 1.2 Hz, 3H).^{13}C {^{1}H} NMR (101 MHz, $CDCl_3$): δ (ppm), 159.35, 152.13, 144.48, 129.98, 126.95, 124.15, 111.65, 40.20, 15.63. ***Z*-7d**: ^{1}H NMR (400 MHz, $CDCl_3$): 8.09 (s, 1H), 7.71 (d, *J* = 7.0 Hz, 2H), 6.76 – 6.73 (m, 1H), 6.70 (d, *J* = 9.0, 2H), 5.36 (p, *J* = 7.2 Hz, 1H), 3.03 (s, 6H), 2.06 (dd, *J* = 7.0, 1.6 Hz, 3H). ^{13}C {^{1}H} NMR (101 MHz, $CDCl_3$): δ (ppm) 159.7, 152.2, 142.2, 130.0, 124.8, 124.3, 111.6, 40.2, 12.5. NMR spectra are consistent with previous reported data.[23]

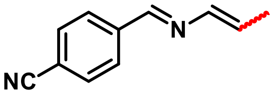

Compound 7e. **Prepared according to general procedure (C)**. (Pale yellow solid, 80 ˚C, 6h, 87% yield, *E/Z* 2.1:1.0 (**5 mol%, Co-Me**)) ***E*-7e**: ^{1}H NMR (400 MHz, $CDCl_3$): δ (ppm) 8.14 (s, 1H), 7.86 (d, *J* = 8.2 Hz, 2H), 7.69 (d, *J* = 8.1 Hz, 2H), 6.88 (d, *J* = 12.8 Hz, 1H), 6.27 (dq, *J* = 14.0, 7.1 Hz, 1H), 1.88 (d, *J* = 7.0 Hz, 3H).^{13}C {^{1}H} NMR (101 MHz, $CDCl_3$): δ (ppm) 156.38, 143.48, 140.36, 132.50, 128.77, 128.67, 118.71, 113.71, 15.90. ***Z*-7e**: ^{1}H NMR (400 MHz, $CDCl_3$): 8.18 (s, 1H), 7.91 (d, *J* = 8.1 Hz, 2H), 7.69 (d, *J* = 8.1 Hz, 2H), 6.83 (d, *J* = 7.6 Hz, 1H), 5.66 (p, *J* = 7.1 Hz, 1H), 2.07 (d, *J* = 6.3 Hz, 3H). ^{13}C {^{1}H} NMR (101 MHz, $CDCl_3$): δ (ppm) 156.9, 141.1, 140.6, 130.9, 128.7, 128.6, 118.7, 113.7, 12.8. NMR spectra are consistent with previous reported data.[23]

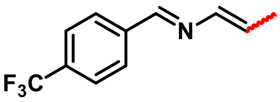

Compound 7f. **Prepared according to general procedure (C)**. (80 ˚C, 16h, 90% yield, *E/Z* 2.5:1.0 (**5 mol%, Co-Me**)) ***E*-7f**: ^{1}H NMR (400 MHz, $CDCl_3$): δ (ppm) 8.18 (s, 1H), 7.88 (d, *J* = 8.1 Hz, 2H), 7.67 (d, *J* = 8.1 Hz, 2H), 6.88 (d, *J* = 12.9 Hz, 1H), 6.25 (dq, *J* = 14.0, 7.1 Hz, 1H), 1.88 (d, *J* = 7.0 Hz, 3H). ^{13}C {^{1}H} NMR

(101 MHz, $CDCl_3$): δ (ppm) 157.09, 143.62, 139.61, 132.77 – 131.56 (m), 129.94, 128.55, 125.83 – 125.60 (m), 125.45, 15.85. ***Z*-7f**: ^{1}H NMR (400 MHz, $CDCl_3$): 8.22 (s, 1H), 7.93 (d, *J* = 8.1 Hz, 2H), 7.67 (d, *J* = 8.1 Hz, 2H), 6.83 (d, *J* = 9.3 Hz, 1H), 5.62 (p, *J* = 7.2 Hz, 1H), 2.09 (d, *J* = 7.0 Hz, 3H). ^{13}C {^{1}H} NMR (101 MHz, $CDCl_3$): δ (ppm) 157.5, 141.2, 139.9, 132.8 – 131.5 (m), 128.6, 127.7, 125.8 – 125.6 (m), 122.7, 12.8. HRMS (APCI, positive ion, m/z): calcd. for ($[C_{11}H_{10}F_3N+H]^+$): 214.0844; found 214.0886.

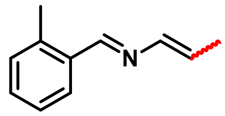

Compound 7g. **Prepared according to general procedure (C)**. (80 °C, 18h, 92% yield, *E/Z* 2.2:1.0 (**5 mol%, Co-Me**)) ***E*-7g**: ^{1}H NMR (400 MHz, $CDCl_3$): δ (ppm) 8.49 (s, 1H), 8.05 – 7.97 (m, 1H), 7.26 (m, 3H), 6.89 (d, *J* = 12.7 Hz, 1H), 6.19 (dq, *J* = 13.9, 7.0 Hz, 1H), 2.53 (s, 3H), 1.88 (d, *J* = 6.9 Hz, 3H).^{13}C {^{1}H} NMR (101 MHz, $CDCl_3$): δ (ppm), 157.39, 144.51, 138.05, 134.29, 130.94, 130.47, 127.63, 127.33, 126.35, 19.38, 15.76. ***Z*-7g**: ^{1}H NMR (400 MHz, $CDCl_3$): 8.50 (s, 1H), 8.05 – 7.97 (m, 1H), 7.26 (m, 3H), 6.85 (d, *J* = 20.4 Hz, 1H), 5.54 (p, *J* = 7.0 Hz, 1H), 2.57 (s, 3H), 2.09 (d, *J* = 6.4 Hz, 3H). ^{13}C {^{1}H} NMR (101 MHz, $CDCl_3$): δ (ppm) 158.2, 142.2, 138.2, 134.5, 131.0, 130.4, 128.2, 126.2, 125.5, 19.8, 12.8. HRMS (APCI, positive ion, m/z): calcd. for ($[C_{11}H_{13}N+H]^+$): 160.1126; found 160.1146.

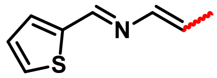

Compound 7h. **Prepared according to general procedure (C)**. (80 °C, 18h, 91% yield, *E/Z* 2.5:1.0 (**5 mol%, Co-Me**)) ***E*-7h**: ^{1}H NMR (400 MHz, $CDCl_3$): δ (ppm) 8.26 (s, 1H), 7.45 – 7.41 (m, 1H), 7.36 – 7.32 (m, 1H), 7.11 – 7.06 (m, 1H), 6.80 (d, *J* = 14.5 Hz, 1H), 6.12 (dq, *J* = 13.8, 7.0 Hz, 1H), 1.84 (d, *J* = 6.9 Hz, 3H). ^{13}C {^{1}H} NMR (101 MHz, $CDCl_3$): δ (ppm) 151.93, 143.41, 141.16, 131.04, 129.50, 127.77, 127.50, 15.75. ***Z*-7h**: ^{1}H NMR (400 MHz, $CDCl_3$): 8.29 (s, 1H), 7.45 – 7.41 (m, 1H), 7.36 – 7.32 (m, 1H), 7.11 – 7.06 (m, 1H), 6.74 (d, *J* = 7.5 Hz, 1H), 5.54 – 5.43 (m, 1H), 2.03 (d, *J* = 6.8 Hz, 3H). ^{13}C {^{1}H} NMR (101 MHz, $CDCl_3$): δ (ppm) 152.3, 143.3, 142.8, 130.9, 129.6, 127.7, 125.4, 12.7. NMR spectra are consistent with previous reported data.[24]

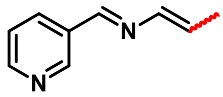

Compound 7i. **Prepared according to general procedure (C)**. (80 °C, 6h, 76% yield, *E/Z* 2.4:1 (**5 mol%, Co-Me**)) ***E*-7i**: ^{1}H NMR (400 MHz, $CDCl_3$): δ (ppm) 8.95 – 8.87 (m, 1H), 8.65 – 8.61 (m, 1H), 8.22 – 8.13 (m, 2H), 7.37 – 7.32 (m, 1H), 6.87 (d, *J* = 12.9 Hz, 1H), 6.23 (td, *J* = 14.0, 7.1 Hz, 1H), 1.87 (d, *J* = 7.0 Hz, 3H). ^{13}C {^{1}H} NMR (101 MHz, $CDCl_3$): δ (ppm) 155.63, 151.46, 150.60, 143.69, 134.49, 132.12, 129.57, 123.83, 15.80. ***Z*-7i**: ^{1}H NMR (400 MHz, $CDCl_3$): 8.95 –

8.87 (m, 1H), 8.65 – 8.61 (m, 1H), 8.22 – 8.13 (m, 2H), 7.37 – 7.32 (m, 1H), 6.81 (d, *J* = 7.1 Hz, 1H), 5.60 (p, *J* = 7.2 Hz, 1H), 2.07 (d, *J* = 7.0 Hz, 3H). ^{13}C {^{1}H} NMR (101 MHz, $CDCl_3$): δ (ppm) 156.1, 151.5, 150.6, 141.3, 134.7, 132.4, 127.4, 123.7, 12.7. NMR spectra are consistent with previous reported data.[25]

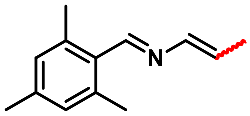

Compound 7j. **Prepared according to general procedure (C)**. (90 °C, 18h, 95% yield, *E/Z* 1.9:1 (**5 mol%, Co-Me**)) ***E*-7j**: ^{1}H NMR (400 MHz, $CDCl_3$): δ (ppm) 8.51 (s, 1H), 6.90 (s, 1H), 6.88 (s, 1H), 6.82 (d, *J* = 3.9 Hz, 1H), 6.12 (dq, *J* = 13.9, 7.0 Hz, 1H), 2.46 (s, 6H), 2.29 (s, 3H), 1.86 (d, *J* = 6.8 Hz, 3H). ^{13}C {^{1}H} NMR (101 MHz, $CDCl_3$): δ (ppm) 159.20, 144.90, 139.19, 138.30, 130.76, 129.70, 126.70, 21.60, 21.12, 15.68. ***Z*-7j**: ^{1}H NMR (400 MHz, $CDCl_3$): 8.58 (s, 1H), 6.88 (s, 1H), 6.84 (s, 1H), 6.76 (d, *J* = 7.4 Hz, 1H), 5.50 (p, *J* = 7.0 Hz, 1H), 2.53 (s, 6H), 2.29 (s, 3H), 2.03 (d, *J* = 6.9 Hz, 3H). ^{13}C {^{1}H} NMR (101 MHz, $CDCl_3$): δ (ppm) 159.3, 142.8, 139.4, 138.8, 130.5, 129.9, 124.7, 21.3, 21.3, 12.8. HRMS (APCI, positive ion, m/z): calcd. for ($[C_{13}H_{17}N+H]^+$): 188.1439; found 188.1452.

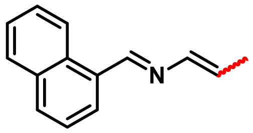

Compound 7k. **Prepared according to general procedure (C)**. (80 °C, 18h, 82% yield, *EE/ZZ* 13.9:1 (**5 mol%, Co-Me**)) ***E*-7k**: ^{1}H NMR (400 MHz, $CDCl_3$): δ (ppm) 8.92 (d, *J* = 8.5 Hz, 1H), 8.85 (s, 1H), 8.02 (d, *J* = 7.1 Hz, 1H), 7.93 – 7.87 (m, 2H), 7.71 – 7.50 (m, 3H), 6.98 (d, *J* = 12.9 Hz, 1H), 6.29 (dq, *J* = 13.8, 7.0 Hz, 1H), 1.92 (d, *J* = 7.0 Hz, 3H). ^{13}C {^{1}H} NMR (101 MHz, $CDCl_3$): δ (ppm) 158.17, 144.69, 134.00, 131.68, 131.54, 131.28, 129.93, 128.82, 128.76, 128.00, 127.22, 126.15, 125.43, 124.20, 15.83. ***Z*-7k**: ^{1}H NMR (400 MHz, $CDCl_3$): 9.16 (d, *J* = 8.6 Hz, 1H), 8.83 (s, 1H), 7.93 – 7.87 (m, 2H), 7.71 – 7.50 (m, 3H), 7.23 (d, *J* = 7.7 Hz, 1H), 6.91 (d, *J* = 7.3 Hz, 1H), 5.67 – 5.56 (m, 1H), 2.17 (d, *J* = 7.3 Hz, 3H). ^{13}C {^{1}H} NMR (101 MHz, $CDCl_3$): δ (ppm) 159.3, 142.5, 134.0, 131.8, 131.5, 131.4, 129.5, 128.5, 128.4, 127.3, 126.2, 125.7, 125.3, 124.8, 13.0. NMR spectra are consistent with previous reported data.[24]

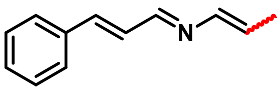

Compound 7l. **Prepared according to general procedure (C)**. (80 °C, 16h, 36% yield, *E/Z* 4.8:1 (**5 mol%, Co-Me**)) ***E*-7l**: ^{1}H NMR (400 MHz, $CDCl_3$): δ (ppm) 7.93 (t, *J* = 4.0 Hz, 1H), 7.52 – 7.48 (m, 2H), 7.39 – 7.26 (m, 3H), 7.06 – 6.95 (m, 2H), 6.77 (d, *J* = 13.0 Hz, 1H), 6.10 (dq, *J* = 13.9, 7.1 Hz, 1H), 1.85 (d, *J* = 6.9 Hz, 3H). ^{13}C {^{1}H} NMR (101 MHz, $CDCl_3$): δ (ppm) 160.52, 144.28, 142.26, 136.04, 129.33, 128.99, 128.51, 127.88, 127.41, 15.86. ***Z*-7l**: ^{1}H NMR (400 MHz, $CDCl_3$): 7.99 (d, *J* = 8.1 Hz, 1H), 7.52 – 7.48 (m, 2H), 7.39 – 7.26 (m, 3H), 7.06 – 6.95 (m, 2H), 6.70 (d, *J* = 7.5 Hz, 1H), 5.48 (p, *J* = 14.5, 1H), 2.01 (d, *J* = 7.0 Hz, 3H). ^{13}C

{^{1}H} NMR (101 MHz, $CDCl_3$): δ (ppm) 161.6, 142.4, 142.3, 137.7, 135.4, 128.7, 128.1, 127.4, 125.4, 12.7. NMR spectra are consistent with previous reported data.[26]

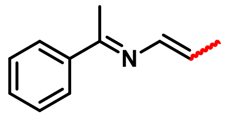

Compound 8a. **Prepared according to general procedure (C)**. (90 ˚C, 16h, 92% yield, *E/Z* 2.0:1.0 (**5 mol%, Co-Me**)) ***E*-8a**: ^{1}H NMR (400 MHz, $CDCl_3$): δ (ppm) 7.90 – 7.85 (m, 2H), 7.42 – 7.35 (m, 3H), 7.13 (d, *J* = 12.9 Hz, 1H), 6.11 (dq, *J* = 13.8, 7.0 Hz, 1H), 2.32 (s, 3H), 1.89 (d, *J* = 7.0 Hz, 3H). ^{13}C {^{1}H} NMR (101 MHz, $CDCl_3$): δ (ppm), 161.54, 140.80 (d, *J* = 9.1 Hz), 137.28 (d, *J* = 18.3 Hz), 129.76, 128.35 (bs), 127.65 (d, *J* = 9.6 Hz), 127.04 – 126.79 (m), 16.17, 15.42. ***Z*-8a**: ^{1}H NMR (400 MHz, $CDCl_3$): 7.97 – 7.95 (m, 2H), 7.42 – 7.35 (m, 3H), 7.01 (d, *J* = 7.8 Hz, 1H), 5.49 (p, *J* = 7.1 Hz, 1H), 2.30 (s, 3H), 1.99 (d, *J* = 6.9 Hz, 3H). ^{13}C {^{1}H} NMR (101 MHz, $CDCl_3$): δ (ppm) 162.0, 137.8, 135.2 (d, *J* = 23.9 Hz), 129.9, 128.4 (bs), 127.2 – 127.1 (m), 123.1 (d, *J* = 22.0 Hz), 15.6, 12.3. NMR spectra are consistent with previous reported data.[24]

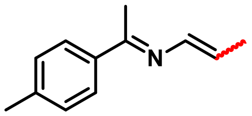

Compound 8b. **Prepared according to general procedure (C)**. (90 ˚C, 18h, 93% yield, *E/Z* 1.8:1.0 (**5 mol%, Co-Me**)) ***E*-8b**: ^{1}H NMR (400 MHz, $CDCl_3$): δ (ppm) 7.78 (d, *J* = 8.1 Hz, 2H), 7.22 – 7.19 (m, 2H), 7.12 (d, *J* = 13.2 Hz, 1H), 6.08 (dq, *J* = 13.8, 7.0 Hz, 1H), 2.38 (s, 3H), 2.30 (s, 3H), 1.88 (d, *J* = 6.9 Hz, 3H). ^{13}C {^{1}H} NMR (101 MHz, $CDCl_3$): δ (ppm), 161.48, 139.89, 138.07, 137.33, 129.07, 127.02, 126.92, 21.43, 16.13, 15.39. ***Z*-8b**: ^{1}H NMR (400 MHz, $CDCl_3$): 7.86 (d, *J* = 8.1 Hz, 2H), 7.22 – 7.19 (m, 2H), 7.00 (d, *J* = 9.1 Hz, 1H), 5.46 (p, *J* = 7.1 Hz, 1H), 2.39 (s, 3H), 2.28 (s, 3H), 1.98 (d, *J* = 6.9 Hz, 3H). ^{13}C {^{1}H} NMR (101 MHz, $CDCl_3$): δ (ppm) 161.9, 140.0, 138.0, 135.2, 129.0, 127.0, 122.5, 21.4, 16.1, 12.3. NMR spectra are consistent with previous reported data.[24]

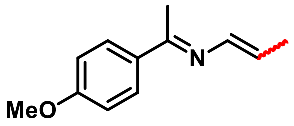

Compound 8c. **Prepared according to general procedure (C)**. (90 ˚C, 18h, 95% yield, *E/Z* 2.7:1.0 (**5 mol%, Co-Me**)) ***E*-8c**: ^{1}H NMR (400 MHz, $CDCl_3$): δ (ppm) 7.85 (d, *J* = 8.8 Hz, 2H), 7.10 (d, *J* = 12.9 Hz, 1H), 6.92 – 6.89 (m, 2H), 6.05 (dq, *J* = 13.9, 7.0 Hz, 1H), 3.84 (s, 3H), 2.29 (s, 3H), 1.87 (d, *J* = 6.9 Hz, 3H). ^{13}C {^{1}H} NMR (101 MHz, $CDCl_3$): δ (ppm), 161.06, 160.88, 137.31, 133.47, 128.51, 126.45, 113.63, 55.43, 16.11, 15.21. ***Z*-8c**: ^{1}H NMR (400 MHz, $CDCl_3$): 7.93 (d, *J* = 8.8 Hz, 2H), 6.98 (d, *J* = 7.9 Hz, 1H), 6.92 – 6.89 (m, 2H), 5.43 (p, *J* = 7.1 Hz, 1H), 3.84 (s, 3H), 2.27 (s, 3H), 1.97 (d, *J* = 6.9 Hz, 3H). ^{13}C {^{1}H} NMR (101 MHz, $CDCl_3$): δ (ppm) 161.3, 161.2, 137.8, 135.2, 128.6, 122.1, 113.6, 55.4, 16.1, 12.3. NMR spectra are consistent with previous reported data.[24]

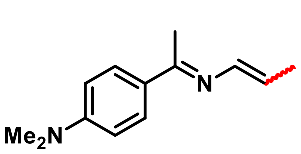

Compound 8d. **Prepared according to general procedure (C)**. (90 °C, 18h, 91% yield, *E/Z* 3.8:1.0 (**5 mol%, Co-Me**)) ***E*-8d**: ^{1}H NMR (400 MHz, $CDCl_3$): δ (ppm) 7.82 (d, *J* = 8.8 Hz, 2H), 7.10 (d, *J* = 12.9 Hz, 1H), 6.71 – 6.68 (m, 2H), 6.00 (td, *J* = 13.6, 6.8 Hz, 1H), 3.01 (s, 3H), 2.27 (s, 3H), 1.86 (d, *J* = 6.9 Hz, 3H). ^{13}C {^{1}H} NMR (101 MHz, $CDCl_3$): δ (ppm), 161.39, 151.59, 137.48, 128.48, 128.33, 124.88, 111.53, 40.36, 16.10, 14.94. ***Z*-8d**: ^{1}H NMR (400 MHz, $CDCl_3$): 7.89 (d, *J* = 8.9 Hz, 2H), 6.99 (d, *J* = 9.1 Hz, 1H), 6.71 – 6.68 (m, 2H), 5.40 – 5.32 (m, 1H), 3.01 (s, 3H), 2.25 (s, 3H), 1.96 (d, *J* = 6.8 Hz, 3H). ^{13}C {^{1}H} NMR (101 MHz, $CDCl_3$): δ (ppm) 161.7, 151.6, 135.5, 128.6, 128.4, 120.6, 111.5, 40.3, 16.1, 12.2. HRMS (APCI, positive ion, m/z): calcd. for ($[C_{13}H_{18}N_2+H]^+$): 203.1548; found 203.1547.

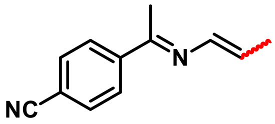

Compound 8e. **Prepared according to general procedure (C)**. (90 °C, 6h, 93% yield, *E/Z* 1.5:1.0 (**5 mol%, Co-Me**)) ***E*-8e**: ^{1}H NMR (400 MHz, $CDCl_3$): δ (ppm) 7.96 (d, *J* = 8.3 Hz, 2H), 7.7 – 7.65 (m, 2H), 7.13 (d, *J* = 12.8 Hz, 1H), 6.20 (dq, *J* = 14.0, 7.1 Hz, 1H), 2.31 (s, 3H), 1.90 (d, *J* = 7.0 Hz, 3H). ^{13}C {^{1}H} NMR (101 MHz, $CDCl_3$): δ (ppm) 159.05, 144.68, 137.04, 132.16, 127.47, 126.30, 118.93, 112.90, 16.28, 15.25. ***Z*-8e**: ^{1}H NMR (400 MHz, $CDCl_3$): 8.03 (d, *J* = 8.3 Hz, 2H), 7.7 – 7.65 (m, 2H), 7.02 (d, *J* = 8.9 Hz, 1H), 5.62 (p, *J* = 7.1 Hz, 1H), 2.27 (s, 3H), 2.00 (d, *J* = 6.9 Hz, 3H). ^{13}C {^{1}H} NMR (101 MHz, $CDCl_3$): δ (ppm) 159.6, 144.6, 134.7, 132.1, 130.4, 127.5, 118.9, 113.0, 15.2, 12.5. NMR spectra are consistent with previous reported data.[24]

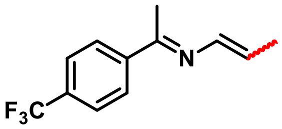

Compound 8f. **Prepared according to general procedure (C)**. (90 °C, 18h, 93% yield, *E/Z* 2.4:1.0 (**5 mol%, Co-Me**)) ***E*-8f**: ^{1}H NMR (400 MHz, $CDCl_3$): δ (ppm) 7.97 (d, *J* = 8.3 Hz, 2H), 7.66 – 7.62 (m, 2H), 7.14 (d, *J* = 12.9 Hz, 1H), 6.17 (dq, *J* = 13.9, 7.0 Hz, 1H), 2.33 (s, 3H), 1.90 (d, *J* = 7.0 Hz, 3H). ^{13}C {^{1}H} NMR (101 MHz, $CDCl_3$): δ (ppm) 159.80, 144.04, 137.11, 131.48, 129.48, 127.24, 125.62, 125.38 – 125.23 (m), 16.22, 15.45. ***Z*-8f**: ^{1}H NMR (400 MHz, $CDCl_3$): 8.04 (d, *J* = 8.3 Hz, 2H), 7.66 – 7.62 (m, 2H), 7.02 (d, *J* = 7.8 Hz, 1H), 5.58 (p, *J* = 7.1 Hz, 1H), 2.31 (s, 3H), 2.00 (d, *J* = 6.9 Hz, 3H). ^{13}C {^{1}H} NMR (101 MHz, $CDCl_3$): δ (ppm) 160.4, 144.0, 134.9, 131.1, 127.3, 125.4 – 125.2 (m), 125.1, 122.9, 15.4, 12.4. HRMS (APCI, positive ion, m/z): calcd. for ($[C_{12}H_{12}F_3N+H]^+$): 228.1000; found 228.1023.

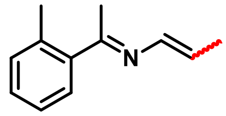

Compound 8g. **Prepared according to general procedure (C)**. (90 ˚C, 24h, 97% yield, *EE/ZZ* 9.2:1 (**5 mol%, Co-Me**)) ***EE*-8g**: ^{1}H NMR (400 MHz, $CDCl_3$): δ (ppm) 7.30 – 7.16 (m, 3H), 7.00 (d, *J* = 6.4 Hz, 1H), 6.31 (d, *J* = 13.1 Hz, 1H), 6.09 – 5.97 (m, 1H), 2.31 (s, 3H), 2.16 (s, 3H), 1.66 (d, *J* = 6.9 Hz, 3H). ^{13}C {^{1}H} NMR (101 MHz, $CDCl_3$): δ (ppm), 167.48, 139.35, 137.79, 135.92, 133.80, 130.28, 128.33, 127.11, 126.10, 28.59, 19.37, 15.67. ***EZ*-8g**: ^{1}H NMR (400 MHz, $CDCl_3$): 7.30 – 7.16 (m, 3H), 7.07 (d, *J* = 13.0 Hz, 1H), 6.22 (d, *J* = 7.7 Hz, 1H), 5.23 (p, *J* = 7.1 Hz, 1H), 2.35 (s, 3H), 2.15 (s, 3H), 1.96 (d, *J* = 6.9 Hz, 3H). ^{13}C {^{1}H} NMR (101 MHz, $CDCl_3$): δ (ppm) 168.5, 139.6, 136.8, 135.0, 133.8, 130.2, 128.2, 126.0, 123.5, 28.8, 19.6, 12.2. NMR spectra are consistent with previous reported data.[24]

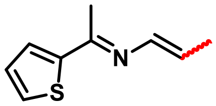

Compound 8h. **Prepared according to general procedure (C)**. (90 ˚C, 18h, 91% yield, *E/Z* 2.2:1.0 (**5 mol%, Co-Me**)) ***E*-8h**: ^{1}H NMR (400 MHz, $CDCl_3$): δ (ppm) 7.38 – 7.35 (m, 2H), 7.07 – 7.01 (m, 2H), 6.07 (dq, *J* = 13.9, 7.0 Hz, 1H), 2.30 (d, *J* = 7.7 Hz, 3H), 1.86 (d, *J* = 7.0 Hz, 3H). ^{13}C {^{1}H} NMR (101 MHz, $CDCl_3$): δ (ppm) 156.23, 147.90, 136.58, 129.24, 127.56, 127.34, 16.12, 15.49. ***Z*-8h**: ^{1}H NMR (400 MHz, $CDCl_3$): 7.38 – 7.35 (m, 2H), 7.07 – 7.01 (m, 1H), 6.95 (d, *J* = 7.8 Hz, 1H), 5.48 (p, *J* = 7.1 Hz, 1H), 2.30 (s, 3H), 1.98 (d, *J* = 6.9 Hz, 3H). ^{13}C {^{1}H} NMR (101 MHz, $CDCl_3$): δ (ppm) 156.8, 148.4, 134.4, 129.4, 127.6, 127.3, 124.0, 15.4, 12.4. NMR spectra are consistent with previous reported data.[24]

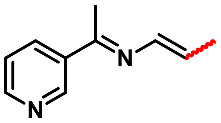

Compound 8i. **Prepared according to general procedure (C)**. (90 ˚C, 8h, 89% yield, *E/Z* 2.1:1 (**5 mol%, Co-Me**)) ***E*-8i**: ^{1}H NMR (400 MHz, $CDCl_3$): δ (ppm) 9.2 – 9.05 (m, 1H), 8.70 – 8.60 (m, 1H), 8.17 (d, *J* = 7.9 Hz, 1H), 7.34 – 7.28 (m, 1H), 7.11 (d, *J* = 11.5 Hz, 1H), 6.13 (dq, *J* = 13.9, 7.0 Hz, 1H), 2.30 (s, 3H), 1.87 (d, *J* = 6.9 Hz, 3H). ^{13}C {^{1}H} NMR (101 MHz, $CDCl_3$): δ (ppm) 158.79, 150.47, 148.52, 136.88, 136.16, 134.13, 129.20, 123.40, 16.13, 15.18. ***Z*-8i**: ^{1}H NMR (400 MHz, $CDCl_3$): 9.2 – 9.05 (m, 1H), 8.70 – 8.60 (m, 1H), 8.24 (d, *J* = 7.9 Hz, 1H), 7.34 – 7.28 (m, 1H), 7.00 (d, *J* = 7.8 Hz, 1H), 5.55 (p, *J* = 7.1 Hz, 1H), 2.29 (s, 3H), 1.97 (d, *J* = 6.9 Hz, 3H). ^{13}C {^{1}H} NMR (101 MHz, $CDCl_3$): δ (ppm) 159.4, 150.5, 148.6, 136.2, 134.6, 134.2, 125.0, 123.5, 15.1, 12.3. NMR spectra are consistent with previous reported data.[24]

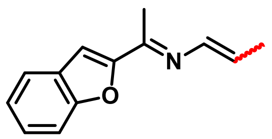

Compound 8j. **Prepared according to general procedure (C)**. (50% Et_2O in pentane, pale yellow oil, 90 °C, 18h, 70% (51%) yield, *E/Z* 1.7:1 (**5 mol%, Co-Me**)) ***E*-8j**: ^{1}H NMR (400 MHz, $CDCl_3$): δ (ppm) 7.63 – 7.53 (m, 2H), 7.34 – 7.31 (m, 1H), 7.26 – 7.20 (m, 1H), 7.17 (s, 1H), 7.13 (d, *J* = 12.9 Hz, 1H), 6.29 (td, *J* = 14.0, 7.0 Hz, 1H), 2.32 (s, 3H), 1.90 (d, *J* = 6.9 Hz, 3H). ^{13}C {^{1}H} NMR (101 MHz, $CDCl_3$): δ (ppm) 155.66, 155.53, 152.50, 136.81, 130.07, 128.31, 126.14, 123.26, 121.84, 112.12, 108.68, 16.37, 14.98. ***Z*-8j**: ^{1}H NMR (400 MHz, $CDCl_3$): 7.63 – 7.53 (m, 2H), 7.34 – 7.31 (m, 1H), 7.26 – 7.20 (m, 2H), 7.03 (d, *J* = 7.8 Hz, 1H), 5.61 – 5.52 (m, 1H), 2.32 (s, 3H), 2.04 (d, *J* = 6.9 Hz, 3H). ^{13}C {^{1}H} NMR (101 MHz, $CDCl_3$): δ (ppm) 155.9, 155.6, 153.8, 134.8, 128.3, 126.0, 125.3, 123.2, 121.9, 112.0, 108.1, 15.0, 12.6. HRMS (APCI, positive ion, m/z): calcd. for ($[C_{13}H_{13}NO+H]^+$): 200.1075; found 200.1095.

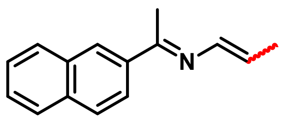

Compound 8k. **Prepared according to general procedure (C)**. (50% Et_2O in pentane, colorless oil, 90 °C, 18h, 89% (59%) yield, *E/Z* 2.5:1 (**5 mol%, Co-Me**)) ***E*-8k**: ^{1}H NMR (400 MHz, $CDCl_3$): δ (ppm) 8.25 (s, 1H), 8.18 (dd, *J* = 8.7, 1.6 Hz, 1H), 7.87 – 7.84 (m, 3H), 7.54 – 7.46 (m, 2H), 7.21 (d, *J* = 12.9 Hz, 1H), 6.19 (dq, *J* = 13.9, 7.0 Hz, 1H), 2.44 (s, 3H), 1.93 (d, *J* = 7.0 Hz, 3H). ^{13}C {^{1}H} NMR (101 MHz, $CDCl_3$): δ (ppm) 161.24, 137.42, 134.20, 133.17, 128.91, 127.94, 127.90, 127.73, 127.08, 126.88, 126.29, 124.37, 16.23, 15.37. ***Z*-8k**: ^{1}H NMR (400 MHz, $CDCl_3$): 8.33 – 8.26 (m, 2H), 7.93 – 7.88 (m, 3H), 7.56 – 7.46 (m, 2H), 7.09 (d, *J* = 9.4 Hz, 1H), 5.59 – 5.50 (m, 1H), 2.43 (s, 3H), 2.06 (d, *J* = 8.3 Hz, 3H). ^{13}C {^{1}H} NMR (101 MHz, $CDCl_3$): δ (ppm) 161.7, 138.1, 135.3, 134.3, 128.9, 127.9, 127.9, 127.7, 127.2, 126.9, 124.4, 123.5, 15.3, 12.4. HRMS (APCI, positive ion, m/z): calcd. for ($[C_{15}H_{15}N+H]^+$): 210.1283; found 210.1276.

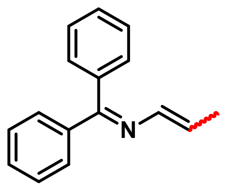

Compound 8l. **Prepared according to general procedure (C)**. (40% Et_2O in pentane, pale yellow oil, 90 °C, 18h, 90% (84%) yield, *E/Z* 2.1:1 (Internal standard 1 mg) (**5 mol%, Co-Me**)) ***E*-8l**: ^{1}H NMR (400 MHz, $CDCl_3$): δ (ppm) 7.70 – 7.68 (m, 2H), 7.52 – 7.43 (m, 3H), 7.42 – 7.32 (m, 3H), 7.25 – 7.19 (m, 2H), 6.78 (dd, *J* = 12.9, 1.6 Hz, 1H), 6.27 (dq, *J* = 14.1, 7.1 Hz, 1H), 1.77 (dd, *J* = 7.1, 1.6 Hz, 3H). ^{13}C {^{1}H} NMR (101 MHz, $CDCl_3$): 164.55, 138.92, 136.60, 130.16, 128.87, 128.85, 128.74, 128.58, 128.21, 126.07, 16.05. ***Z*-8l**: ^{1}H NMR (400 MHz, $CDCl_3$): 7.78 – 7.76 (m, 2H), 7.52 – 7.43 (m, 3H), 7.42 – 7.32 (m, 3H), 7.25 – 7.19 (m, 2H), 6.70 (dd, *J* = 7.7, 1.8 Hz, 1H), 5.54 – 5.38 (m, 1H), 2.13 (dd, *J* = 7.0, 1.8 Hz, 1H). ^{13}C {^{1}H} NMR (101 MHz, $CDCl_3$): 164.7, 139.9, 139.7, 136.7, 130.2, 129.2, 128.8, 128.7, 128.5, 128.2, 12.6. NMR spectra are consistent with previous reported data.[27]

General procedure for selective isomerization of N-allyl substrate at gram scale (D). Inside the glovebox, an oven-dried 100 mL Schlenk tube was charged with substrate (5.8 mmol (**2a**) or 4.5 mmol (**4l**)) and catalyst **Co-Me** in toluene (0.174 mmol (3 mol%) to 0.225 mmol (5 mol%)) was added. To the reaction mixture was added an additional amount of toluene to maintain 0.38M solution and the Schlenk tube was sealed and immersed in a pre-heated oil bath at 90 ˚C for 18h. After completion of the reaction, the Schlenk tube with imine compound was transferred to the glovebox and the crude reaction mixture was filtered through short alumina plug to remove the cobalt catalyst otherwise all the manipulations were carried out under air. The alumina was washed with an additional DCM or EtOAc to collect all the organic products. The isomerized products were isolated by flash column chromatography using SiO_2 (eluent as 20% EtOAc in hexane; **6a**) and neutral alumina (eluent as 40% Et_2O in pentane; **8l**). Yield: 0.93 g (93%, **6a**) or 0.86 g (86%, **8l).**

General method for the synthesis of 2-phenylpyridine derivatives (E). Inside the glovebox, a Schlenk tube was charged with crude 2-azadiene (0.25 mmol, 1 equiv.), enamine ester (**11**, 0.5 mmol, 2 equiv.), $MgBr_2{\cdot}Et_2O$ (0.5 mmol, 2 equiv.) and 4 Å mol. sieves (100 mg) in toluene (5 mL). The tube was sealed and the crude mixture was heated at 70 ˚C for 12h, whereafter it was cooled to room temperature. Under an inert atmosphere, Pd/C (125 mg, 5 wt. %, 0.059 based on metal) was added to the crude reaction mixture, which was refluxed at 130 ˚C for an additional 12h. Finally, the cooled mixture was loaded on silica gel column and the product was obtained by eluting with ethyl acetate and hexane mixture (0-40%). ^1H and ^{13}C NMR spectroscopy was used to determine yield and purity of the products.

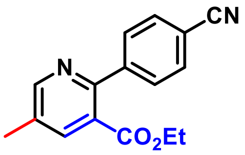

Compound 9a. Prepared according to general procedure (E). Purified by column chromatography (25% EtOAc in hexane, White solid, 23 mg (34%)). ^1H NMR (400 MHz, $CDCl_3$): δ (ppm) 8.61 (d, J = 2.0 Hz, 1H), 8.00 (d, J = 2.0 Hz, 1H), 7.71 (d, J = 8.2 Hz, 2H), 7.60 (d, J = 8.3 Hz, 2H), 4.17 (q, J = 7.1 Hz, 2H), 2.44 (s, 3H), 1.10 (t, J = 7.1 Hz, 3H).). ^{13}C {^1H} NMR (101 MHz, $CDCl_3$): δ (ppm) 167.2, 154.6, 152.1, 144.8, 138.8, 132.8, 131.9, 129.5, 126.6, 118.9, 112.1, 61.8, 18.1, 13.8. HRMS (APCI, positive ion, m/z): calcd. for ($[C_{16}H_{14}N_2O_2+H]^+$): 267.1134; found 267.1146.

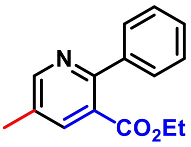

Compound 9b. Prepared according to general procedure (E). Purified by column chromatography (15% EtOAc in hexane, Colourless oil, 19 mg (32%)). ^1H NMR (400 MHz, $CDCl_3$): δ (ppm) 8.58 (d, J = 2.1 Hz, 1H), 7.90 (d, J = 2.1 Hz, 1H), 7.51 – 7.49 (m, 2H), 7.43 – 7.37 (m, 3H), 4.13 (q, J = 7.1 Hz, 2H), 2.41 (s, 3H), 1.03 (t, J = 7.1 Hz, 3H). ^{13}C {^1H} NMR (101 MHz, $CDCl_3$): δ (ppm) 168.4,

156.3, 151.7, 140.2, 138.2, 131.4, 128.6, 128.4, 128.1, 126.9, 61.5, 18.0, 13.7. NMR spectra are consistent with previous reported data.[28]

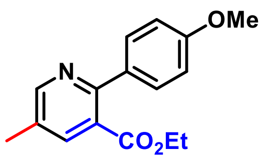

Compound 9c. Prepared according to general procedure (E). Purified by column chromatography (20% EtOAc in hexane, Colourless oil, 16 mg (23%)). ^{1}H NMR (400 MHz, $CDCl_3$): δ (ppm) 8.56 (d, *J* = 2.1 Hz, 1H), 7.86 (d, *J* = 2.1 Hz, 1H), 7.48 (d, *J* = 8.6 Hz, 2H), 6.95 (d, *J* = 8.6 Hz, 2H), 4.19 (q, *J* = 7.2 Hz, 2H), 3.85 (s, 3H), 2.40 (s, 3H), 1.12 (t, *J* = 7.1 Hz, 3H). ^{13}C {^{1}H} NMR (101 MHz, $CDCl_3$): δ (ppm) 168.8, 160.1, 155.7, 151.7, 138.2, 132.7, 130.9, 130.0, 126.6, 113.7, 61.5, 55.4, 18.0, 13.9. HRMS (APCI, positive ion, m/z): calcd. for ($[C_{16}H_{17}NO_3+H]^+$): 272.1287; found 272.1275.

General method for the synthesis of 2-phenylpyridine derivatives (F). Inside the glovebox, a Schlenk tube was charged with N-morpholine allylamine (0.3 mmol, 1.2 equiv.), **Co-Me** (2.5 mol% catalyst) in toluene (0.5 mL). The Schlenk tube was sealed and heated in an oil bath at 90 ˚C for 16 h. Next, the tube was cooled to room temperature and transferred to the glovebox, where N-allyl aldimine (0.25 mmol, 1 equiv.) was added. The tube was again sealed and the mixture further heated at 95 ˚C for 16h. Hereafter, the Schlenk tube was transferred to the glovebox, where $MgBr_2{\cdot}Et_2O$ (0.5 mmol, 2 equiv.) and 4 Å mol. sieves (100 mg) were added to the crude reaction mixture. An additional amount of toluene (5 mL) was added again. The tube was sealed and heated at 90 ˚C for 14h, whereafter it was cooled to room temperature. Finally, under an inert atmosphere, Pd/C (125 mg, 5 wt. %, 0.059 based on metal) was added and the reaction mixture was refluxed at 130 ˚C for an additional 12h. After 12h, the cooled mixture was filtered through a pad of ®Celite and washed with ethyl acetate (10 mL) the resulting solution was evaporated to dryness. The crude mixture was dissolved in ethyl acetate and passed through a short plug of neutral alumina to afford the 2-phenylpyridine as a clean product. ^{1}H and ^{13}C NMR spectroscopy were used to determine the purity and yields of the products.

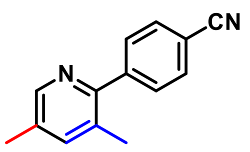

Compound 10a. Prepared according to general procedure (F). Purified by column chromatography (15% EtOAc in hexane, deep yellow oil, 20 mg (38%)). ^{1}H NMR (400 MHz, $CDCl_3$): δ (ppm) 8.36 (s, 1H), 7.73 (d, *J* = 8.2 Hz, 2H), 7.63 (d, *J* = 8.2 Hz, 2H), 7.43 (s, 1H), 2.36 (s, 3H), 2.32 (s, 3H). ^{13}C {^{1}H} NMR (101 MHz, $CDCl_3$): δ (ppm) 153.8, 147.9, 145.2, 139.6, 132.8, 132.1, 130.4, 129.9, 119.0, 111.5, 19.8, 18.1. HRMS (APCI, positive ion, m/z): calcd. for ($[C_{14}H_{12}N_2+H]^+$): 209.1079; found 209.1062.

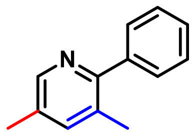

Compound 10b. Prepared according to general procedure (F). Purified by column chromatography (20% EtOAc in hexane, yellow oil, 38 mg (83%)). ^{1}H NMR (400 MHz, $CDCl_3$): δ (ppm) 8.35 (s, 1H), 7.50 (d, J = 7.1 Hz, 2H), 7.42 (d, J = 7.6 Hz, 2H), 7.39 – 7.34 (m, 2H), 2.34 (s, 3H), 2.32 (s, 3H). ^{13}C {^{1}H} NMR (101 MHz, $CDCl_3$): δ (ppm) 156.0, 147.4, 140.7, 139.2, 131.5, 130.2, 129.0, 128.2, 127.7, 20.0, 18.0. NMR spectra are consistent with previous reported data.[29]

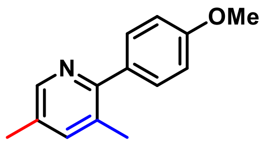

Compound 10c. Prepared according to general procedure (F). Purified by column chromatography (20% EtOAc in hexane, deep yellow oil, 38 mg (71%)). ^{1}H NMR (400 MHz, $CDCl_3$): δ (ppm) 8.32 (s, 1H), 7.46 (d, J = 8.6 Hz, 2H), 7.36 (s, 1H), 6.96 (d, J = 8.7 Hz, 2H), 3.84 (s, 3H), 2.32 (s, 6H). ^{13}C {^{1}H} NMR (101 MHz, $CDCl_3$): δ (ppm) 159.3, 155.6, 147.4, 139.2, 133.2, 131.1, 130.3, 130.1, 113.6, 55.4, 20.2, 18.0. NMR spectra are consistent with previous reported data.[29]

NMR and HRMS of synthesized starting materials.

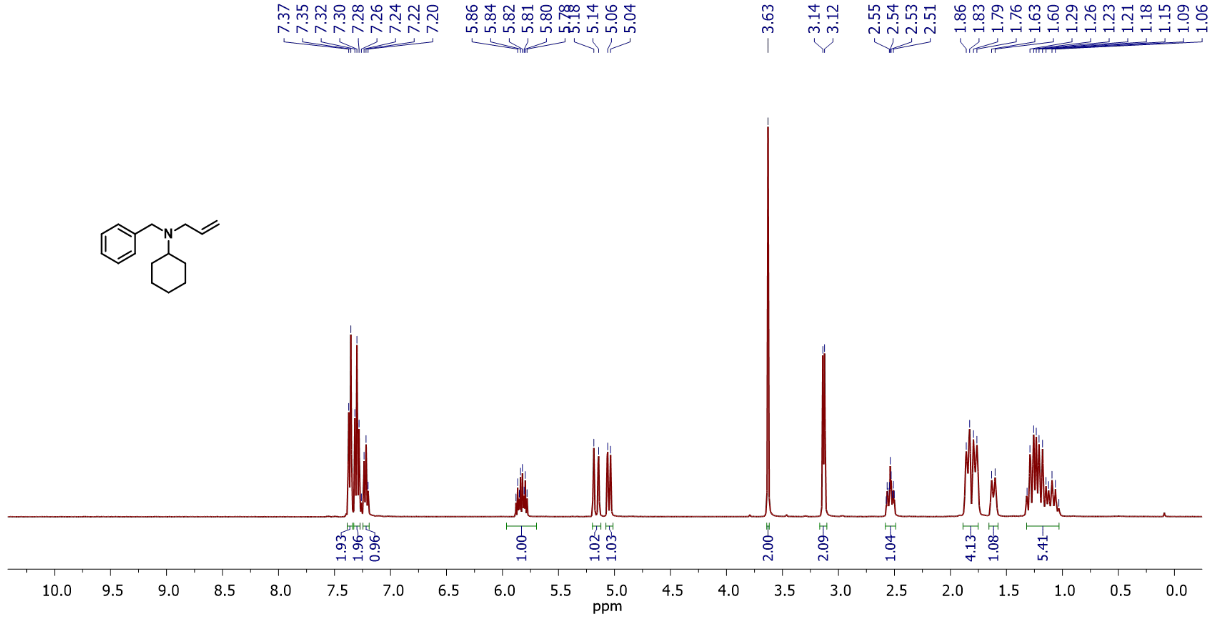

Figure S1. ^{1}H NMR spectrum (400 MHz) of **1g** in $CDCl_3$.

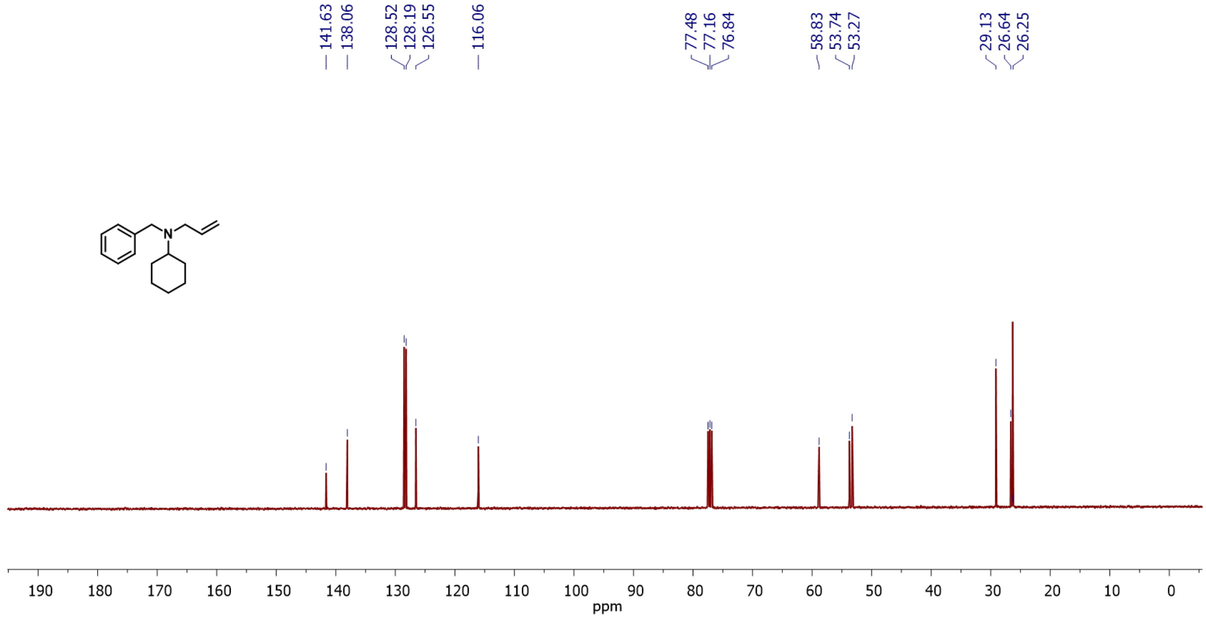

Figure S2. ^{13}C {^{1}H} NMR spectrum (101 MHz) of **1g** in $CDCl_3$.

Compound Spectrum SmartFormula Report

Analysis Info

Analysis Name	D:\Data\Graham\Dr_5211000001.d	Acquisition Date	06/12/2023 13:08:21
Method	APCI_pos_SolidProbe.m	Operator	Larisa Panz
Sample Name	SRK 1g	Instrument	maXis impact 282001.00128
Comment			

Acquisition Parameter

Source Type	APCI	Ion Polarity	Positive	Set Nebulizer	1.2 Bar
Focus	Active	Set Capillary	3000 V	Set Dry Heater	120 °C
Scan Begin	50 m/z	Set End Plate Offset	-500 V	Set Dry Gas	1.5 l/min
Scan End	2000 m/z	Set Charging Voltage	2000 V	Set Divert Valve	Source
		Set Corona	5000 nA	Set APCI Heater	250 °C

+MS, 1.3-1.3min #76-78

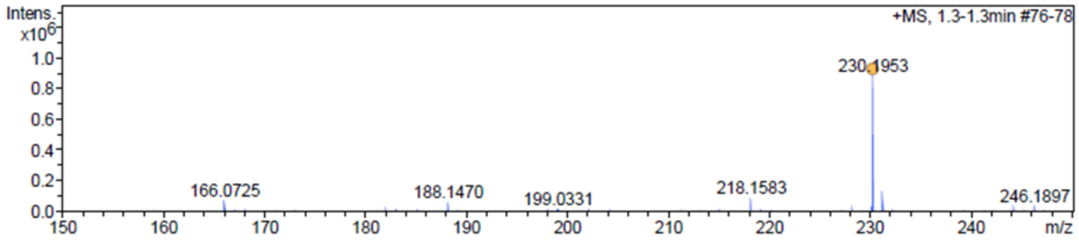

Meas. m/z	#	Ion Formula	m/z	err [ppm]	mSigma	# mSigma	Score	rdb	e⁻ Conf	N-Rule	|err| [mDa]
230.1953	1	C12H24NO3	230.1751	-87.7	9.7	1	0.00	1.5	even	ok	20.2
	2	C16H24N	230.1903	-21.4	14.7	2	100.00	5.5	even	ok	4.9
	3	C8H24NO6	230.1598	-154.0	34.0	3	0.00	-2.5	even	ok	35.4

Figure S3. High resolution mass spectrum of **1g.**

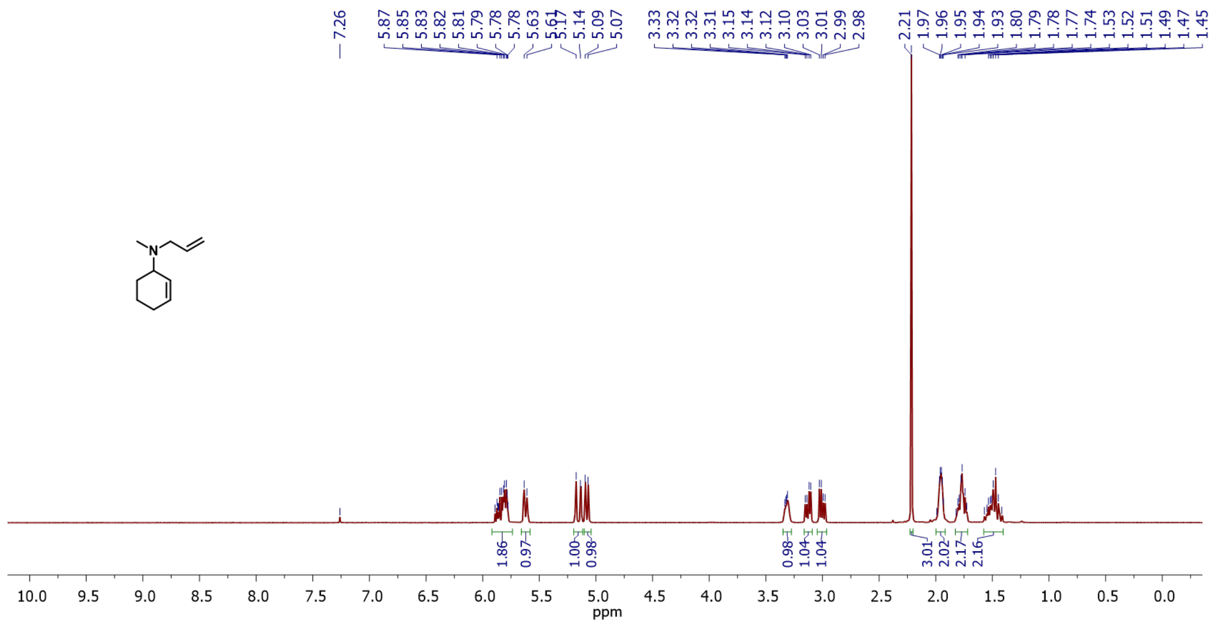

Figure S4. ^{1}H NMR spectrum (400 MHz) of **1i** in $CDCl_3$.

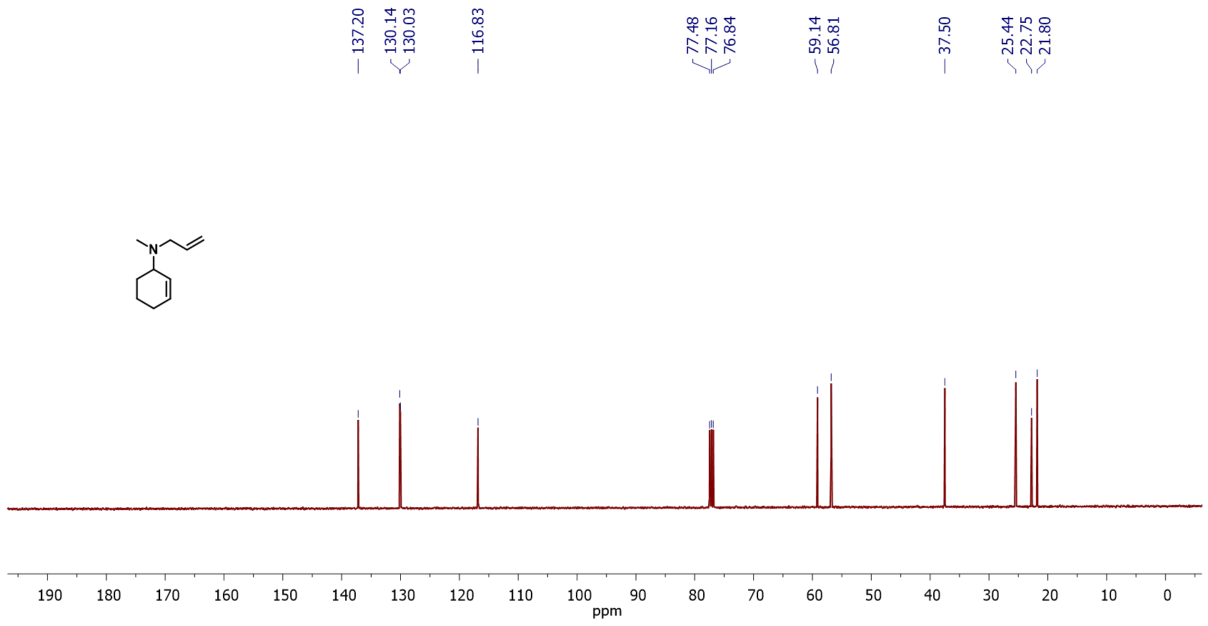

Figure S5. $^{13}C\ \{^{1}H\}$ NMR spectrum (101 MHz) of **1i** in $CDCl_3$.

Compound Spectrum SmartFormula Report

**Analysis Info**			
Analysis Name	D:\Data\Graham\Dr_5209000001.d	Acquisition Date	06/12/2023 13:02:15
Method	APCI_pos_SolidProbe.m	Operator	Larisa Panz
Sample Name	SRK 1I	Instrument	maXis impact 282001.00128
Comment			

Acquisition Parameter

Source Type	APCI	Ion Polarity	Positive	Set Nebulizer	1.2 Bar
Focus	Active	Set Capillary	3000 V	Set Dry Heater	120 °C
Scan Begin	50 m/z	Set End Plate Offset	-500 V	Set Dry Gas	1.5 l/min
Scan End	2000 m/z	Set Charging Voltage	2000 V	Set Divert Valve	Source
		Set Corona	5000 nA	Set APCI Heater	250 °C

+MS, 0.5-0.6min #28-34

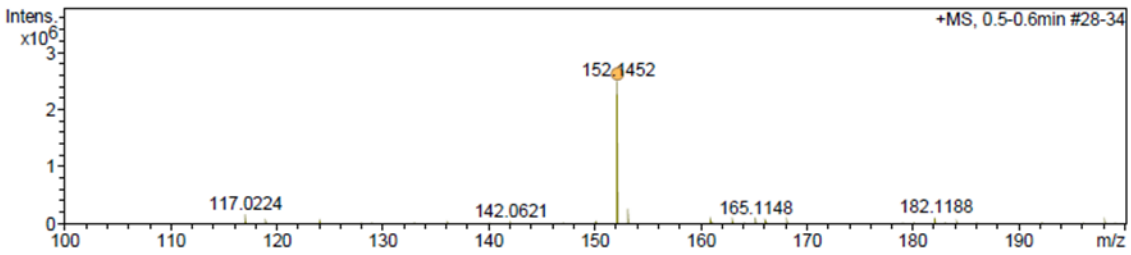

Meas. m/z	#	Ion Formula	m/z	err [ppm]	mSigma	# mSigma	Score	rdb	e⁻ Conf	N-Rule	|err| [mDa]
152.1452	1	C10H18N	152.1434	-12.3	3.9	1	100.00	2.5	even	ok	1.9
	2	C6H18NO3	152.1281	-112.6	20.6	2	0.00	-1.5	even	ok	17.1

Figure S6. High resolution mass spectrum of **1i.**

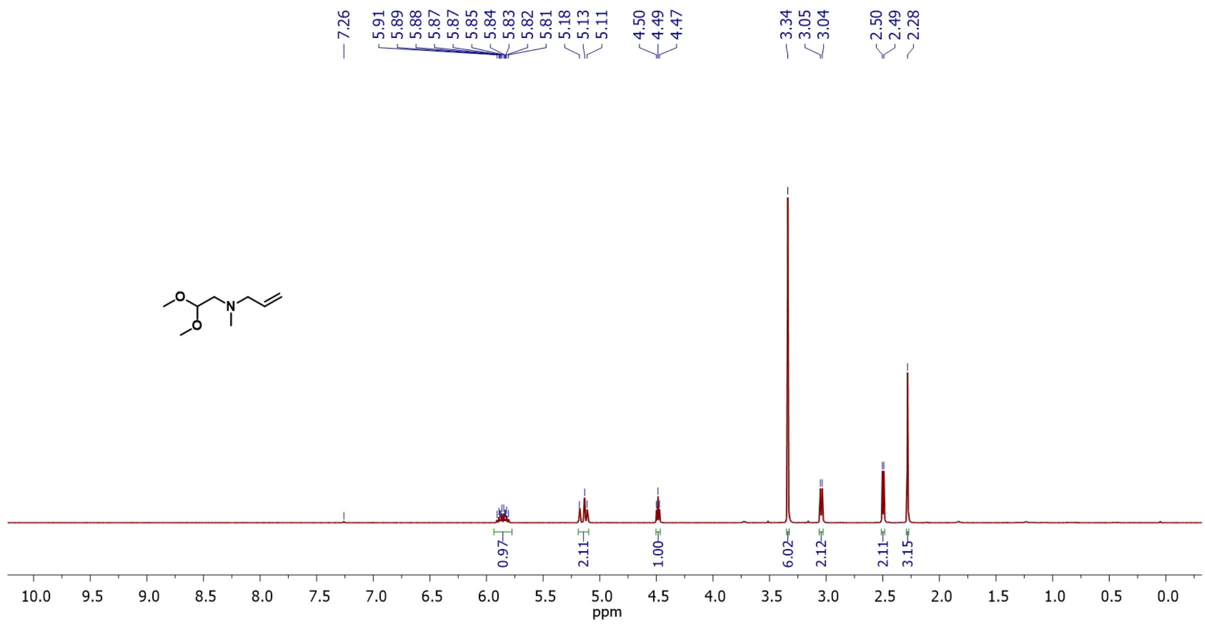

Figure S7. ^{1}H NMR spectrum (400 MHz) of **1k** in $CDCl_3$.

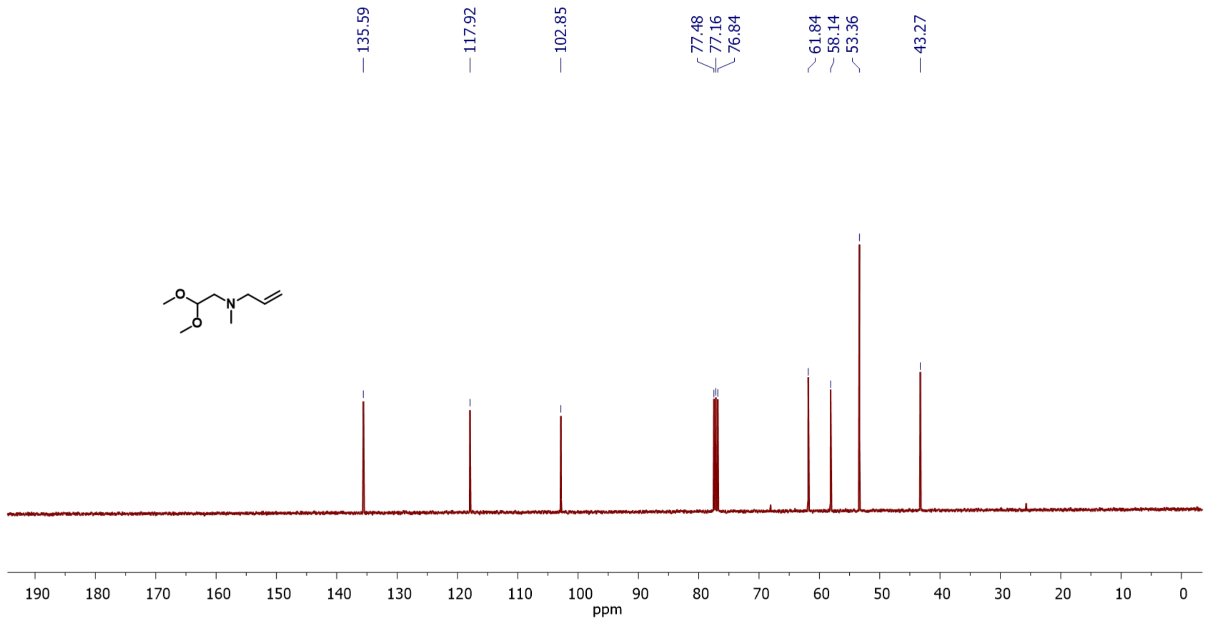

Figure S8. ^{13}C {^{1}H} NMR spectrum (101 MHz) of **1k** in $CDCl_3$.

Compound Spectrum SmartFormula Report

Analysis Info

Analysis Name	D:\Data\Graham\Dr_5212000001.d	Acquisition Date	06/12/2023 13:12:43
Method	APCI_pos_SolidProbe.m	Operator	Larisa Panz
Sample Name	SRK 1K	Instrument	maXis impact 282001.00128
Comment			

Acquisition Parameter

Source Type	APCI	Ion Polarity	Positive	Set Nebulizer	1.2 Bar
Focus	Active	Set Capillary	3000 V	Set Dry Heater	120 °C
Scan Begin	50 m/z	Set End Plate Offset	-500 V	Set Dry Gas	1.5 l/min
Scan End	2000 m/z	Set Charging Voltage	2000 V	Set Divert Valve	Source
		Set Corona	5000 nA	Set APCI Heater	250 °C

+MS, 0.4-0.4min #22-25

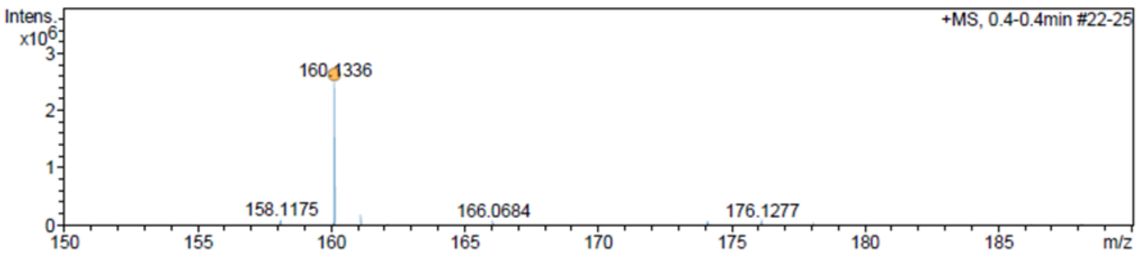

Meas. m/z	#	Ion Formula	m/z	err [ppm]	mSigma	# mSigma	Score	rdb	e⁻ Conf	N-Rule	|err| [mDa]
160.1336	1	C8H18NO2	160.1332	-2.3	10.7	1	100.00	0.5	even	ok	0.4

Figure S9. High resolution mass spectrum of **1k.**

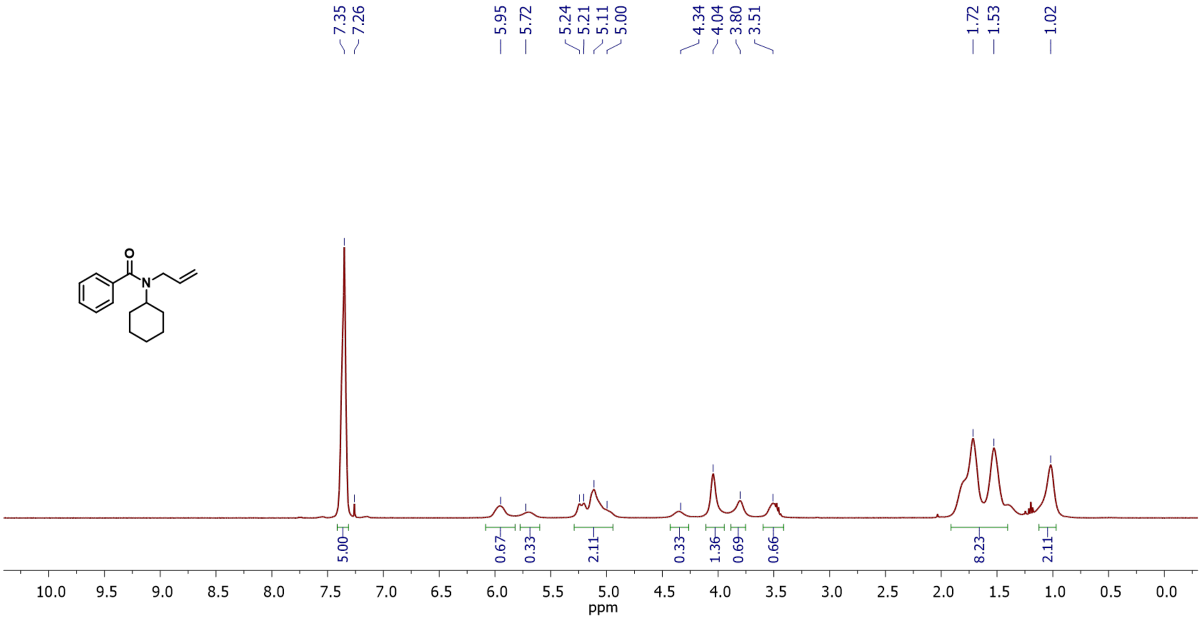

Figure S10. ^{1}H NMR spectrum (400 MHz) of **2j** in $CDCl_3$.

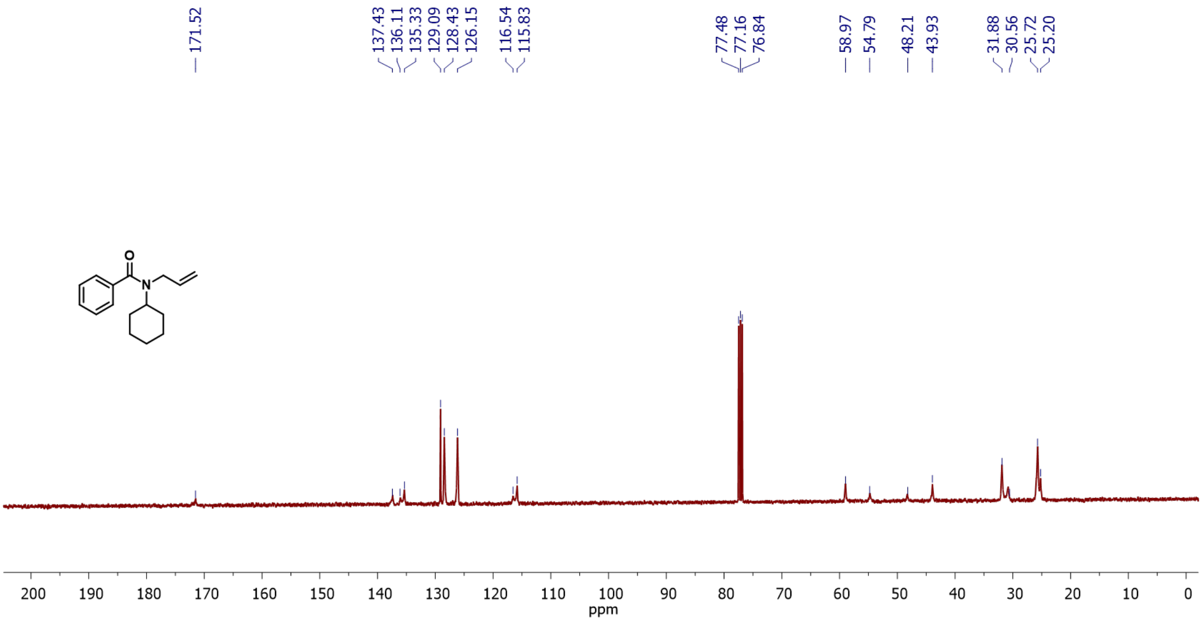

Figure S11. ^{13}C {^{1}H} NMR spectrum (101 MHz) of **2j** in $CDCl_3$.

Compound Spectrum SmartFormula Report

**Analysis Info**		Acquisition Date	06/12/2023 13:20:10
Analysis Name	D:\Data\Graham\Dr_5214000001.d		
Method	APCI_pos_SolidProbe.m	Operator	Larisa Panz
Sample Name	SRK 2j	Instrument	maXis impact 282001.00128
Comment			

Acquisition Parameter

Source Type	APCI	Ion Polarity	Positive	Set Nebulizer	1.2 Bar
Focus	Active	Set Capillary	3000 V	Set Dry Heater	120 °C
Scan Begin	50 m/z	Set End Plate Offset	-500 V	Set Dry Gas	1.5 l/min
Scan End	2000 m/z	Set Charging Voltage	2000 V	Set Divert Valve	Source
		Set Corona	5000 nA	Set APCI Heater	250 °C

+MS, 0.6-0.6min #35-38

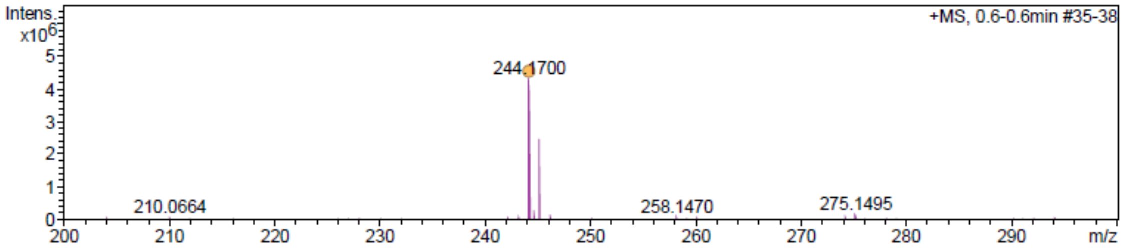

Meas. m/z	#	Ion Formula	m/z	err [ppm]	mSigma	# mSigma	Score	rdb	e⁻ Conf	N-Rule	|err| [mDa]
244.1700	1	C16H22NO	244.1696	-1.6	226.8	1	100.00	6.5	even	ok	0.4

Figure S12. High resolution mass spectrum of **2j**.

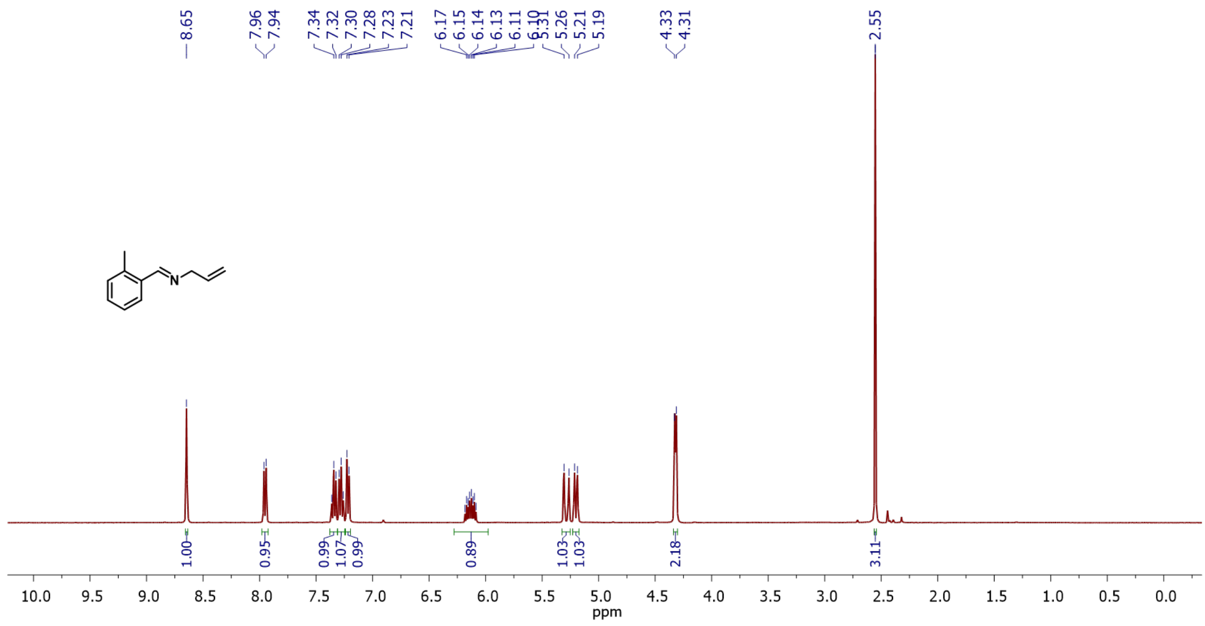

Figure S13. ^{1}H NMR spectrum (400 MHz) of **3g** in $CDCl_3$.

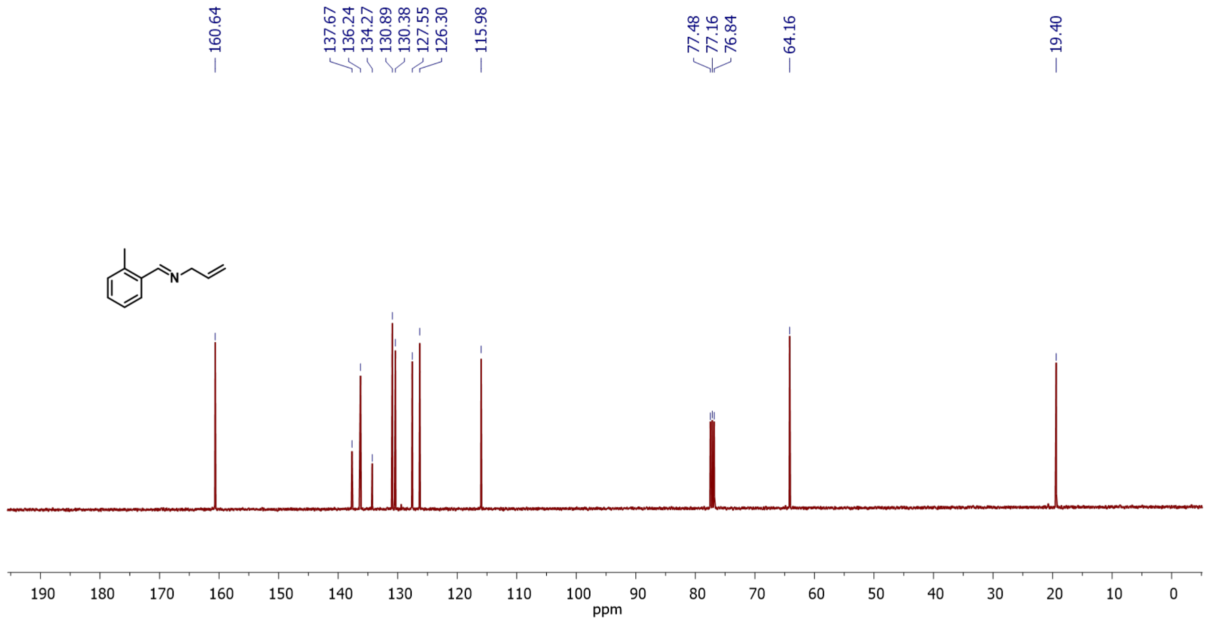

Figure S14. ^{13}C {^{1}H} NMR spectrum (101 MHz) of **3g** in $CDCl_3$.

Compound Spectrum SmartFormula Report

Analysis Info

Analysis Name	D:\Data\Graham\Dr_5213000001.d	Acquisition Date	06/12/2023 13:17:44
Method	APCI_pos_SolidProbe.m	Operator	Larisa Panz
Sample Name	SRK 3g	Instrument	maXis impact 282001.00128
Comment			

Acquisition Parameter

Source Type	APCI	Ion Polarity	Positive	Set Nebulizer	1.2 Bar
Focus	Active	Set Capillary	3000 V	Set Dry Heater	120 °C
Scan Begin	50 m/z	Set End Plate Offset	-500 V	Set Dry Gas	1.5 l/min
Scan End	2000 m/z	Set Charging Voltage	2000 V	Set Divert Valve	Source
		Set Corona	5000 nA	Set APCI Heater	250 °C

+MS, 0.5-0.6min #32-33

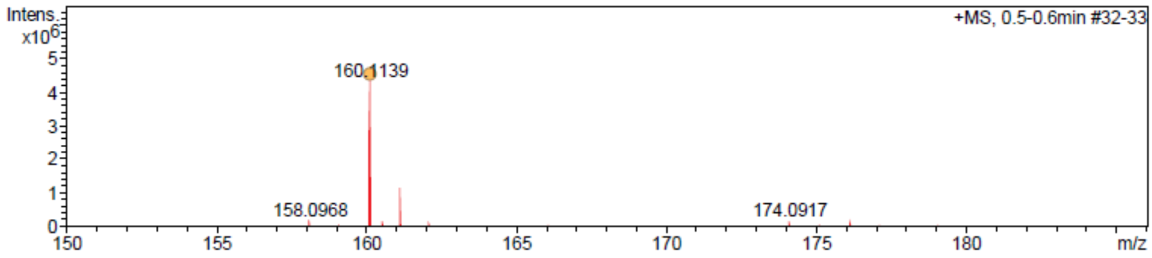

Meas. m/z	#	Ion Formula	m/z	err [ppm]	mSigma	# mSigma	Score	rdb	e⁻ Conf	N-Rule	|err| [mDa]
160.1139	1	C11H14N	160.1121	-11.4	83.3	1	100.00	5.5	even	ok	1.8
	2	C7H14NO3	160.0968	-106.7	107.6	2	0.00	1.5	even	ok	17.1

Figure S15. High resolution mass spectrum of **3g**.

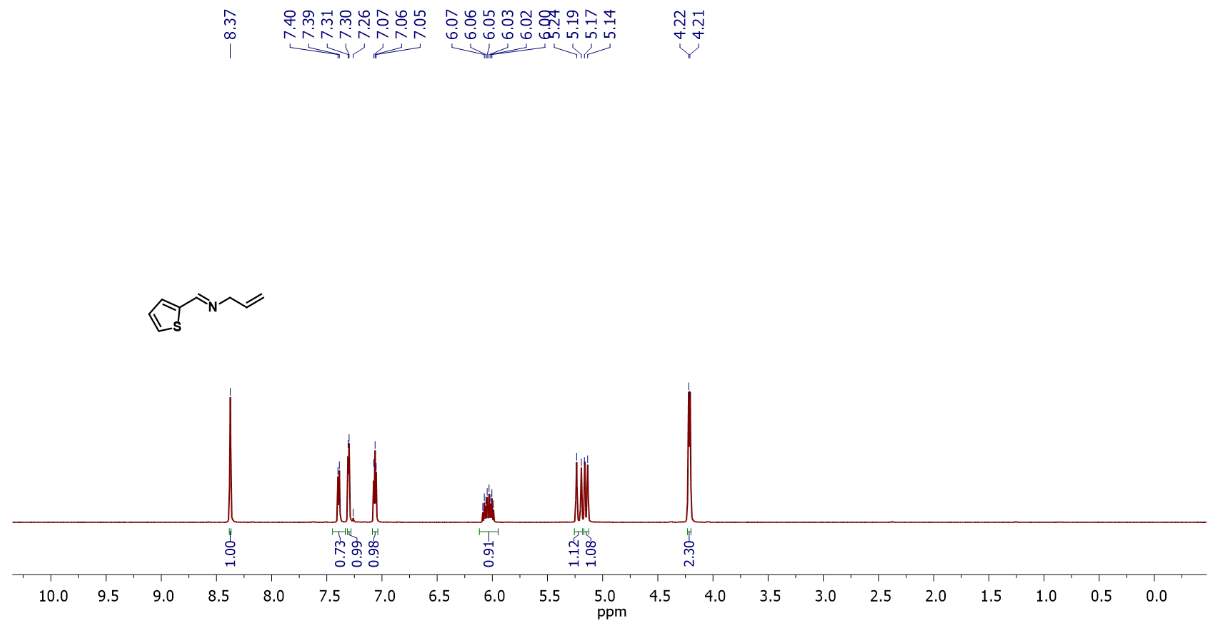

Figure S16. ^{1}H NMR spectrum (400 MHz) of **3h** in $CDCl_3$.

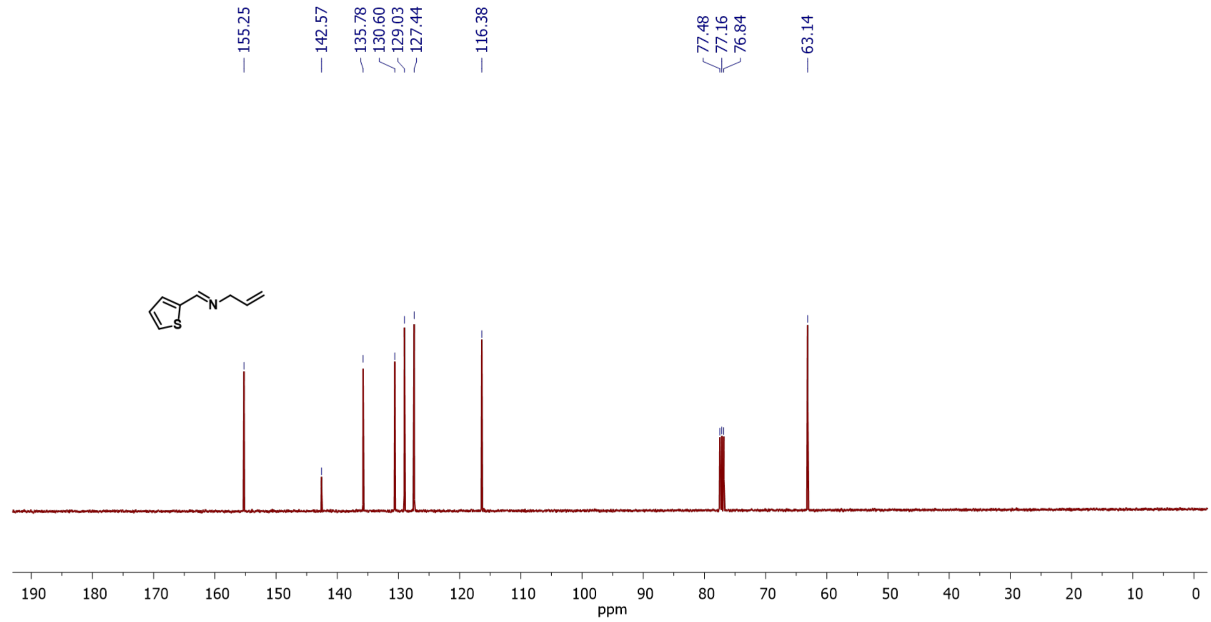

Figure S17. ^{13}C {^{1}H} NMR spectrum (101 MHz) of **3h** in $CDCl_3$.

Compound Spectrum SmartFormula Report

Analysis Info

Analysis Name	D:\Data\Graham\Dr_5215000001.d	Acquisition Date	06/12/2023 13:22:52
Method	APCI_pos_SolidProbe.m	Operator	Larisa Panz
Sample Name	SRK 3h	Instrument	maXis impact 282001.00128
Comment			

Acquisition Parameter

Source Type	APCI	Ion Polarity	Positive	Set Nebulizer	1.2 Bar
Focus	Active	Set Capillary	3000 V	Set Dry Heater	120 °C
Scan Begin	50 m/z	Set End Plate Offset	-500 V	Set Dry Gas	1.5 l/min
Scan End	2000 m/z	Set Charging Voltage	2000 V	Set Divert Valve	Source
		Set Corona	5000 nA	Set APCI Heater	250 °C

+MS, 0.4-0.4min #22-23

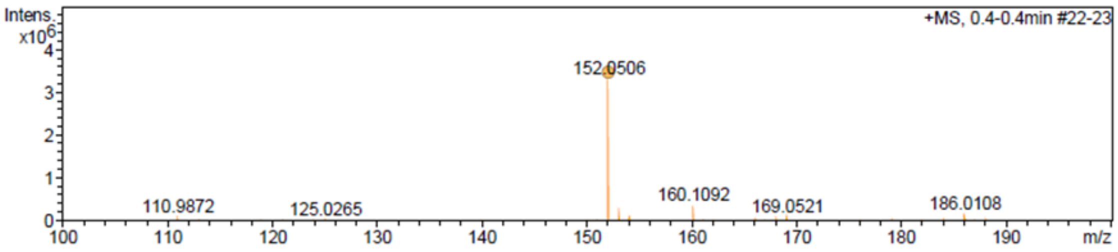

Meas. m/z	#	Ion Formula	m/z	err [ppm]	mSigma	# mSigma	Score	rdb	e⁻ Conf	N-Rule	|err| [mDa]
152.0506	1	C8H10NS	152.0528	14.9	7.8	1	100.00	4.5	even	ok	2.3
	2	C4H10NO3S	152.0376	-85.4	20.9	2	0.00	0.5	even	ok	13.0

Figure S18. High resolution mass spectrum of **3h.**

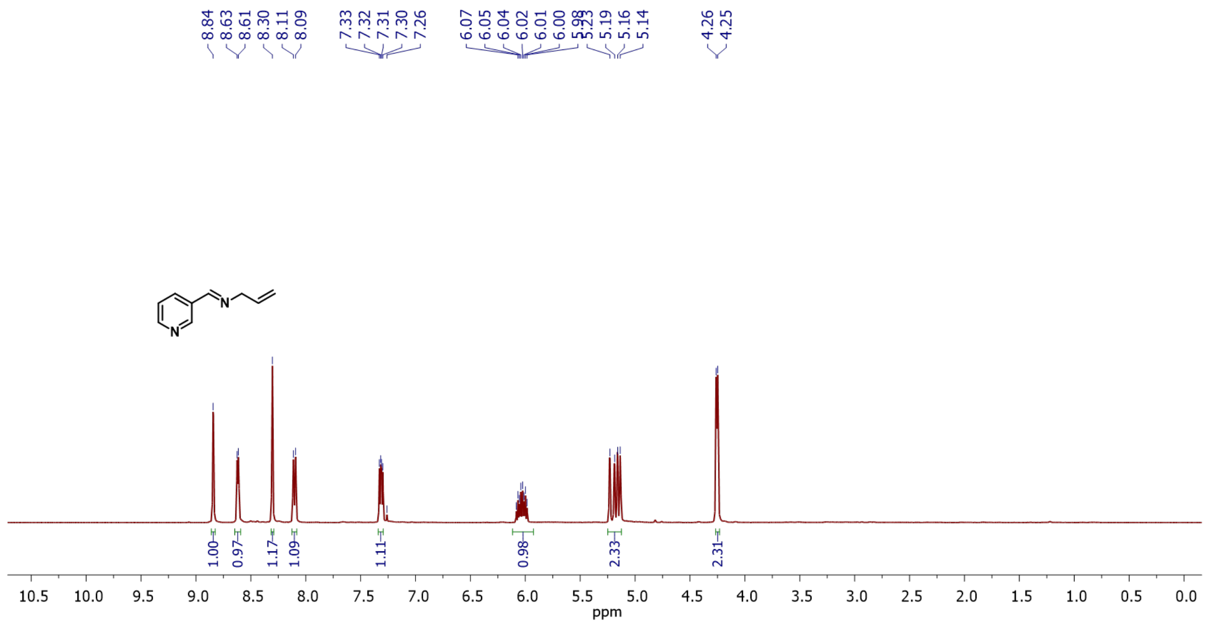

Figure S19. ^{1}H NMR spectrum (400 MHz) of **3i** in $CDCl_3$.

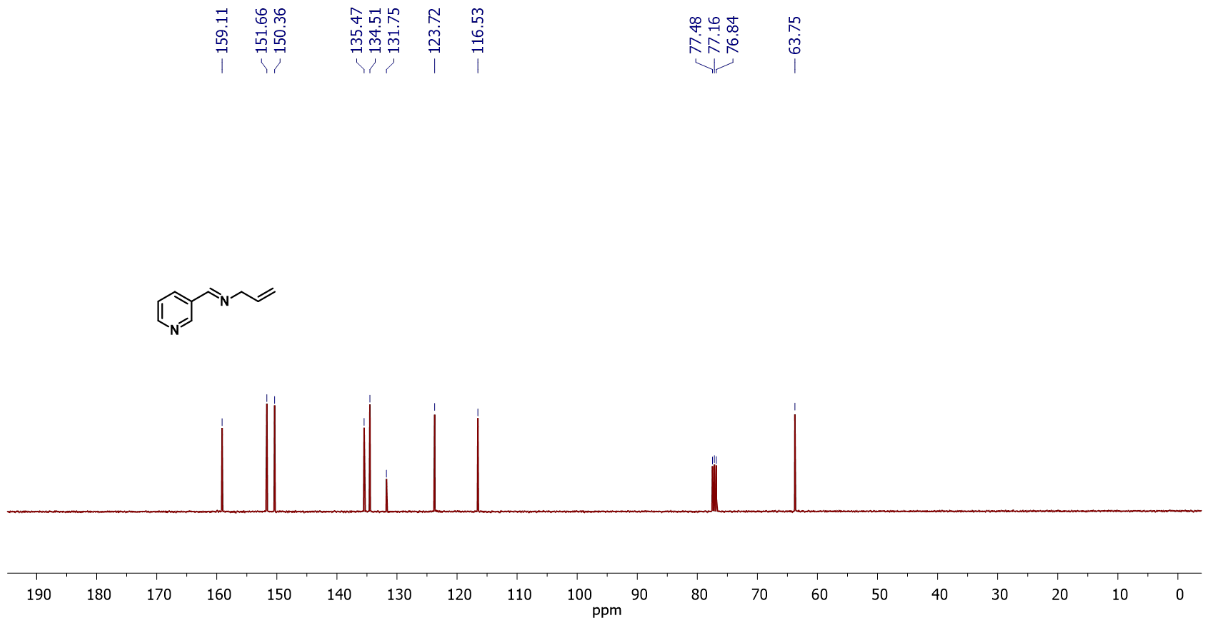

Figure S20. ^{13}C {^{1}H} NMR spectrum (101 MHz) of **3i** in $CDCl_3$.

Compound Spectrum SmartFormula Report

Analysis Info

Analysis Name	D:\Data\Graham\Dr_5217000001.d	Acquisition Date	06/12/2023 13:27:39
Method	APCI_pos_SolidProbe.m	Operator	Larisa Panz
Sample Name	SRK 3i	Instrument	maXis impact 282001.00128
Comment			

Acquisition Parameter

Source Type	APCI	Ion Polarity	Positive	Set Nebulizer	1.2 Bar
Focus	Active	Set Capillary	3000 V	Set Dry Heater	120 °C
Scan Begin	50 m/z	Set End Plate Offset	-500 V	Set Dry Gas	1.5 l/min
Scan End	2000 m/z	Set Charging Voltage	2000 V	Set Divert Valve	Source
		Set Corona	5000 nA	Set APCI Heater	250 °C

+MS, 0.3-0.4min #20-22

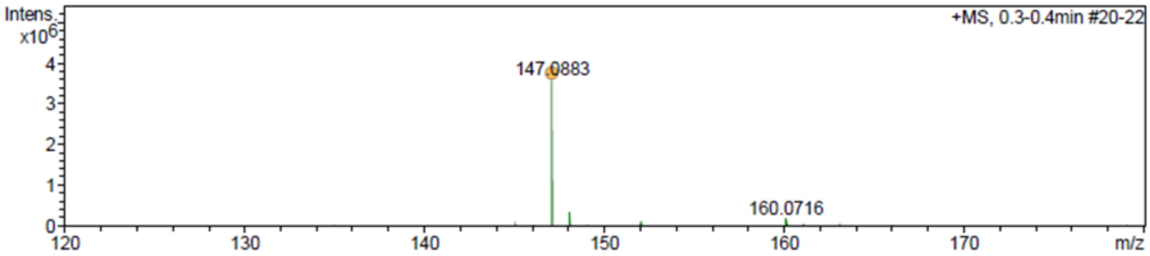

Meas. m/z	#	Ion Formula	m/z	err [ppm]	mSigma	# mSigma	Score	rdb	e⁻ Conf	N-Rule	|err| [mDa]
147.0883	1	C9H11N2	147.0917	22.8	4.5	1	100.00	5.5	even	ok	3.4
	2	C5H11N2O3	147.0764	-80.9	20.0	2	0.00	1.5	even	ok	11.9

Figure S21. High resolution mass spectrum of **3i.**

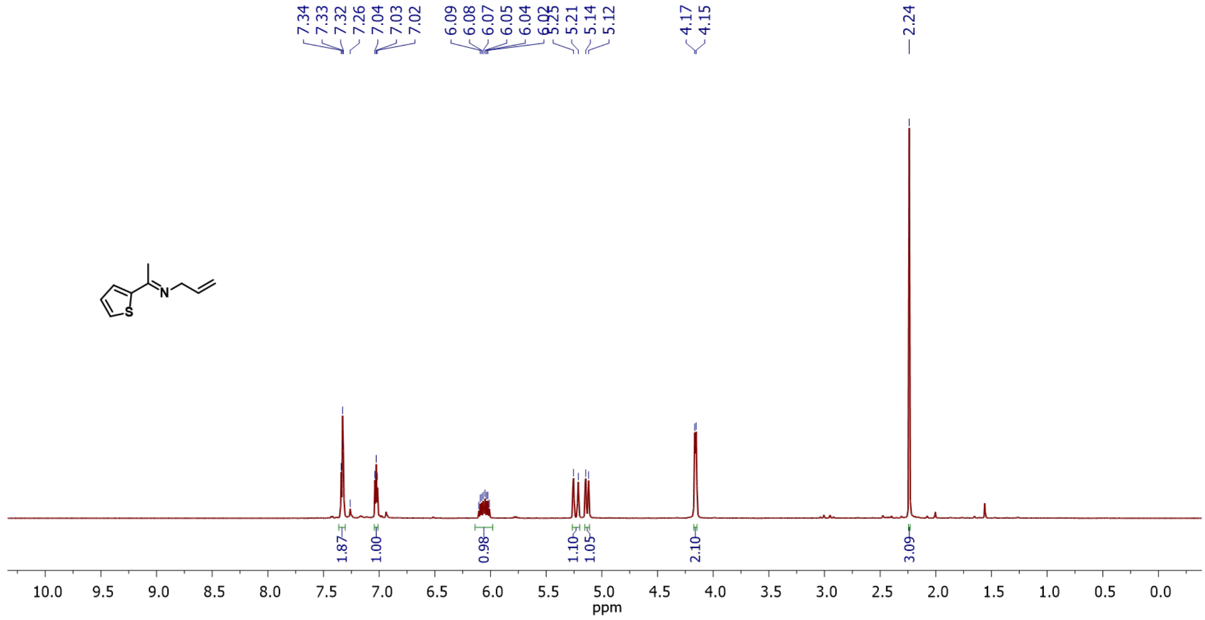

Figure S22. ^{1}H NMR spectrum (400 MHz) of **4h** in $CDCl_3$.

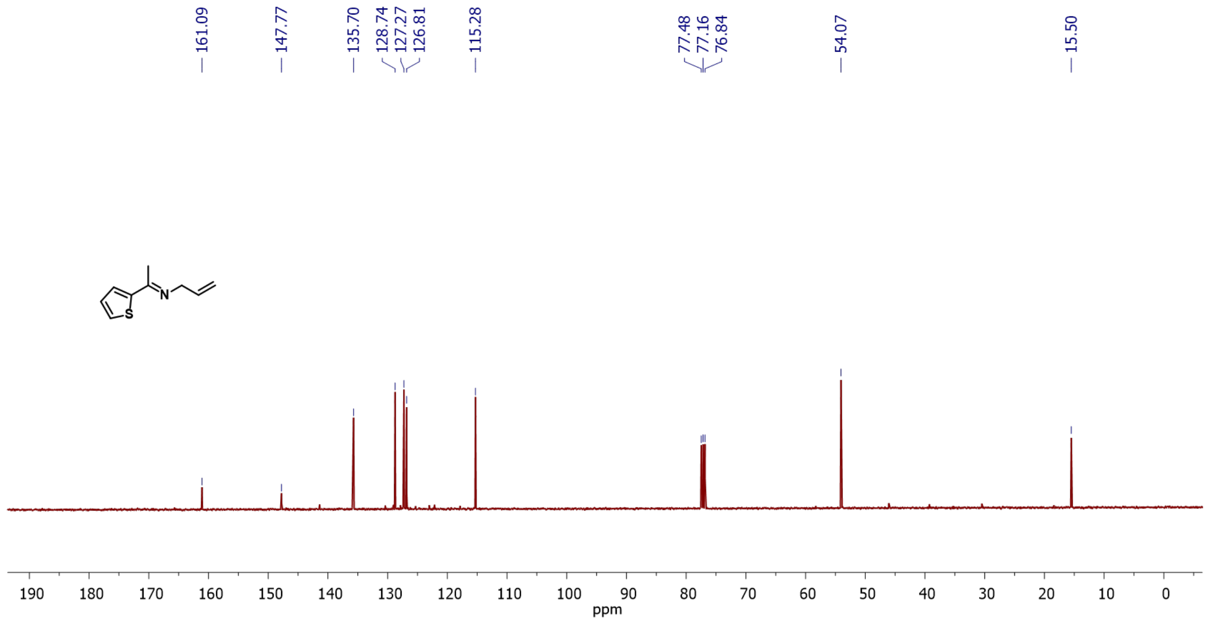

Figure S23. ^{13}C {^{1}H} NMR spectrum (101 MHz) of **4h** in $CDCl_3$.

Compound Spectrum SmartFormula Report

Analysis Info			
Analysis Name	D:\Data\Graham\Dr_5210000001.d	Acquisition Date	06/12/2023 13:06:05
Method	APCI_pos_SolidProbe.m	Operator	Larisa Panz
Sample Name	SRK 4h	Instrument	maXis impact 282001.00128
Comment			

Acquisition Parameter

Source Type	APCI	Ion Polarity	Positive	Set Nebulizer	1.2 Bar
Focus	Active	Set Capillary	3000 V	Set Dry Heater	120 °C
Scan Begin	50 m/z	Set End Plate Offset	-500 V	Set Dry Gas	1.5 l/min
Scan End	2000 m/z	Set Charging Voltage	2000 V	Set Divert Valve	Source
		Set Corona	5000 nA	Set APCI Heater	250 °C

+MS, 0.4-0.5min #26-28

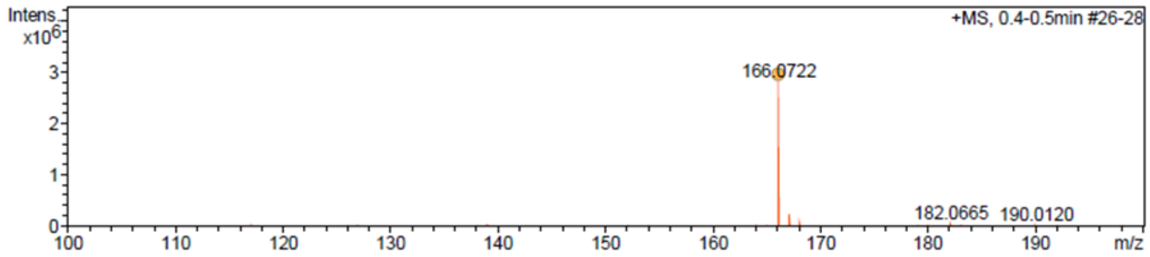

Meas. m/z	#	Ion Formula	m/z	err [ppm]	mSigma	# mSigma	Score	rdb	e⁻ Conf	N-Rule	|err| [mDa]
166.0722	1	C5H12NO3S	166.0532	-114.4	14.7	1	0.00	0.5	even	ok	19.0
	2	C9H12NS	166.0685	-22.5	14.7	2	100.00	4.5	even	ok	3.7

Figure S24. High resolution mass spectrum of **4h**.

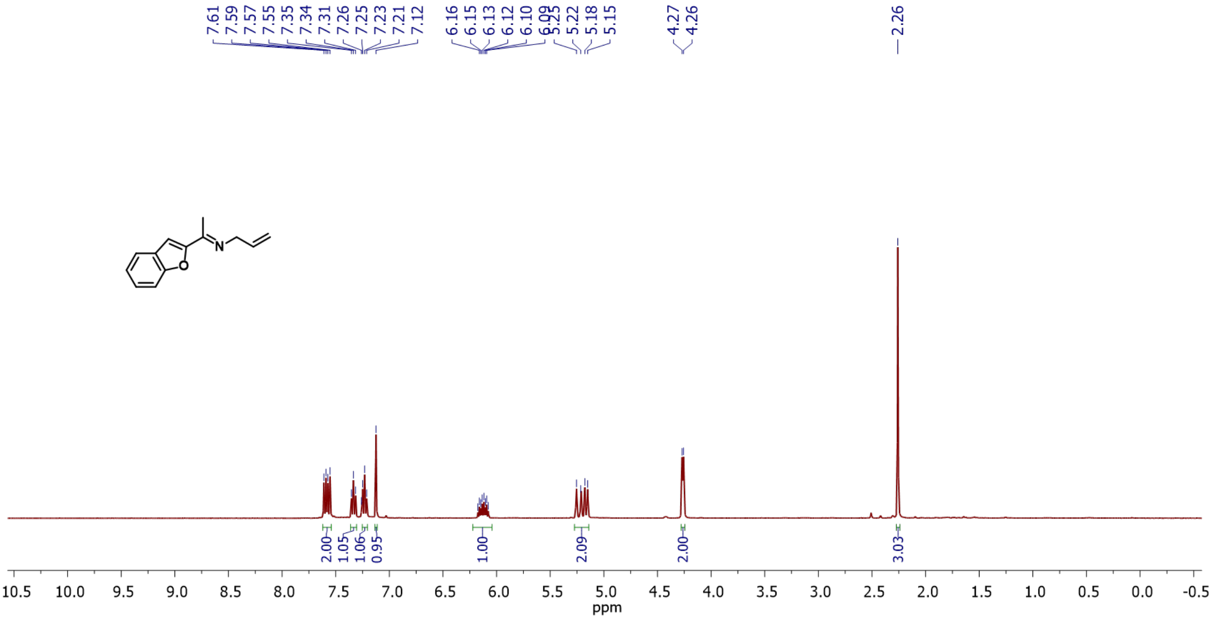

Figure S25. ^{1}H NMR spectrum (400 MHz) of **4j** in $CDCl_3$.

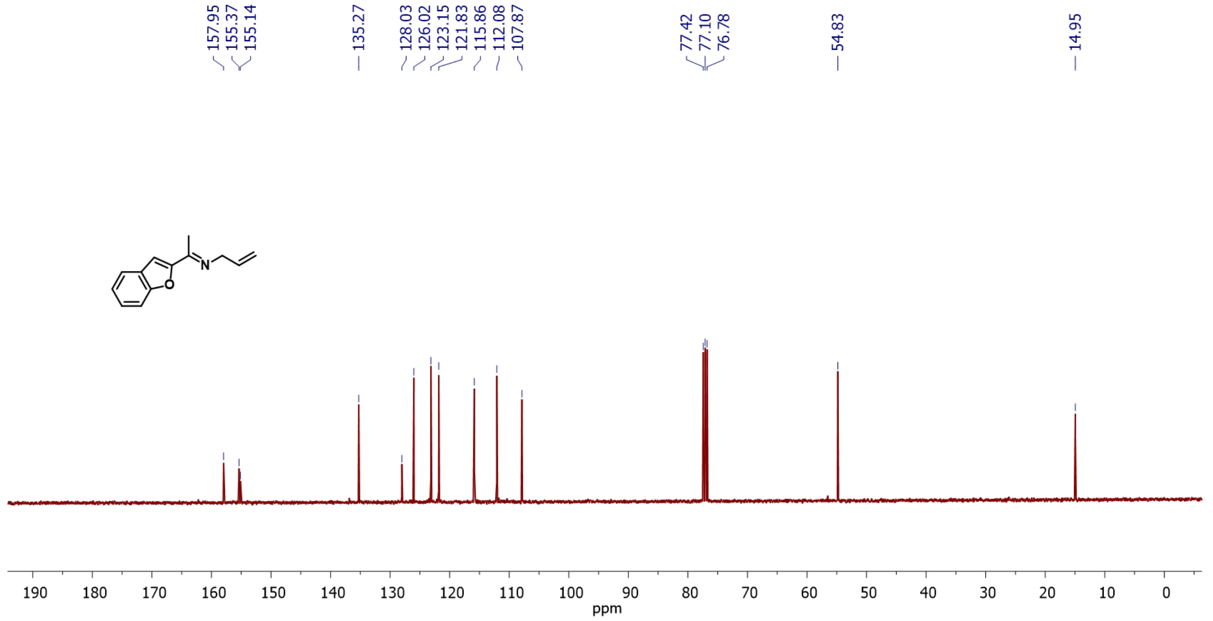

Figure S26. ^{13}C {^{1}H} NMR spectrum (101 MHz) of **4j** in $CDCl_3$.

Compound Spectrum SmartFormula Report

Analysis Info

Analysis Name	D:\Data\Graham\Dr_5216000001.d	Acquisition Date	06/12/2023 13:25:13
Method	APCI_pos_SolidProbe.m	Operator	Larisa Panz
Sample Name	SRK 4j	Instrument	maXis impact 282001.00128
Comment			

Acquisition Parameter

Source Type	APCI	Ion Polarity	Positive	Set Nebulizer	1.2 Bar
Focus	Active	Set Capillary	3000 V	Set Dry Heater	120 °C
Scan Begin	50 m/z	Set End Plate Offset	-500 V	Set Dry Gas	1.5 l/min
Scan End	2000 m/z	Set Charging Voltage	2000 V	Set Divert Valve	Source
		Set Corona	5000 nA	Set APCI Heater	250 °C

+MS, 0.5-0.5min #30-32

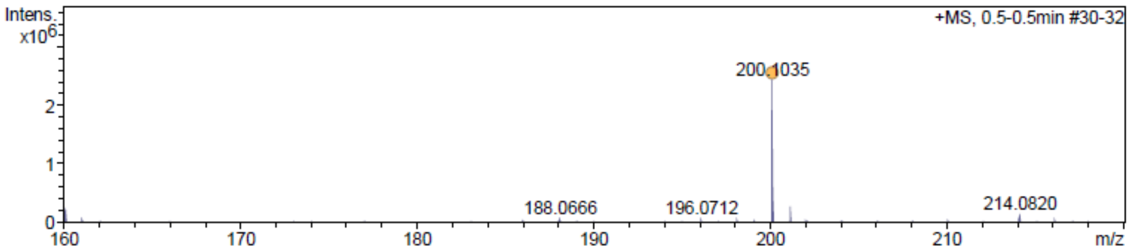

Meas. m/z	#	Ion Formula	m/z	err [ppm]	mSigma	# mSigma	Score	rdb	e⁻ Conf	N-Rule	|err| [mDa]
200.1035	1	C13H14NO	200.1070	17.3	18.7	1	100.00	7.5	even	ok	3.5

Figure S27. High resolution mass spectrum of **4j.**

NMR of Isomerized N-substituted allylamines

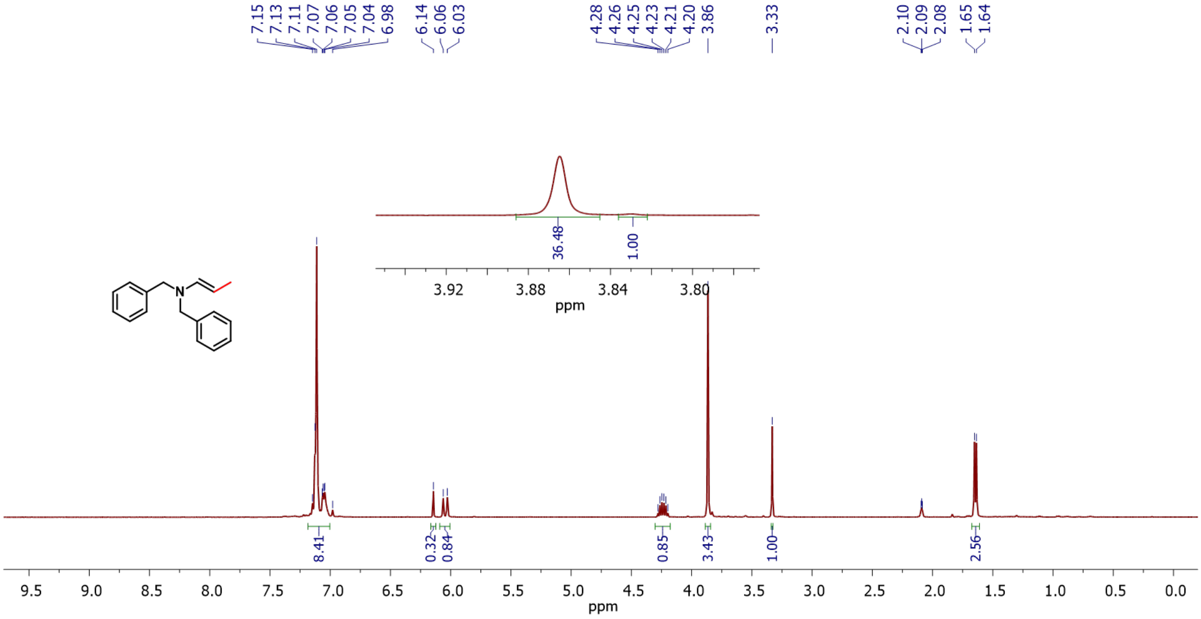

Figure S28. ^{1}H NMR spectrum (400 MHz) of **5a** in toluene-d$_8$.

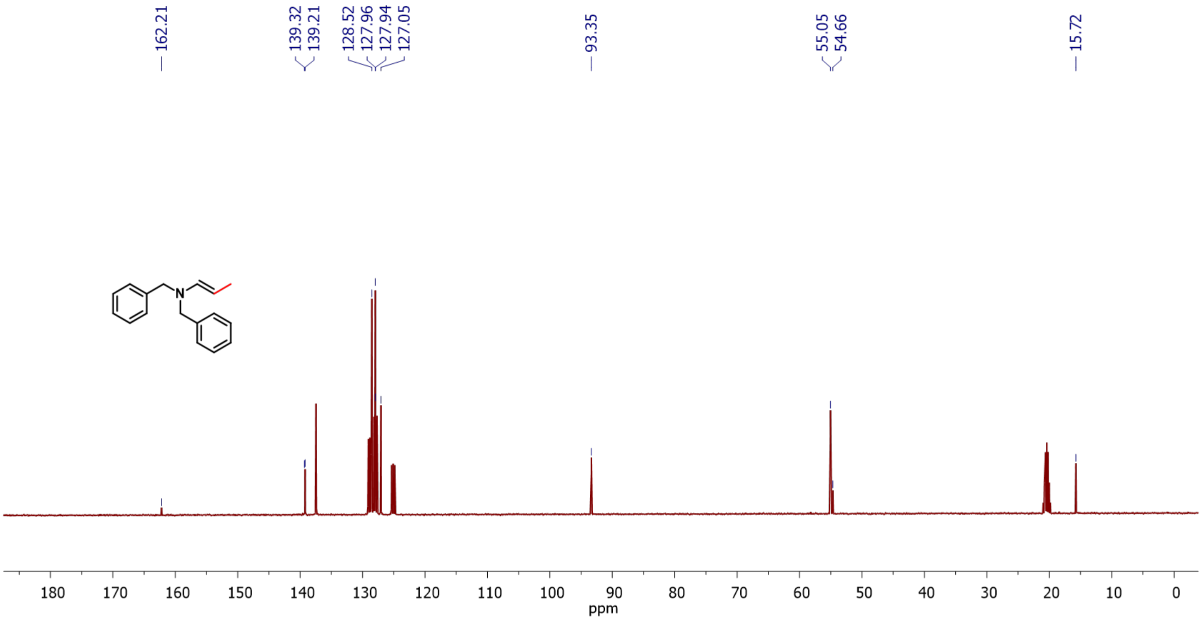

Figure S29. ^{13}C {^{1}H} NMR spectrum (101 MHz) of **5a** in toluene-d$_8$.

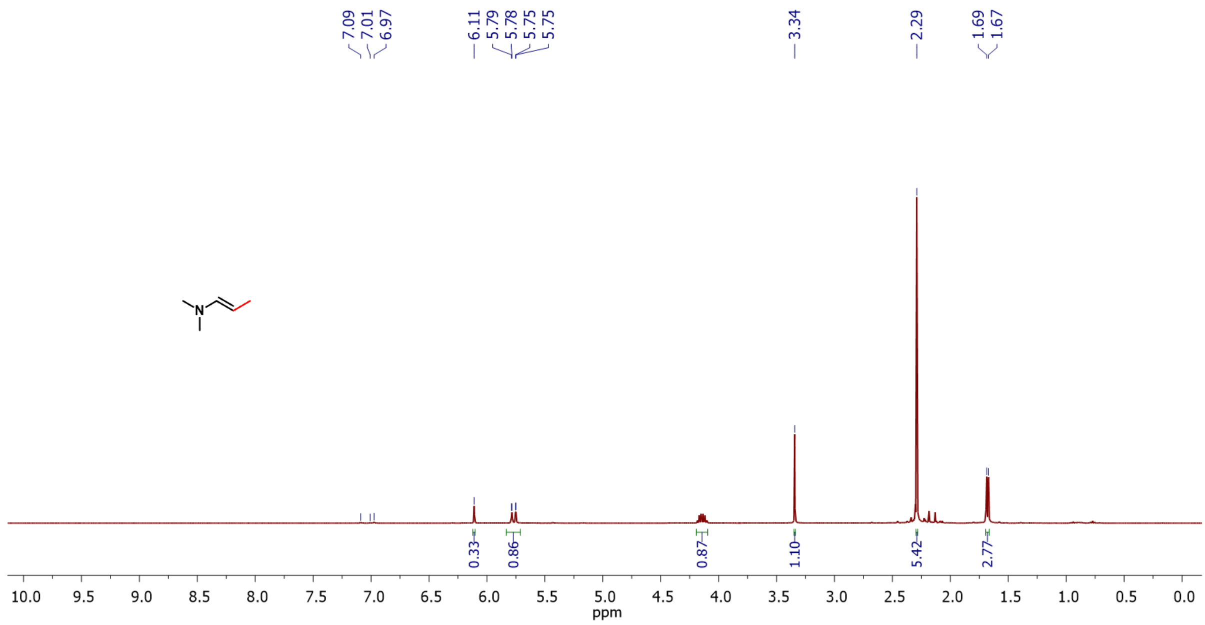

Figure S30. ^{1}H NMR spectrum (400 MHz) of **5b** in toluene-d8.

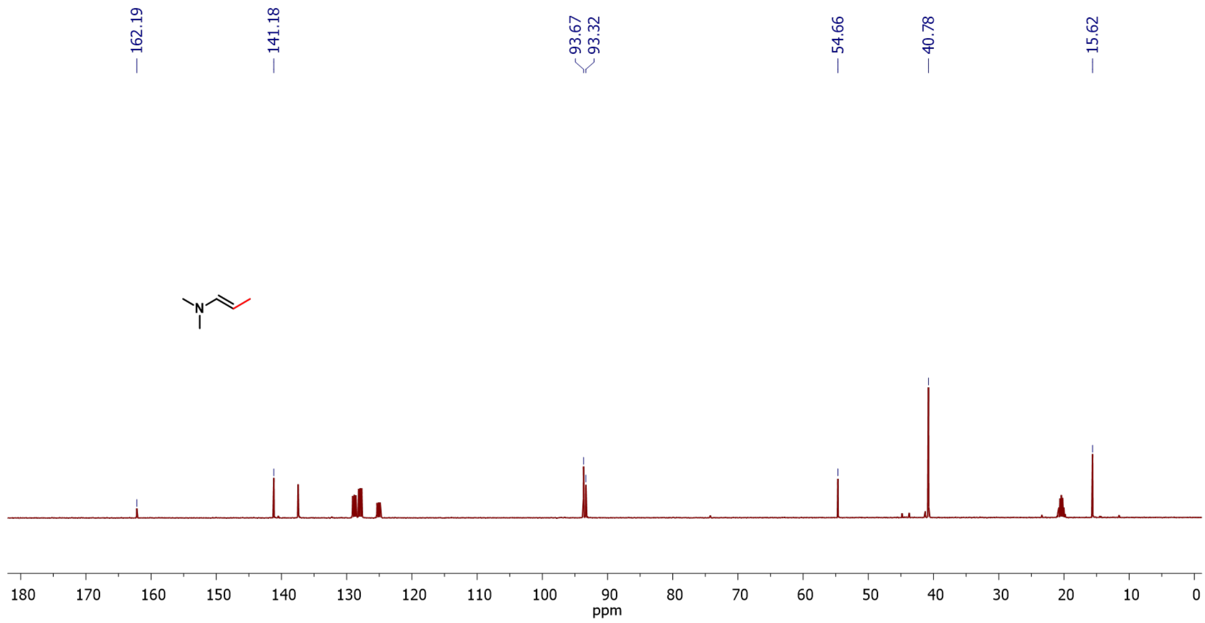

Figure S31. ^{13}C {^{1}H} NMR spectrum (101 MHz) of **5b** in toluene-d8.

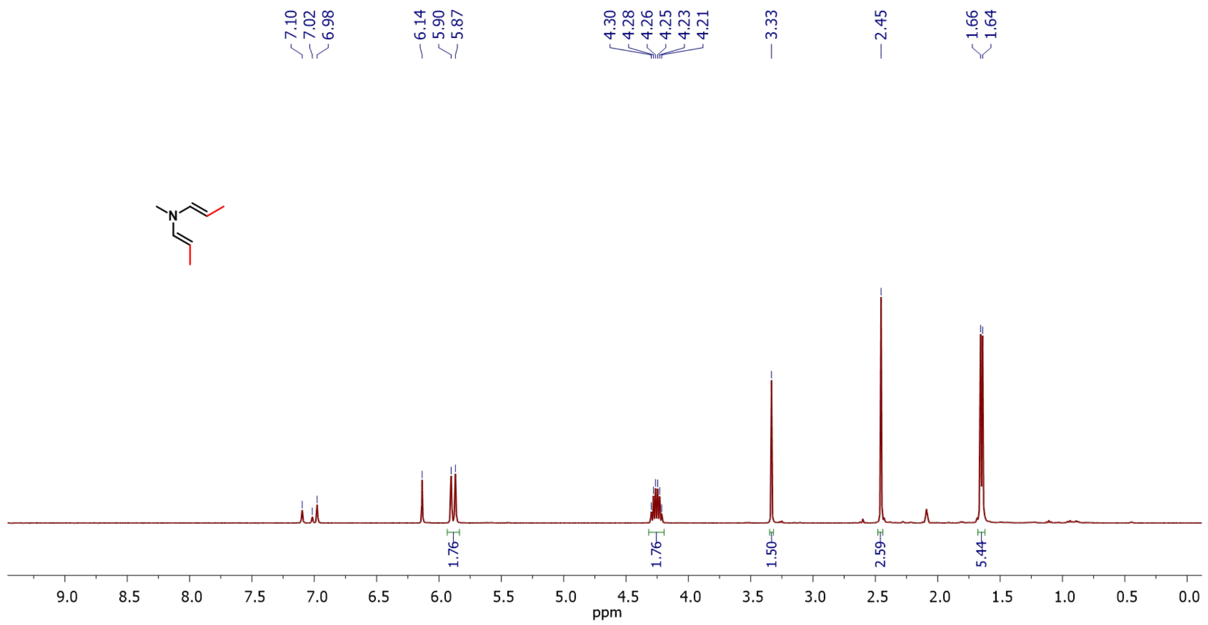

Figure S32. ^{1}H NMR spectrum (400 MHz) of **5c** in toluene-d_8.

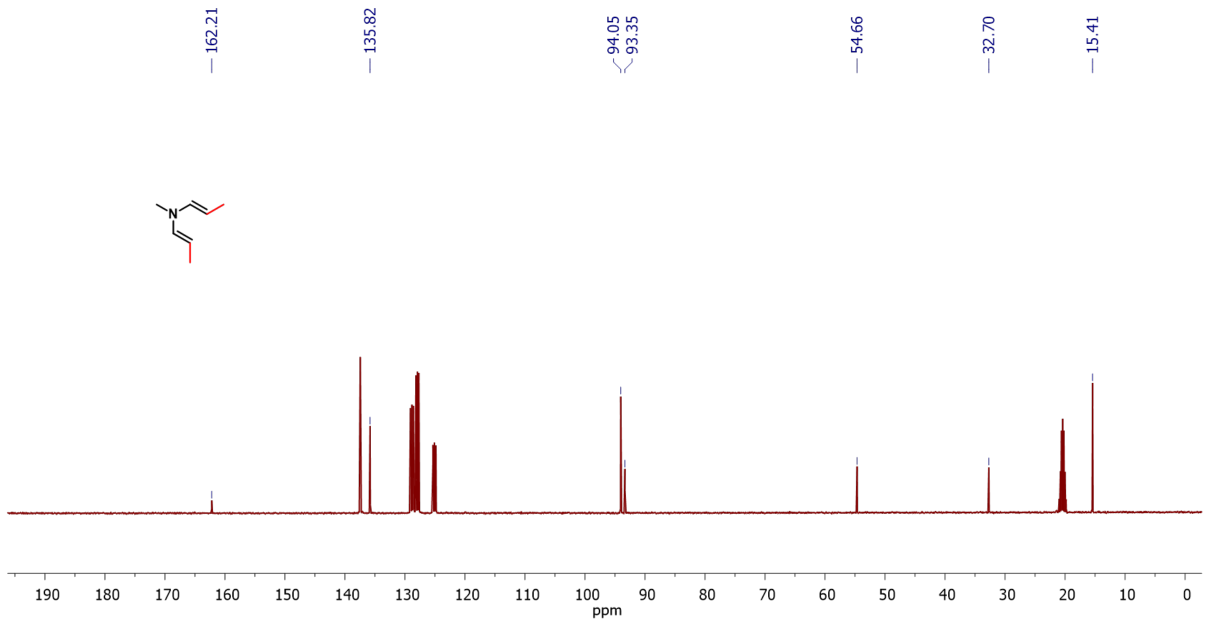

Figure S33. ^{13}C {^{1}H} NMR spectrum (101 MHz) of **5c** in toluene-d_8.

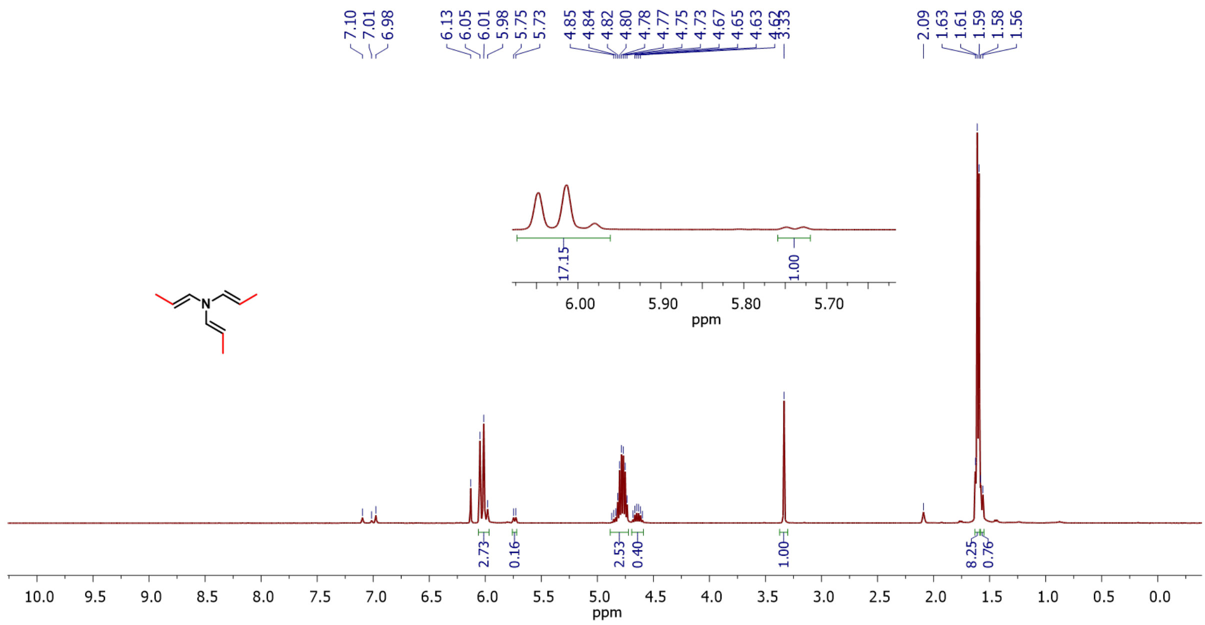

Figure S34. ^{1}H NMR spectrum (400 MHz) of **5d** in toluene-d_8.

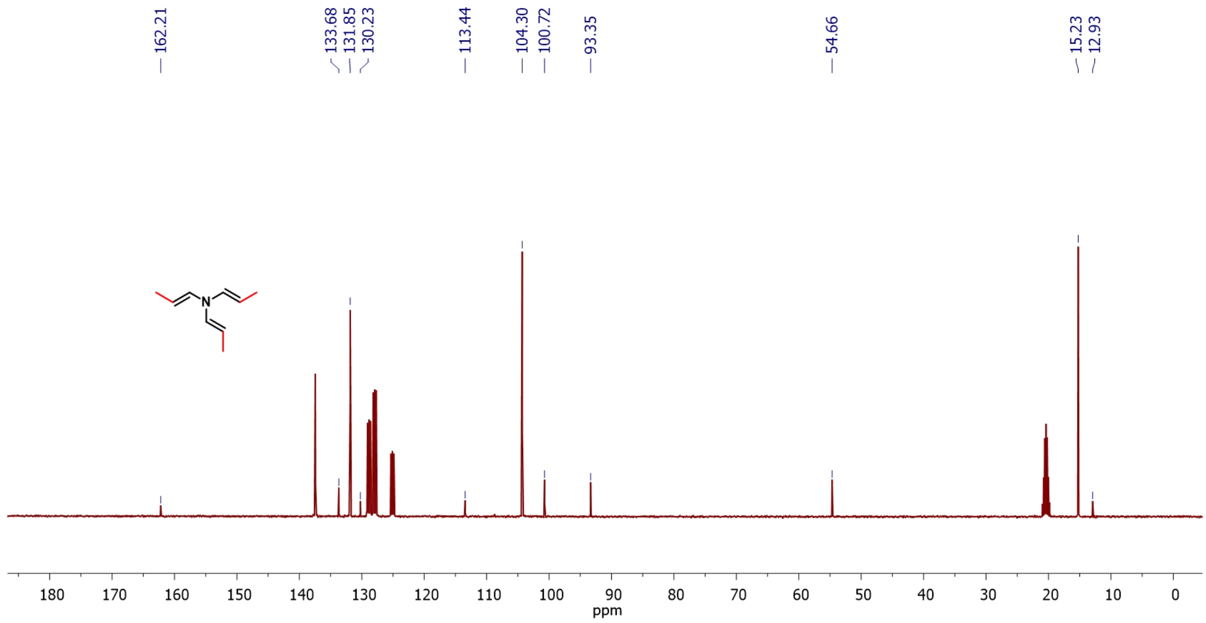

Figure S35. ^{13}C {^{1}H} NMR spectrum (101 MHz) of **5d** in toluene-d_8.

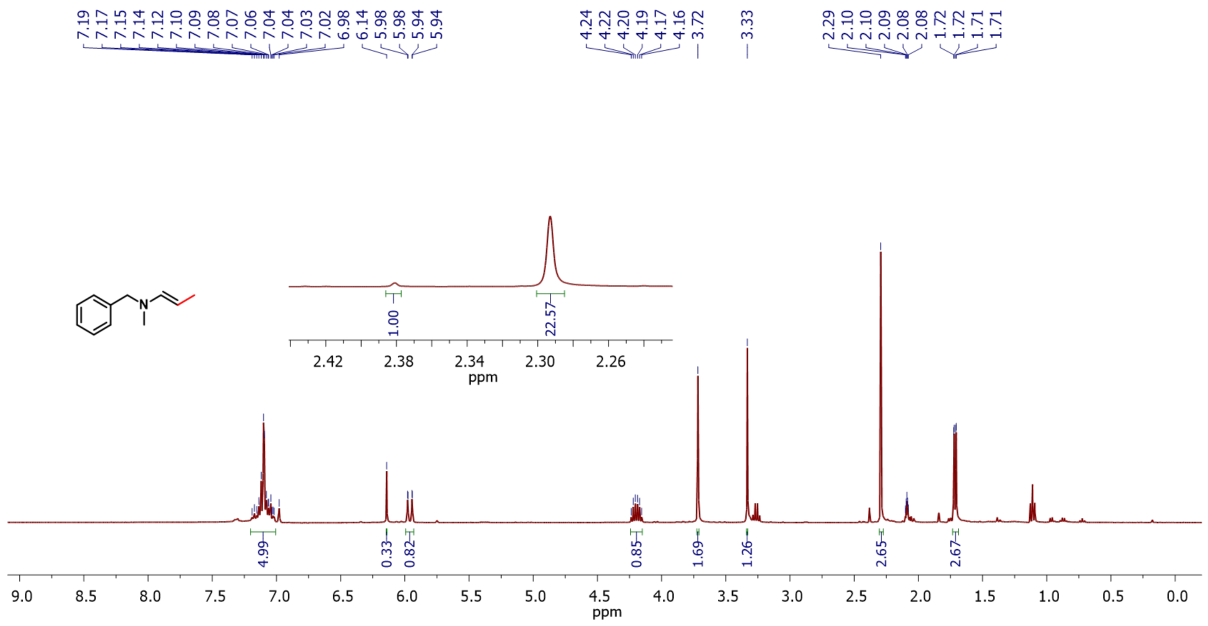

Figure S36. ^{1}H NMR spectrum (400 MHz) of **5e** in toluene-d_8.

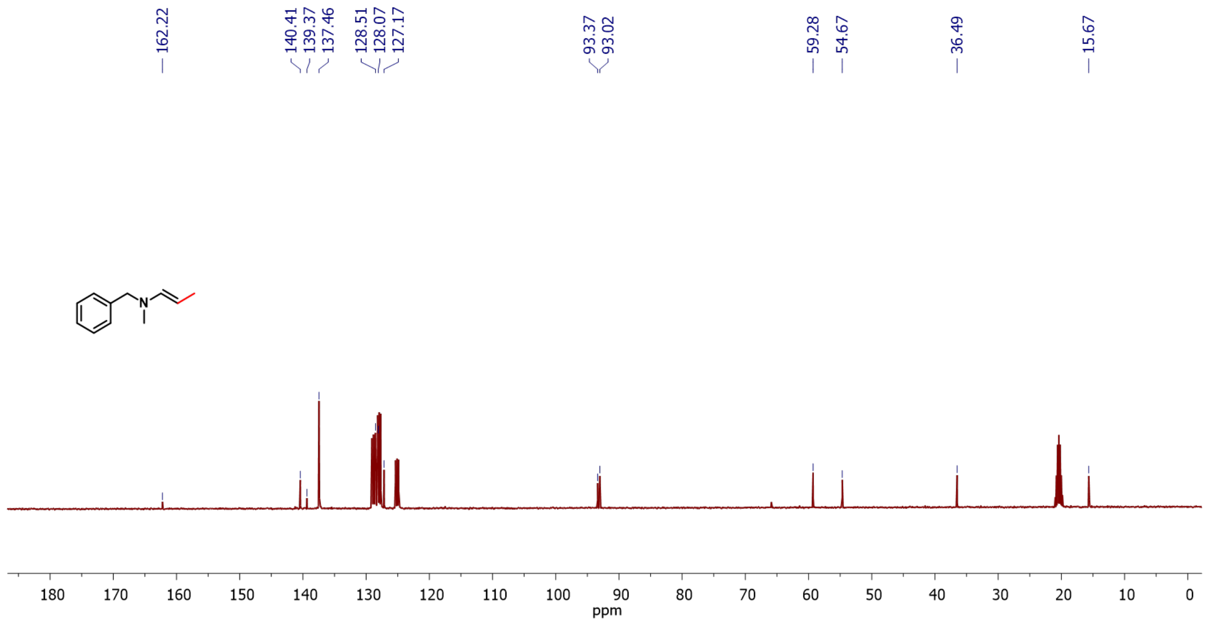

Figure S37. ^{13}C {^{1}H} NMR spectrum (101 MHz) of **5e** in toluene-d_8.

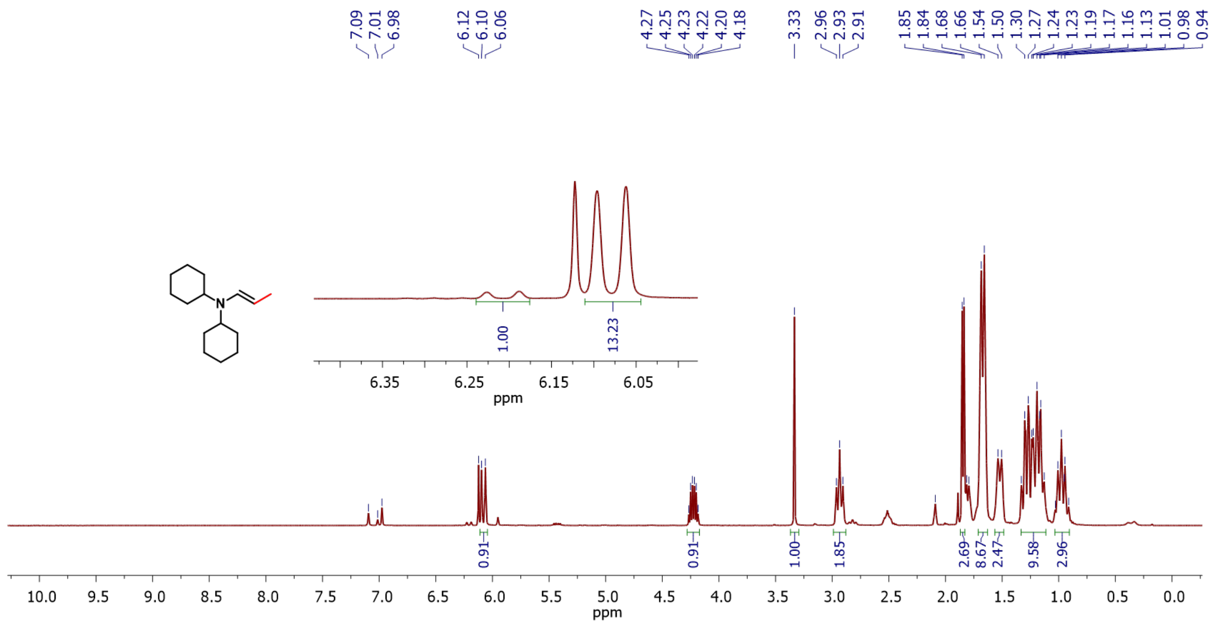

Figure S38. ^{1}H NMR spectrum (400 MHz) of **5f** in toluene-d_8.

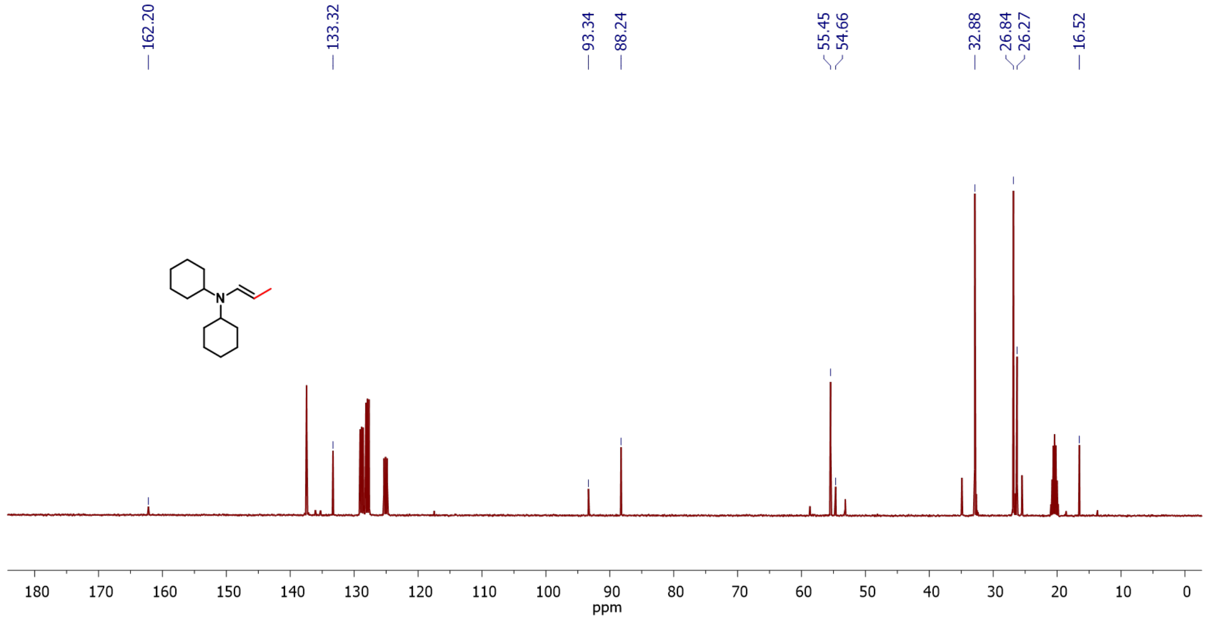

Figure S39. ^{13}C {^{1}H} NMR spectrum (101 MHz) of **5f** in toluene-d_8.

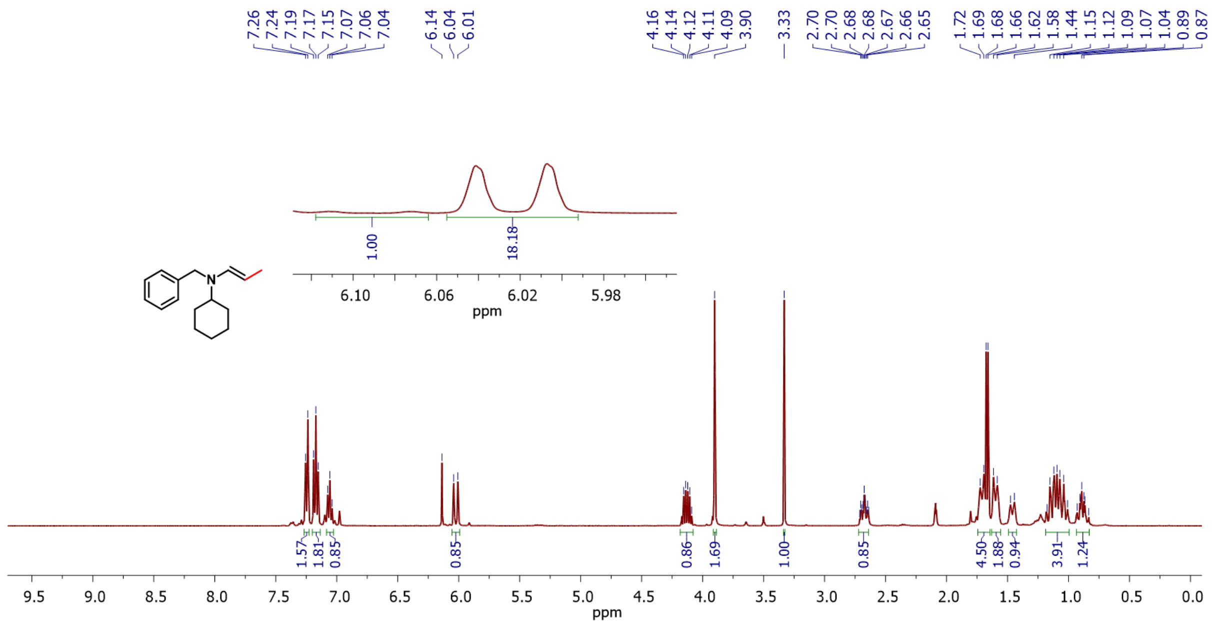

Figure S40. ^{1}H NMR spectrum (400 MHz) of **5g** in toluene-d_8.

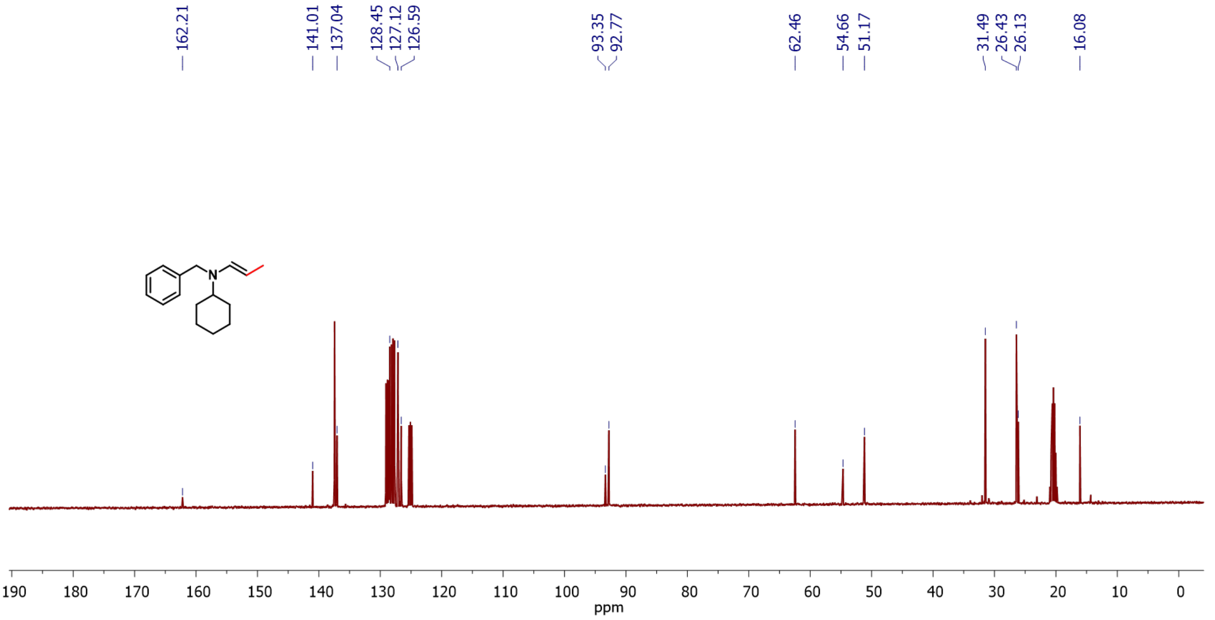

Figure S41. ^{13}C {^{1}H} NMR spectrum (101 MHz) of **5g** in toluene-d_8.

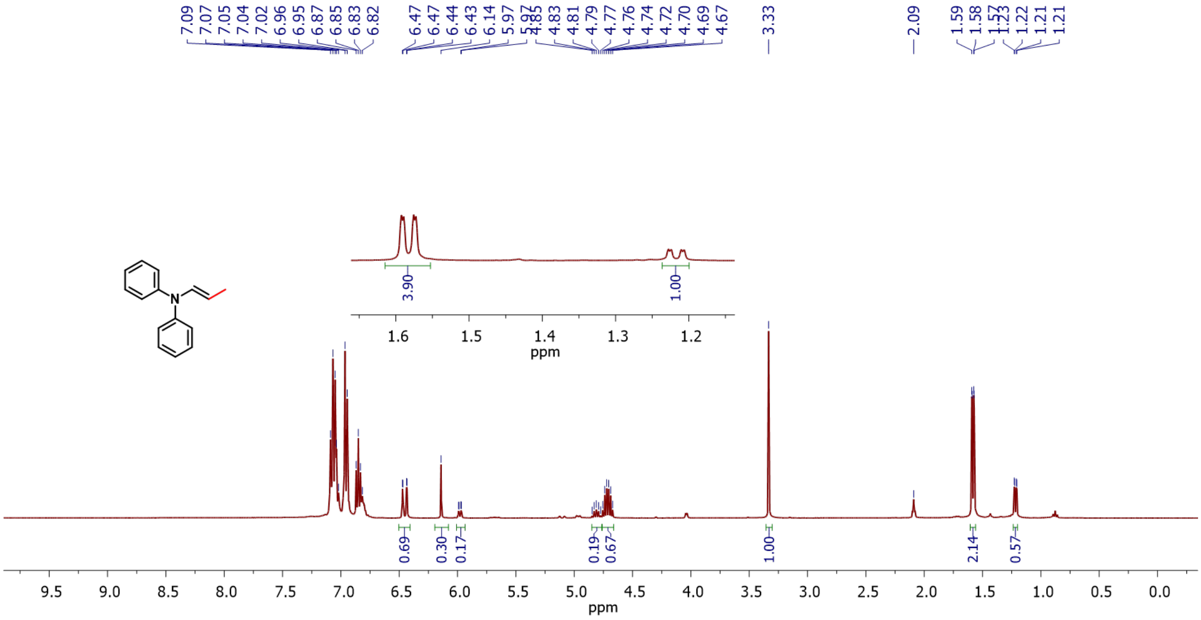

Figure S42. ^{1}H NMR spectrum (400 MHz) of **5h** in toluene-d$_8$.

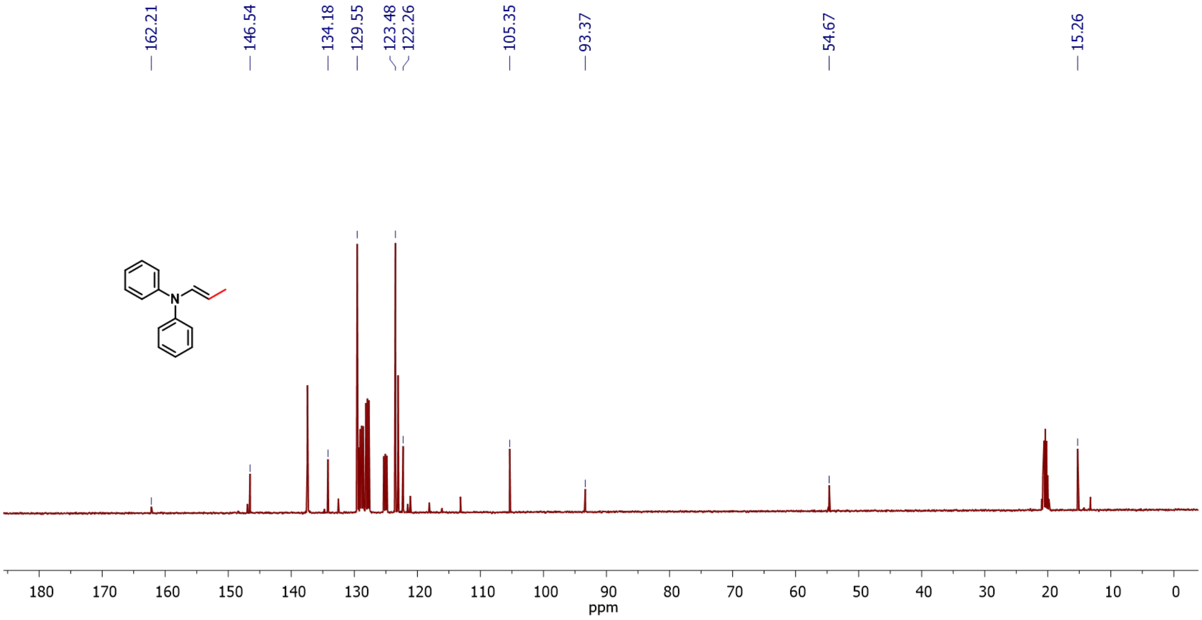

Figure S43. ^{13}C {^{1}H} NMR spectrum (101 MHz) of **5h** in toluene-d$_8$.

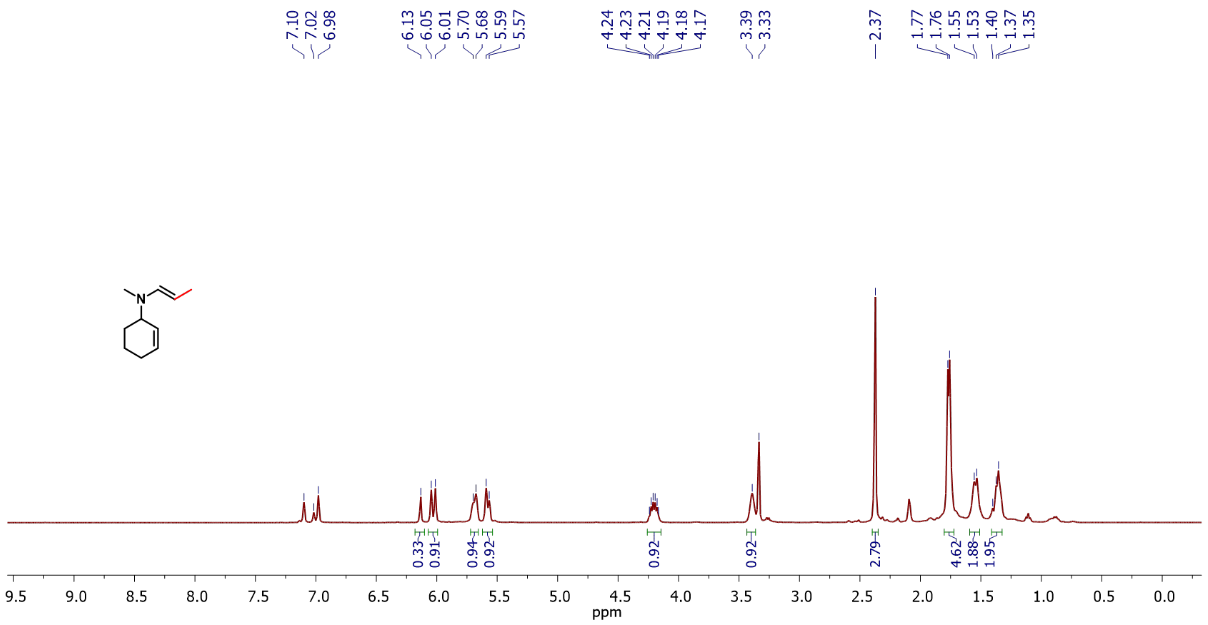

Figure S44. ^{1}H NMR spectrum (400 MHz) of **5i** in toluene-d$_8$.

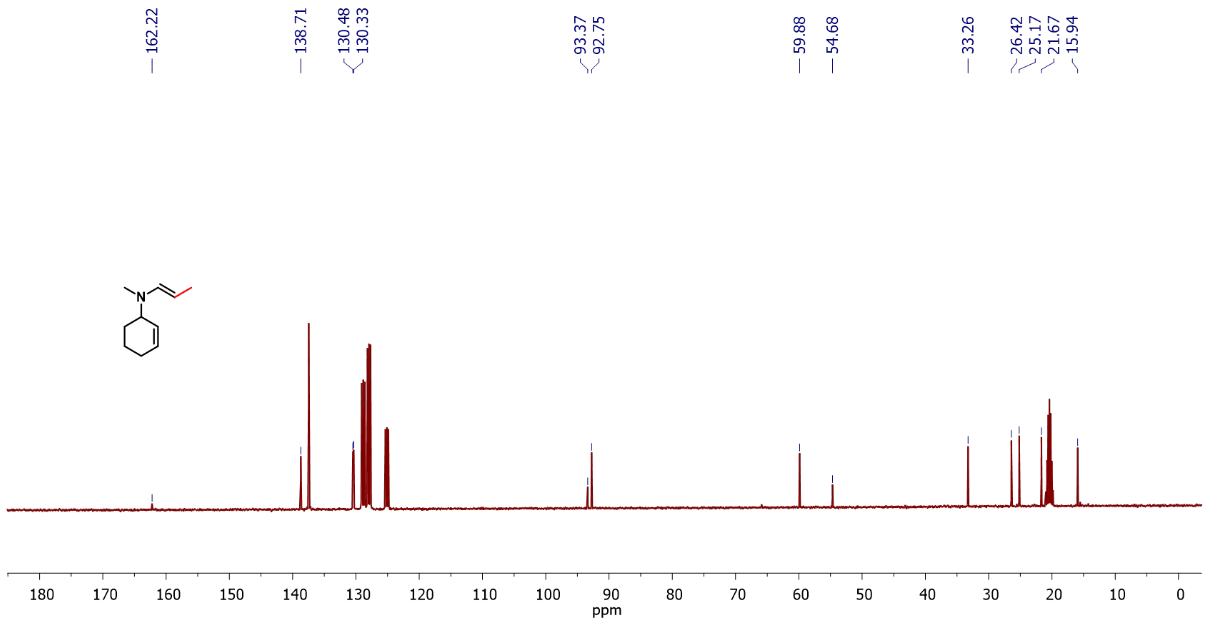

Figure S45. ^{13}C {^{1}H} NMR spectrum (101 MHz) of **5i** in toluene-d$_8$.

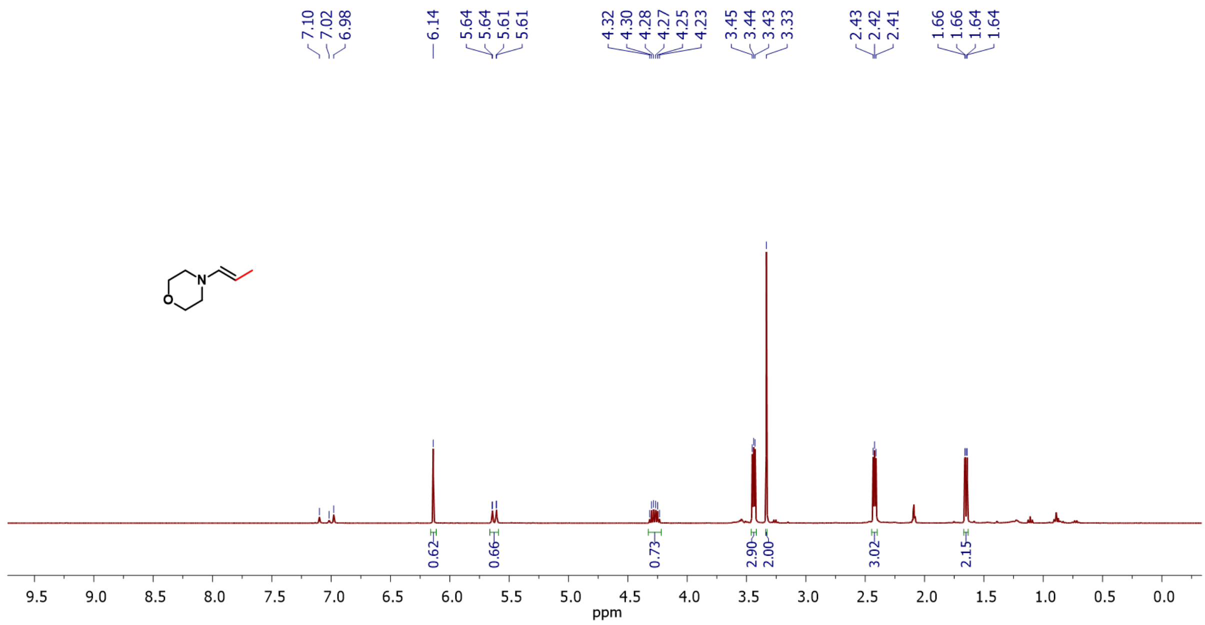

Figure S46. ^{1}H NMR spectrum (400 MHz) of **5j** in toluene-d_8.

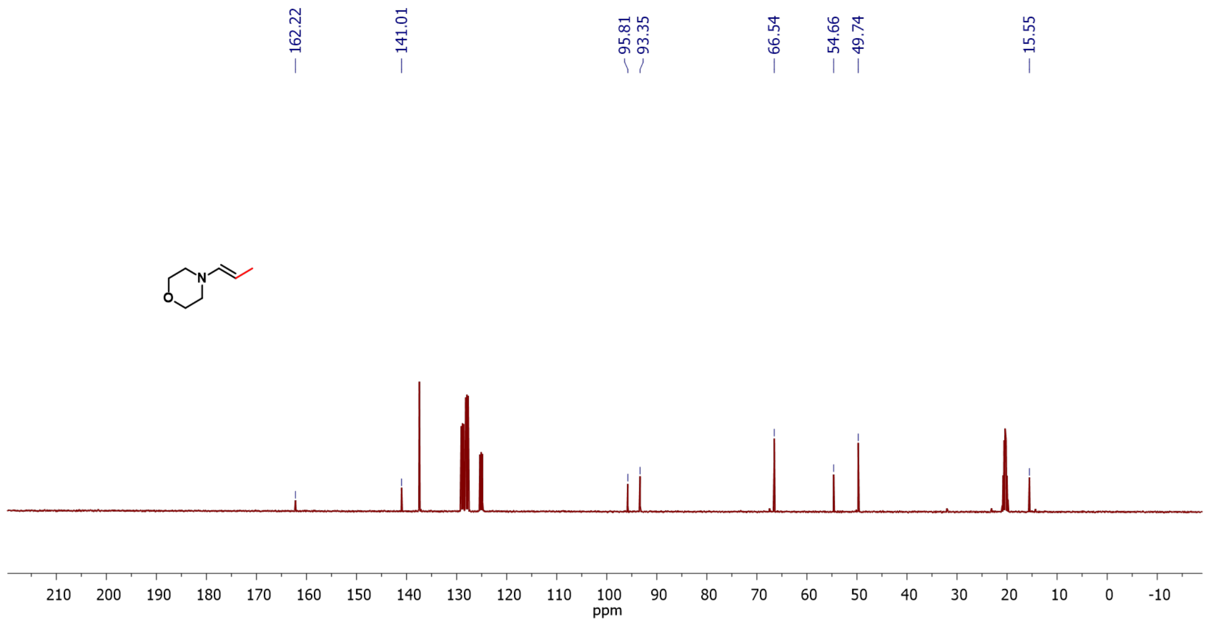

Figure S47. ^{13}C {^{1}H} NMR spectrum (101 MHz) of **5j** in toluene-d_8.

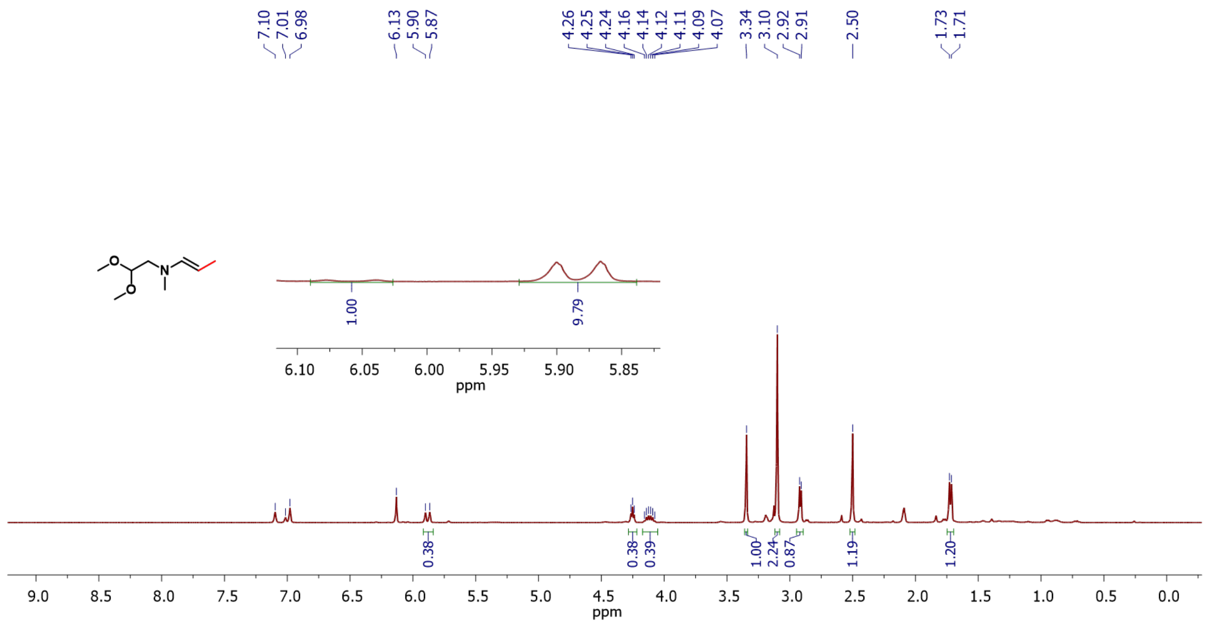

Figure S48. ^{1}H NMR spectrum (400 MHz) of **5k** in toluene-d_8.

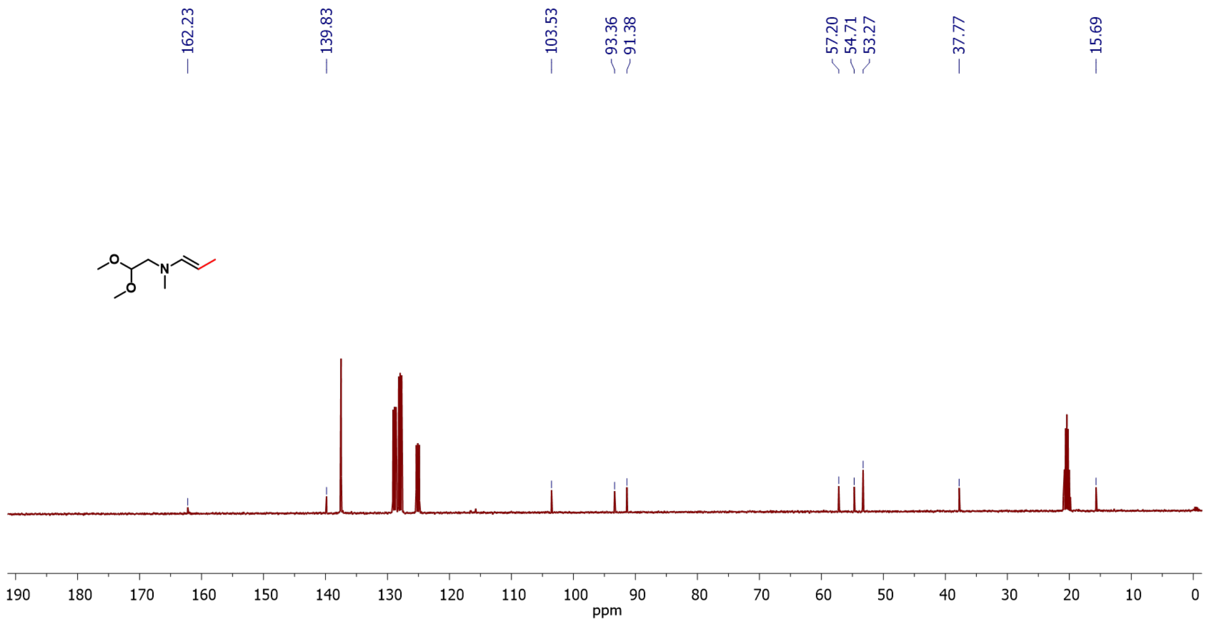

Figure S49. ^{13}C {^{1}H} NMR spectrum (101 MHz) of **5k** in toluene-d_8.

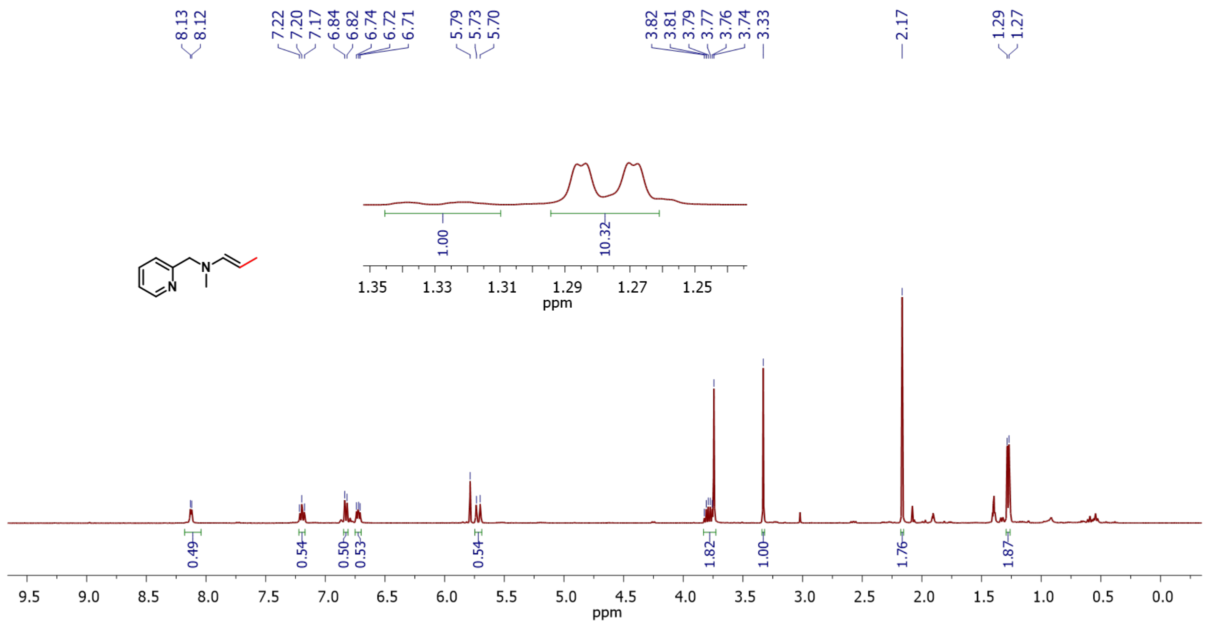

Figure S50. ^{1}H NMR spectrum (400 MHz) of **5l** in toluene-d_8.

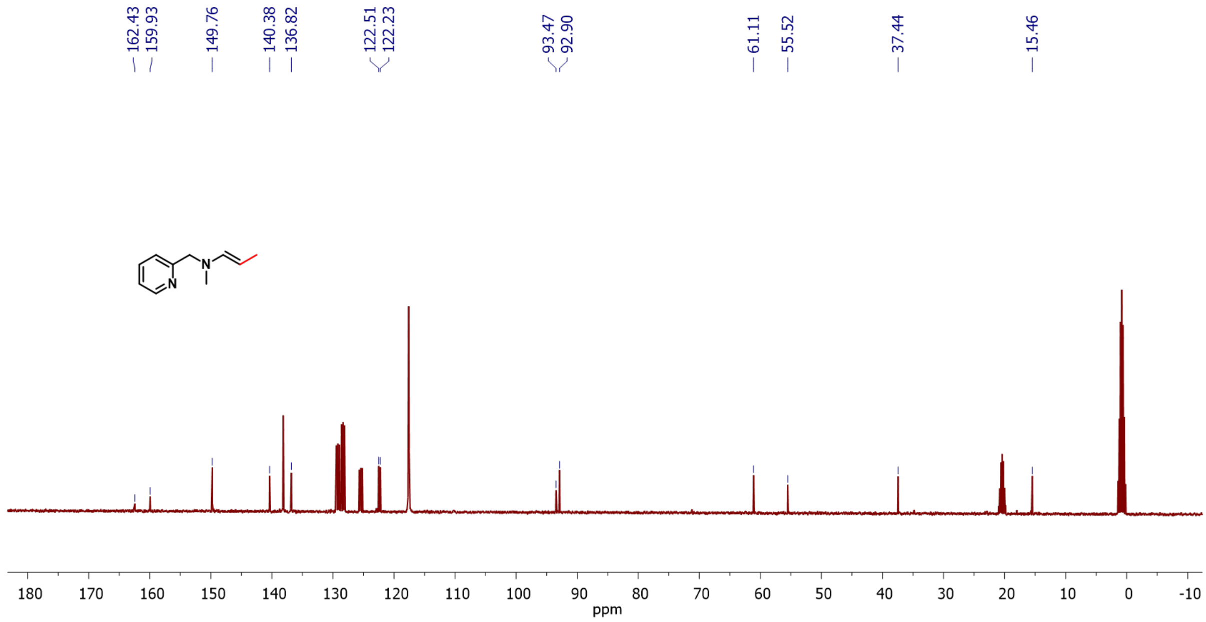

Figure S51. ^{13}C {^{1}H} NMR spectrum (101 MHz) of **5l** in toluene-d_8.

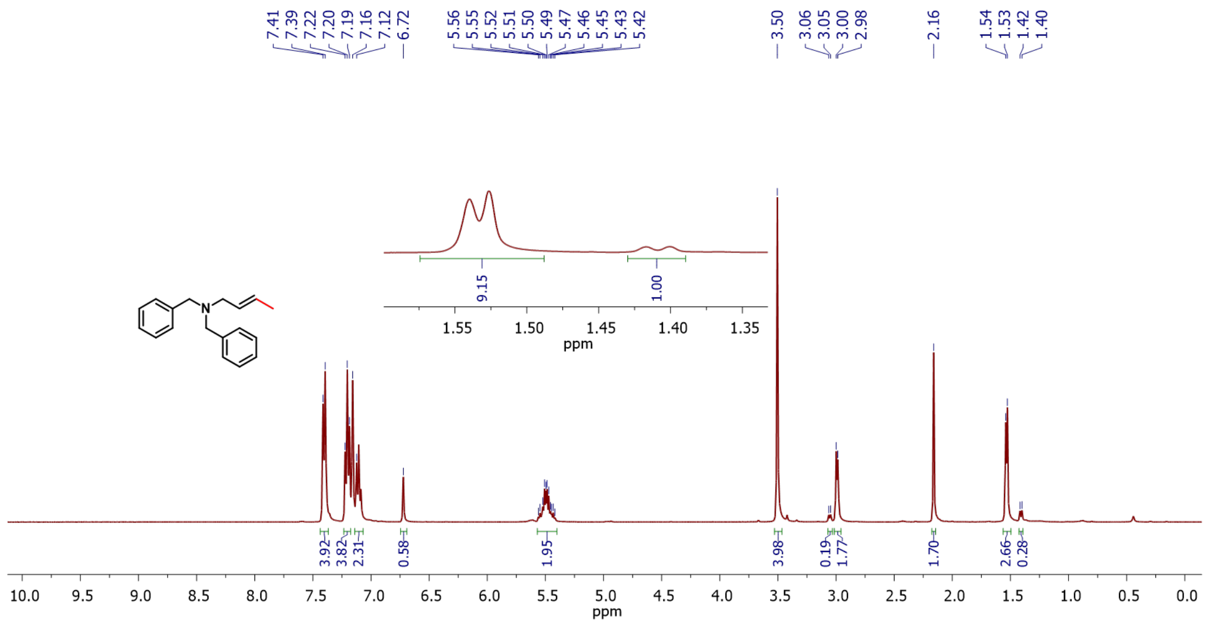

Figure S52. ^{1}H NMR spectrum (400 MHz) of **5m** in benzene-d_6.

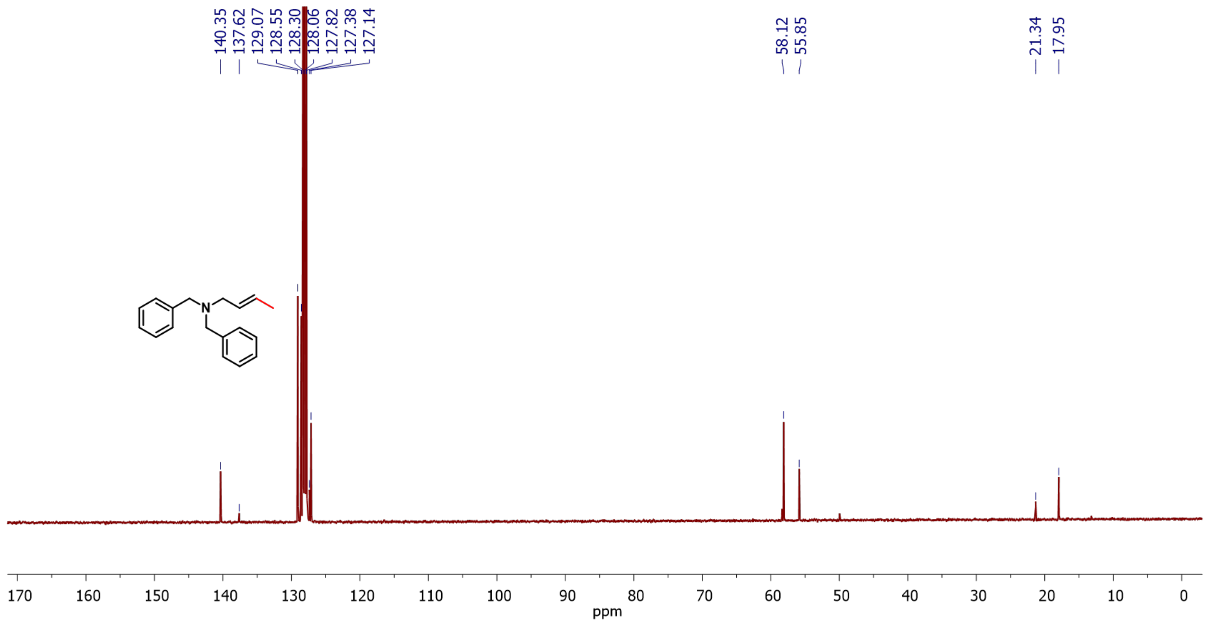

Figure S53. ^{13}C {^{1}H} NMR spectrum (101 MHz) of **5m** in benzene-d_6.

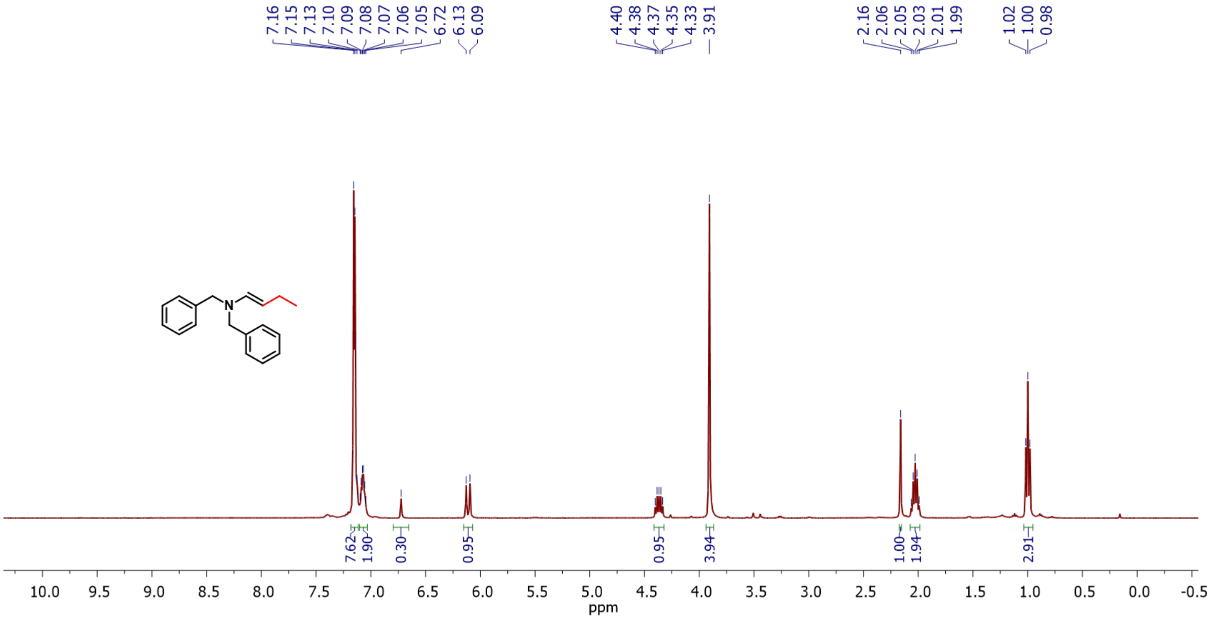

Figure S54. ^{1}H NMR spectrum (400 MHz) of **5n** in benzene-d_6.

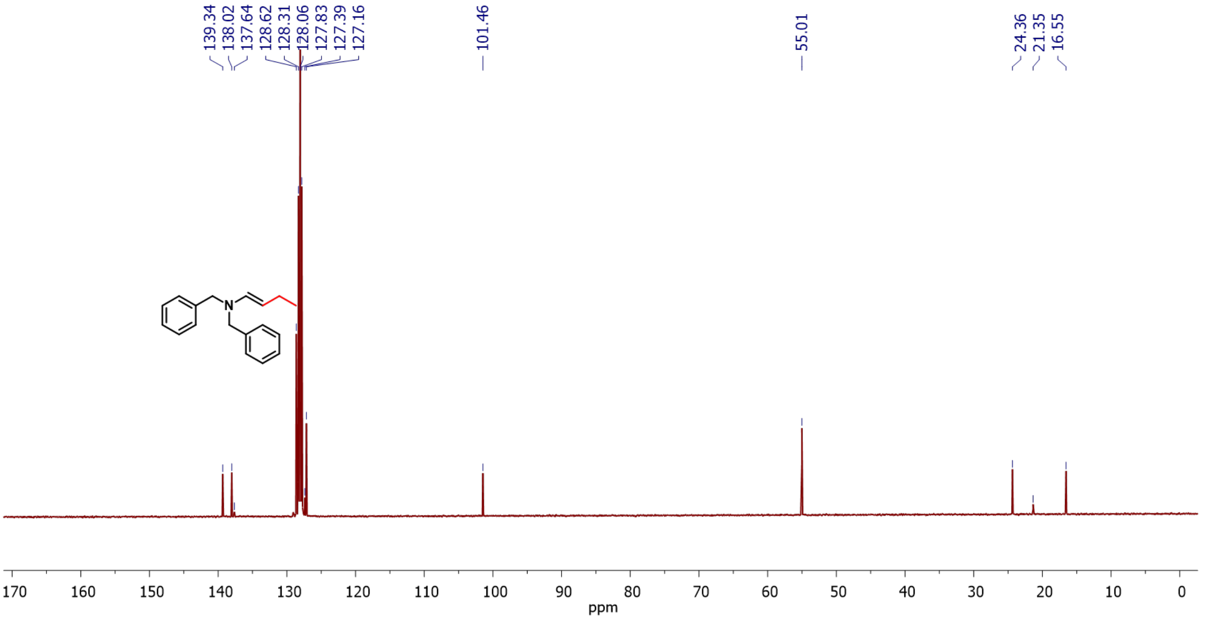

Figure S55. ^{13}C {^{1}H} NMR spectrum (101 MHz) of **5n** in benzene-d_6.

NMR and HRMS of Isomerized N-substituted allylamides.

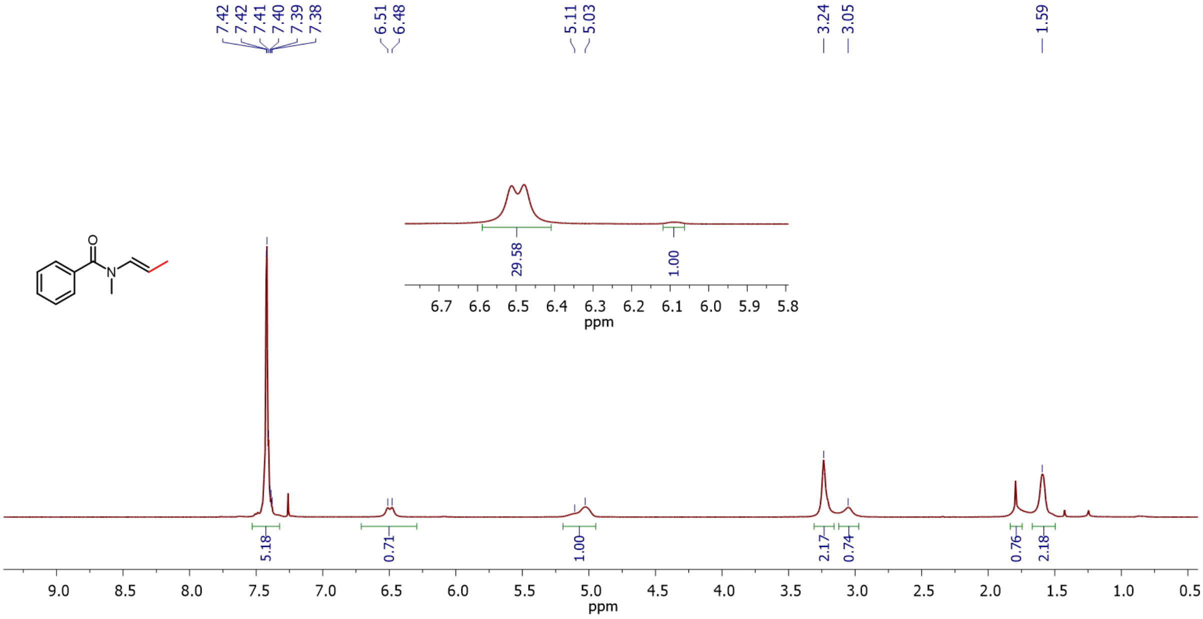

Figure S56. ^{1}H NMR spectrum (400 MHz) of **6a** in $CDCl_3$.

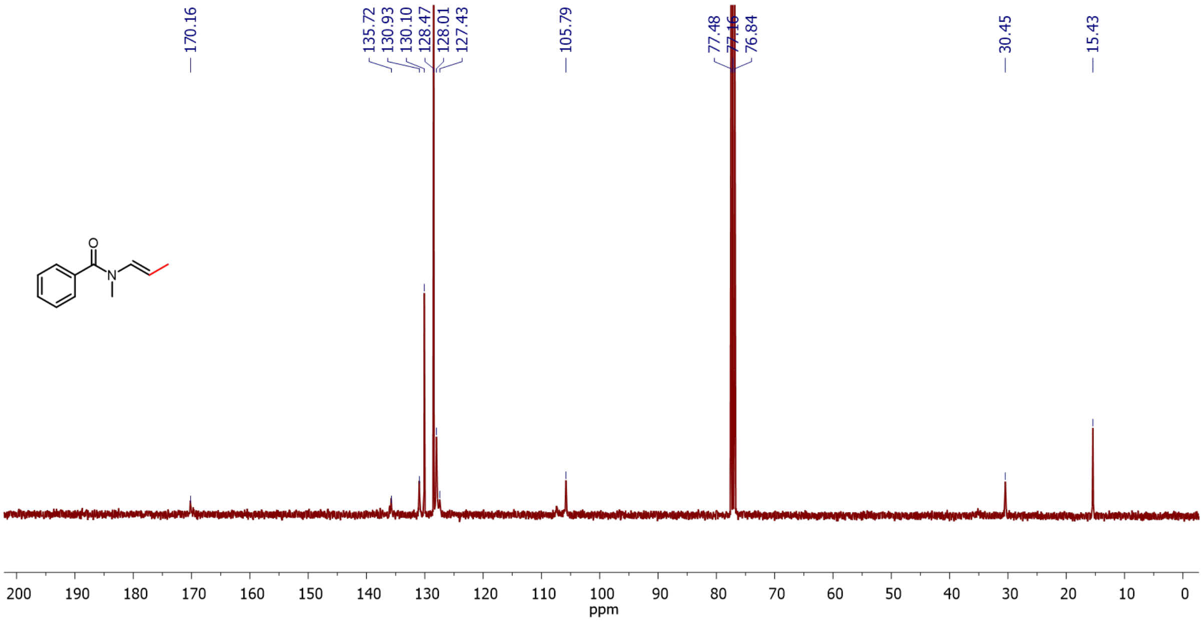

Figure S57. ^{13}C {^{1}H} NMR spectrum (101 MHz) of **6a** in $CDCl_3$.

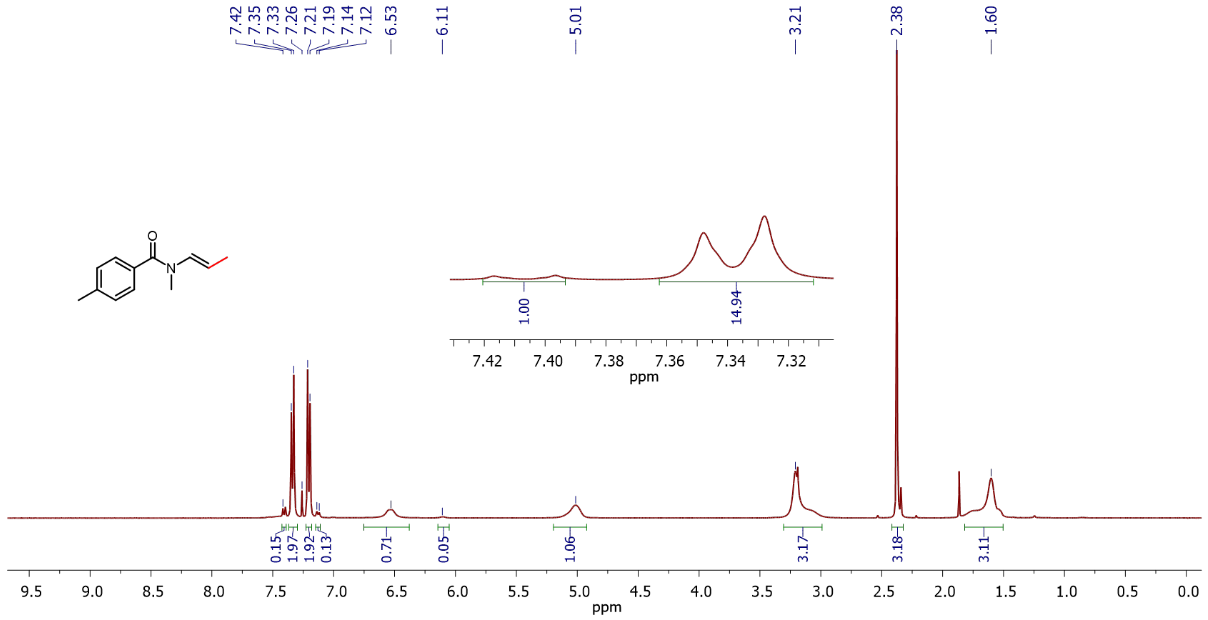

Figure S58. ^{1}H NMR spectrum (400 MHz) of **6b** in $CDCl_3$.

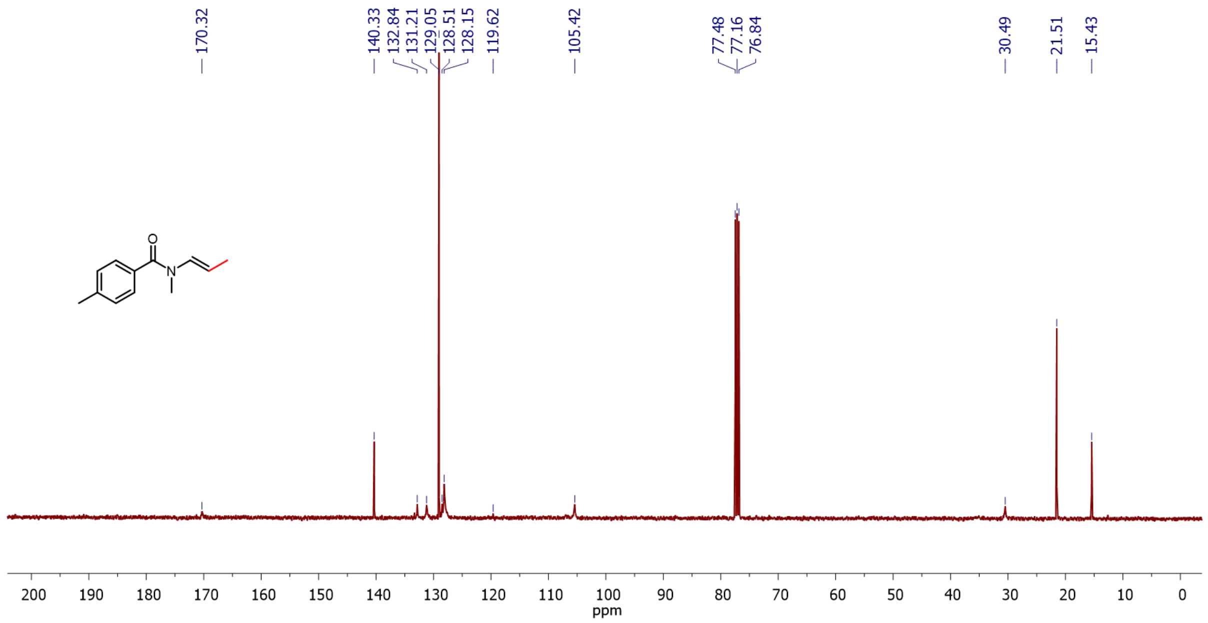

Figure S59. ^{13}C {^{1}H} NMR spectrum (101 MHz) of **6b** in $CDCl_3$.

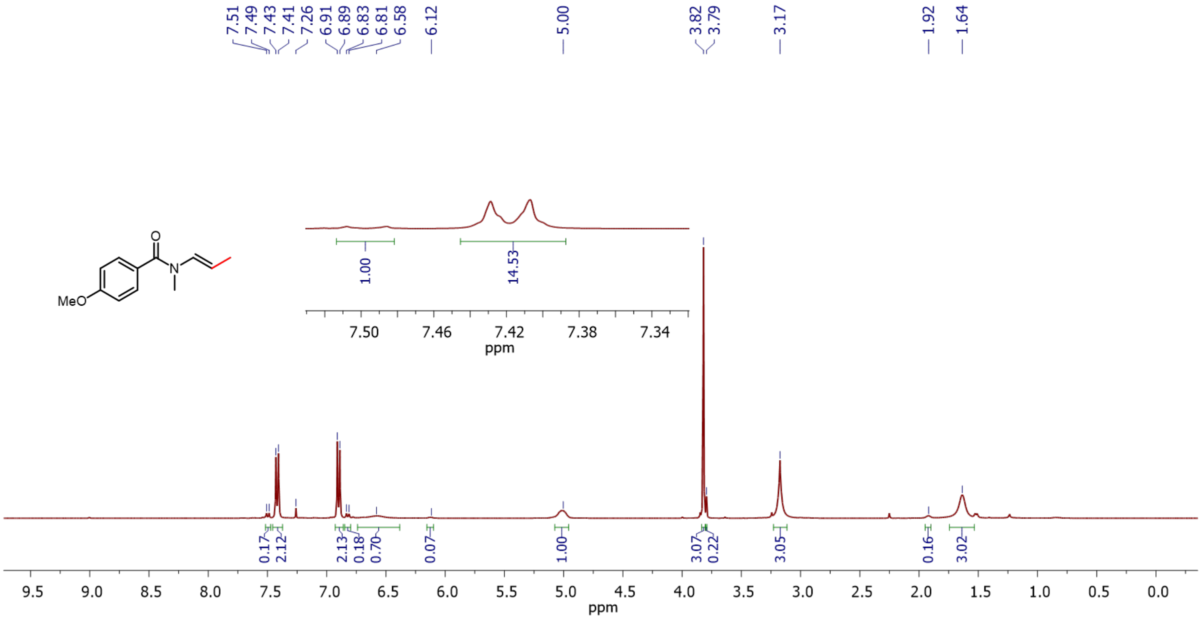

Figure S60. ^{1}H NMR spectrum (400 MHz) of **6c** in $CDCl_3$.

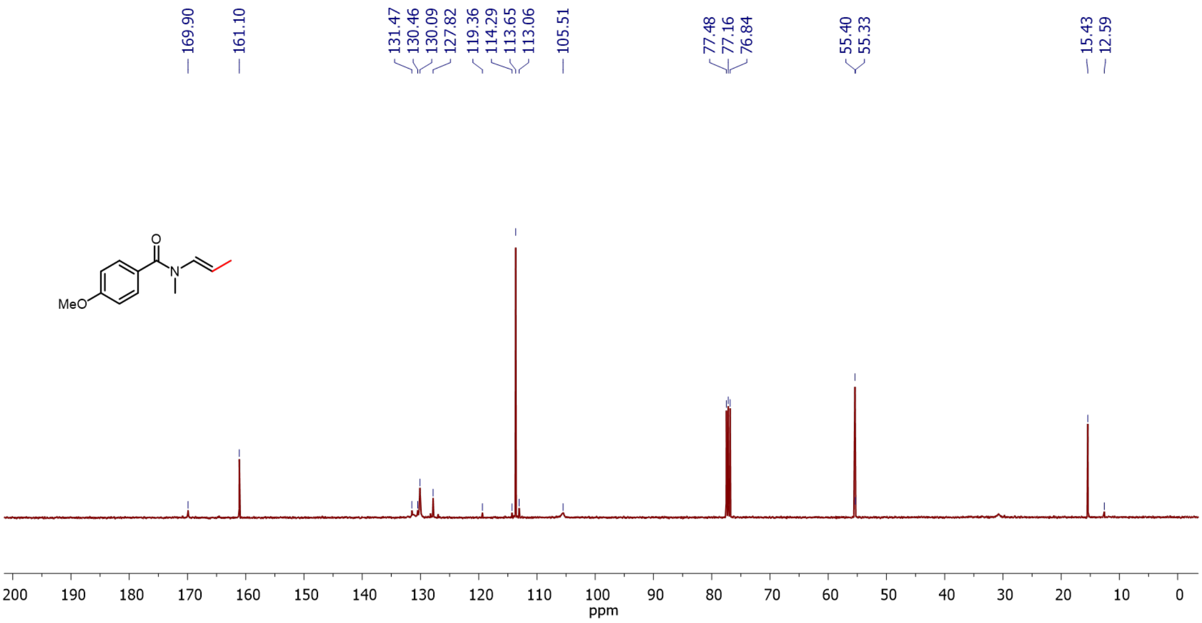

Figure S61. ^{13}C {^{1}H} NMR spectrum (101 MHz) of **6c** in $CDCl_3$.

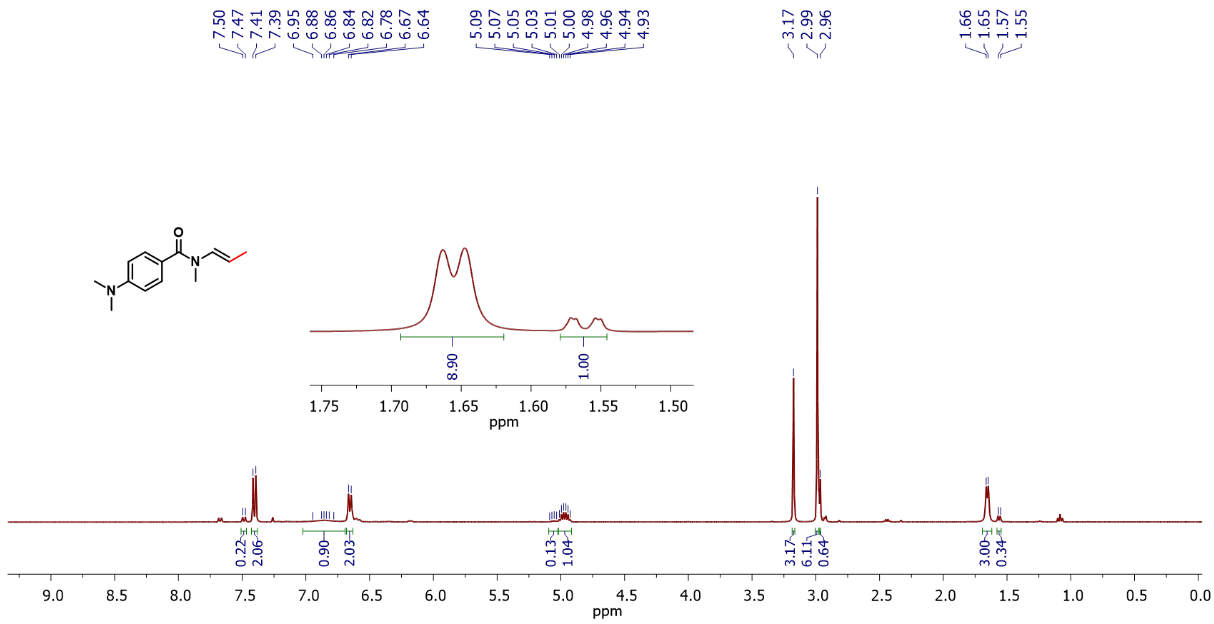

Figure S62. ^{1}H NMR spectrum (400 MHz) of **6d** in $CDCl_3$.

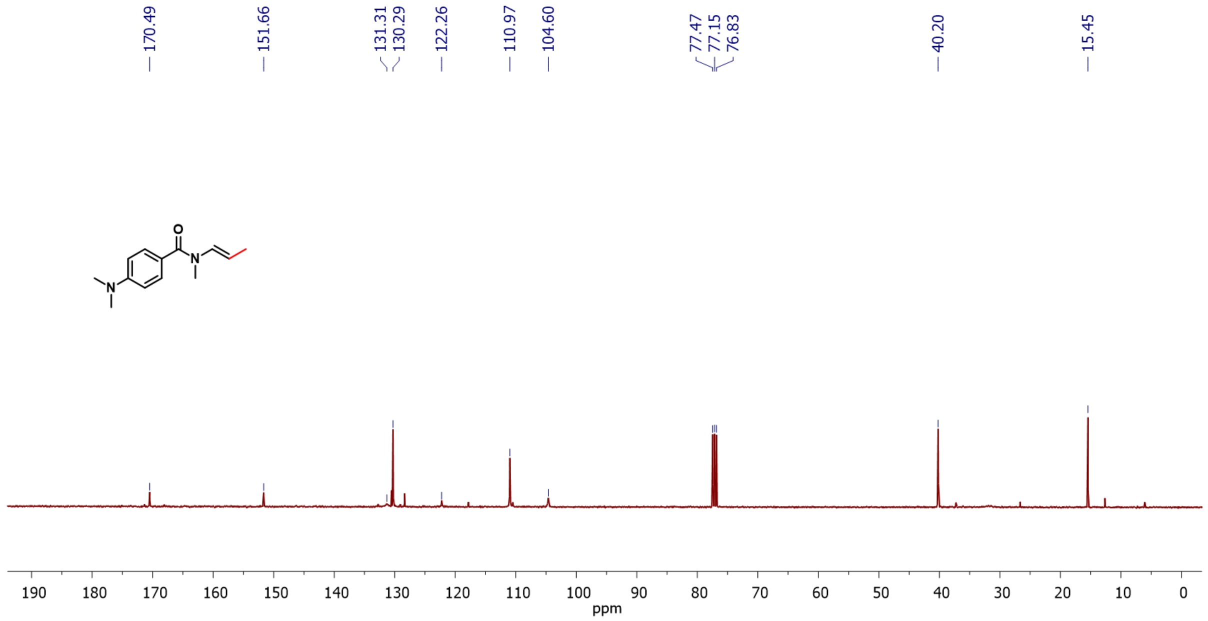

Figure S63. ^{13}C {^{1}H} NMR spectrum (101 MHz) of **6d** in $CDCl_3$.

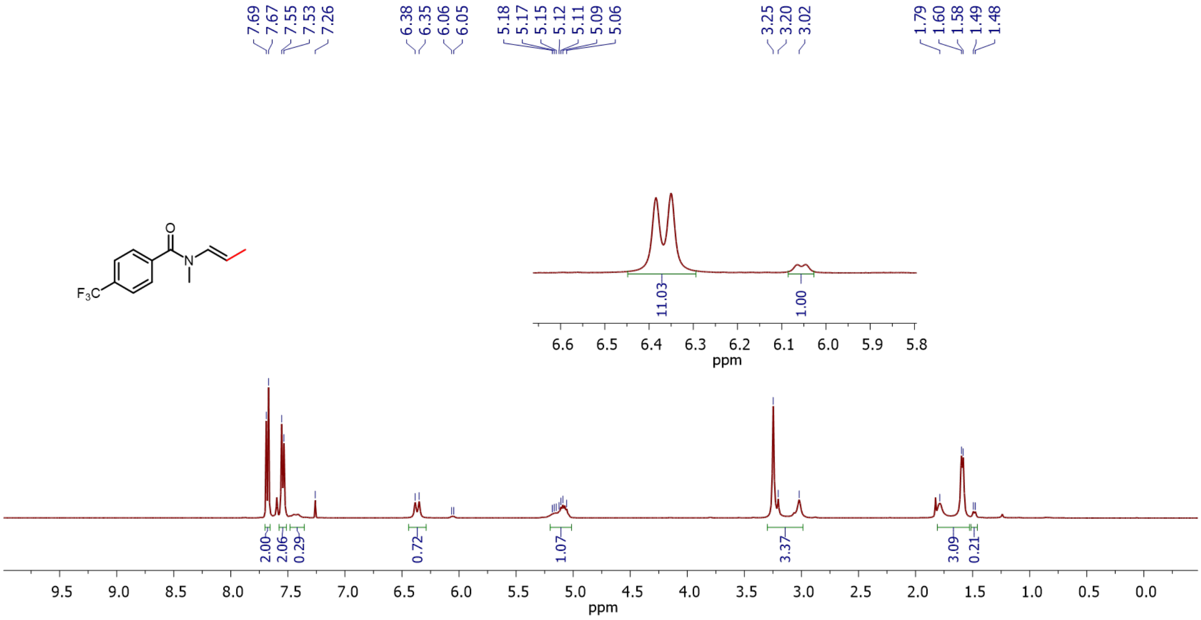

Figure S64. ^{1}H NMR spectrum (400 MHz) of **6e** in $CDCl_3$.

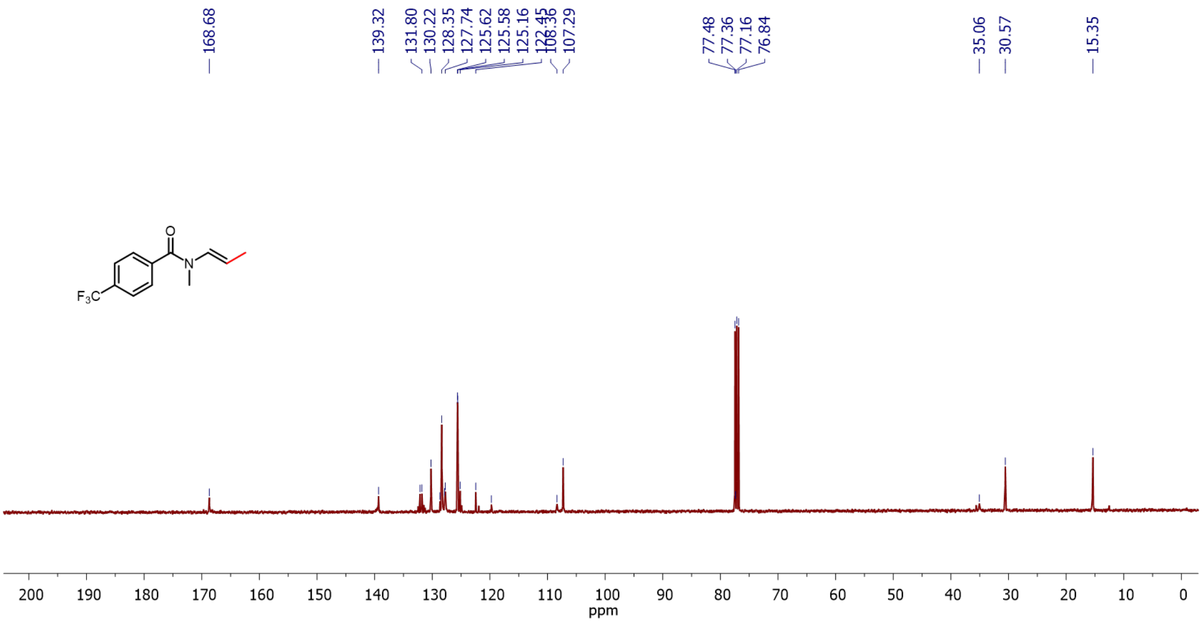

Figure S65. $^{13}C\ \{^{1}H\}$ NMR spectrum (101 MHz) of **6e** in $CDCl_3$.

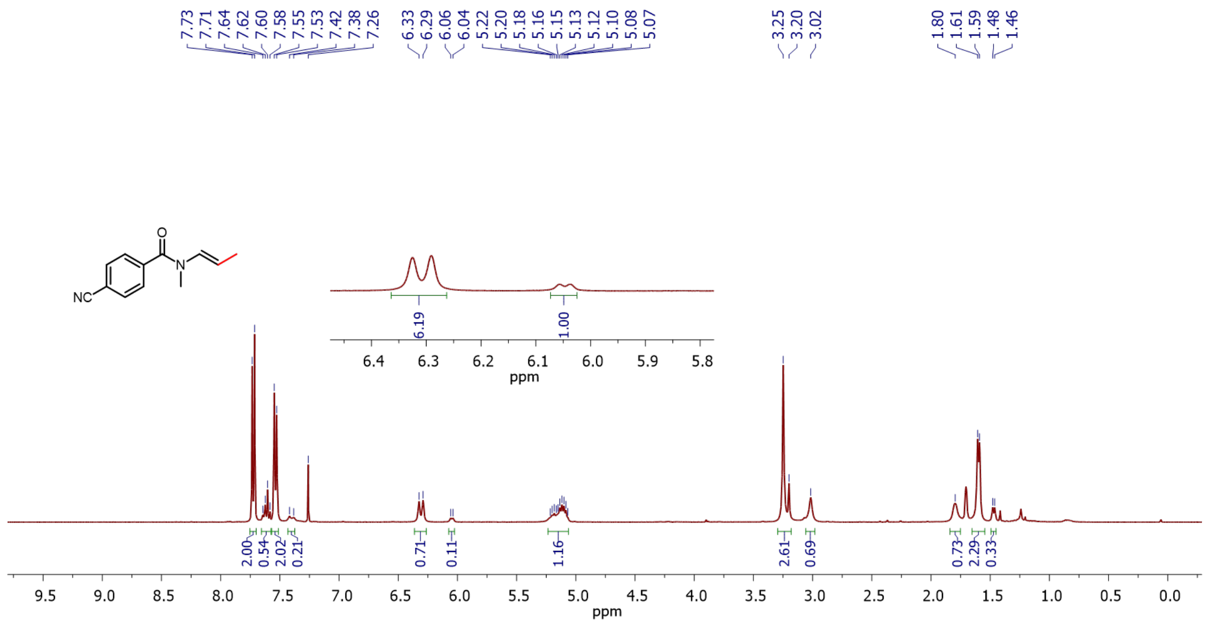

Figure S66. ^{1}H NMR spectrum (400 MHz) of **6f** in $CDCl_3$.

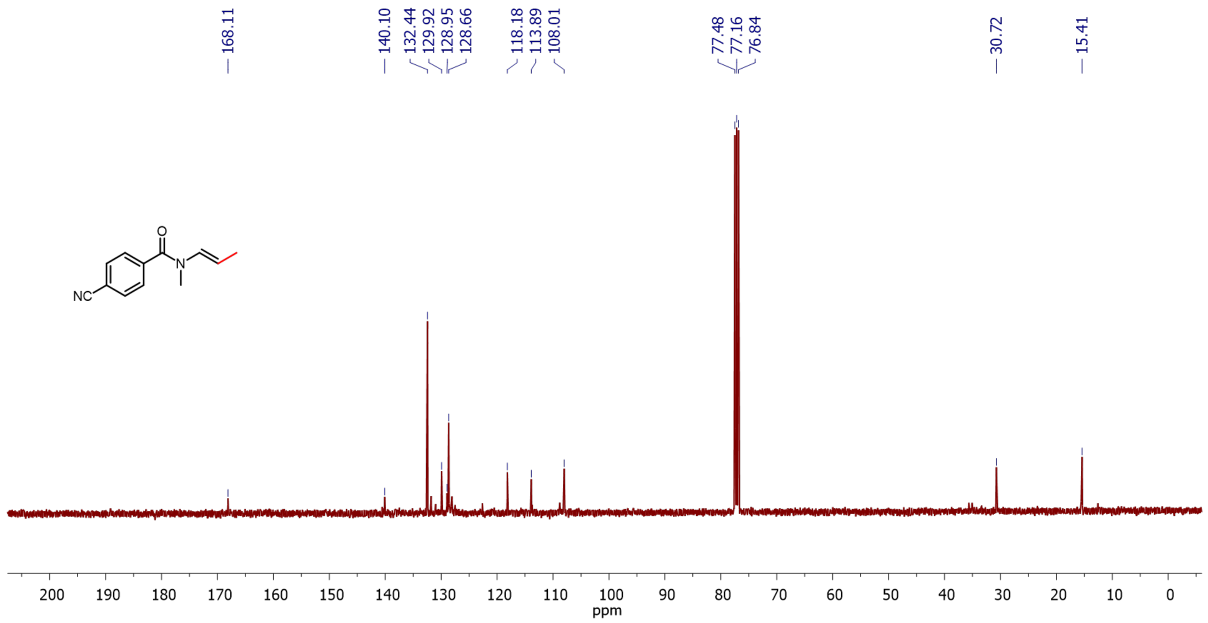

Figure S67. ^{13}C {^{1}H} NMR spectrum (101 MHz) of **6f** in $CDCl_3$.

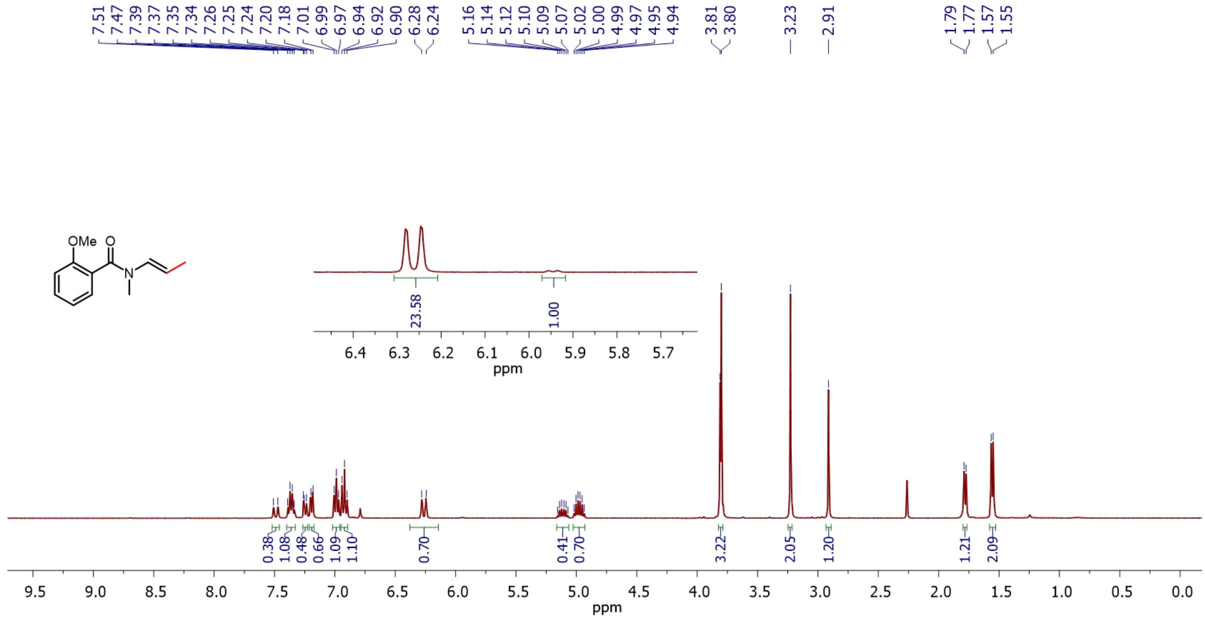

Figure S68. ^{1}H NMR spectrum (400 MHz) of **6g** in $CDCl_3$.

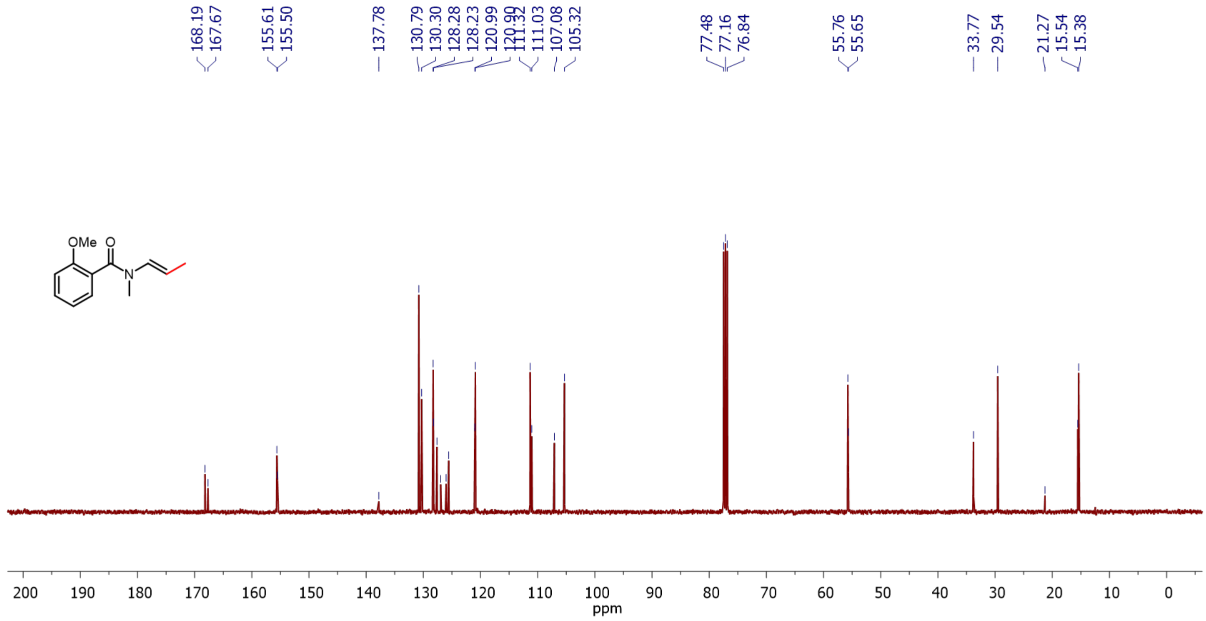

Figure S69. ^{13}C {^{1}H} NMR spectrum (101 MHz) of **6g** in $CDCl_3$.

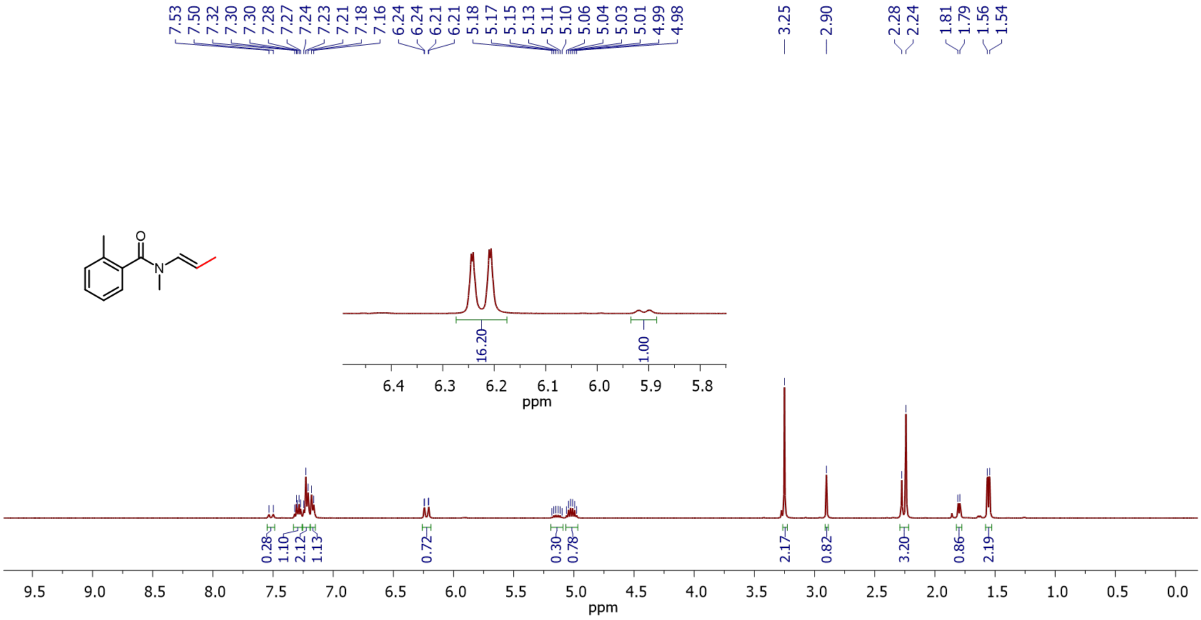

Figure S70. ^{1}H NMR spectrum (400 MHz) of **6h** in $CDCl_3$.

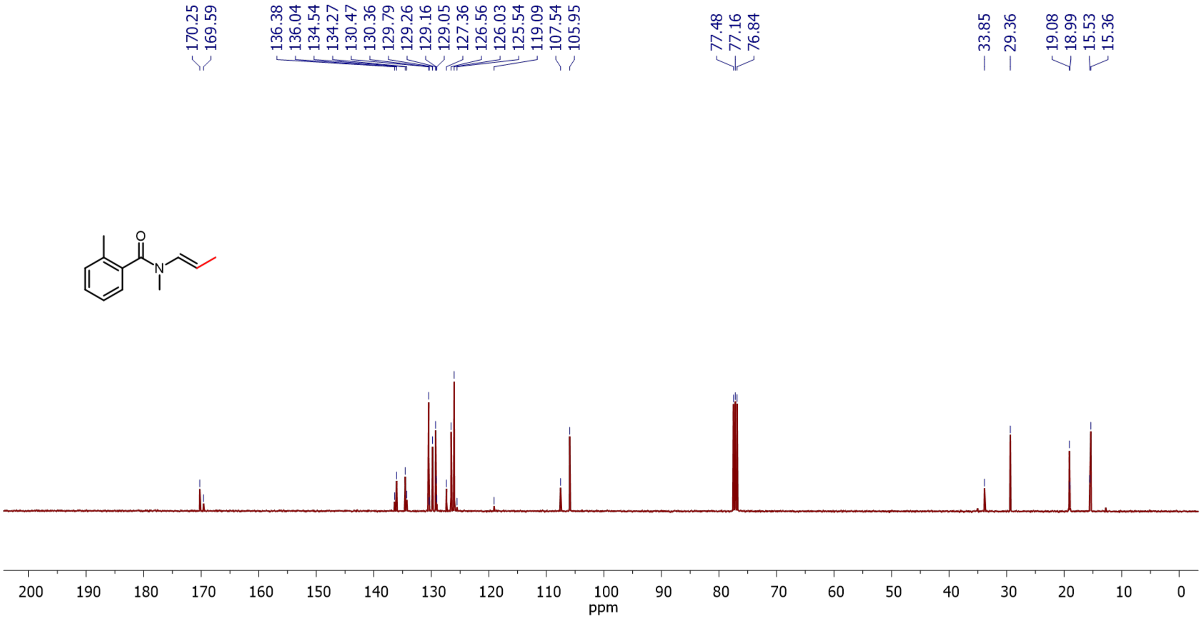

Figure S71. ^{13}C {^{1}H} NMR spectrum (101 MHz) of **6h** in $CDCl_3$.

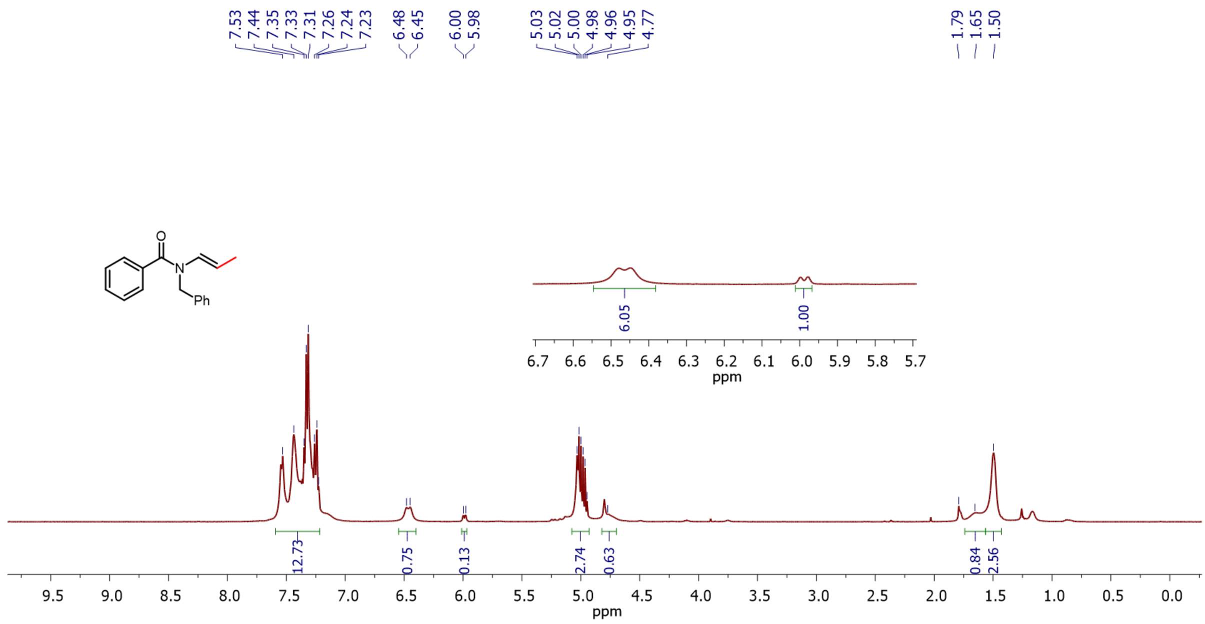

Figure S72. ^{1}H NMR spectrum (400 MHz) of **6i** in $CDCl_3$.

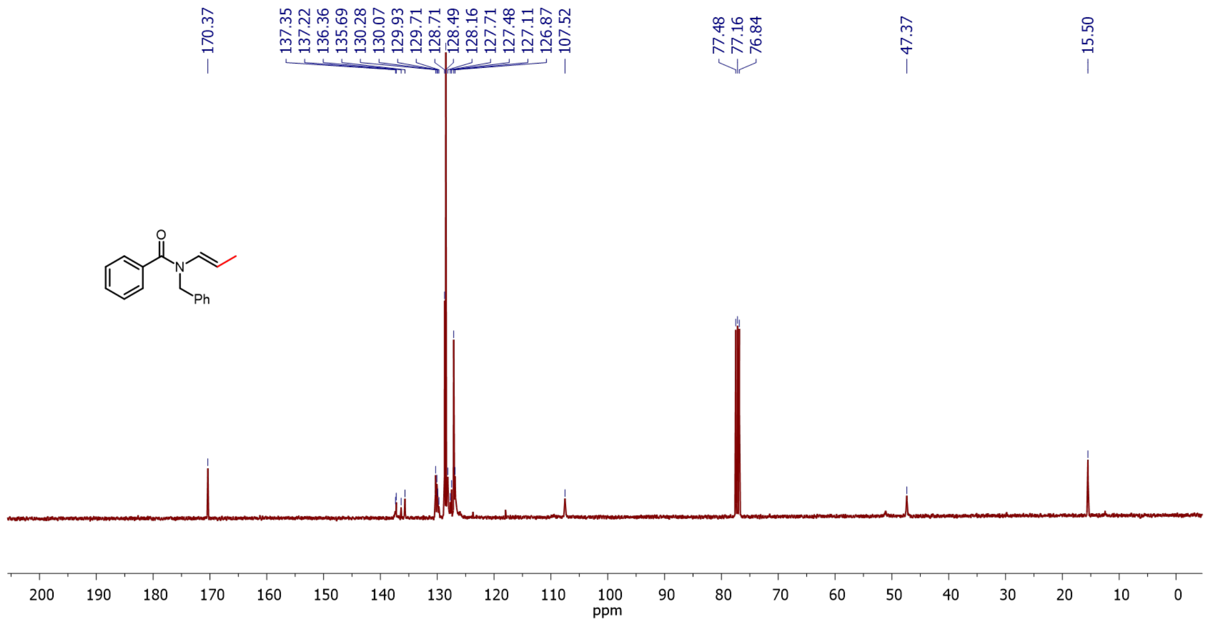

Figure S73. ^{13}C {^{1}H} NMR spectrum (101 MHz) of **6i** in $CDCl_3$.

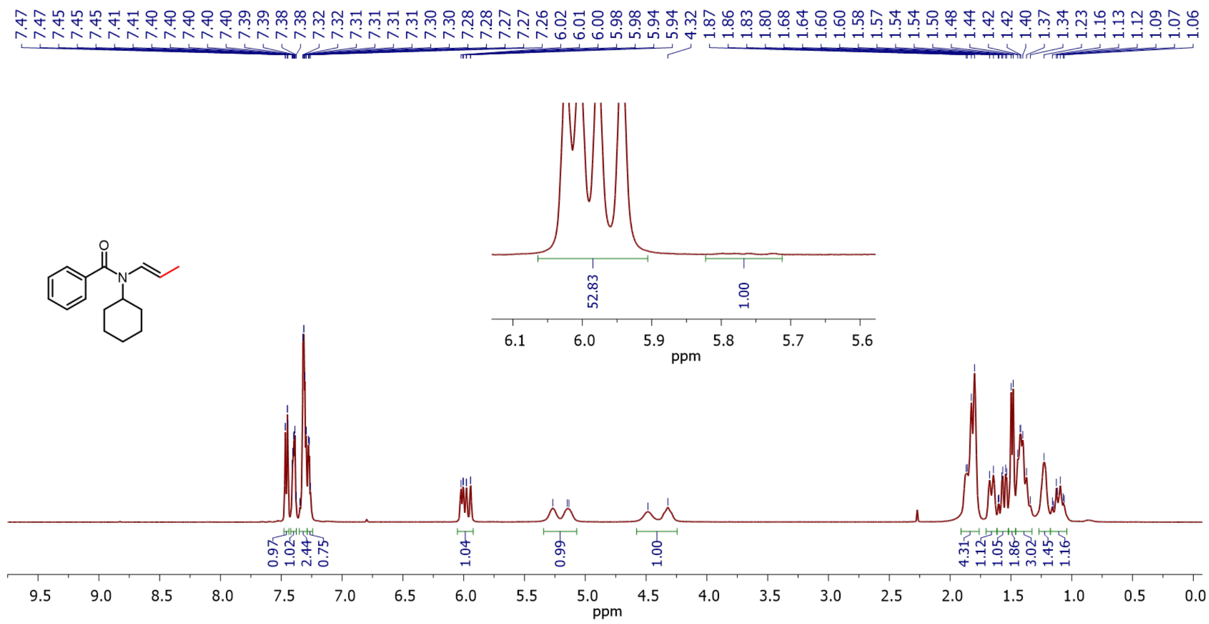

Figure S74. ^{1}H NMR spectrum (400 MHz) of **6j** in $CDCl_3$.

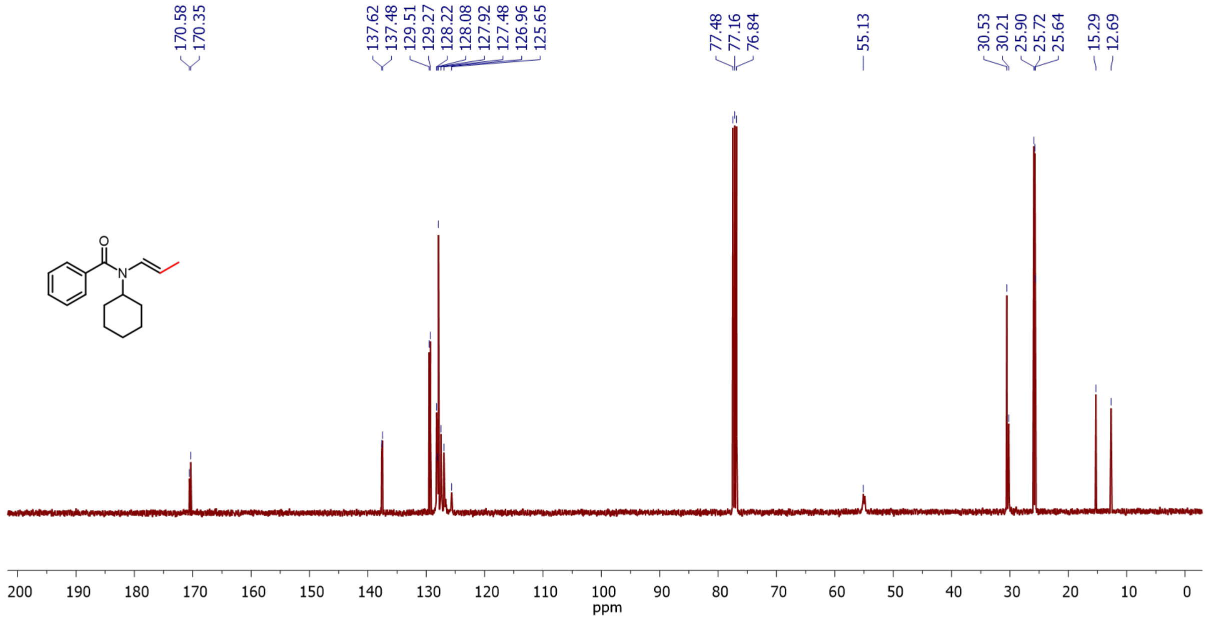

Figure S75. ^{13}C {^{1}H} NMR spectrum (101 MHz) of **6j** in $CDCl_3$.

Compound Spectrum SmartFormula Report

**Analysis Info**			
Analysis Name	D:\Data\Graham\Dr_5223000001.d	Acquisition Date	10/12/2023 11:15:41
Method	APCI_pos_SolidProbe.m	Operator	Larisa Panz
Sample Name	SRK 6j	Instrument	maXis impact 282001.00128
Comment			

Acquisition Parameter

Source Type	APCI	Ion Polarity	Positive	Set Nebulizer	1.2 Bar
Focus	Active	Set Capillary	3000 V	Set Dry Heater	120 °C
Scan Begin	50 m/z	Set End Plate Offset	-500 V	Set Dry Gas	1.5 l/min
Scan End	2000 m/z	Set Charging Voltage	2000 V	Set Divert Valve	Source
		Set Corona	5000 nA	Set APCI Heater	200 °C

+MS, 0.3-0.4min #18-25

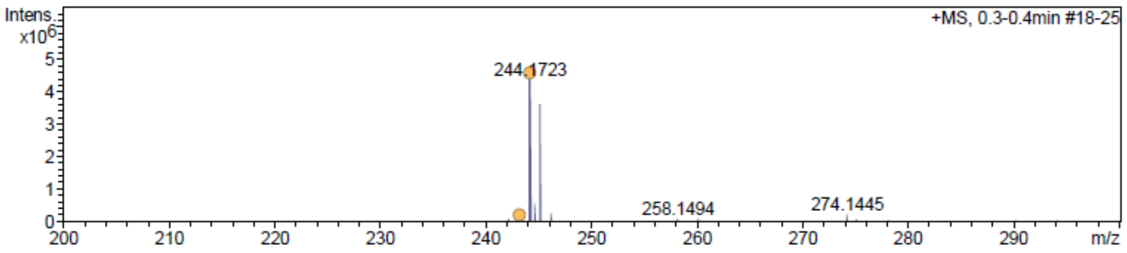

Meas. m/z	#	Ion Formula	m/z	err [ppm]	mSigma	# mSigma	Score	rdb	e⁻ Conf	N-Rule	|err| [mDa]
243.1594	1	C16H21NO	243.1629	14.3	879.6	1	100.00	7.0	odd	ok	3.5
	1	C16H21NO	243.1629	14.3	879.6	1	100.00	7.0	odd	ok	3.5
244.1723	1	C16H22NO	244.1701	-9.0	374.9	1	100.00	6.5	even	ok	2.2
	1	C16H22NO	244.1701	-9.0	374.9	1	100.00	6.5	even	ok	2.2
	1	C16H22NO	244.1707	-6.8	374.9	1	100.00	6.5	even	ok	1.7

Figure S76. High resolution mass spectrum of **6j.**

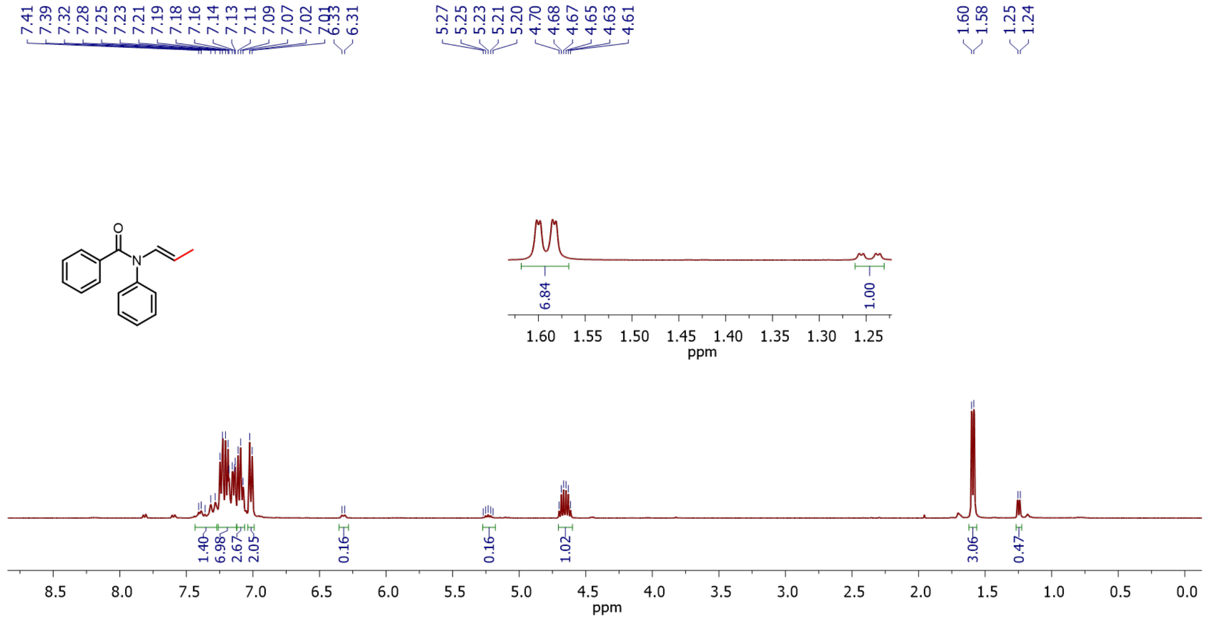

Figure S77. ^{1}H NMR spectrum (400 MHz) of **6k** in $CDCl_3$.

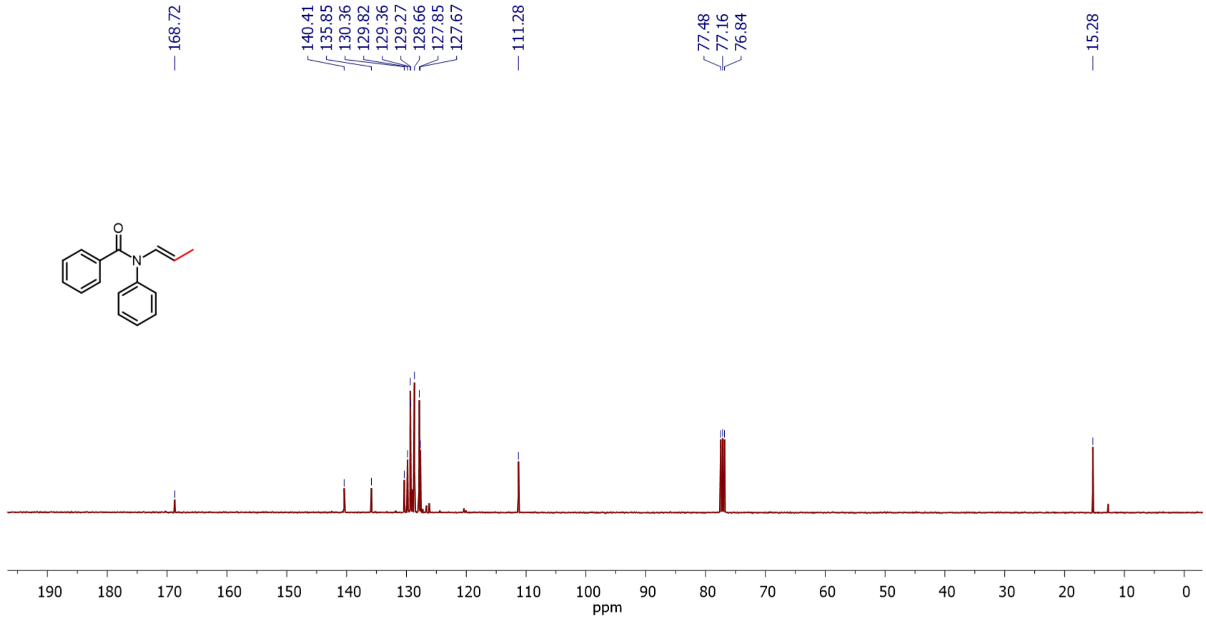

Figure S78. ^{13}C {^{1}H} NMR spectrum (101 MHz) of **6k** in $CDCl_3$.

Compound Spectrum SmartFormula Report

Analysis Info

Analysis Name	D:\Data\Graham\Dr_5224000001.d	Acquisition Date	10/12/2023 11:18:36
Method	APCI_pos_SolidProbe.m	Operator	Larisa Panz
Sample Name	SRK 6K	Instrument	maXis impact 282001.00128
Comment			

Acquisition Parameter

Source Type	APCI	Ion Polarity	Positive	Set Nebulizer	1.2 Bar
Focus	Active	Set Capillary	3000 V	Set Dry Heater	120 °C
Scan Begin	50 m/z	Set End Plate Offset	-500 V	Set Dry Gas	1.5 l/min
Scan End	2000 m/z	Set Charging Voltage	2000 V	Set Divert Valve	Source
		Set Corona	5000 nA	Set APCI Heater	200 °C

+MS, 0.6-0.6min #34-37

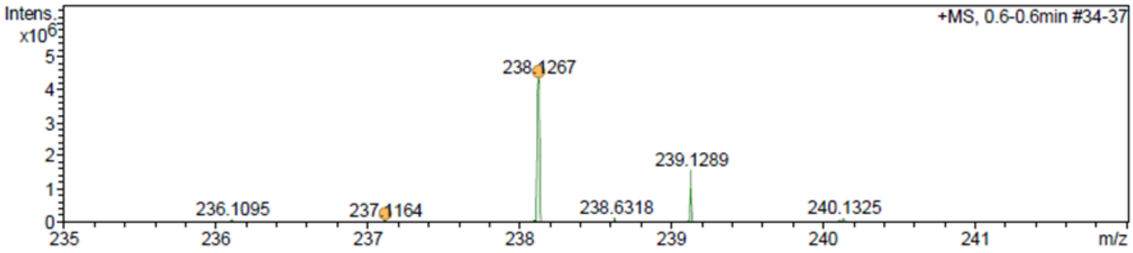

Meas. m/z	#	Ion Formula	m/z	err [ppm]	mSigma	# mSigma	Score	rdb	e⁻ Conf	N-Rule	|err| [mDa]
237.1164	1	C16H15NO	237.1159	-2.0	766.6	1	100.00	10.0	odd	ok	0.5
	1	C16H15NO	237.1159	-2.0	766.6	1	100.00	10.0	odd	ok	0.5
238.1267	1	C16H16NO	238.1232	-14.8	105.7	1	100.00	9.5	even	ok	3.5
	1	C16H16NO	238.1232	-14.8	105.7	1	100.00	9.5	even	ok	3.5

Figure S79. High resolution mass spectrum of **6k**.

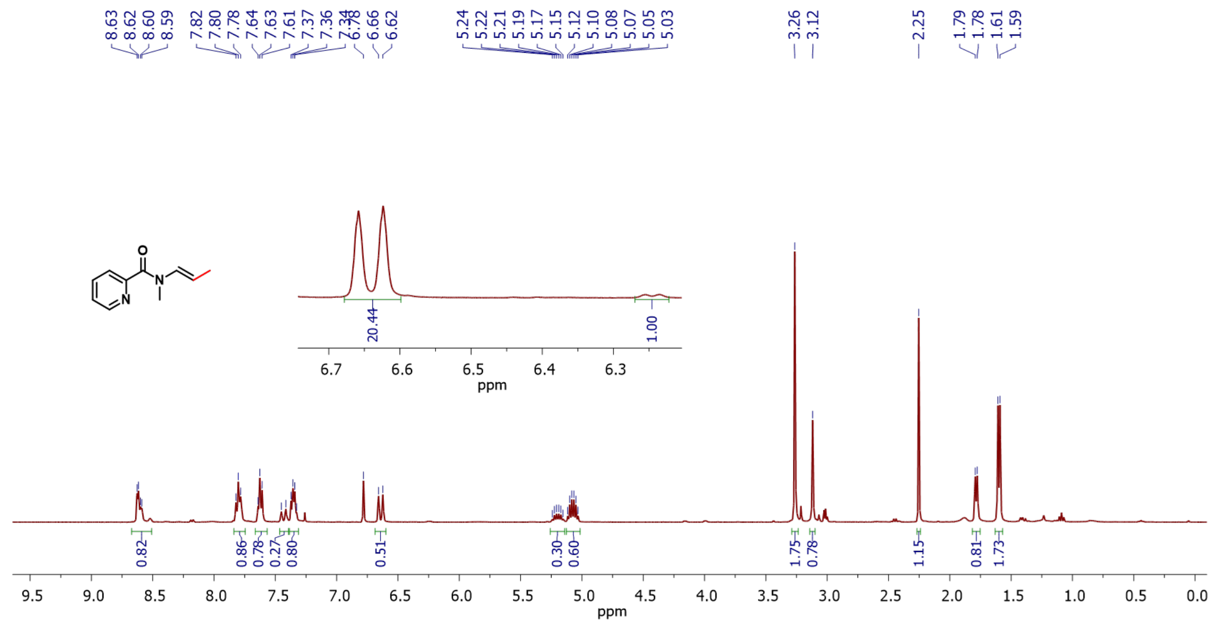

Figure S80. ^{1}H NMR spectrum (400 MHz) of **6l** in $CDCl_3$.

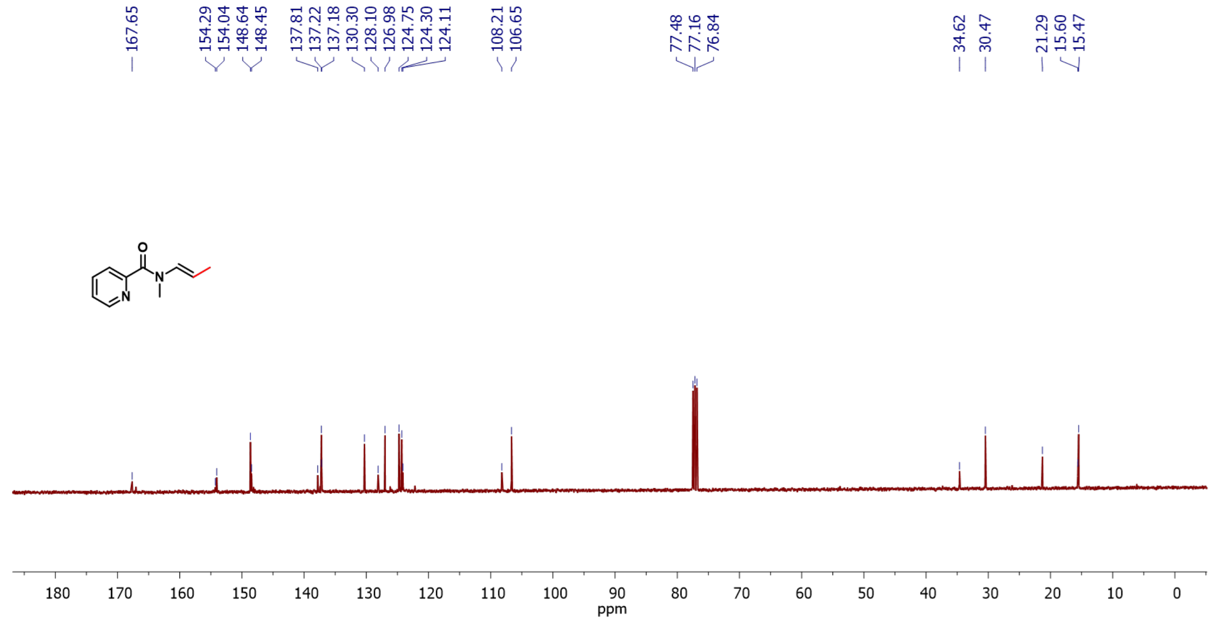

Figure S81. ^{13}C {^{1}H} NMR spectrum (101 MHz) of **6l** in $CDCl_3$.

NMR and HRMS of Isomerized N-substituted allylimines.

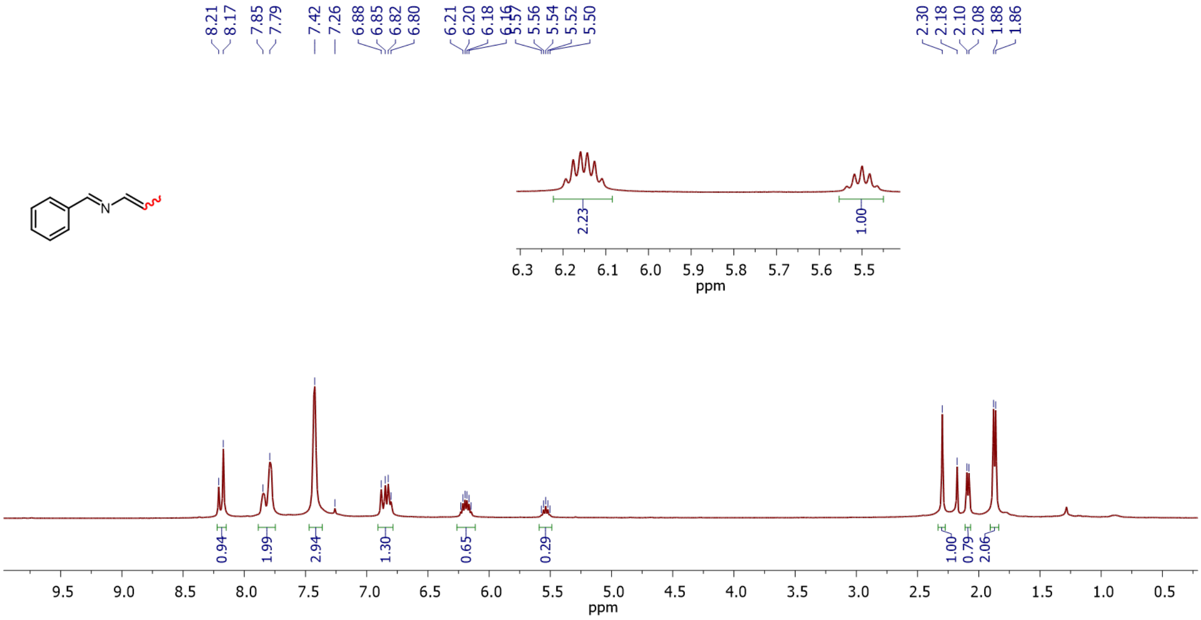

Figure S82. ^{1}H NMR spectrum (400 MHz) of **7a** in $CDCl_3$.

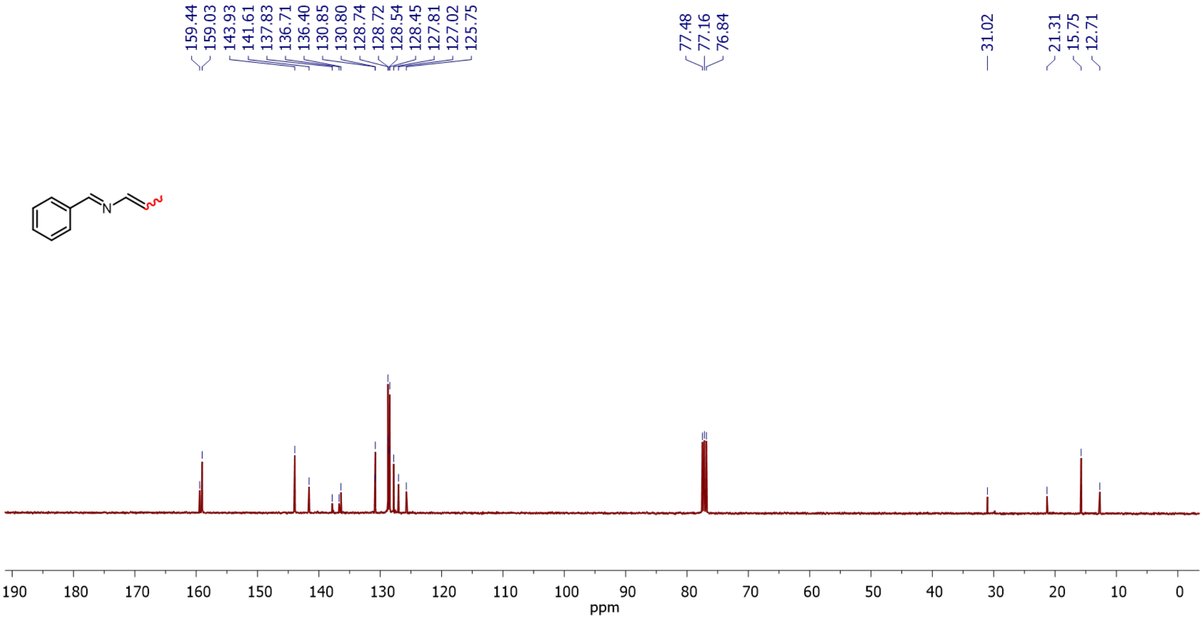

Figure S83. ^{13}C {^{1}H} NMR spectrum (101 MHz) of **7a** in $CDCl_3$.

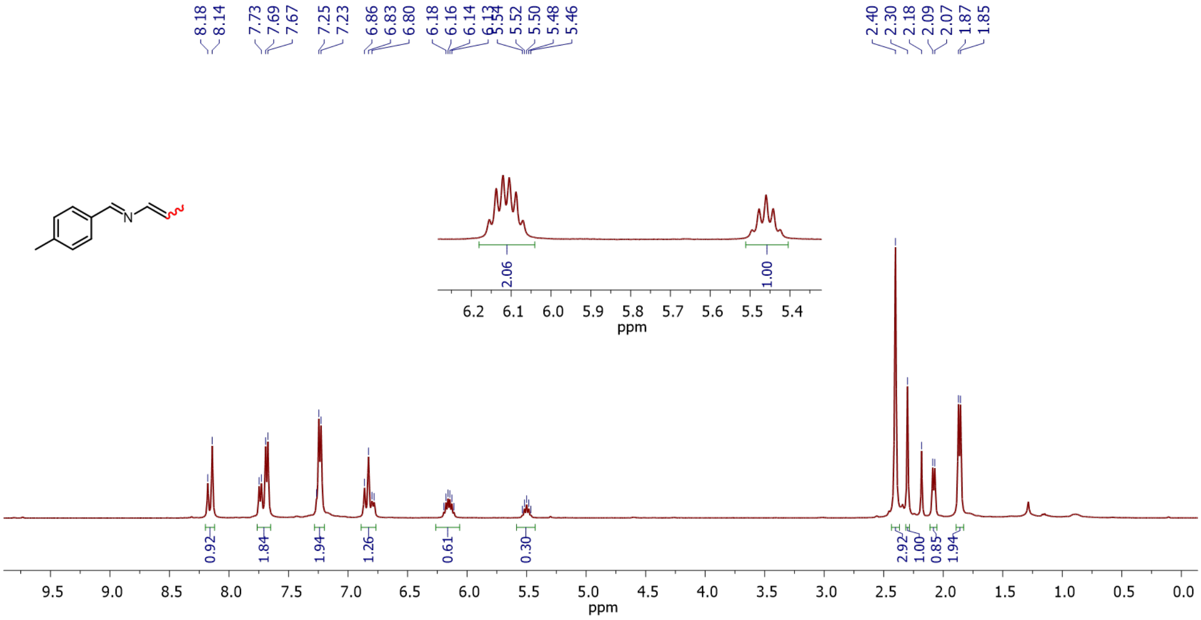

Figure S84. ^{1}H NMR spectrum (400 MHz) of **7b** in $CDCl_3$.

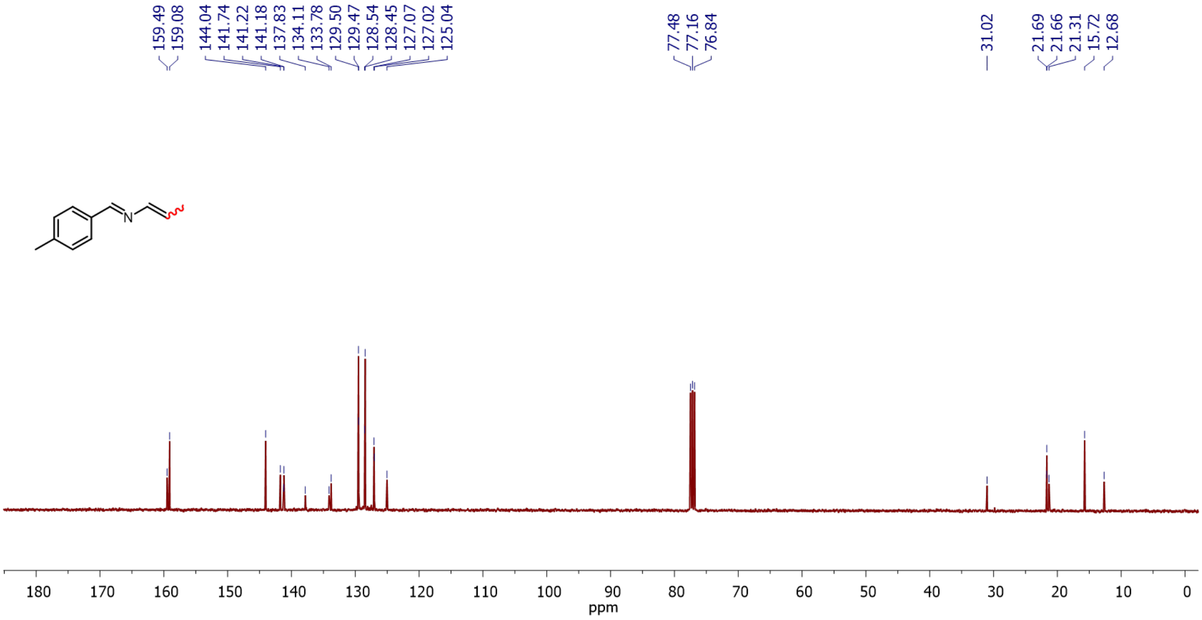

Figure S85. ^{13}C {^{1}H} NMR spectrum (101 MHz) of **7b** in $CDCl_3$.

Compound Spectrum SmartFormula Report

Analysis Info

Analysis Name	D:\Data\Graham\Dr_5225000001.d	Acquisition Date	10/12/2023 11:21:33
Method	APCI_pos_SolidProbe.m	Operator	Larisa Panz
Sample Name	SRK 7b	Instrument	maXis impact 282001.00128
Comment			

Acquisition Parameter

Source Type	APCI	Ion Polarity	Positive	Set Nebulizer	1.2 Bar
Focus	Active	Set Capillary	3000 V	Set Dry Heater	120 °C
Scan Begin	50 m/z	Set End Plate Offset	-500 V	Set Dry Gas	1.5 l/min
Scan End	2000 m/z	Set Charging Voltage	2000 V	Set Divert Valve	Source
		Set Corona	5000 nA	Set APCI Heater	200 °C

+MS, 0.6-0.6min #33-36

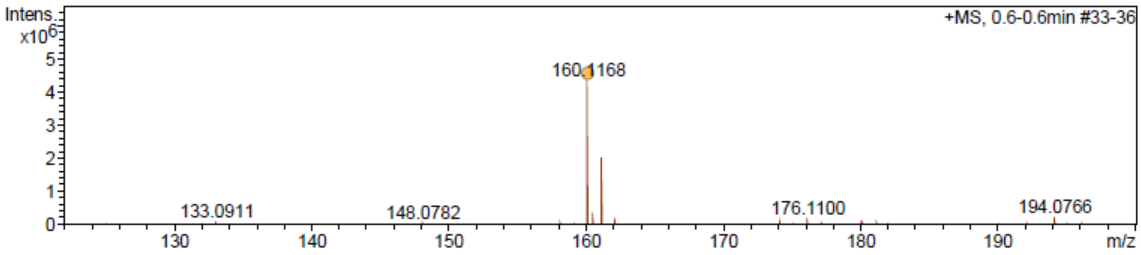

Meas. m/z	#	Ion Formula	m/z	err [ppm]	mSigma	# mSigma	Score	rdb	e⁻ Conf	N-Rule	|err| [mDa]
160.1168	1	C11H14N	160.1126	-26.1	197.8	1	100.00	5.5	even	ok	4.2
	2	C7H14NO3	160.0974	-121.3	221.9	2	0.00	1.5	even	ok	19.4
	1	C11H14N	160.1126	-26.1	197.8	1	100.00	5.5	even	ok	4.2
	2	C7H14NO3	160.0974	-121.3	221.9	2	0.00	1.5	even	ok	19.4

Figure S86. High resolution mass spectrum of **7b.**

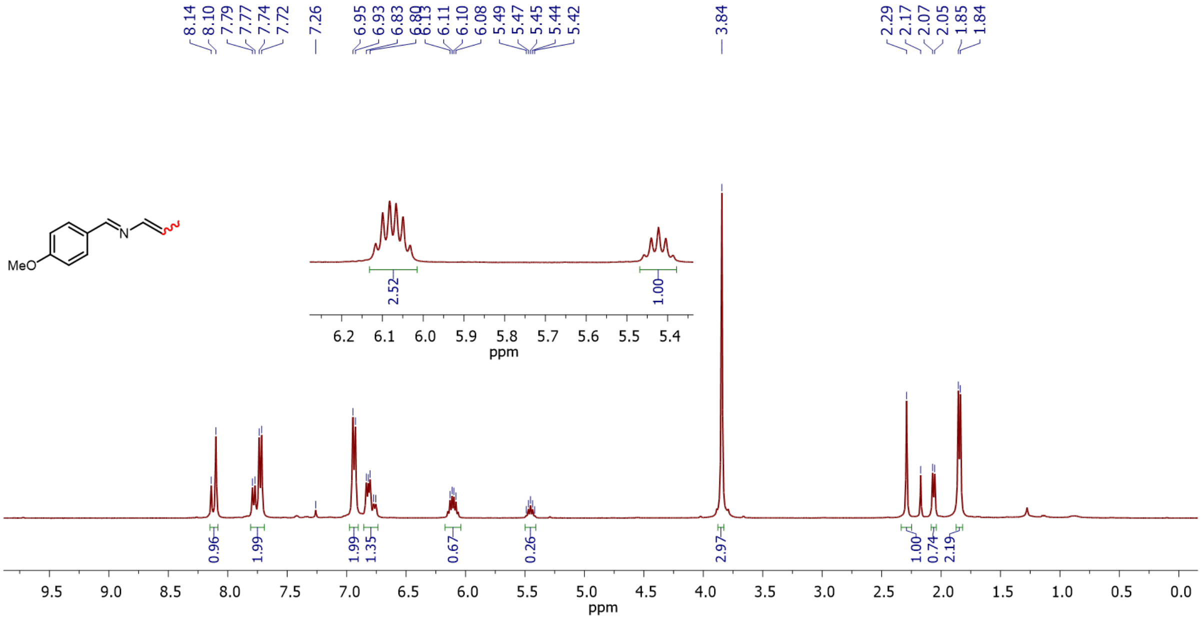

Figure S87. ^{1}H NMR spectrum (400 MHz) of **7c** in $CDCl_3$.

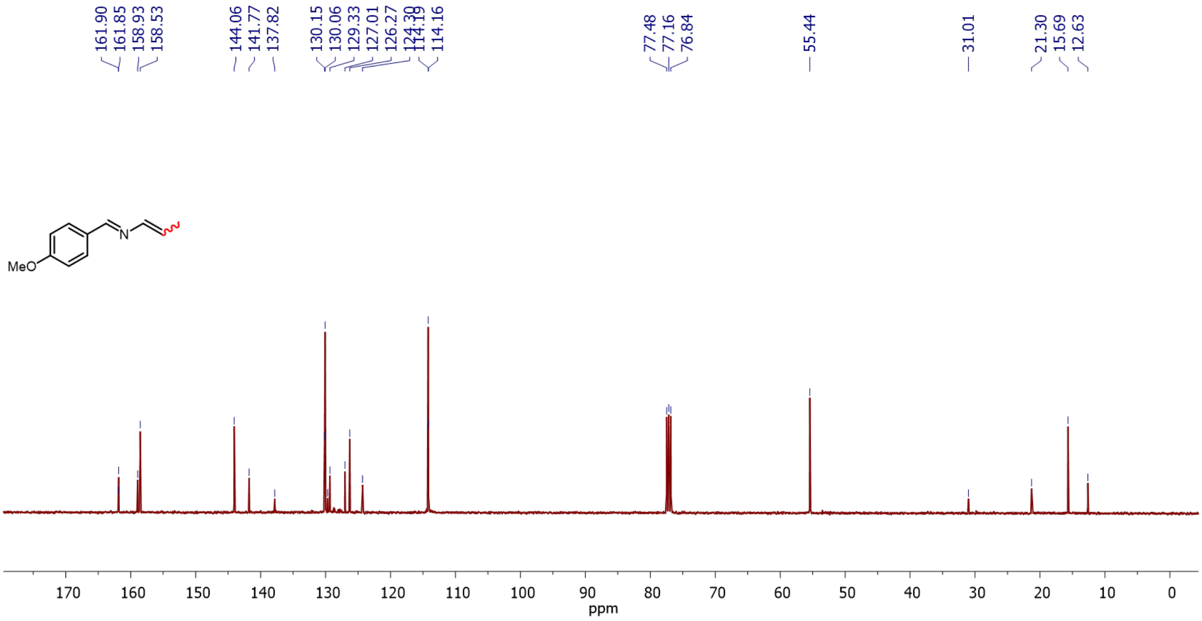

Figure S88. ^{13}C {^{1}H} NMR spectrum (101 MHz) of **7c** in $CDCl_3$.

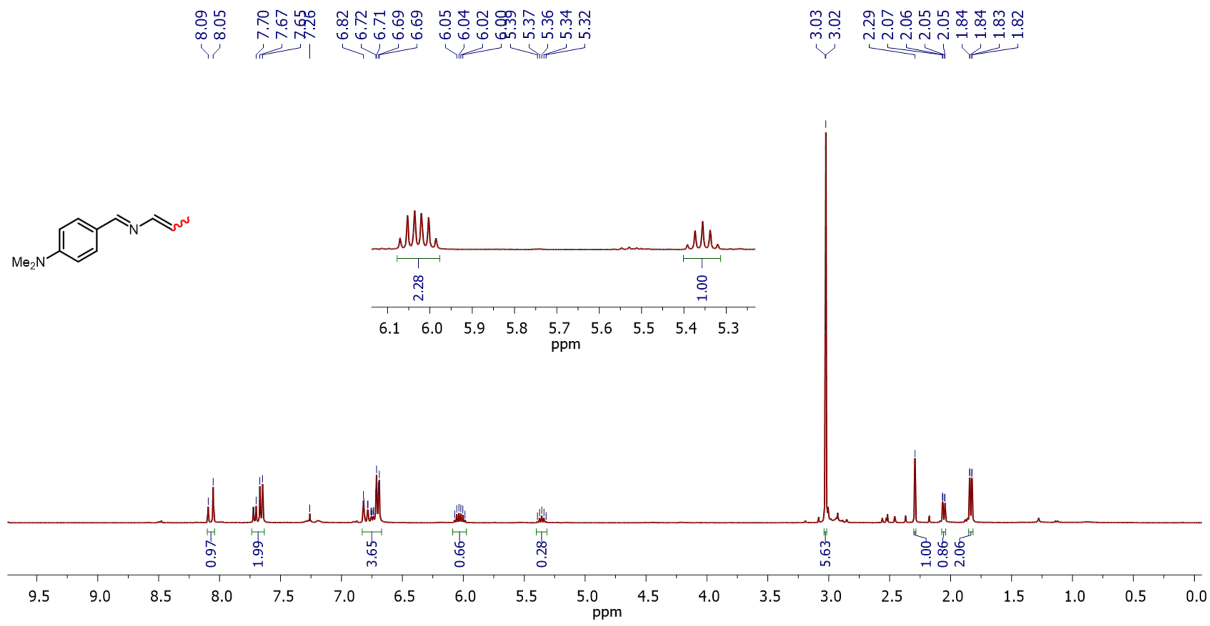

Figure S89. ^{1}H NMR spectrum (400 MHz) of **7d** in $CDCl_3$.

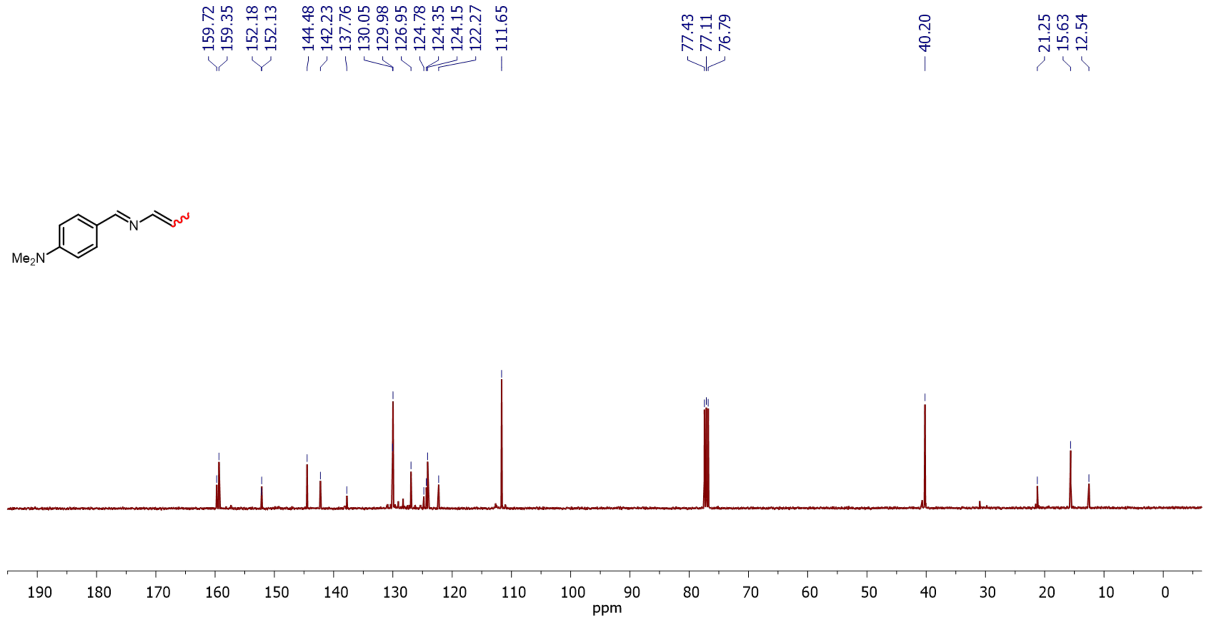

Figure S90. ^{13}C {^{1}H} NMR spectrum (101 MHz) of **7d** in $CDCl_3$.

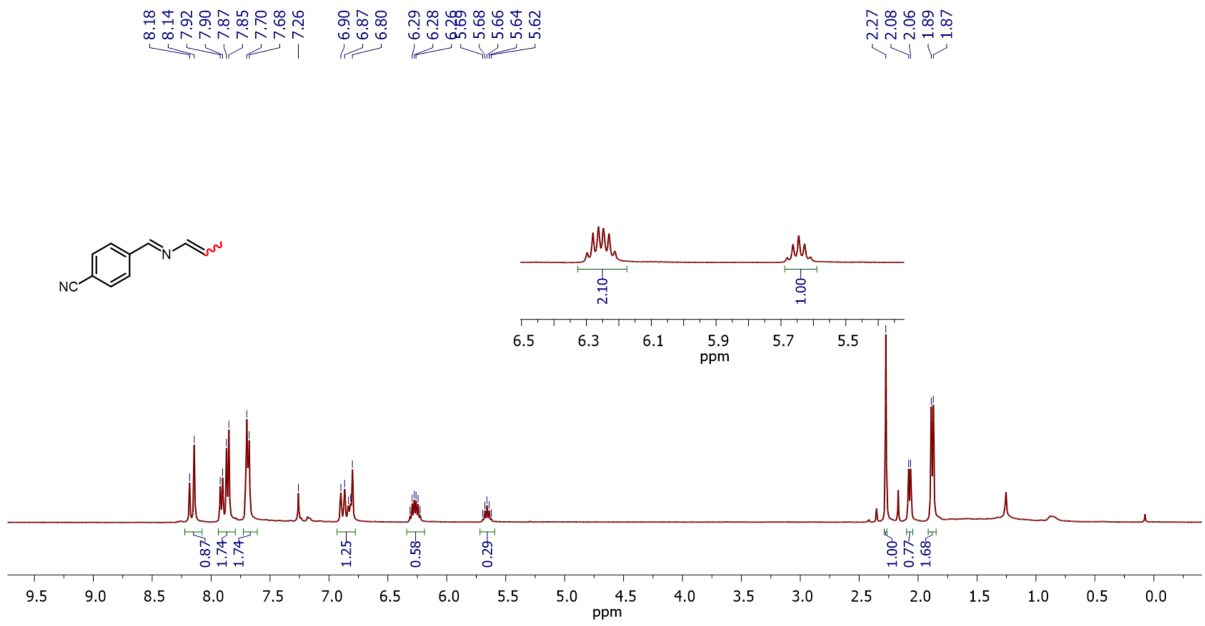

Figure S91. ^{1}H NMR spectrum (400 MHz) of **7e** in $CDCl_3$.

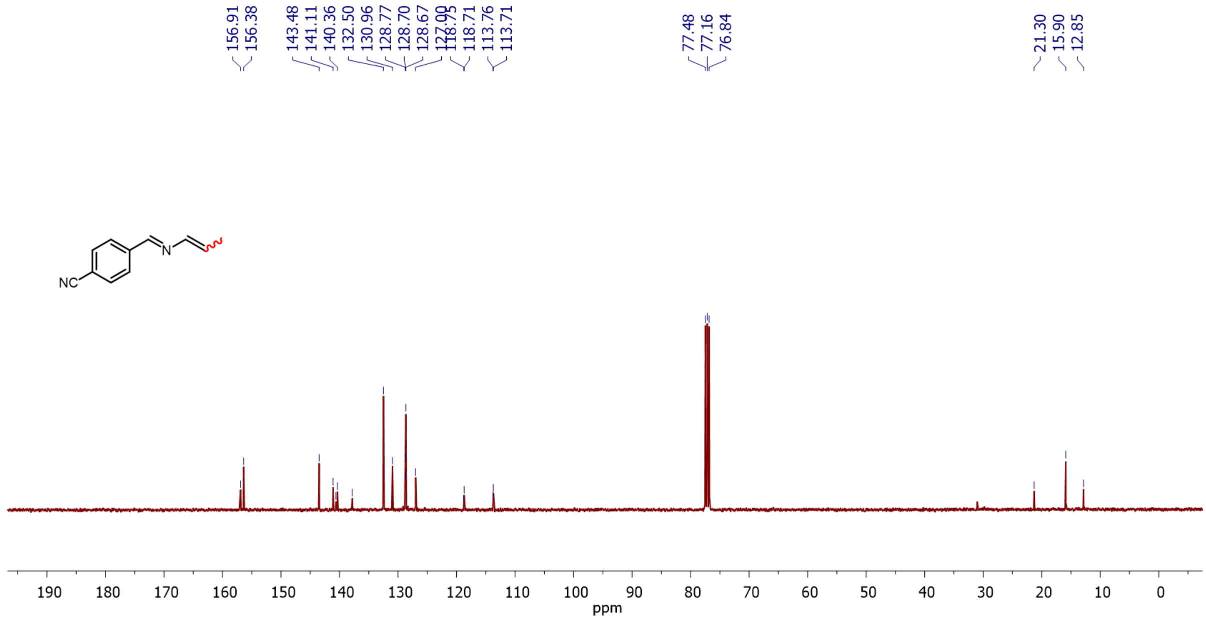

Figure S92. ^{13}C {^{1}H} NMR spectrum (101 MHz) of **7e** in $CDCl_3$.

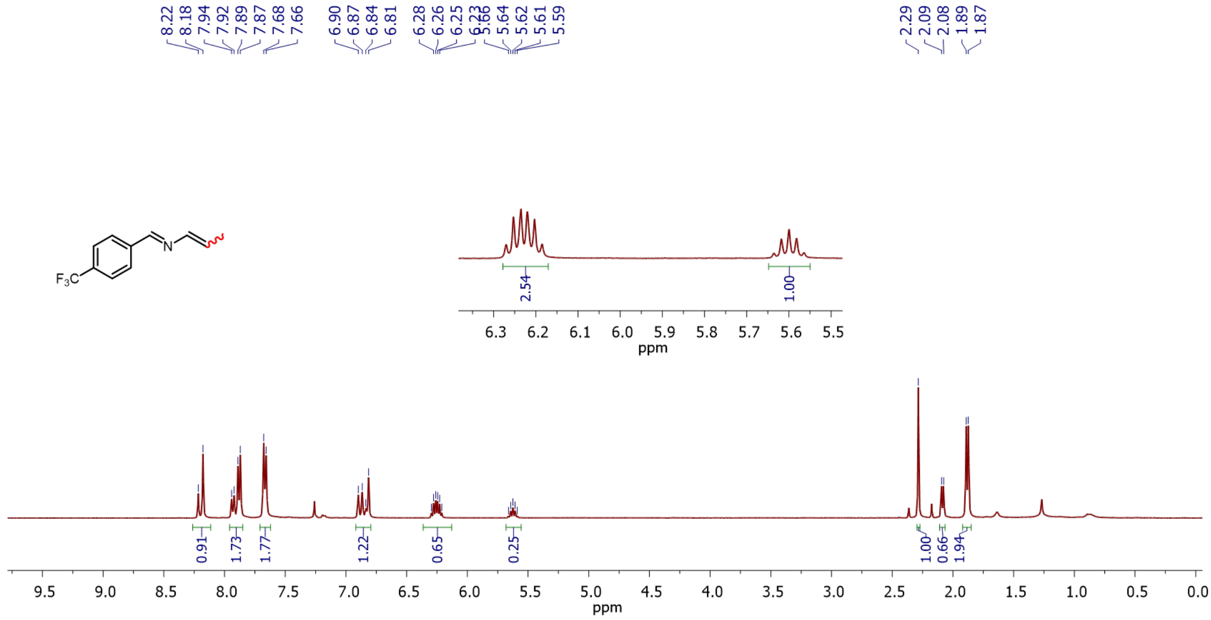

Figure S93. ^{1}H NMR spectrum (400 MHz) of **7f** in $CDCl_3$.

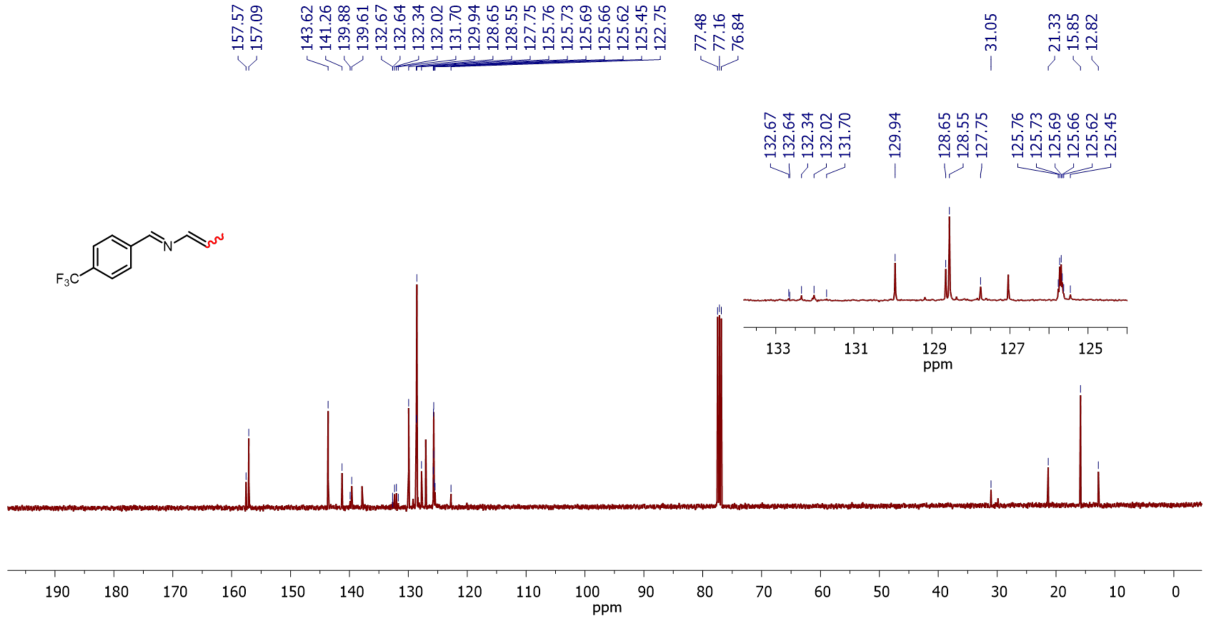

Figure S94. ^{13}C {^{1}H} NMR spectrum (101 MHz) of **7f** in $CDCl_3$.

Compound Spectrum SmartFormula Report

Analysis Info

Analysis Name	D:\Data\Graham\Dr_5226000001.d	Acquisition Date	10/12/2023 11:24:12
Method	APCI_pos_SolidProbe.m	Operator	Larisa Panz
Sample Name	SRK 7f	Instrument	maXis impact 282001.00128
Comment			

Acquisition Parameter

Source Type	APCI	Ion Polarity	Positive	Set Nebulizer	1.2 Bar
Focus	Active	Set Capillary	3000 V	Set Dry Heater	120 °C
Scan Begin	50 m/z	Set End Plate Offset	-500 V	Set Dry Gas	1.5 l/min
Scan End	2000 m/z	Set Charging Voltage	2000 V	Set Divert Valve	Source
		Set Corona	5000 nA	Set APCI Heater	200 °C

+MS, 0.6-0.7min #36-38

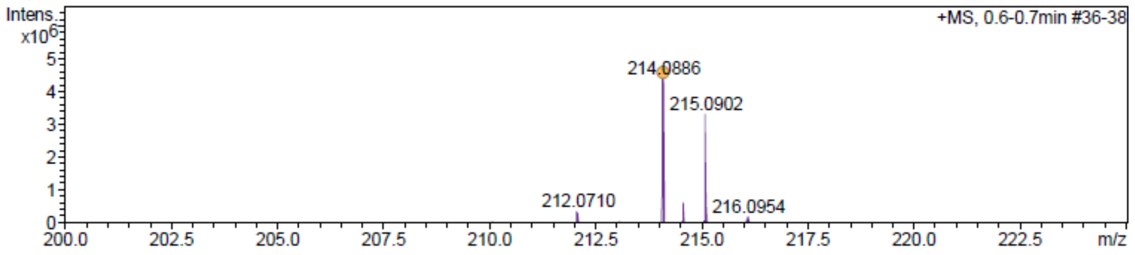

Meas. m/z	#	Ion Formula	m/z	err [ppm]	mSigma	# mSigma	Score	rdb	e⁻ Conf	N-Rule	|err| [mDa]
214.0886	1	C11H11F3N	214.0844	-19.9	368.0	1	100.00	5.5	even	ok	4.3
	2	C7H11F3NO3	214.0691	-91.2	392.3	2	0.00	1.5	even	ok	19.5
	1	C11H11F3N	214.0844	-19.9	368.0	1	100.00	5.5	even	ok	4.3
	2	C7H11F3NO3	214.0691	-91.2	392.3	2	0.00	1.5	even	ok	19.5

Figure S95. High resolution mass spectrum of **7f.**

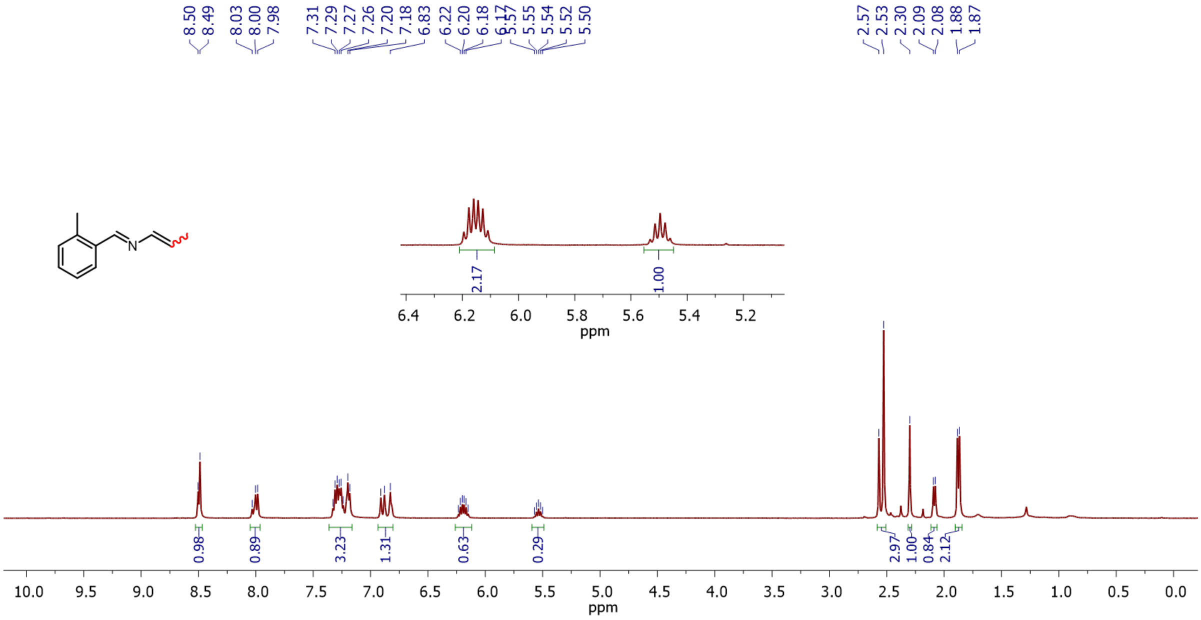

Figure S96. ^{1}H NMR spectrum (400 MHz) of **7g** in $CDCl_3$.

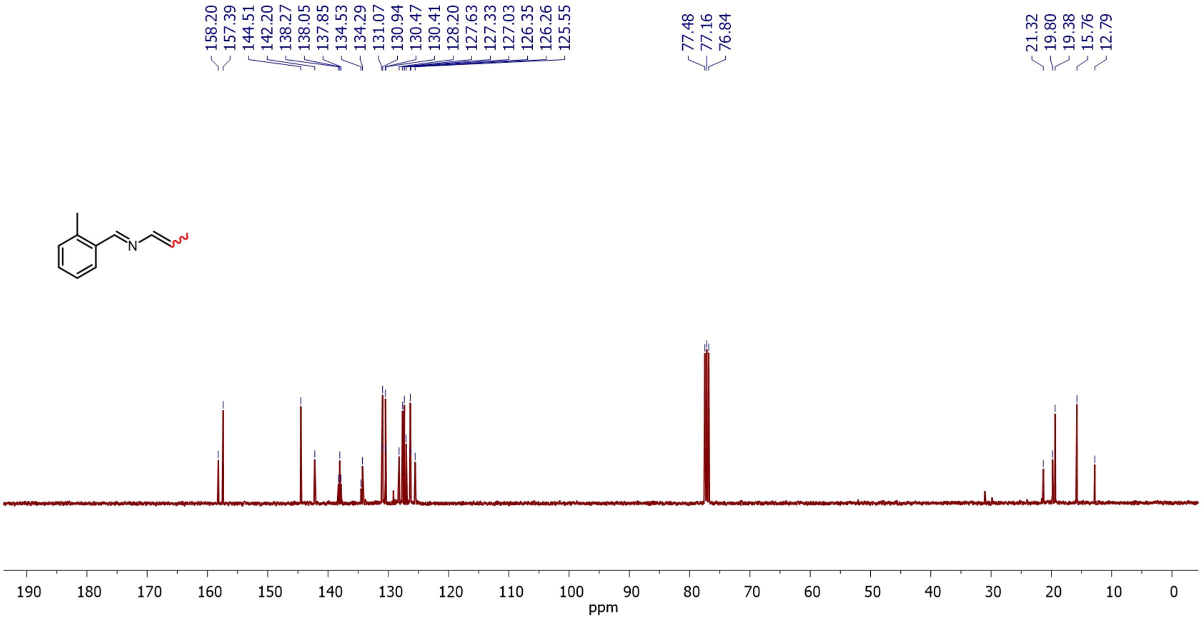

Figure S97. ^{13}C {^{1}H} NMR spectrum (101 MHz) of **7g** in $CDCl_3$.

Compound Spectrum SmartFormula Report

Analysis Info

Analysis Name	D:\Data\Graham\Dr_5227000001.d	Acquisition Date	10/12/2023 11:27:22
Method	APCI_pos_SolidProbe.m	Operator	Larisa Panz
Sample Name	SRK 7g	Instrument	maXis impact 282001.00128
Comment			

Acquisition Parameter

Source Type	APCI	Ion Polarity	Positive	Set Nebulizer	1.2 Bar
Focus	Active	Set Capillary	3000 V	Set Dry Heater	120 °C
Scan Begin	50 m/z	Set End Plate Offset	-500 V	Set Dry Gas	1.5 l/min
Scan End	2000 m/z	Set Charging Voltage	2000 V	Set Divert Valve	Source
		Set Corona	5000 nA	Set APCI Heater	200 °C

+MS, 0.5-0.5min #27-30

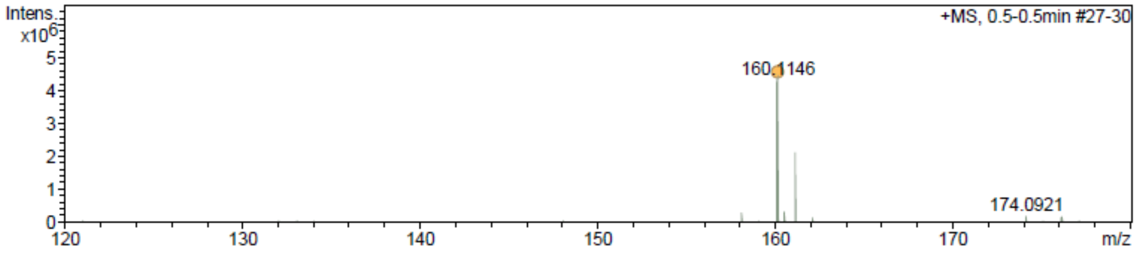

Meas. m/z	#	Ion Formula	m/z	err [ppm]	mSigma	# mSigma	Score	rdb	e⁻ Conf	N-Rule	|err| [mDa]
160.1146	1	C11H14N	160.1126	-12.1	208.3	1	100.00	5.5	even	ok	1.9
	2	C7H14NO3	160.0974	-107.4	232.6	2	0.00	1.5	even	ok	17.2
	1	C11H14N	160.1126	-12.1	208.3	1	100.00	5.5	even	ok	1.9
	2	C7H14NO3	160.0974	-107.4	232.6	2	0.00	1.5	even	ok	17.2

Figure S98. High resolution mass spectrum of **7g.**

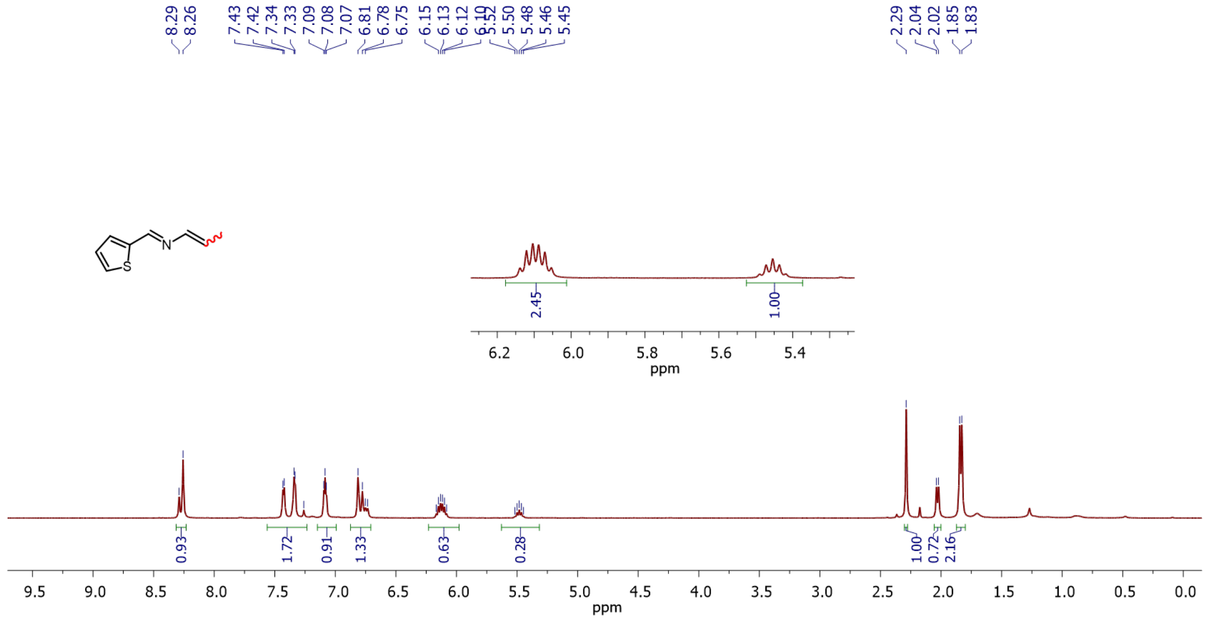

Figure S99. ^{1}H NMR spectrum (400 MHz) of **7h** in $CDCl_3$.

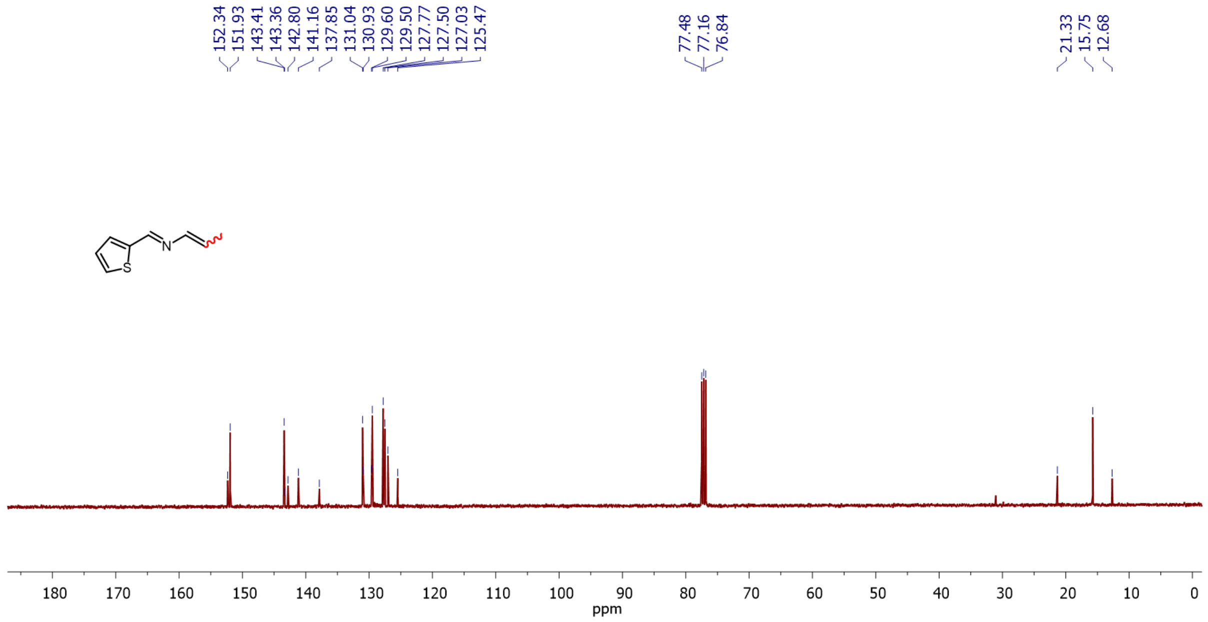

Figure S100. ^{13}C {^{1}H} NMR spectrum (101 MHz) of **7h** in $CDCl_3$.

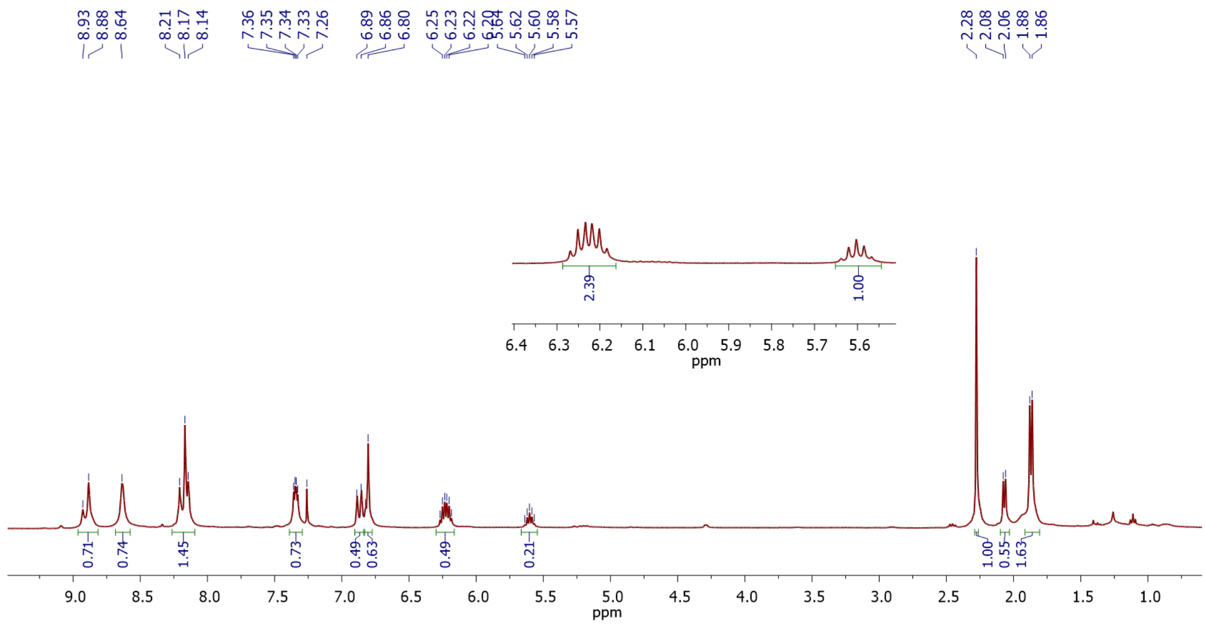

Figure S101. ^{1}H NMR spectrum (400 MHz) of **7i** in $CDCl_3$.

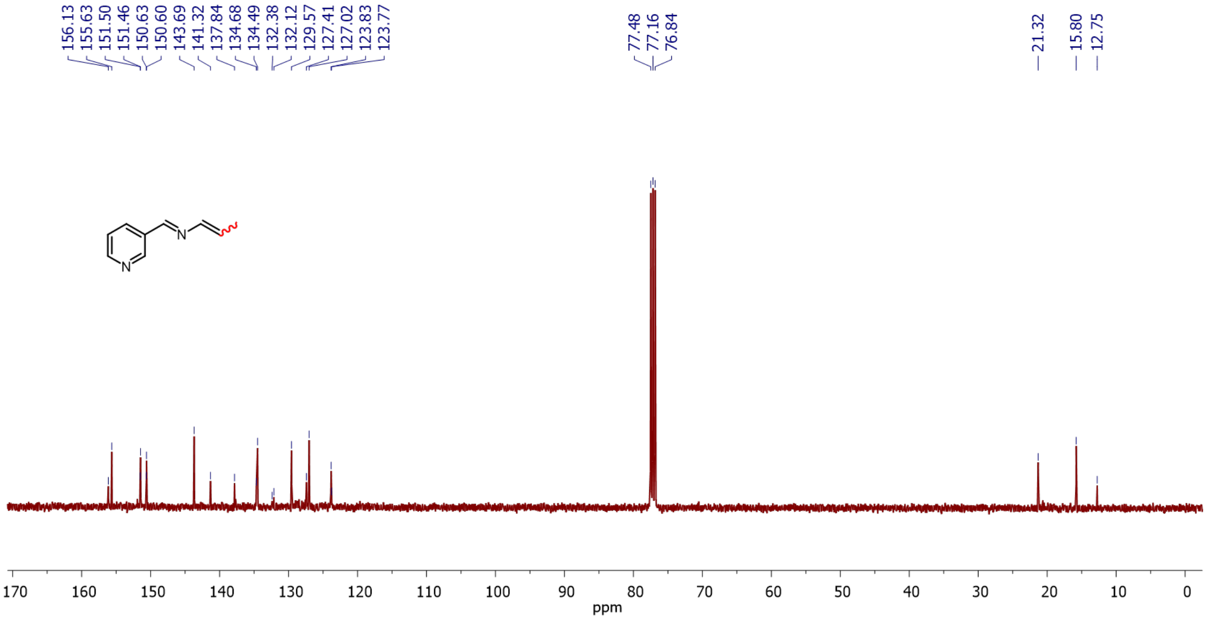

Figure S102. ^{13}C {^{1}H} NMR spectrum (101 MHz) of **7i** in $CDCl_3$.

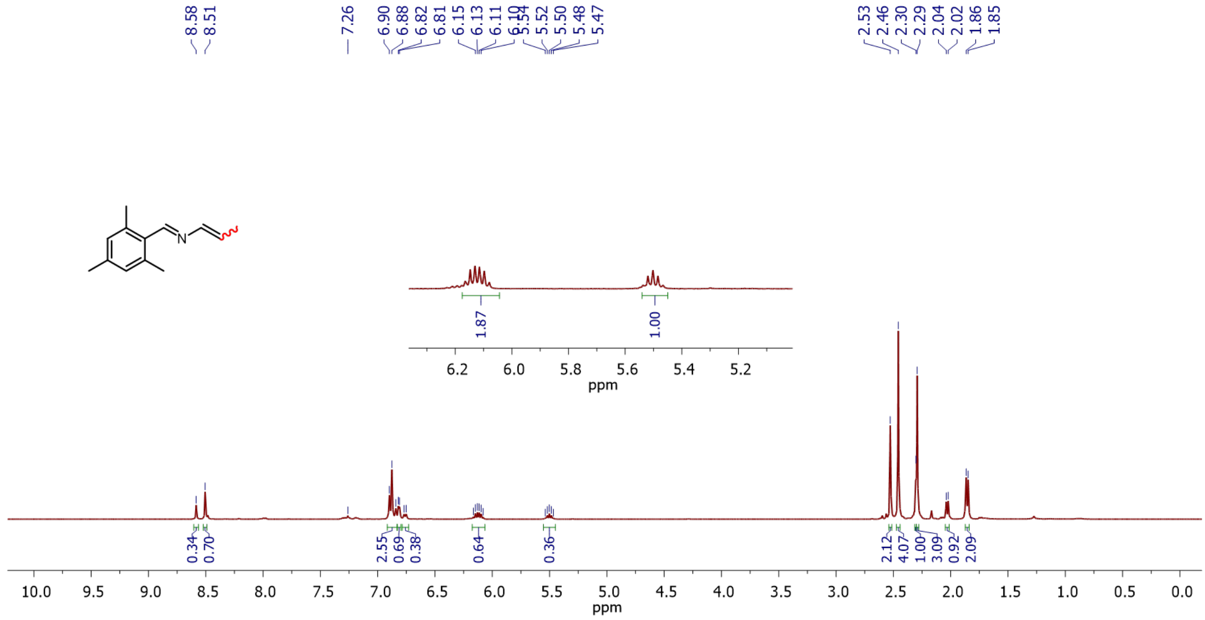

Figure S103. ^{1}H NMR spectrum (400 MHz) of **7j** in $CDCl_3$.

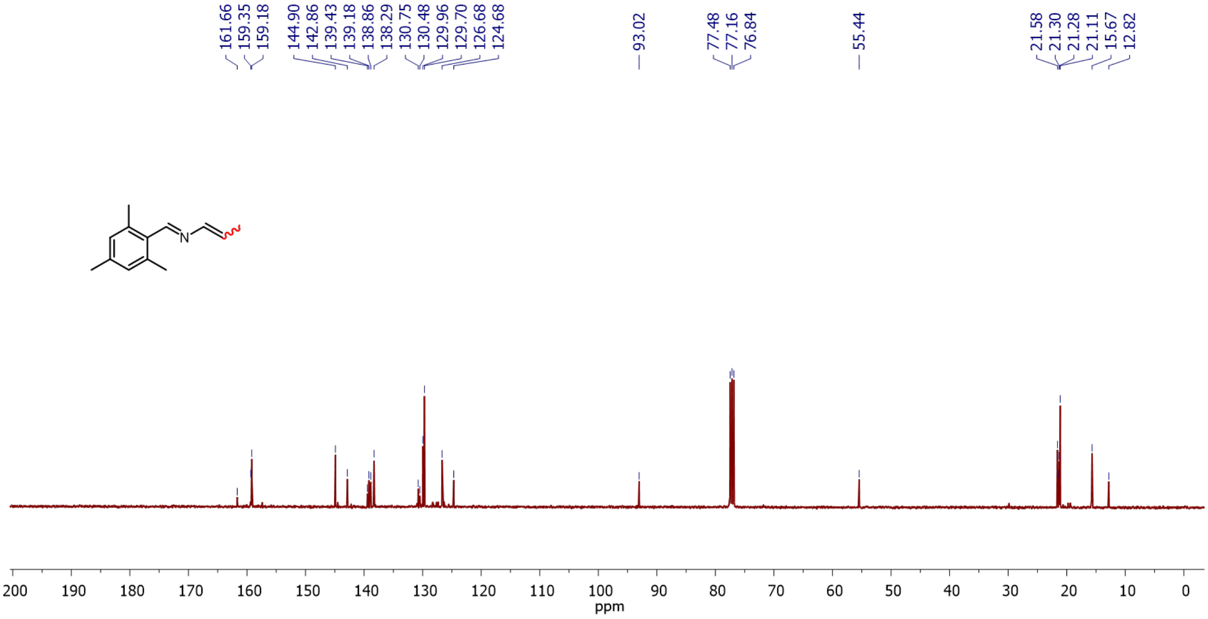

Figure S104. ^{13}C {^{1}H} NMR spectrum (101 MHz) of **7j** in $CDCl_3$.

Compound Spectrum SmartFormula Report

**Analysis Info**		Acquisition Date	10/12/2023 11:29:43	
Analysis Name	D:\Data\Graham\Dr_5228000001.d			
Method	APCI_pos_SolidProbe.m	Operator	Larisa Panz	
Sample Name	SRK 7j	Instrument	maXis impact	282001.00128
Comment				

Acquisition Parameter

Source Type	APCI	Ion Polarity	Positive	Set Nebulizer	1.2 Bar
Focus	Active	Set Capillary	3000 V	Set Dry Heater	120 °C
Scan Begin	50 m/z	Set End Plate Offset	-500 V	Set Dry Gas	1.5 l/min
Scan End	2000 m/z	Set Charging Voltage	2000 V	Set Divert Valve	Source
		Set Corona	5000 nA	Set APCI Heater	200 °C

+MS, 0.8-0.9min #49-52

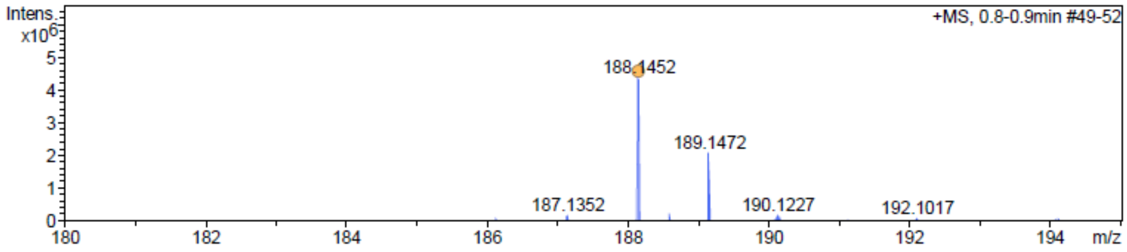

Meas. m/z	#	Ion Formula	m/z	err [ppm]	mSigma	# mSigma	Score	rdb	e⁻ Conf	N-Rule	|err| [mDa]
188.1452	1	C13H18N	188.1439	-6.5	190.9	1	100.00	5.5	even	ok	1.2
	2	C9H18NO3	188.1287	-87.6	215.0	2	0.00	1.5	even	ok	16.5
	1	C13H18N	188.1439	-6.5	190.9	1	100.00	5.5	even	ok	1.2
	2	C9H18NO3	188.1287	-87.6	215.0	2	0.00	1.5	even	ok	16.5

Figure S105. High resolution mass spectrum of **7j.**

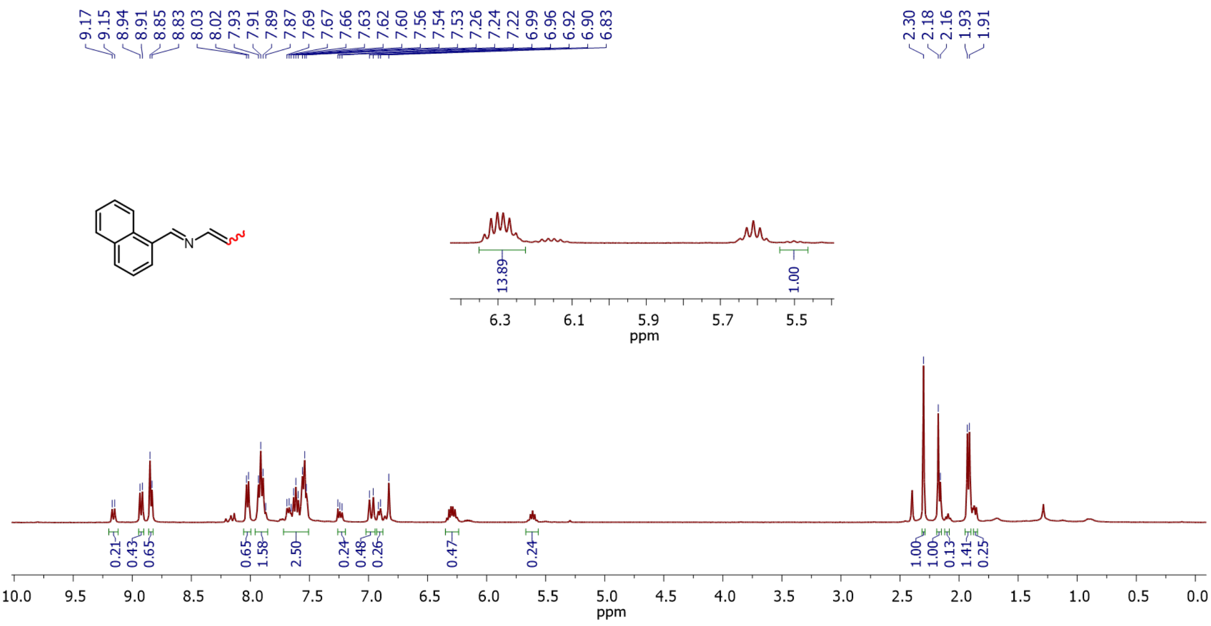

Figure S106. ^{1}H NMR spectrum (400 MHz) of **7k** in $CDCl_3$.

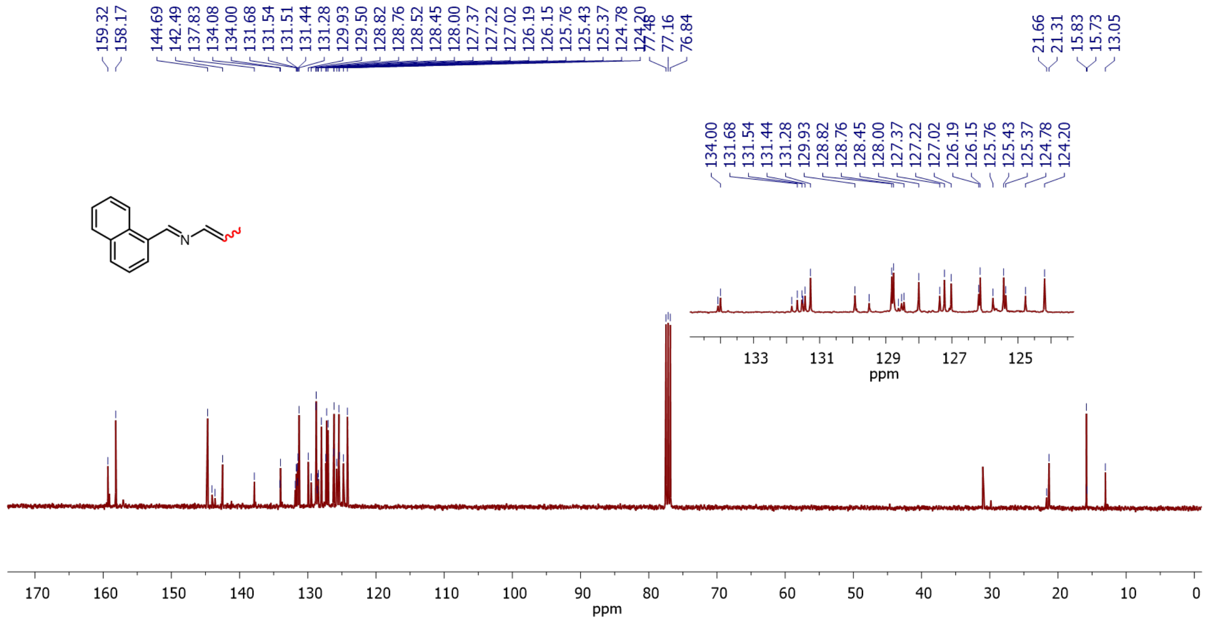

Figure S107. ^{13}C {^{1}H} NMR spectrum (101 MHz) of **7k** in $CDCl_3$.

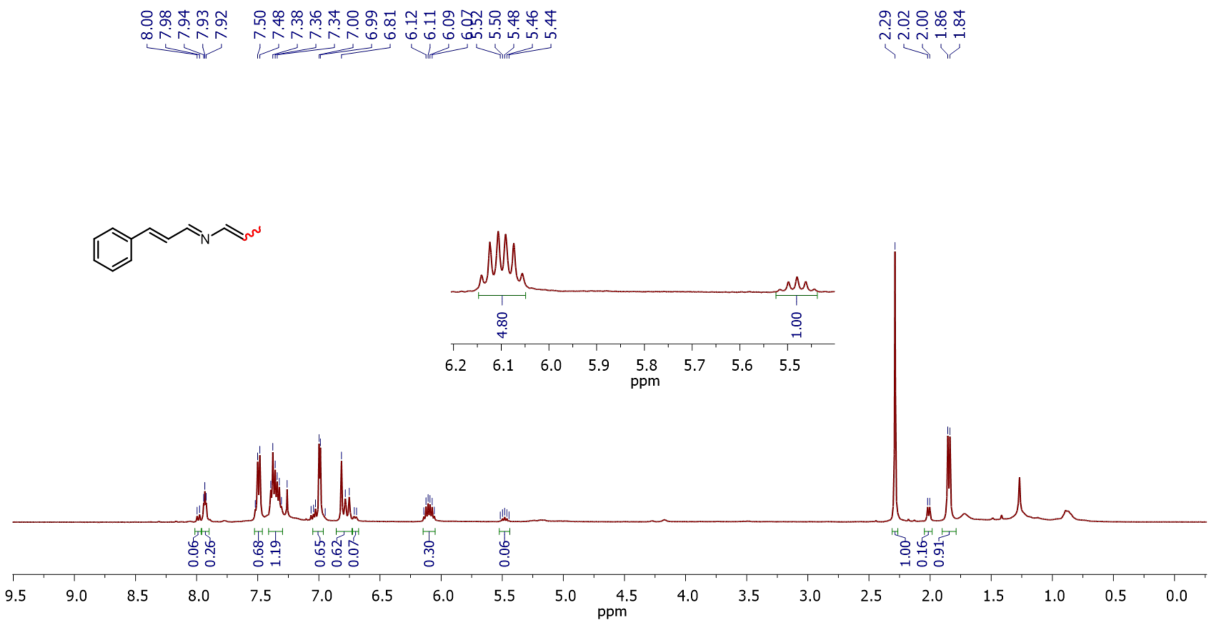

Figure S108. ^{1}H NMR spectrum (400 MHz) of **7l** in $CDCl_3$.

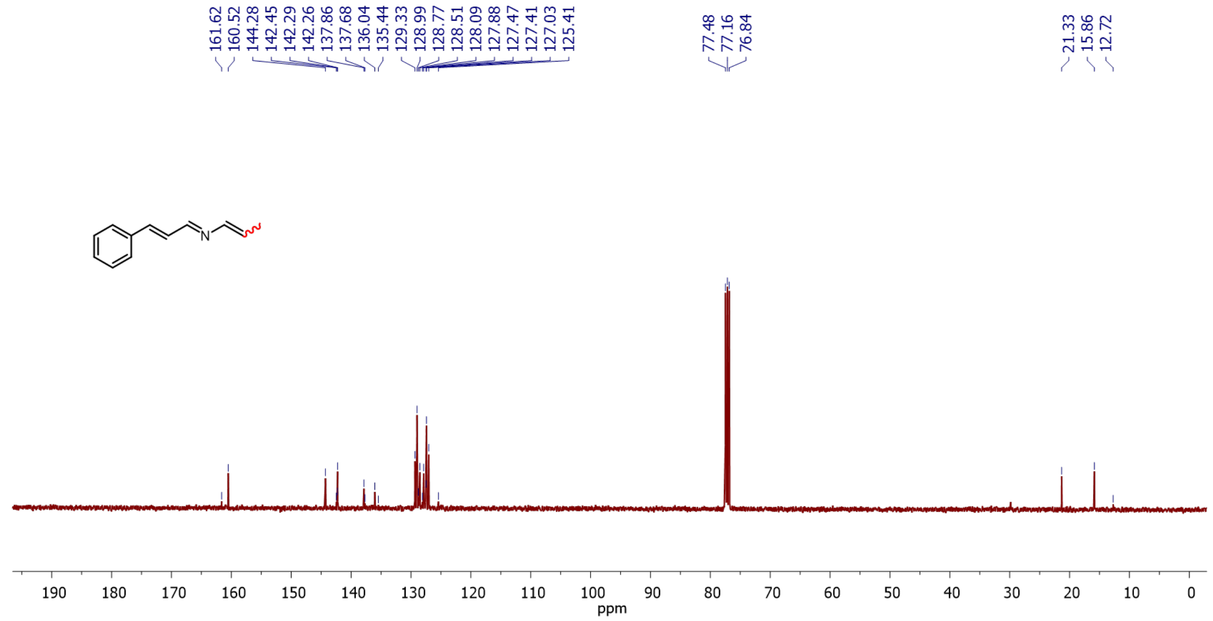

Figure S109. ^{13}C {^{1}H} NMR spectrum (101 MHz) of **7l** in $CDCl_3$.

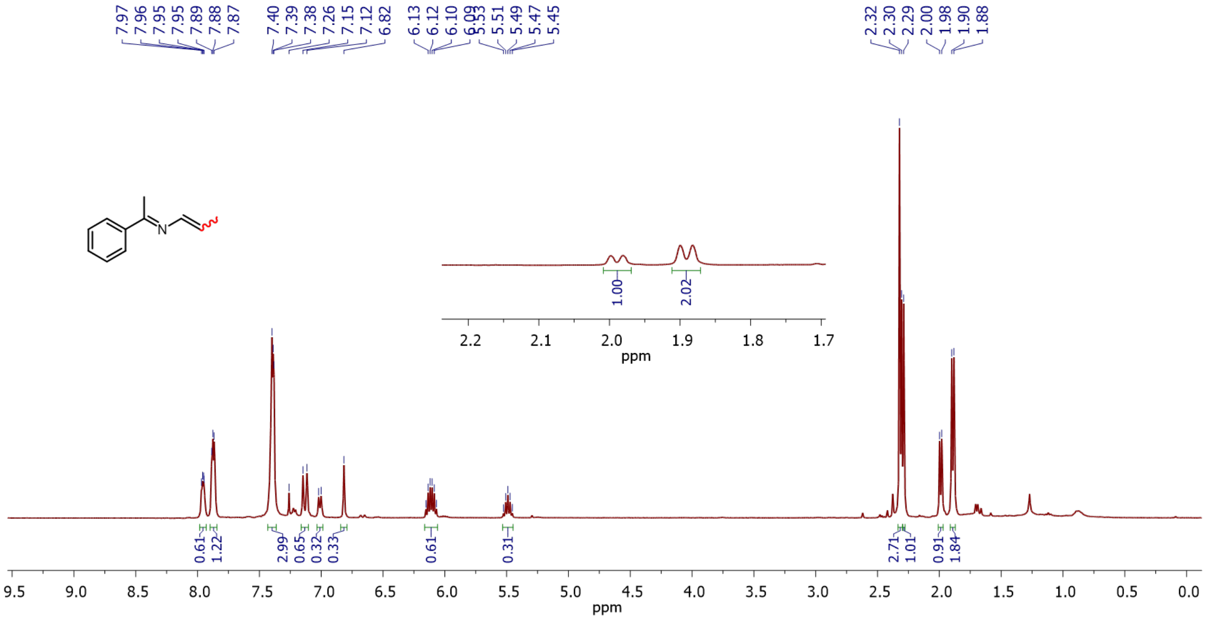

Figure S110. ^{1}H NMR spectrum (400 MHz) of **8a** in $CDCl_3$.

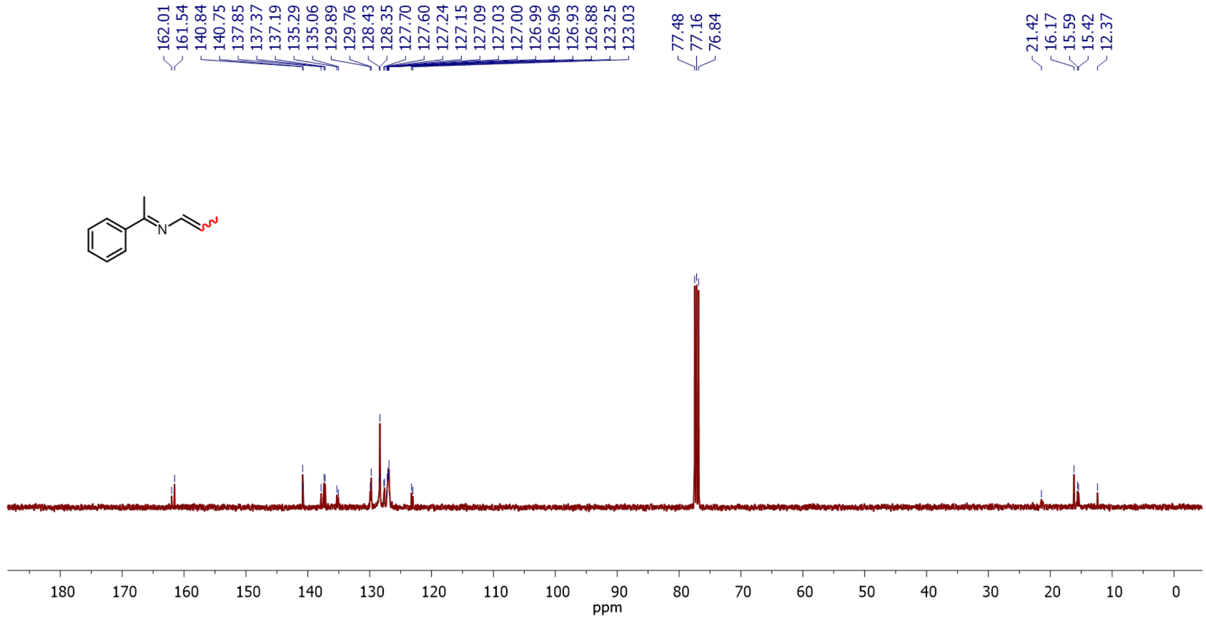

Figure S111. ^{13}C {^{1}H} NMR spectrum (101 MHz) of **8a** in $CDCl_3$.

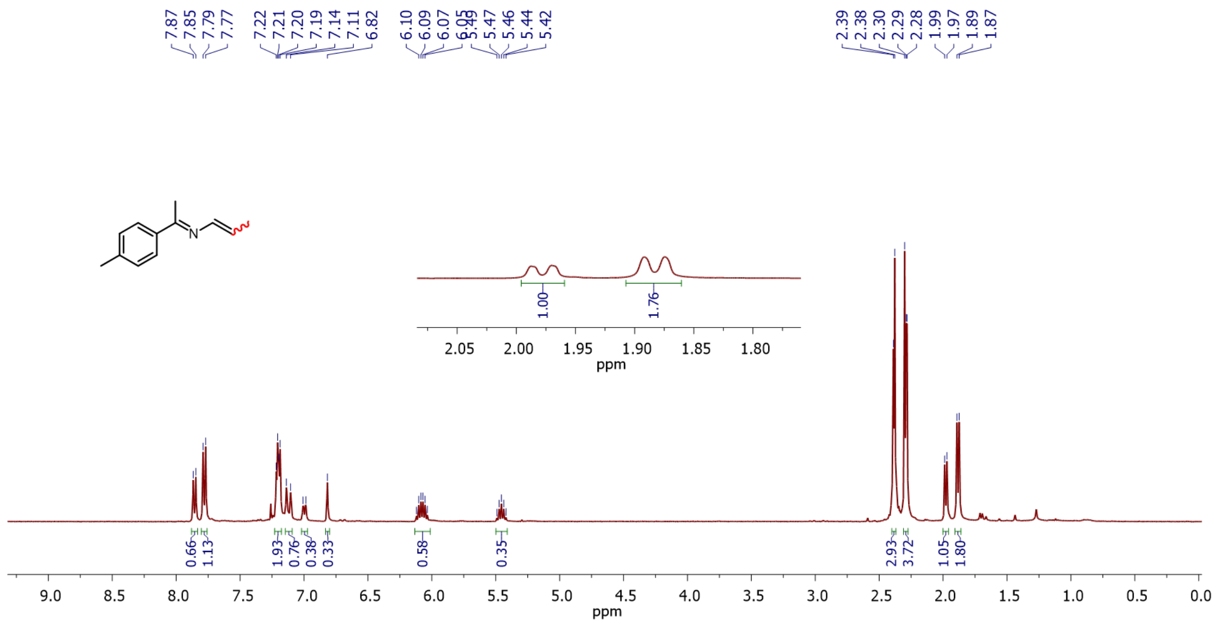

Figure S112. ^{1}H NMR spectrum (400 MHz) of **8b** in $CDCl_3$.

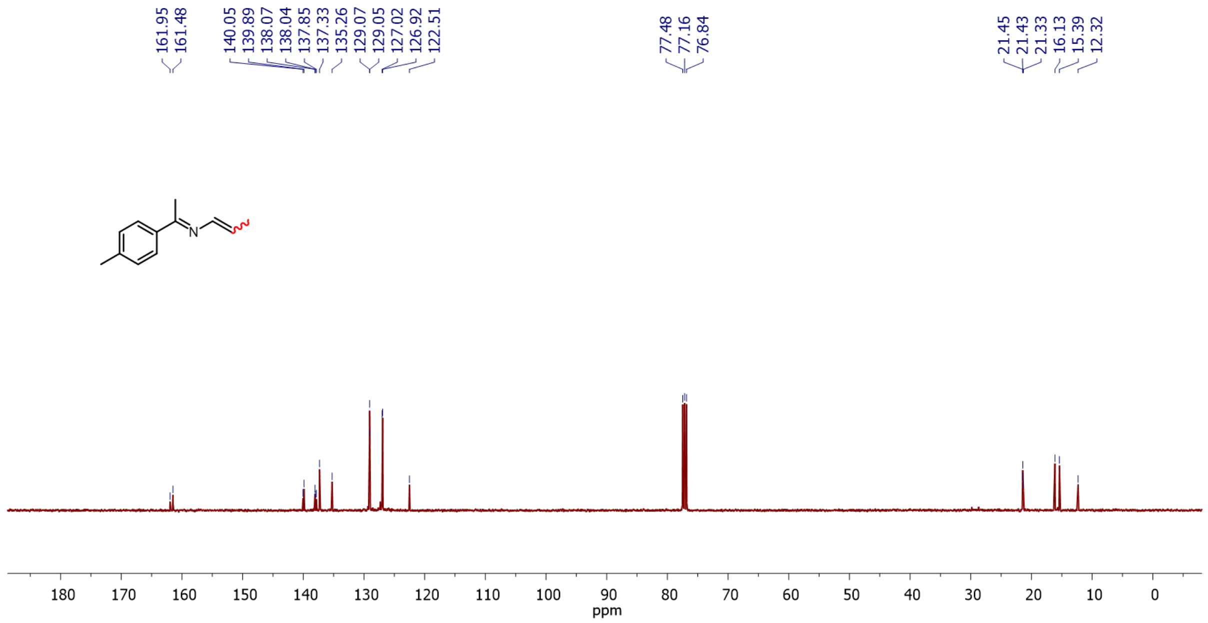

Figure S113. ^{13}C {^{1}H} NMR spectrum (101 MHz) of **8b** in $CDCl_3$.

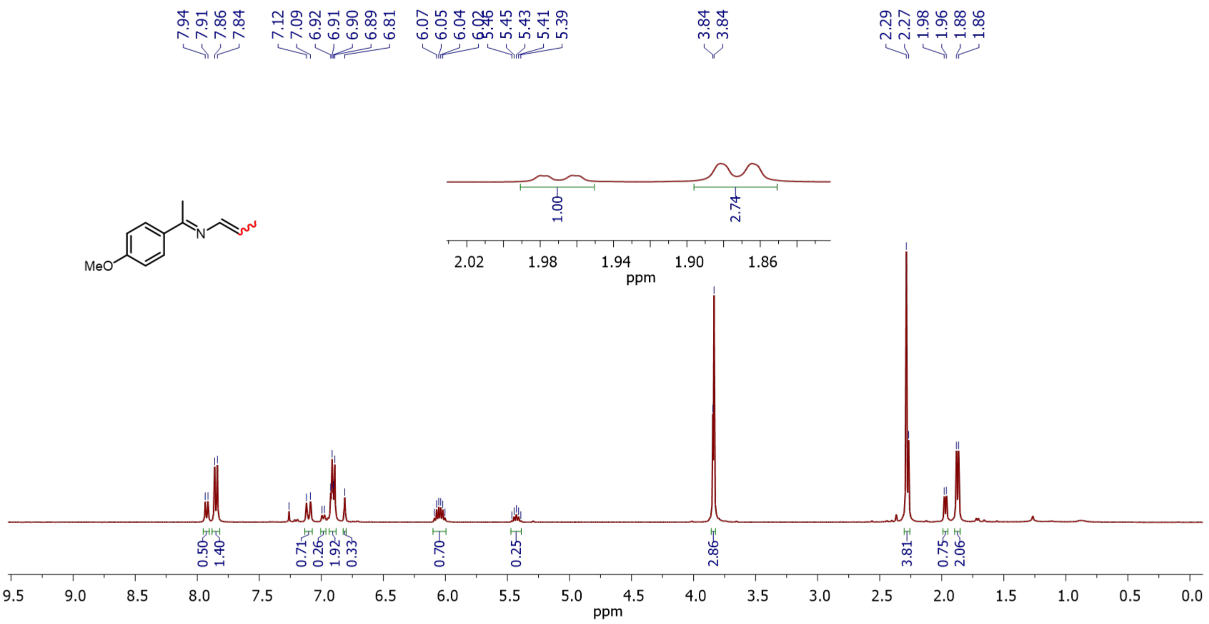

Figure S114. ^{1}H NMR spectrum (400 MHz) of **8c** in $CDCl_3$.

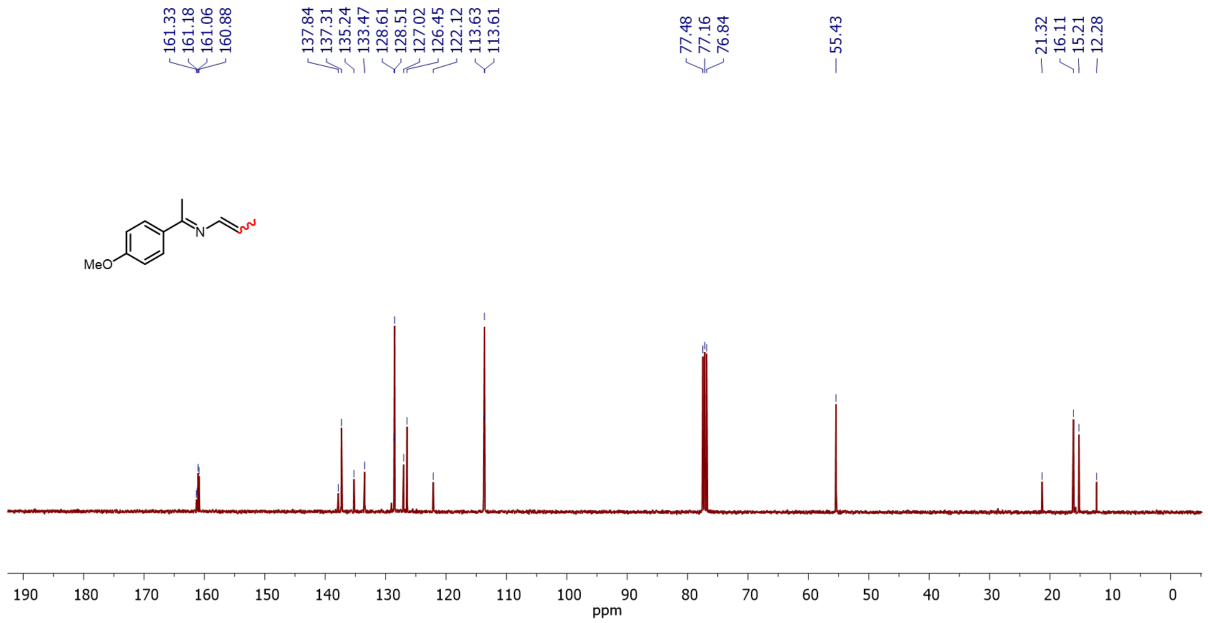

Figure S115. ^{13}C {^{1}H} NMR spectrum (101 MHz) of **8c** in $CDCl_3$.

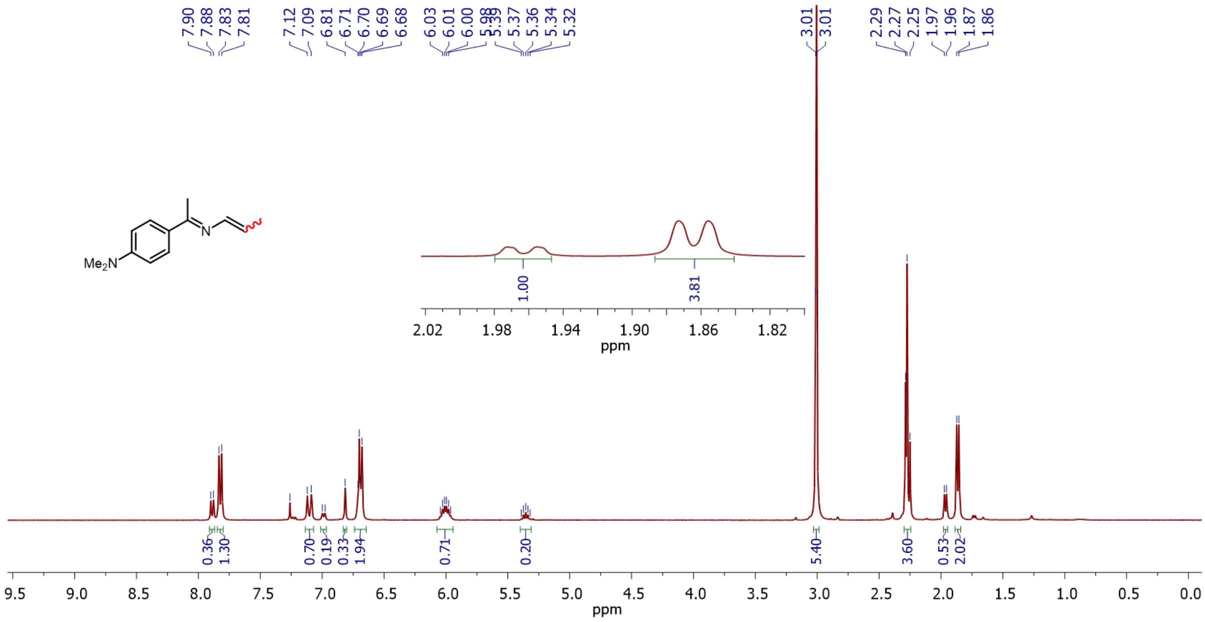

Figure S116. ^{1}H NMR spectrum (400 MHz) of **8d** in $CDCl_3$.

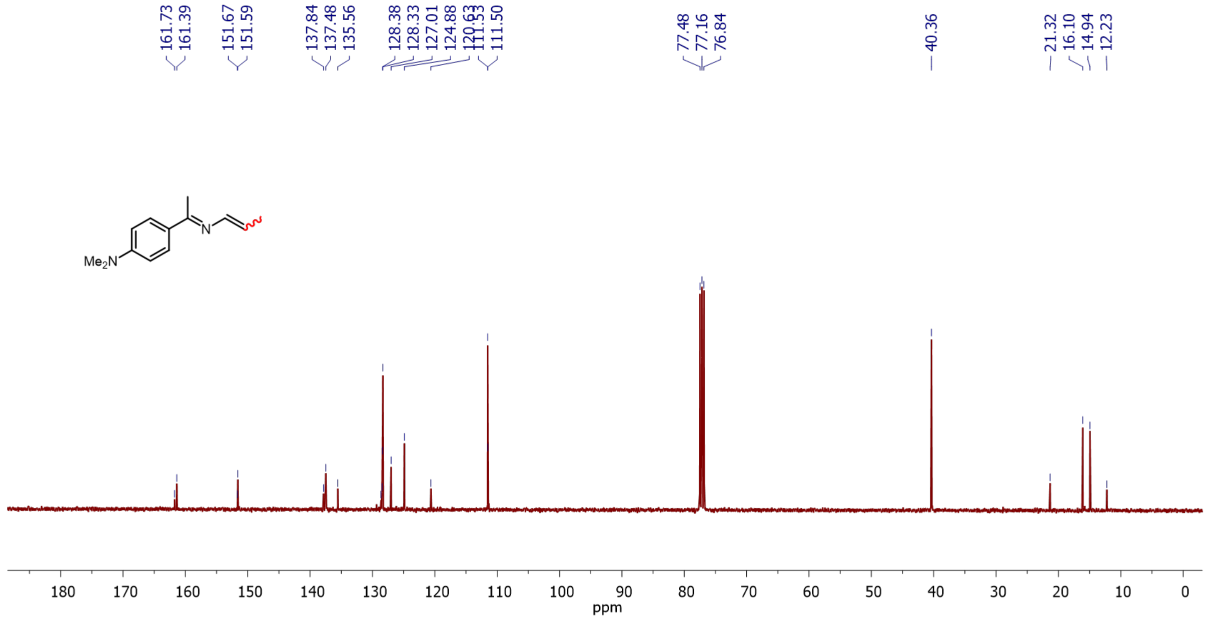

Figure S117. ^{13}C {^{1}H} NMR spectrum (101 MHz) of **8d** in $CDCl_3$.

Compound Spectrum SmartFormula Report

Analysis Info

Analysis Name	D:\Data\Graham\Dr_5229000001.d	Acquisition Date	10/12/2023 11:32:32
Method	APCI_pos_SolidProbe.m	Operator	Larisa Panz
Sample Name	SRK 8d	Instrument	maXis impact 282001.00128
Comment			

Acquisition Parameter

Source Type	APCI	Ion Polarity	Positive	Set Nebulizer	1.2 Bar
Focus	Active	Set Capillary	3000 V	Set Dry Heater	120 °C
Scan Begin	50 m/z	Set End Plate Offset	-500 V	Set Dry Gas	1.5 l/min
Scan End	2000 m/z	Set Charging Voltage	2000 V	Set Divert Valve	Source
		Set Corona	5000 nA	Set APCI Heater	200 °C

+MS, 0.3-0.3min #17-20

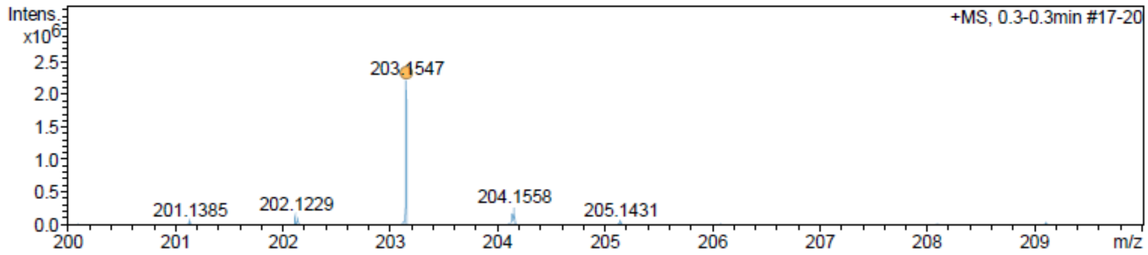

Meas. m/z	#	Ion Formula	m/z	err [ppm]	mSigma	# mSigma	Score	rdb	e¯ Conf	N-Rule	|err| [mDa]
203.1547	1	C9H19N2O3	203.1396	-74.5	13.5	1	0.00	1.5	even	ok	15.1
	2	C13H19N2	203.1548	0.6	19.1	2	100.00	5.5	even	ok	0.1
	1	C9H19N2O3	203.1396	-74.5	13.5	1	0.00	1.5	even	ok	15.1
	2	C13H19N2	203.1548	0.6	19.1	2	100.00	5.5	even	ok	0.1

Figure S118. High resolution mass spectrum of **8d.**

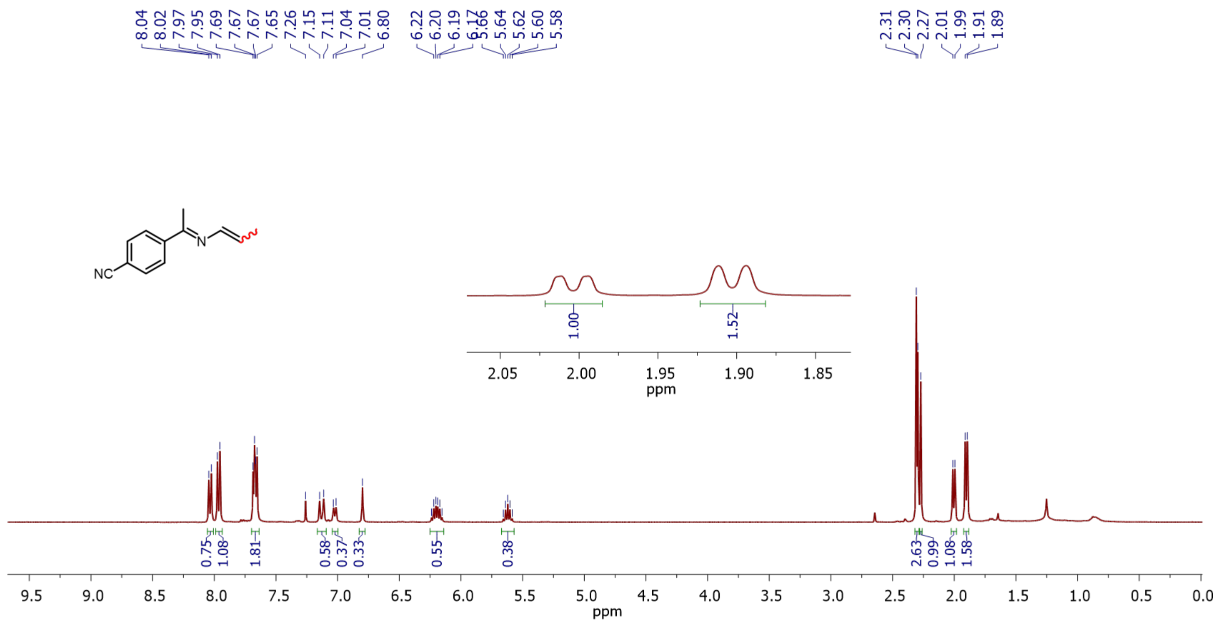

Figure S119. ^{1}H NMR spectrum (400 MHz) of **8e** in $CDCl_3$.

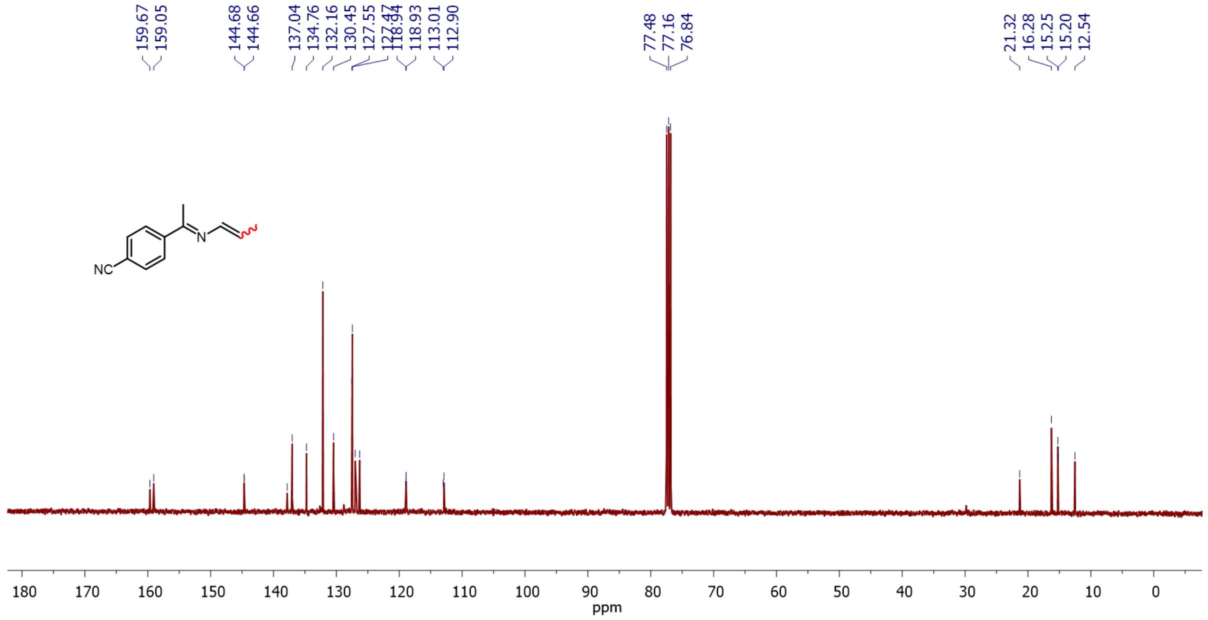

Figure S120. ^{13}C {^{1}H} NMR spectrum (101 MHz) of **8e** in $CDCl_3$.

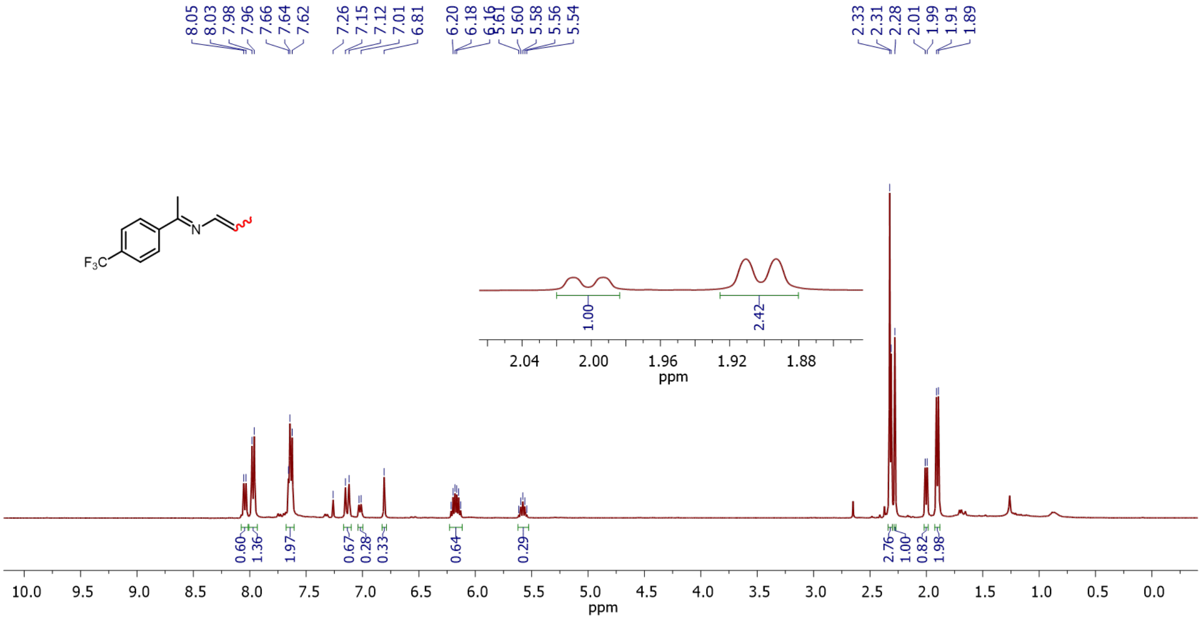

Figure S121. ^{1}H NMR spectrum (400 MHz) of **8f** in $CDCl_3$.

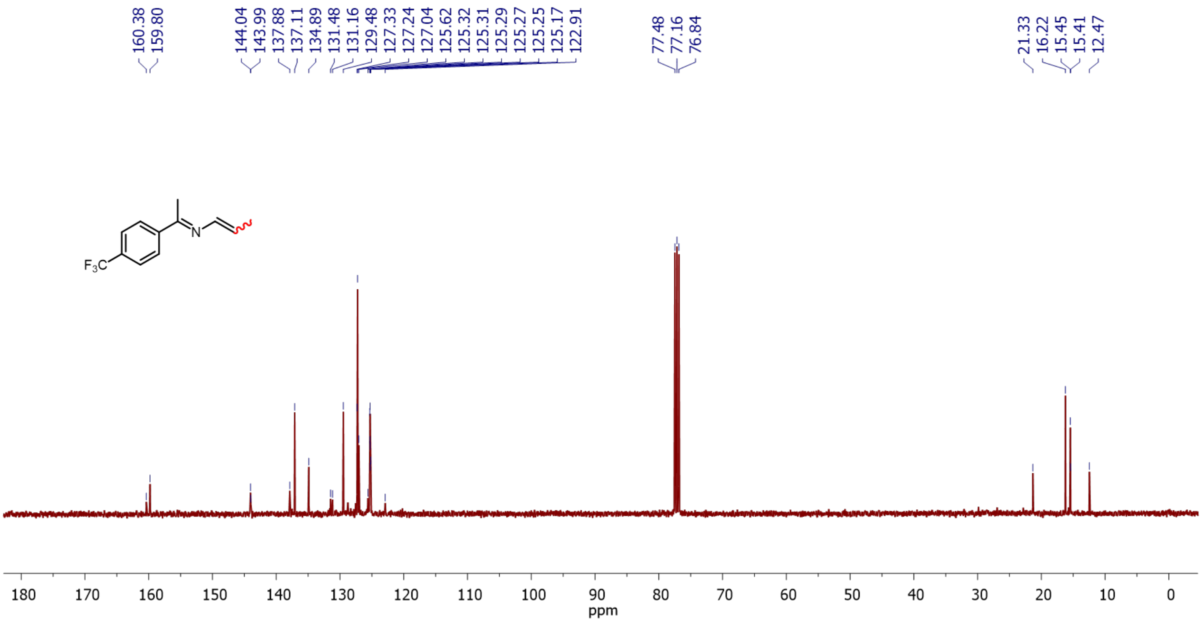

Figure S122. ^{13}C {^{1}H} NMR spectrum (101 MHz) of **8f** in $CDCl_3$.

Compound Spectrum SmartFormula Report

**Analysis Info**		Acquisition Date	10/12/2023 11:35:08
Analysis Name	D:\Data\Graham\Dr_5230000001.d		
Method	APCI_pos_SolidProbe.m	Operator	Larisa Panz
Sample Name	SRK 8f	Instrument	maXis impact 282001.00128
Comment			

Acquisition Parameter

Source Type	APCI	Ion Polarity	Positive	Set Nebulizer	1.2 Bar
Focus	Active	Set Capillary	3000 V	Set Dry Heater	120 °C
Scan Begin	50 m/z	Set End Plate Offset	-500 V	Set Dry Gas	1.5 l/min
Scan End	2000 m/z	Set Charging Voltage	2000 V	Set Divert Valve	Source
		Set Corona	5000 nA	Set APCI Heater	200 °C

+MS, 0.6-0.6min #33-35

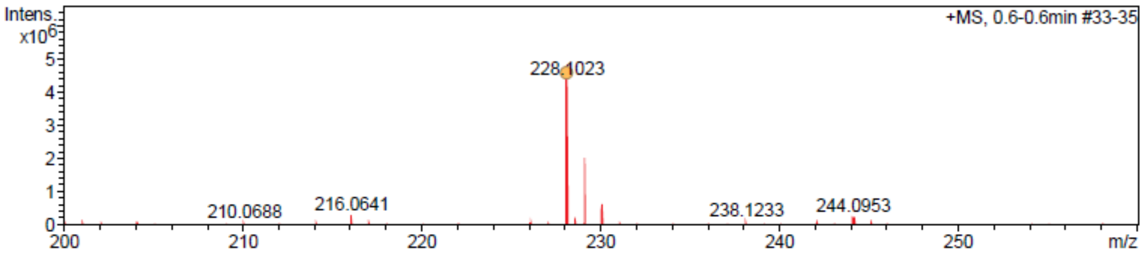

Meas. m/z	#	Ion Formula	m/z	err [ppm]	mSigma	# mSigma	Score	rdb	e⁻ Conf	N-Rule	|err| [mDa]
228.1023	1	C12H13F3N	228.1000	-9.9	206.4	1	100.00	5.5	even	ok	2.3
	2	C8H13F3NO3	228.0848	-76.8	228.7	2	0.00	1.5	even	ok	17.5
	1	C12H13F3N	228.1000	-9.9	206.4	1	100.00	5.5	even	ok	2.3
	2	C8H13F3NO3	228.0848	-76.8	228.7	2	0.00	1.5	even	ok	17.5

Figure S123. High resolution mass spectrum of **8f.**

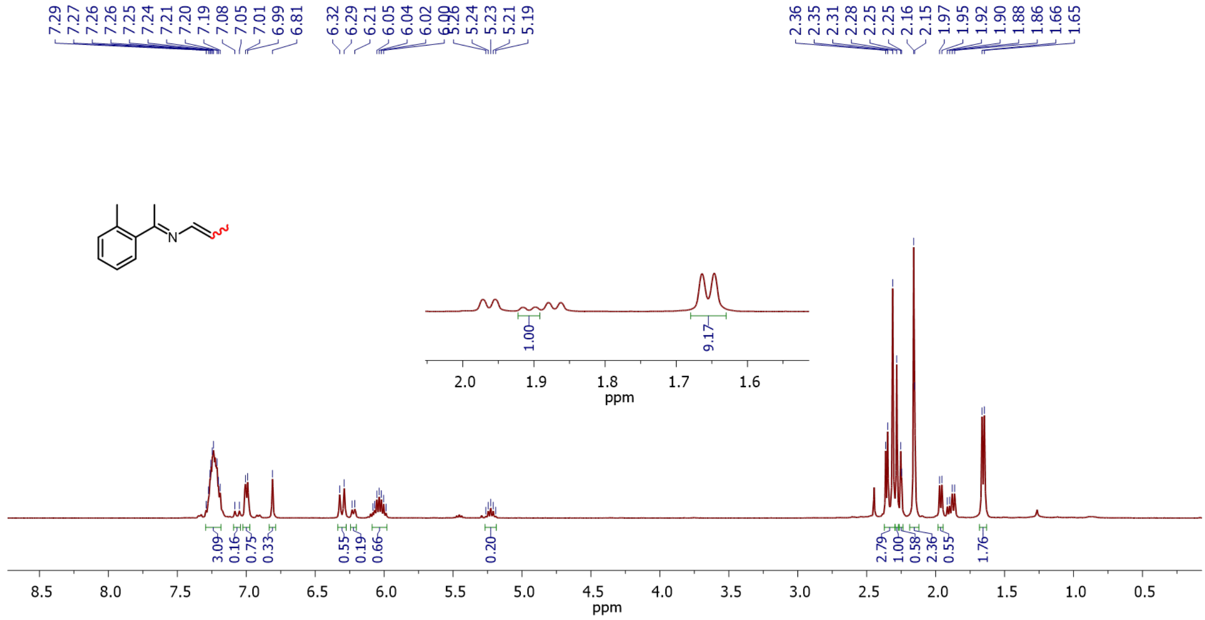

Figure S124. ^{1}H NMR spectrum (400 MHz) of **8g** in $CDCl_3$.

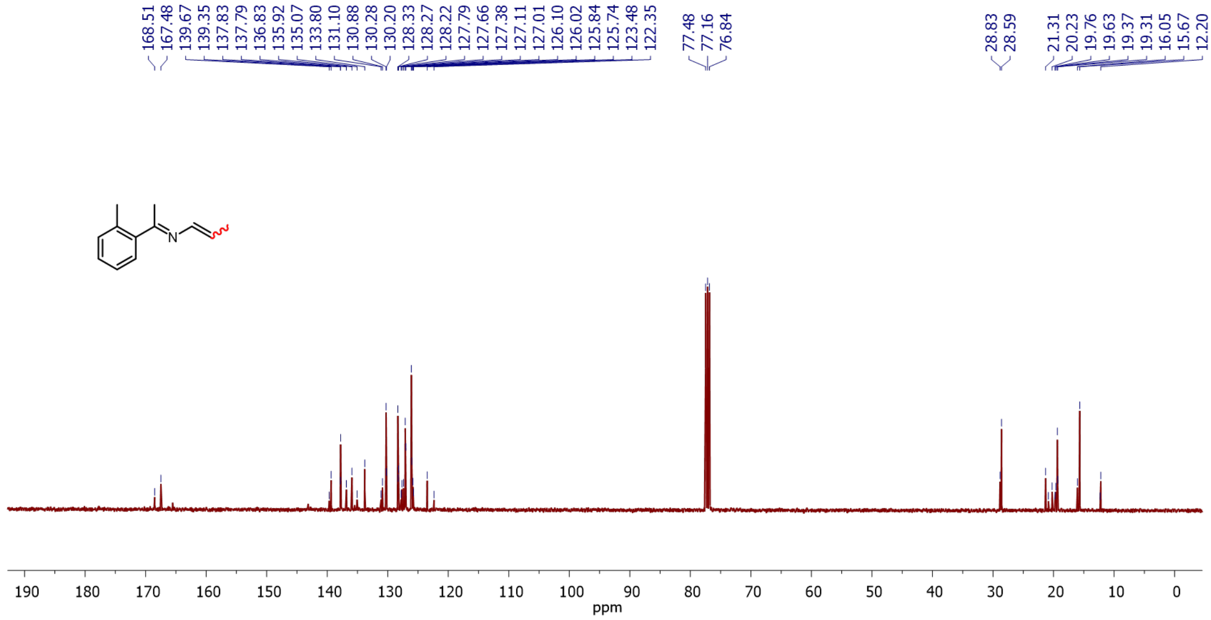

Figure S125. ^{13}C {^{1}H} NMR spectrum (101 MHz) of **8g** in $CDCl_3$.

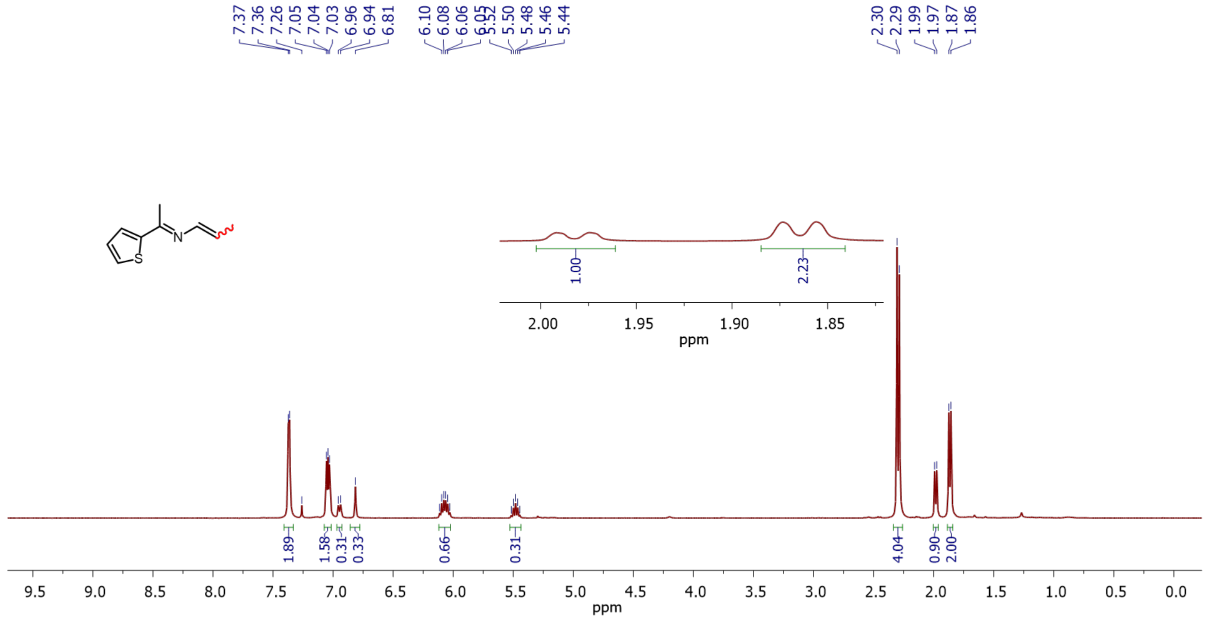

Figure S126. ^{1}H NMR spectrum (400 MHz) of **8h** in $CDCl_3$.

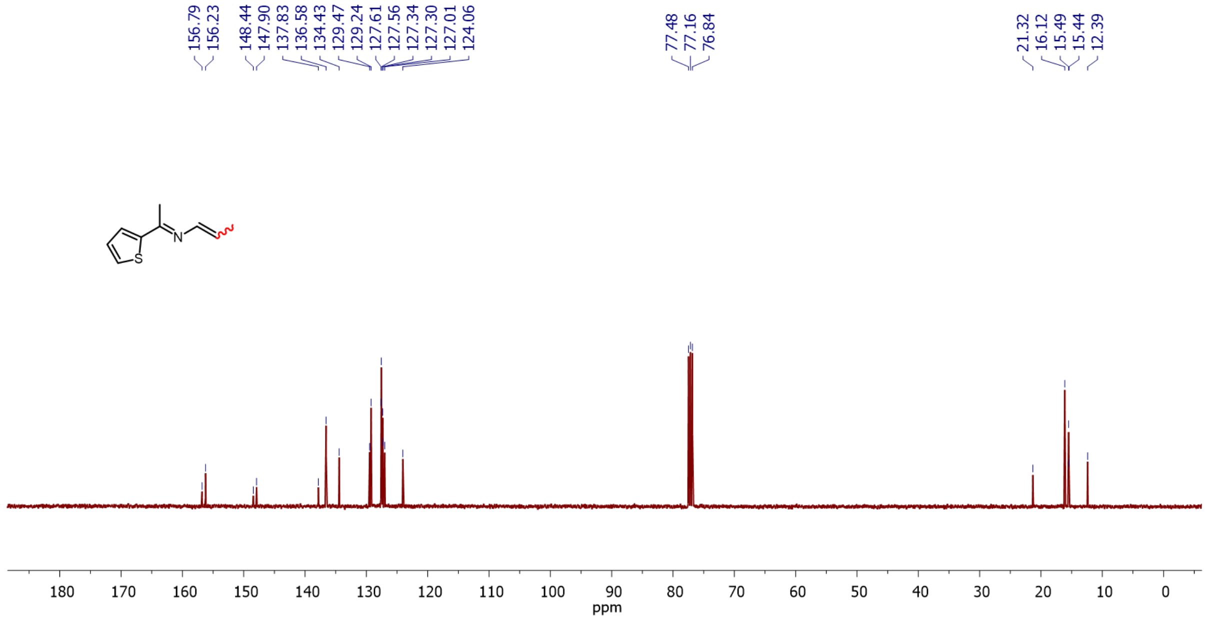

Figure S127. ^{13}C {^{1}H} NMR spectrum (101 MHz) of **8h** in $CDCl_3$.

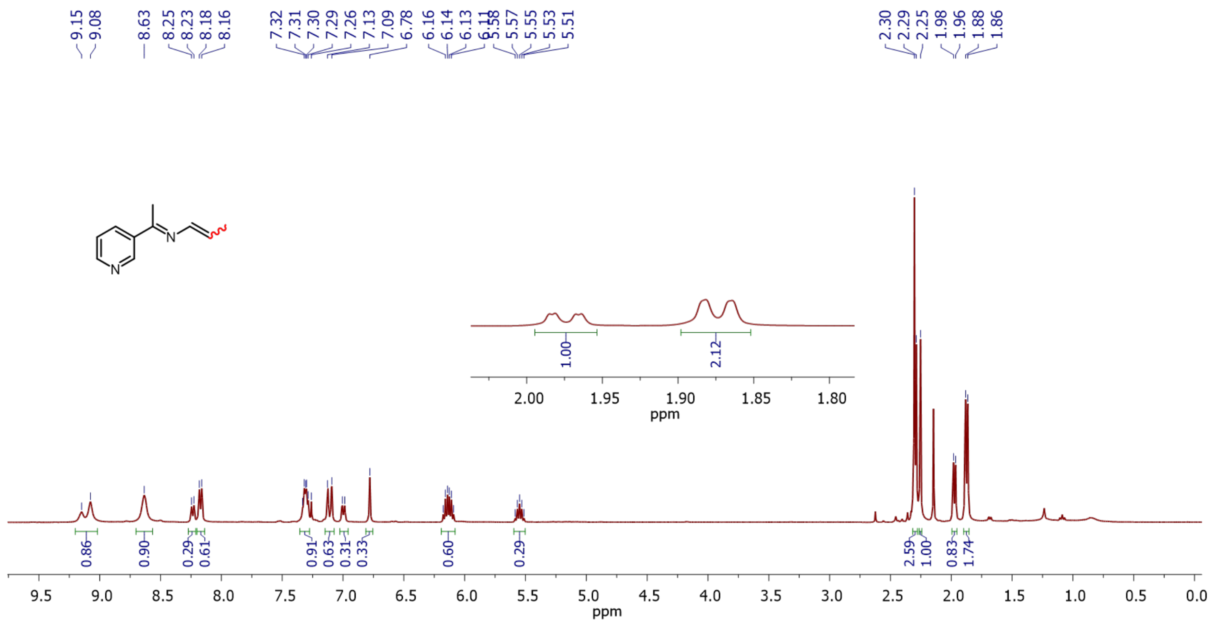

Figure S128. ^{1}H NMR spectrum (400 MHz) of **8i** in $CDCl_3$.

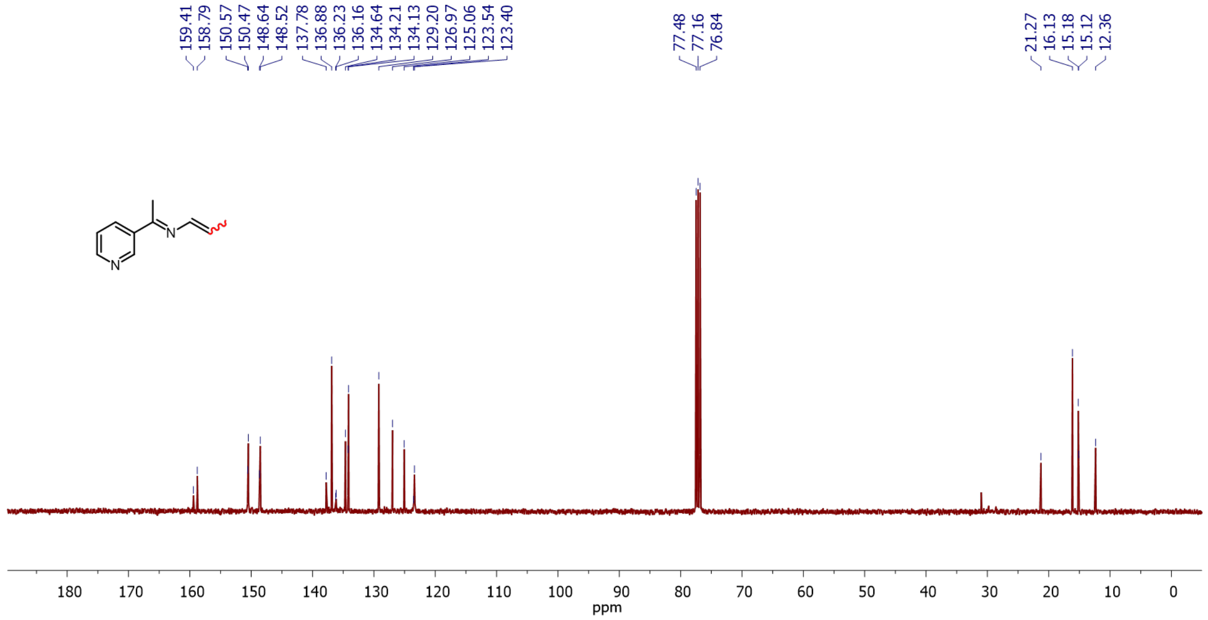

Figure S129. ^{13}C {^{1}H} NMR spectrum (101 MHz) of **8i** in $CDCl_3$.

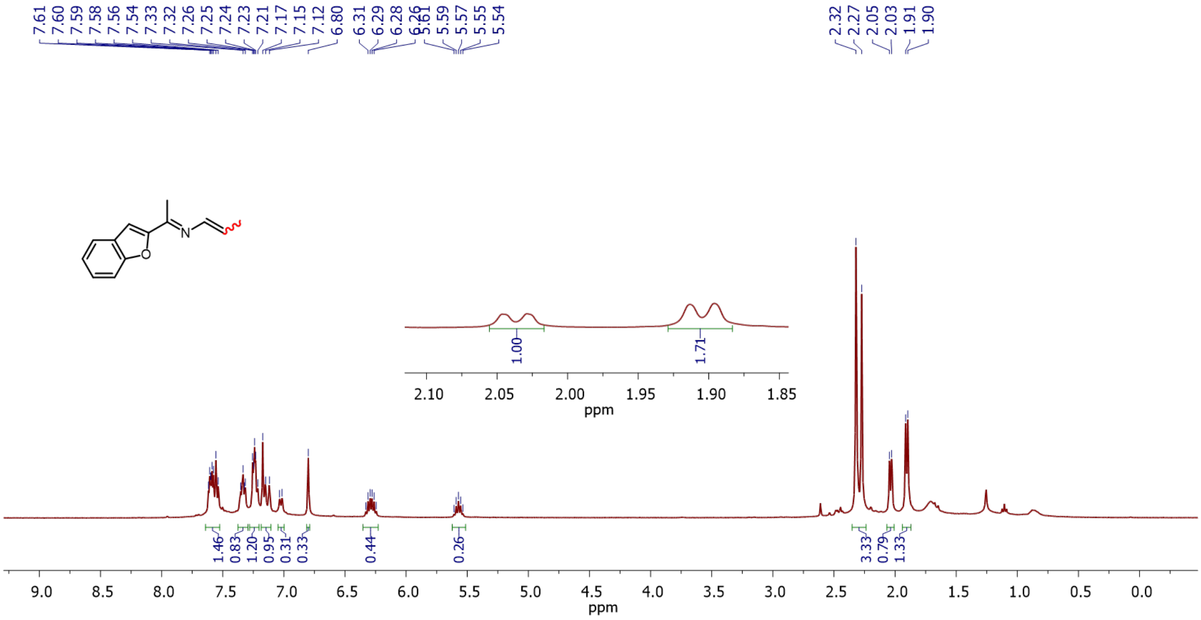

Figure S130. ^{1}H NMR spectrum (400 MHz) of **8j** in $CDCl_3$.

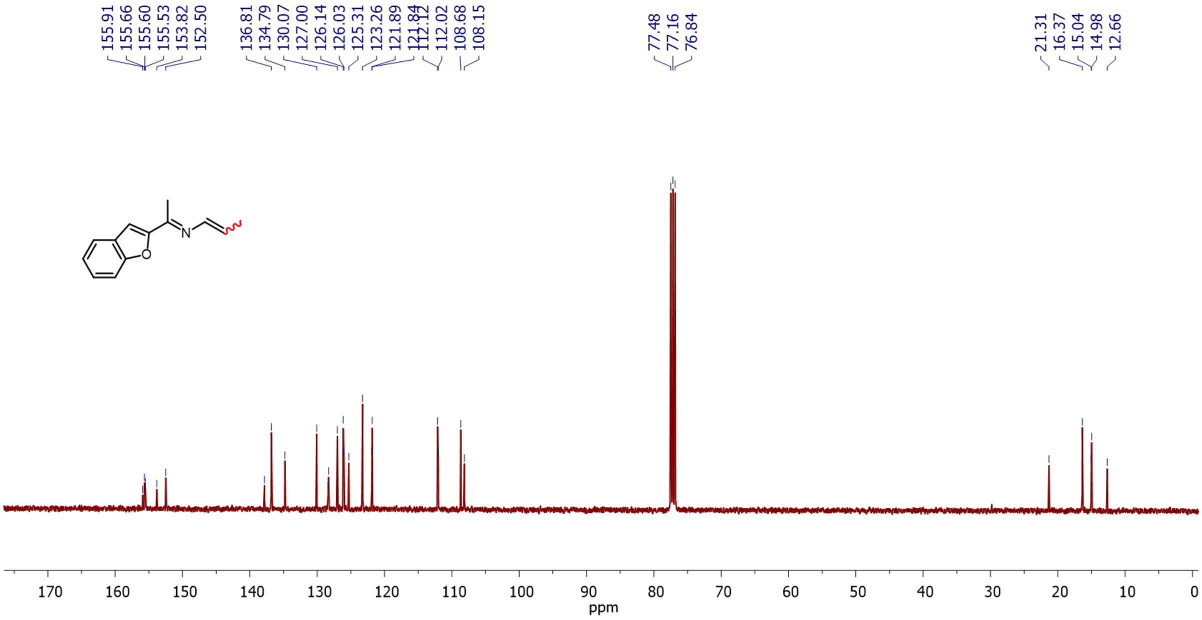

Figure S131. $^{13}C\{^{1}H\}$ NMR spectrum (101 MHz) of **8j** in $CDCl_3$.

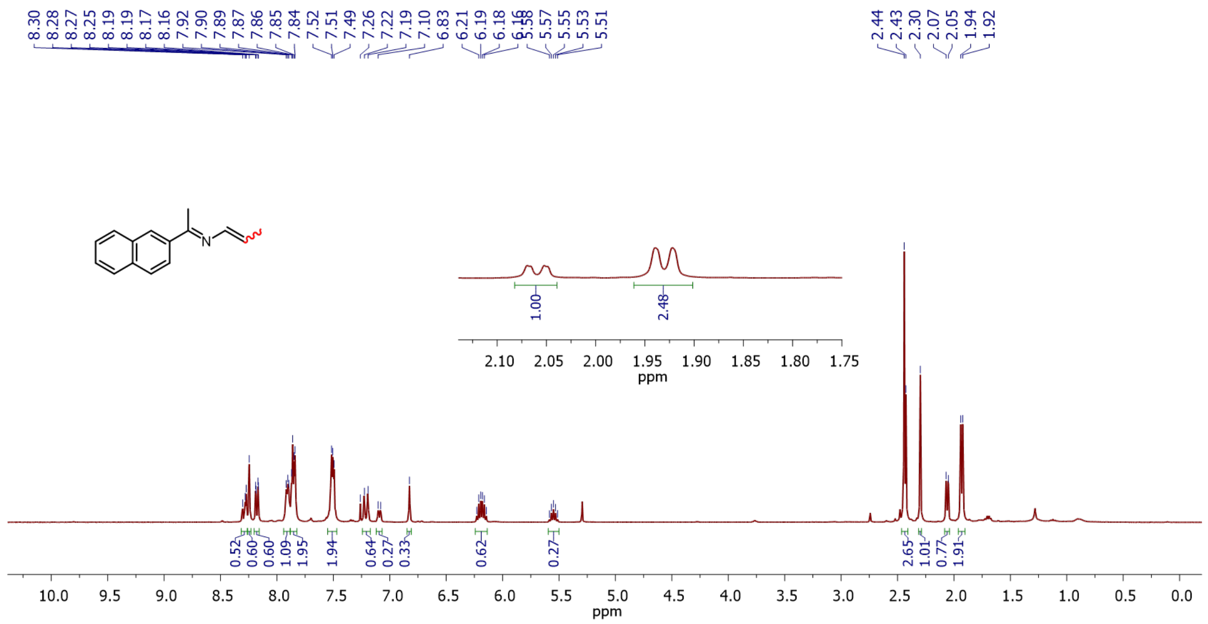

Figure S132. ^{1}H NMR spectrum (400 MHz) of **8k** in $CDCl_3$.

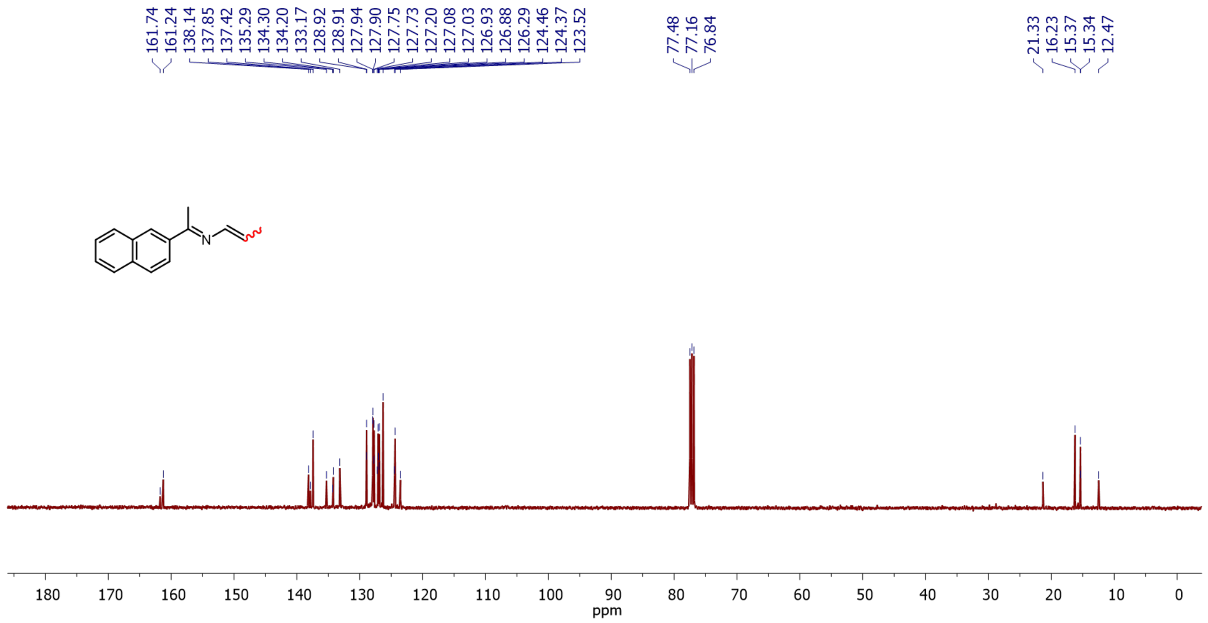

Figure S133. ^{13}C {^{1}H} NMR spectrum (101 MHz) of **8k** in $CDCl_3$.

Compound Spectrum SmartFormula Report

Analysis Info

Analysis Name	D:\Data\Graham\Dr_5232000002.d	Acquisition Date	10/12/2023 11:42:10
Method	APCI_pos_SolidProbe.m	Operator	Larisa Panz
Sample Name	SRK 8K	Instrument	maXis impact 282001.00128
Comment			

Acquisition Parameter

Source Type	APCI	Ion Polarity	Positive	Set Nebulizer	1.2 Bar
Focus	Active	Set Capillary	3000 V	Set Dry Heater	120 °C
Scan Begin	50 m/z	Set End Plate Offset	-500 V	Set Dry Gas	1.5 l/min
Scan End	2000 m/z	Set Charging Voltage	2000 V	Set Divert Valve	Source
		Set Corona	5000 nA	Set APCI Heater	200 °C

+MS, 0.1-0.2min #6-11

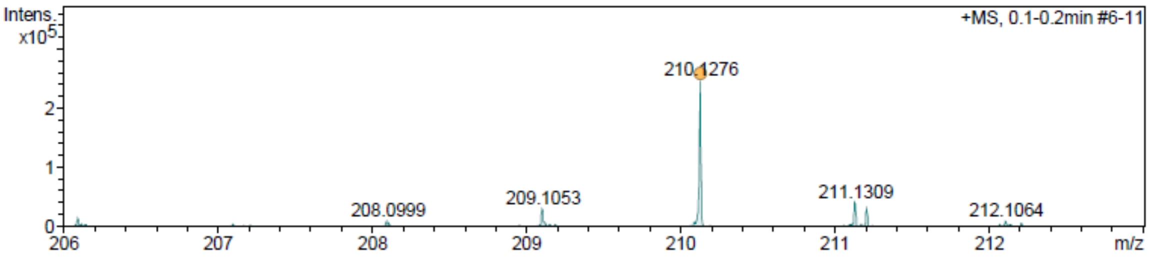

Meas. m/z	#	Ion Formula	m/z	err [ppm]	mSigma	# mSigma	Score	rdb	e⁻ Conf	N-Rule	|err| [mDa]
210.1276	1	C15H16N	210.1283	3.0	2.9	1	100.00	8.5	even	ok	0.6
	2	C11H16NO3	210.1130	-69.6	23.4	2	0.00	4.5	even	ok	14.6
	1	C15H16N	210.1283	3.0	2.9	1	100.00	8.5	even	ok	0.6
	2	C11H16NO3	210.1130	-69.6	23.4	2	0.00	4.5	even	ok	14.6

Figure S134. High resolution mass spectrum of **8k.**

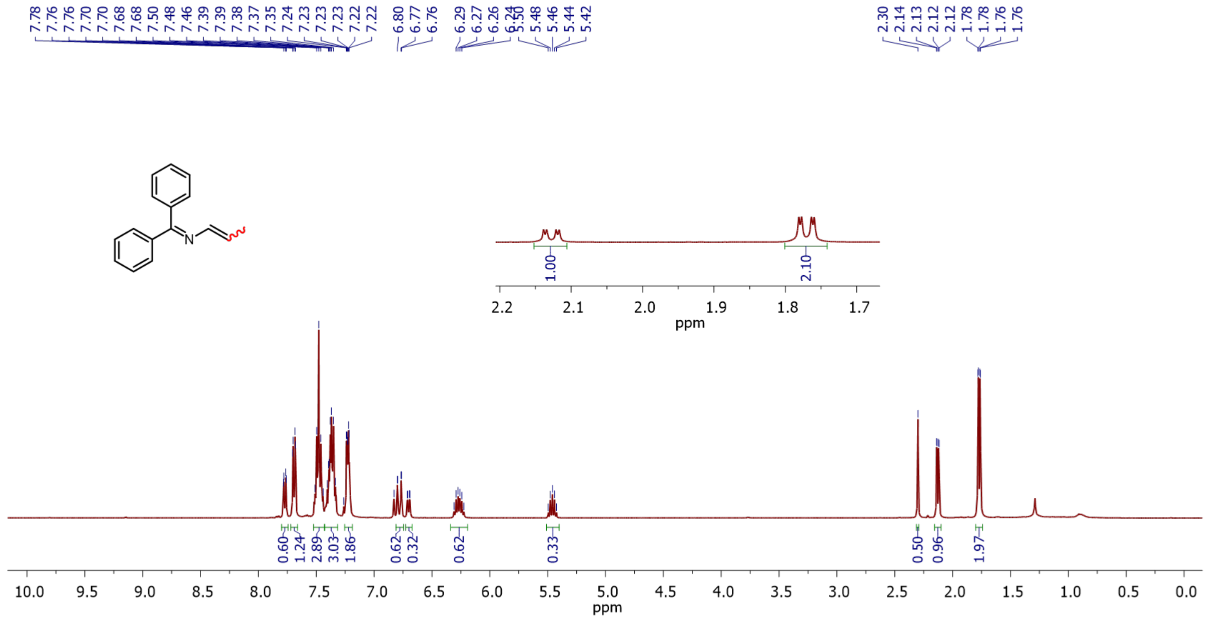

Figure S135. ^{1}H NMR spectrum (400 MHz) of **8l** in $CDCl_3$.

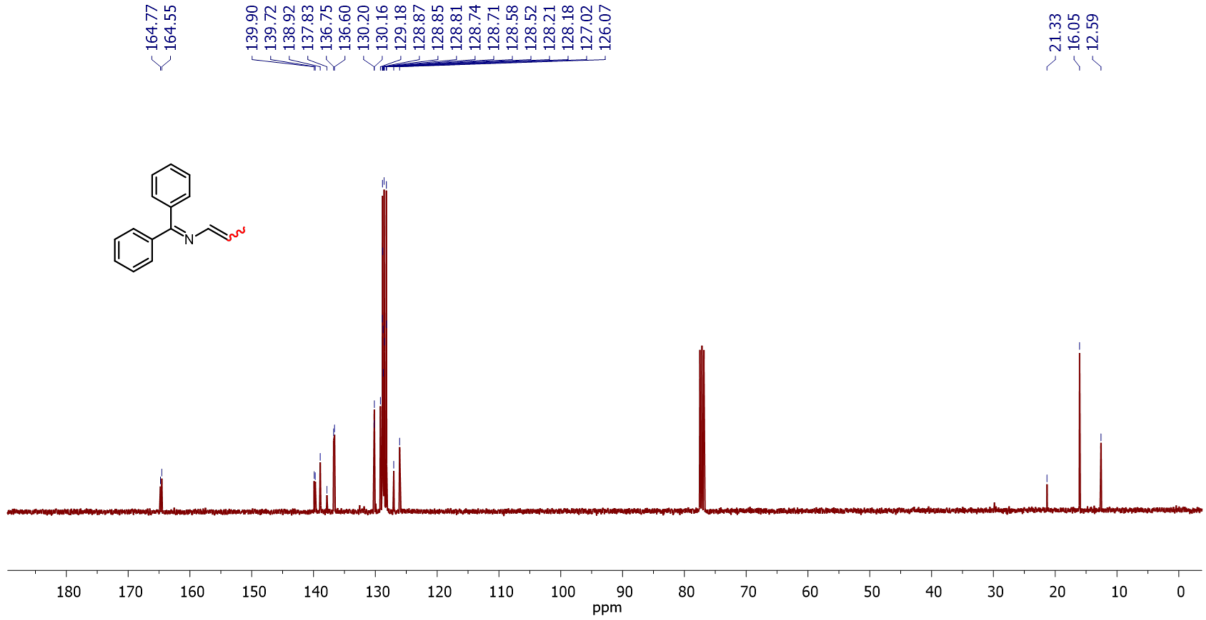

Figure S136. ^{13}C {^{1}H} NMR spectrum (101 MHz) of **8l** in $CDCl_3$.

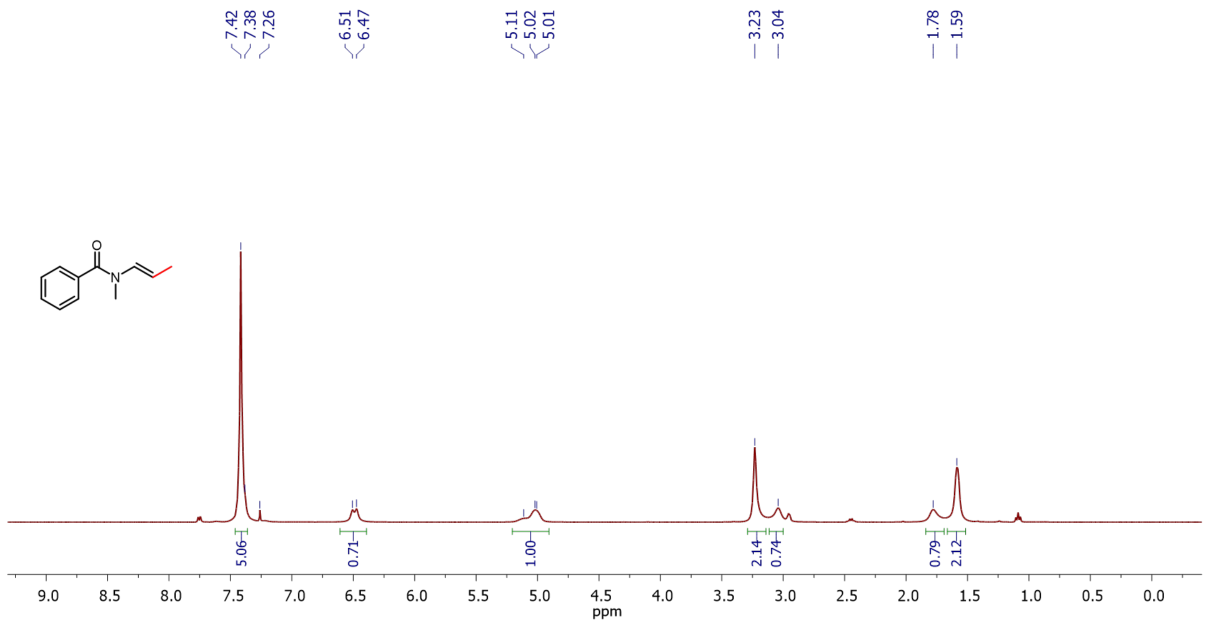

Figure S137. ^{1}H NMR spectrum (400 MHz) after gram scale isomerization (**4a**) in $CDCl_3$.

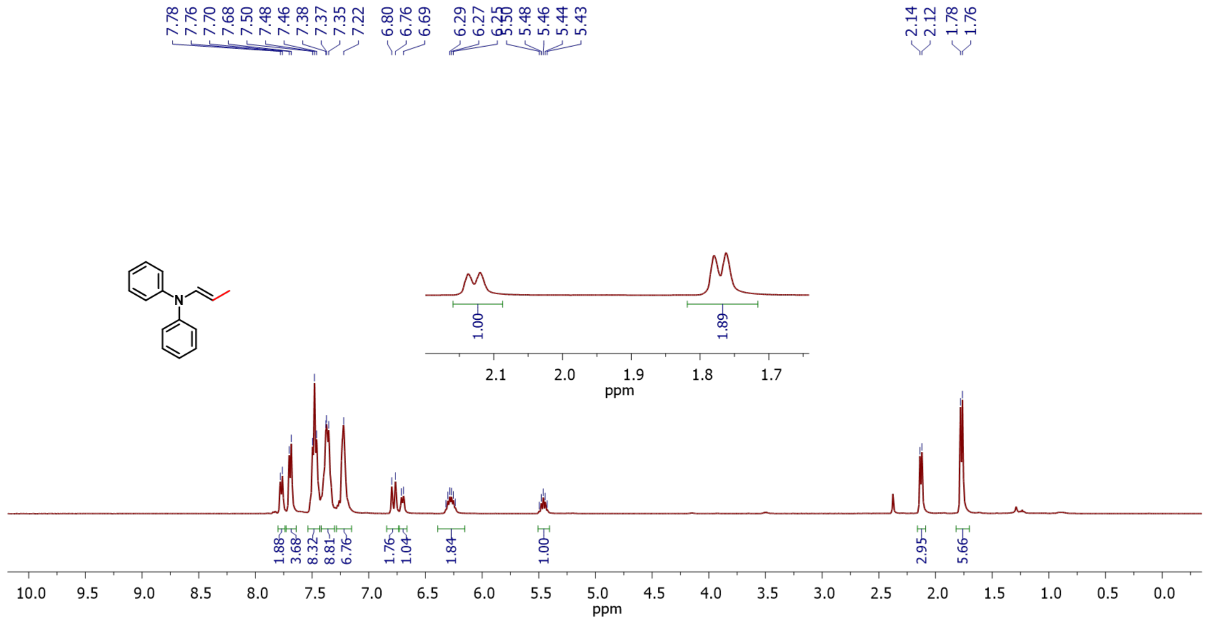

Figure S138. ^{1}H NMR spectrum (400 MHz) after gram scale isomerization (**8l**) in $CDCl_3$.

NMR of 2-phenylpyridine derivatives

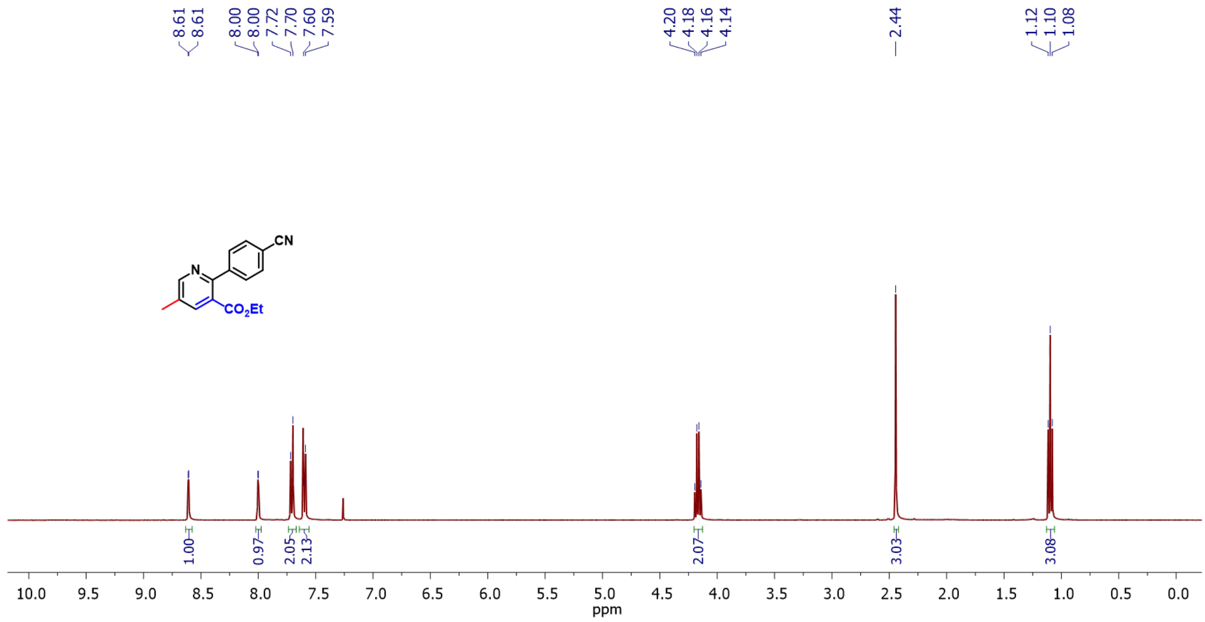

Figure S139. ^{1}H NMR spectrum (400 MHz) of **9a** in $CDCl_3$.

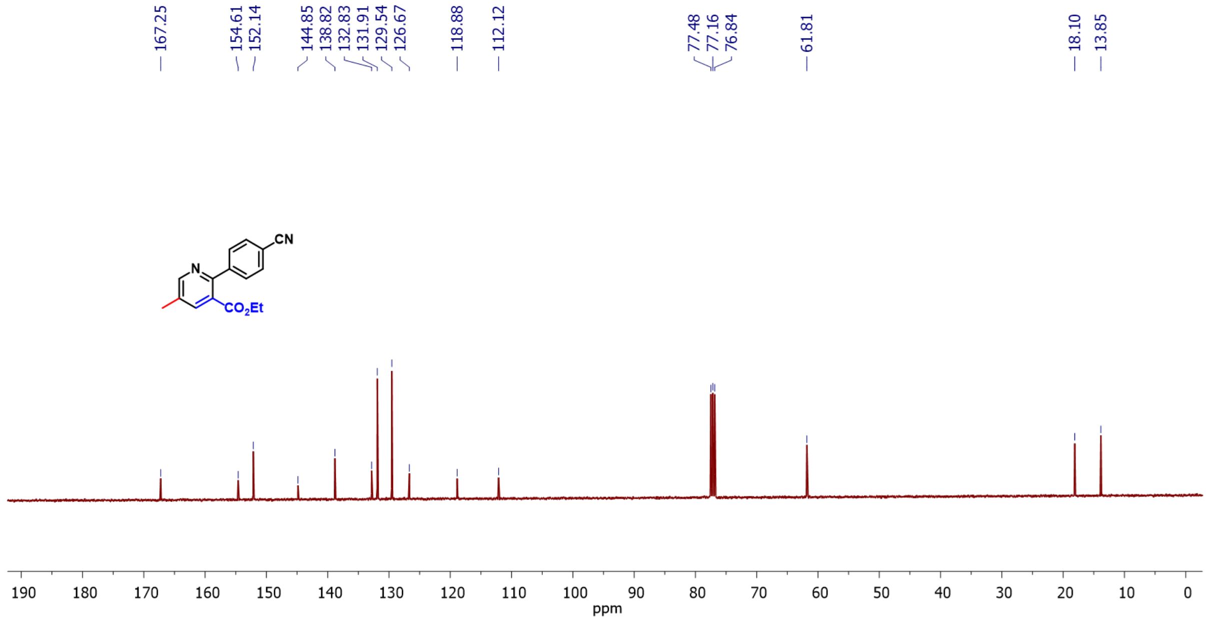

Figure S140. ^{13}C {^{1}H} NMR spectrum (101 MHz) of **9a** in $CDCl_3$.

Compound Spectrum SmartFormula Report

Analysis Info

Analysis Name	D:\Data\Graham\Dr_5233000001.d	Acquisition Date	10/12/2023 11:44:11
Method	APCI_pos_SolidProbe.m	Operator	Larisa Panz
Sample Name	SRK 9a	Instrument	maXis impact 282001.00128
Comment			

Acquisition Parameter

Source Type	APCI	Ion Polarity	Positive	Set Nebulizer	1.2 Bar
Focus	Active	Set Capillary	3000 V	Set Dry Heater	120 °C
Scan Begin	50 m/z	Set End Plate Offset	-500 V	Set Dry Gas	1.5 l/min
Scan End	2000 m/z	Set Charging Voltage	2000 V	Set Divert Valve	Source
		Set Corona	5000 nA	Set APCI Heater	200 °C

+MS, 0.5min #30

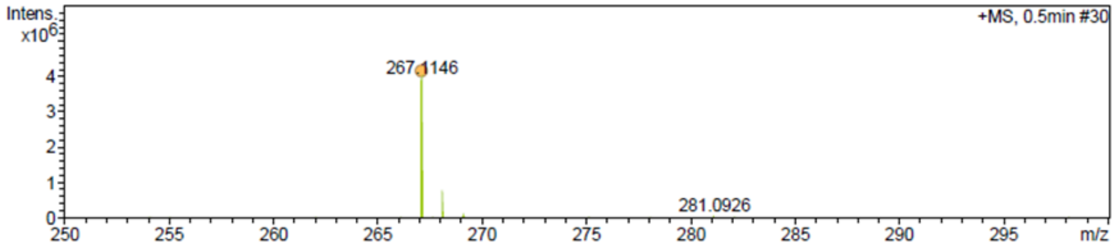

Meas. m/z	#	Ion Formula	m/z	err [ppm]	mSigma	# mSigma	Score	rdb	e⁻ Conf	N-Rule	|err| [mDa]
267.1146	1	C16H15N2O2	267.1134	-4.8	9.5	1	100.00	10.5	even	ok	1.3
	1	C16H15N2O2	267.1134	-4.8	9.5	1	100.00	10.5	even	ok	1.3

Figure S141. High resolution mass spectrum of **9a.**

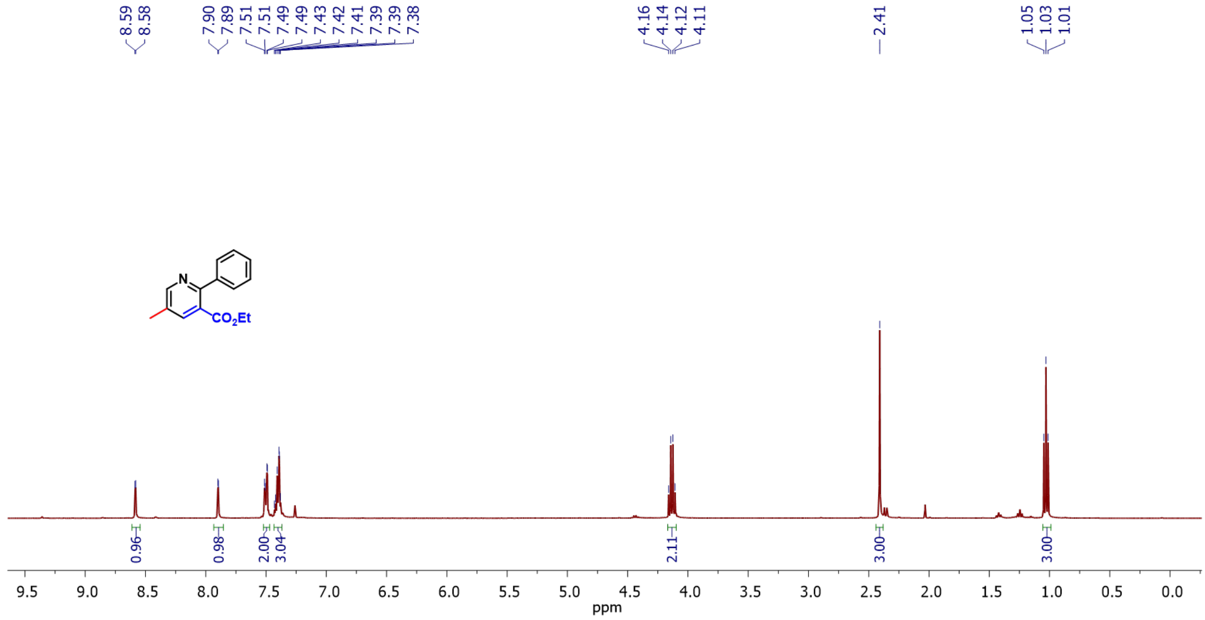

Figure S142. ^{1}H NMR spectrum (400 MHz) of **9b** in $CDCl_3$.

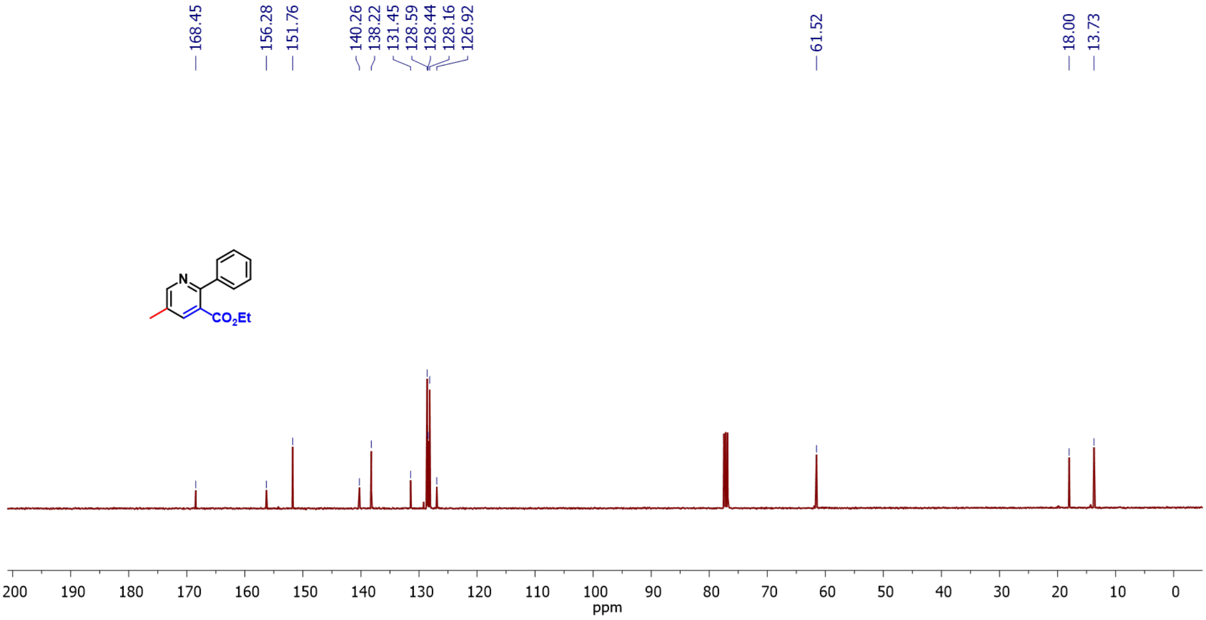

Figure S143. ^{13}C {^{1}H} NMR spectrum (101 MHz) of **9b** in $CDCl_3$.

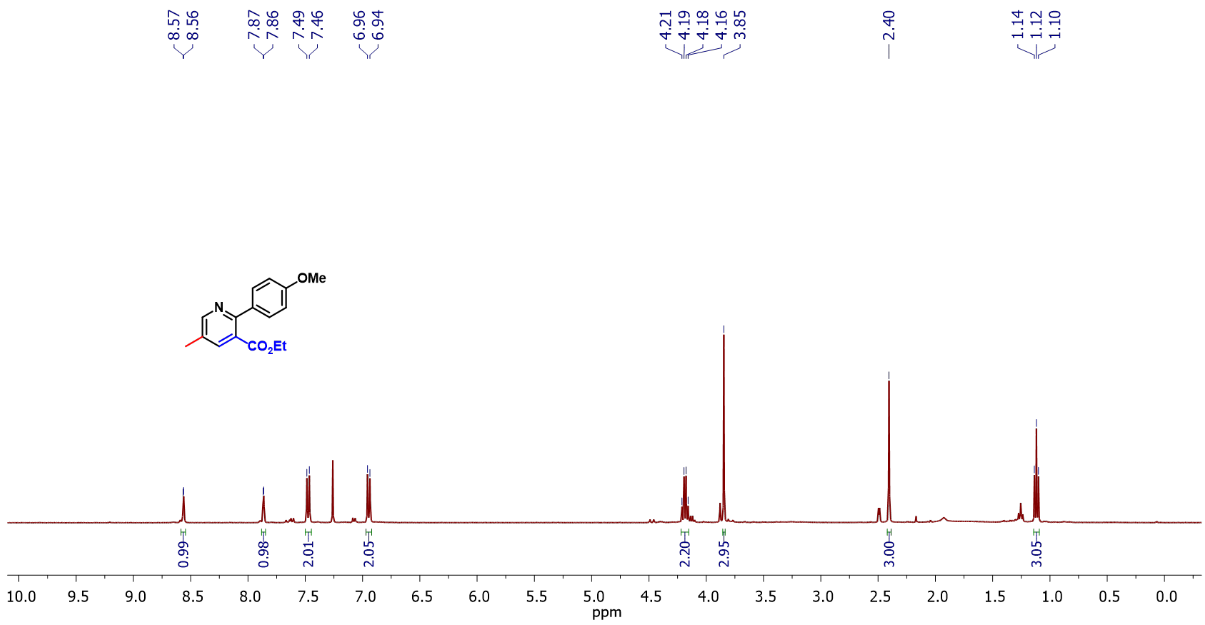

Figure S144. ^{1}H NMR spectrum (400 MHz) of **9c** in $CDCl_3$.

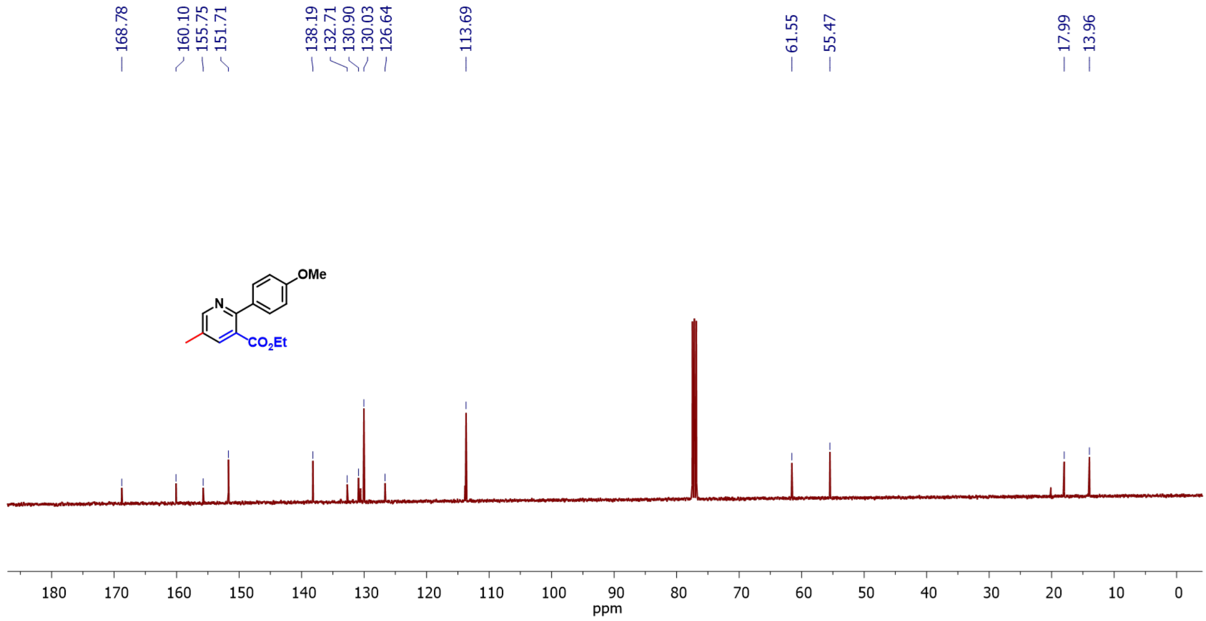

Figure S145. ^{13}C {^{1}H} NMR spectrum (101 MHz) of **9c** in $CDCl_3$.

Compound Spectrum SmartFormula Report

Analysis Info

Analysis Name	D:\Data\Graham\Dr_5234000001.d	Acquisition Date	10/12/2023 11:46:31
Method	APCI_pos_SolidProbe.m	Operator	Larisa Panz
Sample Name	SRK 9c	Instrument	maXis impact 282001.00128
Comment			

Acquisition Parameter

Source Type	APCI	Ion Polarity	Positive	Set Nebulizer	1.2 Bar
Focus	Active	Set Capillary	3000 V	Set Dry Heater	120 °C
Scan Begin	50 m/z	Set End Plate Offset	-500 V	Set Dry Gas	1.5 l/min
Scan End	2000 m/z	Set Charging Voltage	2000 V	Set Divert Valve	Source
		Set Corona	5000 nA	Set APCI Heater	200 °C

+MS, 0.4-0.4min #25-26

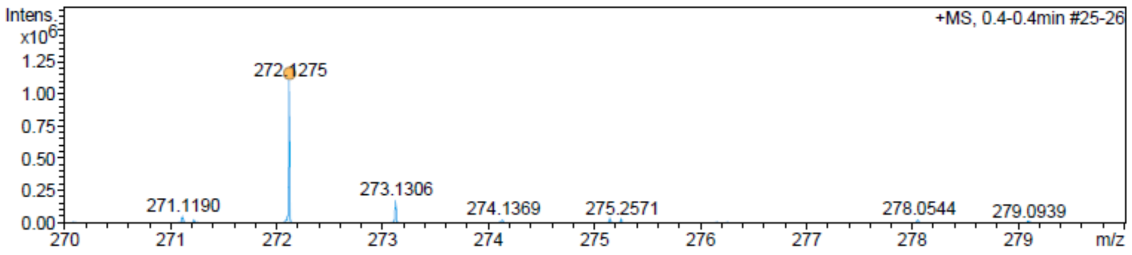

Meas. m/z	#	Ion Formula	m/z	err [ppm]	mSigma	# mSigma	Score	rdb	e⁻ Conf	N-Rule	|err| [mDa]
272.1275	1	C16H18NO3	272.1287	4.2	10.6	1	100.00	8.5	even	ok	1.1
	1	C16H18NO3	272.1287	4.2	10.6	1	100.00	8.5	even	ok	1.1

Figure S146. High resolution mass spectrum of **9c.**

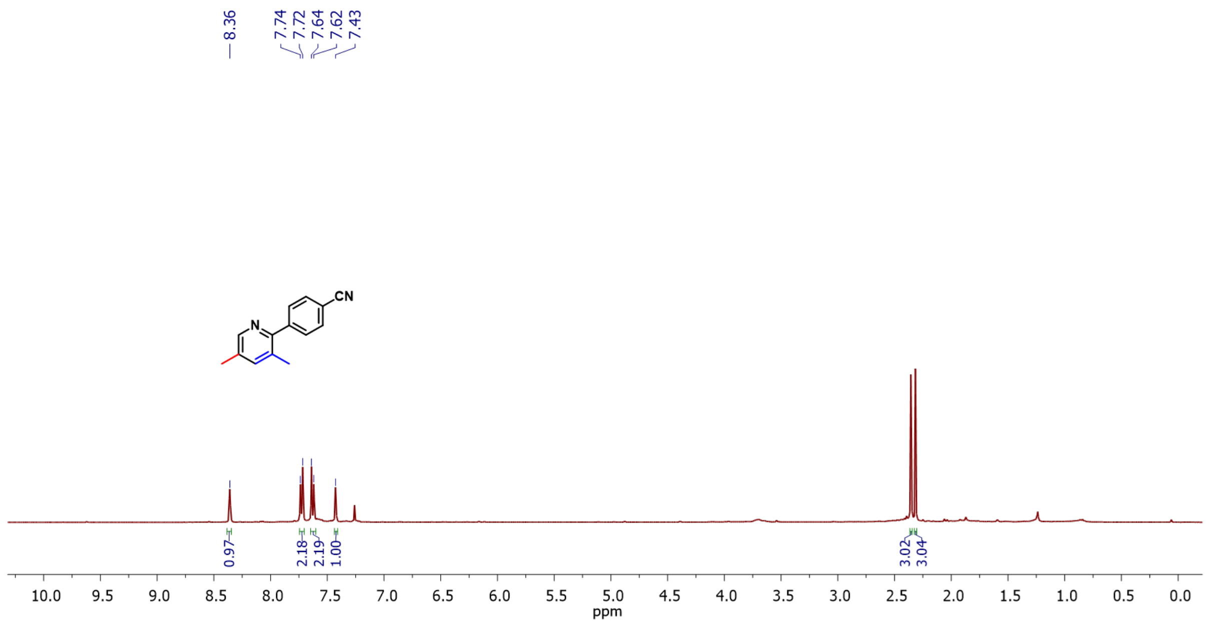

Figure S147. ^{1}H NMR spectrum (400 MHz) of **10a** in $CDCl_3$.

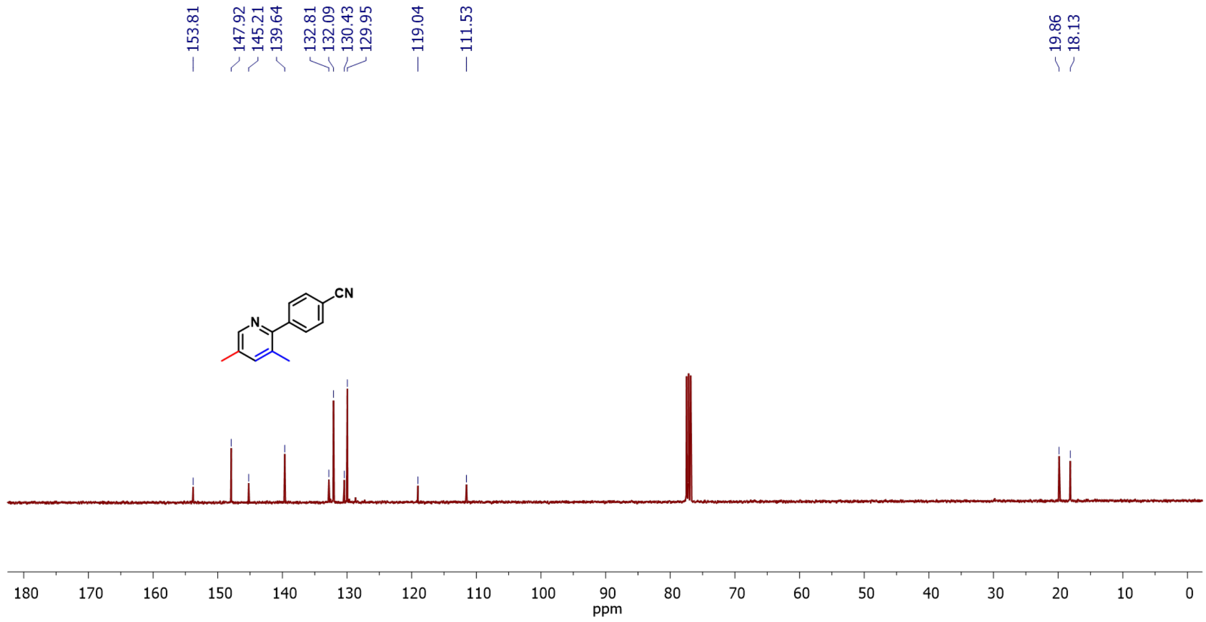

Figure S148. ^{13}C {^{1}H} NMR spectrum (101 MHz) of **10a** in $CDCl_3$.

Compound Spectrum SmartFormula Report

Analysis Info

Analysis Name	D:\Data\Graham\Dr_5235000001.d	Acquisition Date	10/12/2023 11:48:59
Method	APCI_pos_SolidProbe.m	Operator	Larisa Panz
Sample Name	SRK 10a	Instrument	maXis impact 282001.00128
Comment			

Acquisition Parameter

Source Type	APCI	Ion Polarity	Positive	Set Nebulizer	1.2 Bar
Focus	Active	Set Capillary	3000 V	Set Dry Heater	120 °C
Scan Begin	50 m/z	Set End Plate Offset	-500 V	Set Dry Gas	1.5 l/min
Scan End	2000 m/z	Set Charging Voltage	2000 V	Set Divert Valve	Source
		Set Corona	5000 nA	Set APCI Heater	200 °C

+MS, 0.3-0.3min #18-19

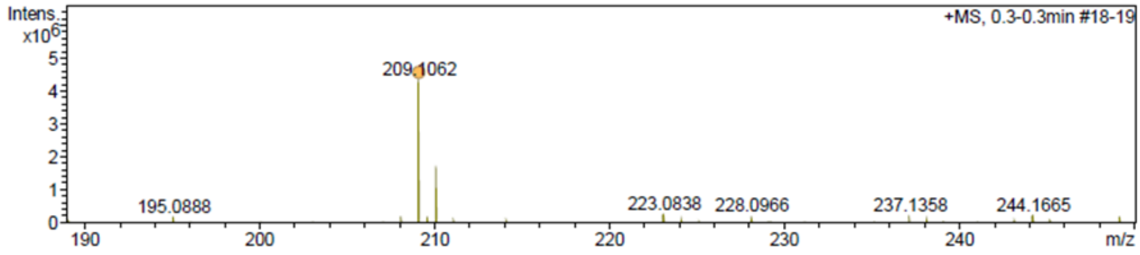

Meas. m/z	#	Ion Formula	m/z	err [ppm]	mSigma	# mSigma	Score	rdb	e⁻ Conf	N-Rule	|err| [mDa]
209.1062	1	C14H13N2	209.1079	8.0	135.8	1	100.00	9.5	even	ok	1.7
	2	C10H13N2O3	209.0926	-65.0	160.1	2	0.00	5.5	even	ok	13.6
	3	C6H13N2O6	209.0774	-138.0	184.3	3	0.00	1.5	even	ok	28.8
	1	C14H13N2	209.1079	8.0	135.8	1	100.00	9.5	even	ok	1.7
	2	C10H13N2O3	209.0926	-65.0	160.1	2	0.00	5.5	even	ok	13.6
	3	C6H13N2O6	209.0774	-138.0	184.3	3	0.00	1.5	even	ok	28.8

Figure S149. High resolution mass spectrum of **10a.**

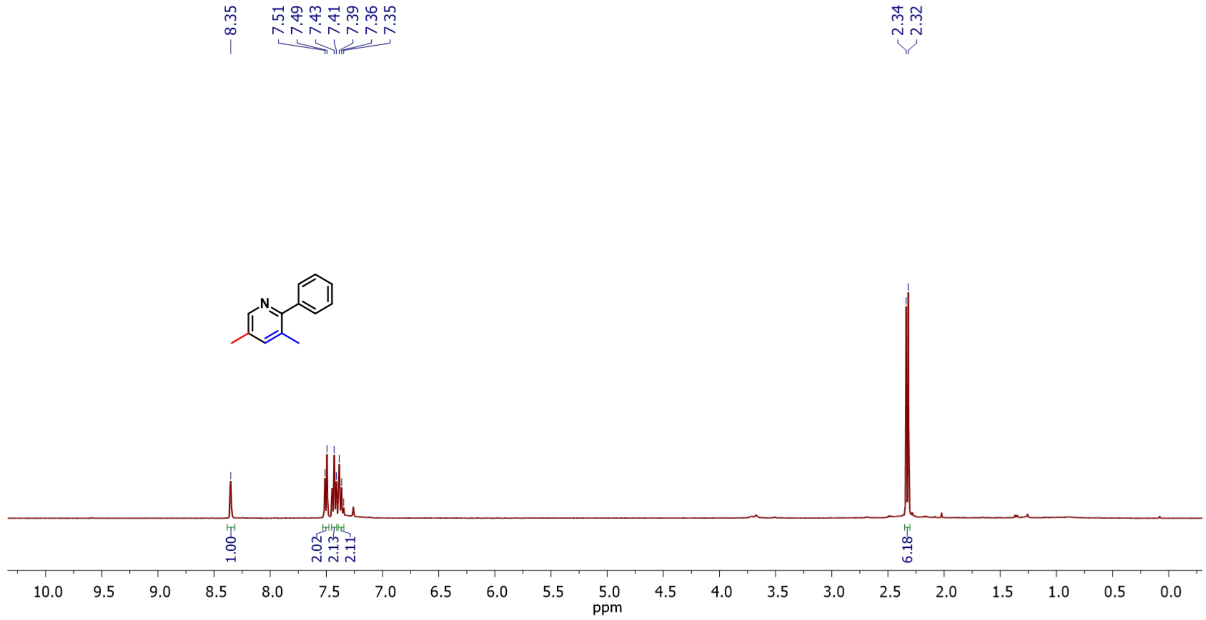

Figure S150. ^{1}H NMR spectrum (400 MHz) of **10b** in $CDCl_3$.

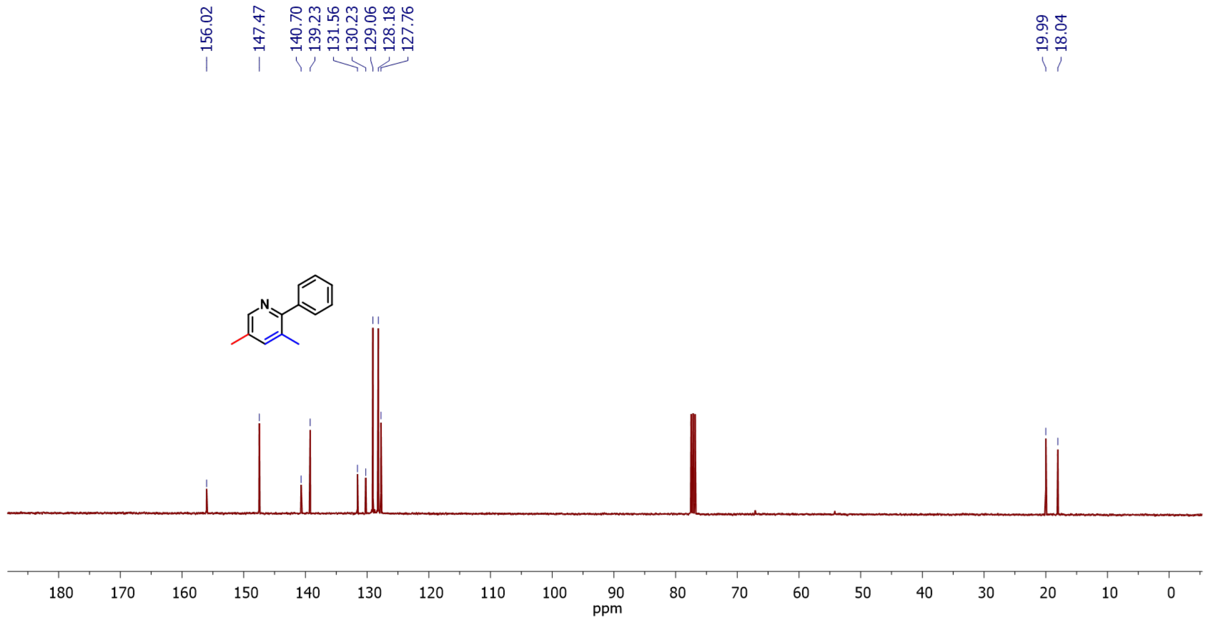

Figure S151. ^{13}C {^{1}H} NMR spectrum (101 MHz) of **10b** in $CDCl_3$.

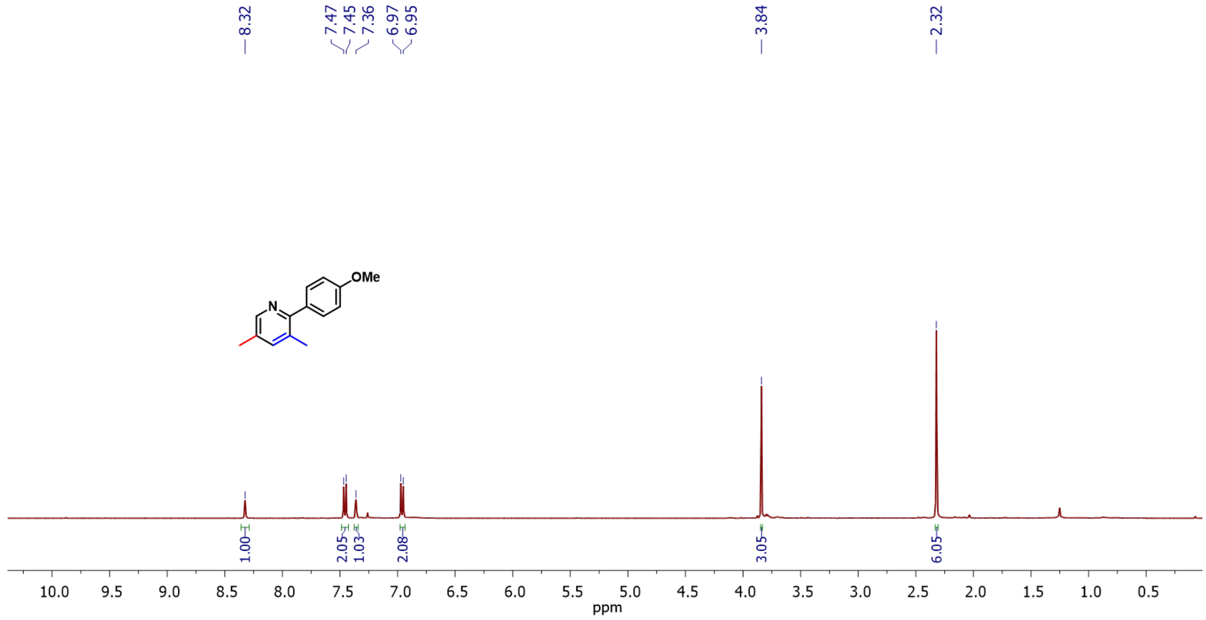

Figure S152. ^{1}H NMR spectrum (400 MHz) of **10c** in $CDCl_3$.

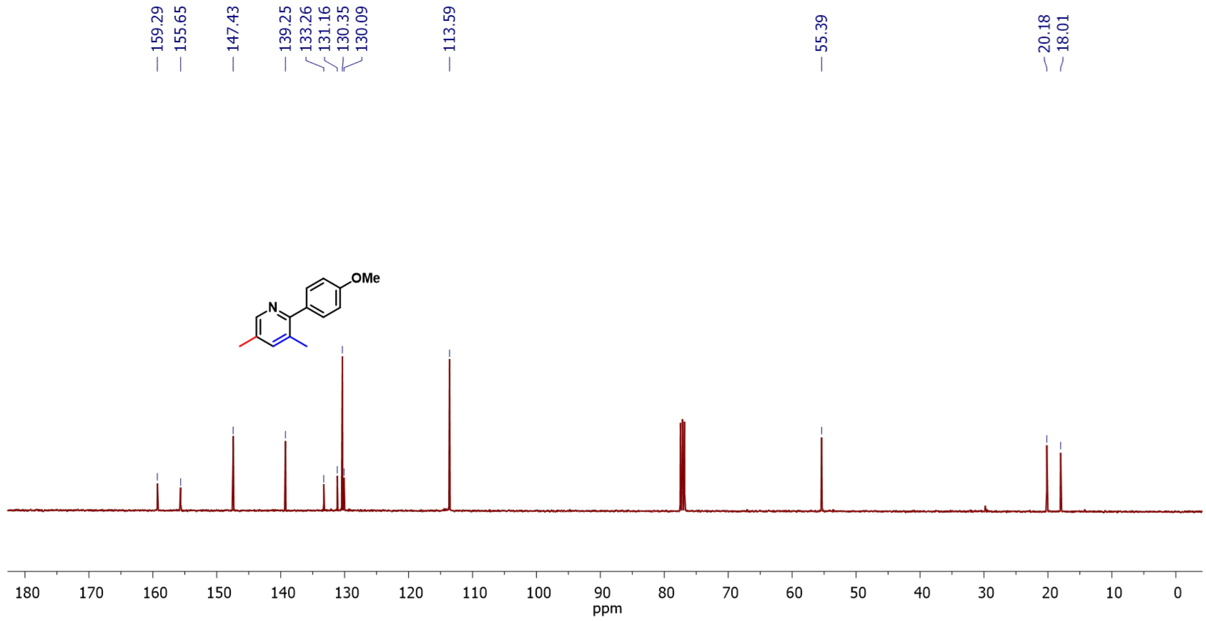

Figure S153. ^{13}C {^{1}H} NMR spectrum (101 MHz) of **10c** in $CDCl_3$.

One-pot isomerization.

One-pot isomerization of N-allylamine and N-allylaldimine with Co-Me as catalyst.

Inside the glovebox, a J-Young tube was charged with N-morpholine allylamine (19 mg, 0.15 mmol), N-allylbenzaldimine (22 mg, 0.15 mmol) and **Co-Me** (125 µL, 0.0075 mmol (2.5 mol%), from a 0.06M stock solution prepared in toluene-d_8) was added. To the mixture was added additional toluene-d_8 to make total volume 400 µL. The tube was sealed and heated in an oil bath and the progress of the reaction was monitored by ^{1}H NMR spectroscopy at different time interval.

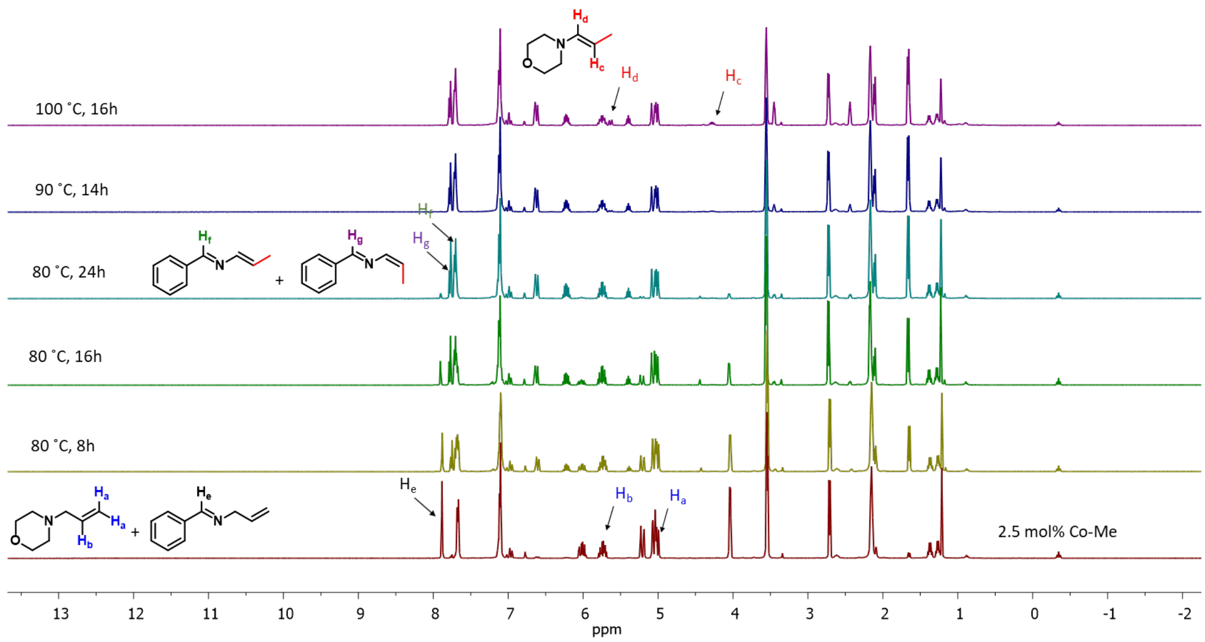

One-pot sequential isomerization of N-allylamine and N-allylaldimine with Co-Me as catalyst. Inside the glovebox, a J-Young tube was charged with N-morpholine allylamine (19 mg, 0.15 mmol) and **Co-Me** (125 μL, 0.0075 mmol (2.5 mol%), from a 0.06M stock solution prepared in toluene-d_8) was added. To the mixture was added additional toluene-d_8 to make total volume 400 μL. The tube was sealed and heated in an oil bath at 80 ˚C for 8h then the tube was transferred to glovebox. To the mixture was added N-allylbenzaldimine (22 mg, 0.15 mmol), whereafter the tube was sealed and heated in an oil bath at 80 or 90 ˚C for 22h and the progress of the reaction was monitored by ^{1}H NMR spectroscopy at different time interval.

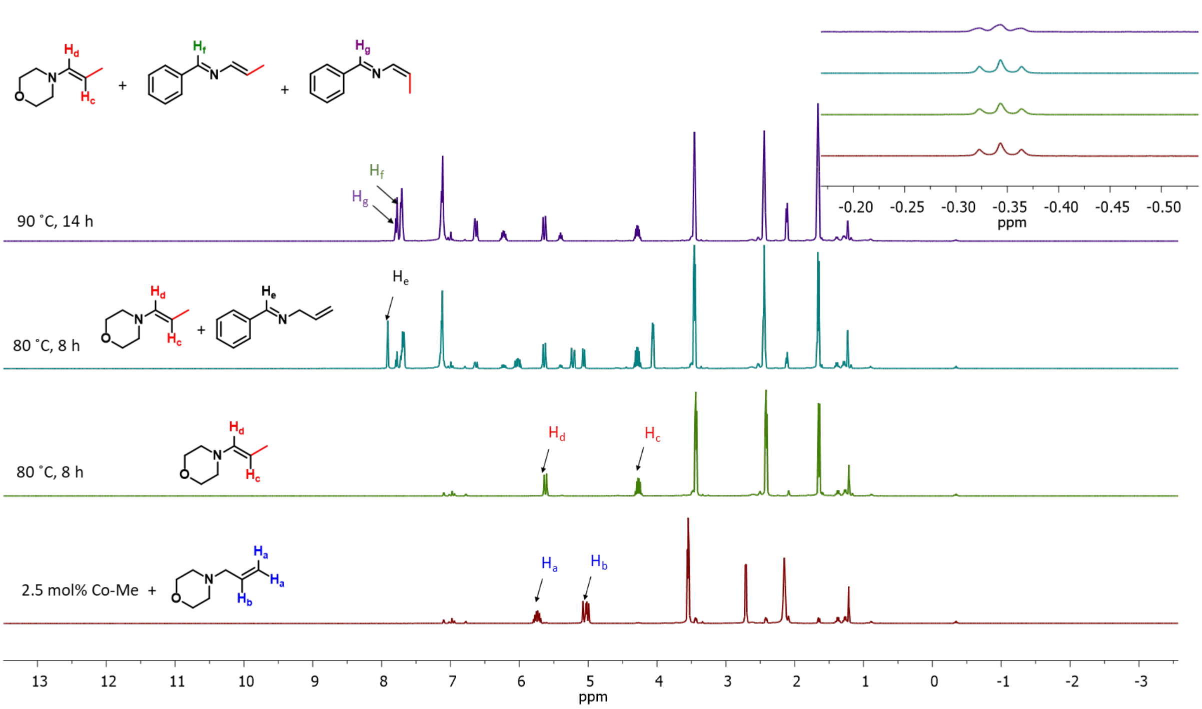

References

1. S. Garhwal, S. Raje, K. Młodzikowska-Pieńko, T. S. Mohammed, R. Rafaeli, N. Fridman, L. J. W. Shimon, R. Gershoni-Poranne and G. de Ruiter, *Submitted*, 2023.
2. R. Li, X. Wang, Z. Wei, C. Wu and F. Shi, *Org. Lett.*, 2013, **15**, 4366-4369.
3. H. Liang, L. Bao, Y. Du, Y. Zhang, S. Pang and C. Sun, *Synlett*, 2017, **28**, 2675-2679.
4. B. Alcaide, P. Almendros and J. M. Alonso, *Chem. - Eur. J.*, 2003, **9**, 5793-5799.
5. X. Zhao, D. Liu, H. Guo, Y. Liu and W. Zhang, *J. Am. Chem. Soc.*, 2011, **133**, 19354-19357.
6. Z. Chen, M. Tan, C. Shan, X. Yuan, L. Chen, J. Shi, Y. Lan and Y. Li, *Angew. Chem., Int. Ed.*, 2022, **61**, e202212160.
7. W. Xu, X. Cong, K. An, S.-J. Lou, Z. Li, M. Nishiura, T. Murahashi and Z. Hou, *Angew. Chem., Int. Ed.*, 2022, **61**, e202210624.
8. J. L. M. Matos, S. A. Green, Y. Chun, V. Q. Dang, R. G. Dushin, P. Richardson, J. S. Chen, D. W. Piotrowski, B. M. Paegel and R. A. Shenvi, *Angew. Chem., Int. Ed.*, 2020, **59**, 12998-13003.
9. G. Kundu, T. Sperger, K. Rissanen and F. Schoenebeck, *Angew. Chem., Int. Ed.*, 2020, **59**, 21930-21934.
10. V. Cadierno, J. Gimeno and N. Nebra, *Chem. - Eur. J.*, 2007, **13**, 6590.
11. D. Olivieri, R. Tarroni, N. Della Ca, R. Mancuso, B. Gabriele, G. Spadoni and C. Carfagna, *ChemCatChem*, 2022, **14**, e202101923.
12. B. M. Trost, S. Mahapatra and M. Hansen, *Angew. Chem., Int. Ed.*, 2015, **54**, 6032-6036.
13. A. R. Ickes, S. C. Ensign, A. K. Gupta and K. L. Hull, *J. Am. Chem. Soc.*, 2014, **136**, 11256-11259.
14. R. S. Klausen, C. R. Kennedy, A. M. Hyde and E. N. Jacobsen, *J. Am. Chem. Soc.*, 2017, **139**, 12299-12309.
15. H. Tafazolian and J. A. R. Schmidt, *Catal. Sci. Technol.*, 2016, **6**, 685-689.
16. J.-M. Huang, J.-F. Zhang, Y. Dong and W. Gong, *J. Org. Chem.*, 2011, **76**, 3511-3514.
17. Z. Chen, B. Lu, Z. Ding, K. Gao and N. Yoshikai, *Organic Letters*, 2013, **15**, 1966-1969.
18. B. Alcaide, P. Almendros, J. M. Alonso and M. F. Aly, *Org. Lett.*, 2001, **3**, 3781-3784.
19. K. Tani, T. Yamagata, S. Akutagawa, H. Kumobayashi, T. Taketomi, H. Takaya, A. Miyashita, R. Noyori and S. Otsuka, *J. Am. Chem. Soc.*, 1984, **106**, 5208.
20. S. Krompiec, M. Pigulla, M. Krompiec, B. Marciniec and D. Chadyniak, *J. Mol. Catal. A: Chem.*, 2005, **237**, 17-25.
21. Y. Lei, J. Huang and W. Zhao, *Org. Lett.*, 2021, **23**, 7797-7802.
22. S. D. Worley, K. G. Taylor, B. Venugopalan and M. S. Clark, Jr., *Tetrahedron*, 1978, **34**, 833.
23. R. Grigg and P. J. Stevenson, *Synthesis*, 1983, DOI: 10.1055/s-1983-30603, 1009.
24. C. K. Govindan and G. Taylor, *J. Org. Chem.*, 1983, **48**, 5348-5354.
25. S. Krompiec, B. Marcol, D. Zych, A. Kurpanik, W. Danikiewicz, M. Matussek and N. Kuznik, *ChemistrySelect*, 2017, **2**, 6717-6727.
26. G. I. Georg, P. He, J. Kant and Z. J. Wu, *J. Org. Chem.*, 1993, **58**, 5771.
27. J. M. I. Serviano, E. J. T. Phipps and P. L. Holland, *J. Org. Chem.*, 2023, **88**, 3277-3281.
28. K. K. Toh, A. Biswas, Y.-F. Wang, Y. Y. Tan and S. Chiba, *J. Am. Chem. Soc.*, 2014, **136**, 6011-6020.
29. K. Okamoto, K. Sasakura, T. Shimbayashi and K. Ohe, *Chem. Lett.*, 2016, **45**, 988-990.
